# Supplementary material for: Spatial colocalization and molecular crosstalk of myofibroblastic CAFs and tumor cells shape lymph node metastasis in oral squamous cell carcinoma
Source: PLoS Genet. 2025 Sep 4;21(9):e1011791. doi: 10.1371/journal.pgen.1011791 (PMC12410789; doi:10.1371/journal.pgen.1011791)
Supplement: S1 Methods — (PDF) [file pgen.1011791.s001.pdf]

S1 METHODS

Bulk OSCC data analysis inclusion criteria

We reviewed 234 cases of oral cancer from TCGA (RRID:SCR_003193) according to the bulk OSCC data analysis inclusion criteria, and 201 HPV-negative patients were selected (S1A Fig). The exclusion criteria were age less than 18 years at diagnosis, pathological diagnosis of basaloid squamous cell carcinoma, HPV-positive status, and previous malignancy. First, we excluded 15 of the 234 patients for whom the WES or RNA-seq data necessary for our analysis were unavailable. We then reviewed the patients' histopathological results and excluded 5 patients with the pathological diagnosis of basaloid squamous cell carcinoma (TCGA-CQ-A4CI, TCGA-QK-A8Z7, TCGA-UF-A71E, TCGA-HD-8314, and TCGA-P3-A5QE). Further, we excluded 3 patients for whom HPV status following p16 testing was positive (TCGA-QK-AA3K, TCGA-CN-A6V6, and TCGA-CN-A6UY) because head and neck cancers caused by HPV are clinically and molecularly distinct [1,2]. Lastly, we excluded 7 patients with a prior malignancy (TCGA-HD-8635, TCGA-BA-4077, TCGA-BA-4075, TCGA-HD-8634, TCGA-CQ-A4C7, TCGA-H7-A6C4, and TCGA-CX-7219) and 3 with unknown American Joint Committee on Cancer pathological stages (TCGA-T2-A6WZ, TCGA-WA-A7GZ, and TCGA-CV-A45T).

Single-cell transcriptome data processing

The OSCC scRNA-seq dataset (GSE103322)[3], which contains 5,884 cells from 17 of the 18 cases (no transcriptome data were obtained from 1 patient) was used (S2A Fig). Five patients had no LNM (TNM stage: N0) and 12 had LNM (TNM stage: N1-N3), of whom 5 had both primary and metastatic sites. We organized the sample IDs and cell-type annotations on the basis of the

source data annotation [3] using Scanpy v1.7.1 [4] (RRID:SCR_018139) (Table S3; S2F-2H Fig). Patients without LNM were identified as HNSCC8, HNSCC12, HNSCC16, HNSCC17, and HNSCC22. The gene-expression matrix excluded samples labeled as HNSCC with an unknown sample name, and sample names with LNM (HN25, HN26, HN28, HNSCC20, HNSCC25, HNSCC26, HNSCC28, and HNSCC5) were extracted. These samples underwent a name change from HN or HNSCC to OSCC for standardization of the sample identifiers. For instance, HN25 and HNSCC25, which shared the same Sample ID, were renamed as OSCC25. The TME of the OSCC scRNA-seq samples contained several cell types: cancer cells, fibroblasts, T cells, B cells, endothelial cells, dendritic cells, macrophages, mast cells, and monocytes (S2B-2D Fig). Previous research [3] classified fibroblasts into 3 groups: myofibroblasts, nonactivated resting fibroblasts, and activated CAFs, with the latter 2 showing similar transcriptomic patterns. In our analysis of the same dataset using Scanpy v1.7.1 [4] (RRID:SCR_018139), we discerned 2 predominant categories. The first cluster—expressing *ACTA2*, which encodes smooth muscle proteins—and *MYL9* contained myCAFs. Furthermore, myCAFs are known to express melanoma cell adhesion molecule (*MCAM*, also known as *CD146*), and transgelin (*TAGLN*) which is induced by *TGFβ* [5]. The second cluster, expressing matrix metallopeptidase 2 (*MMP2*) and platelet-resolved growth factor receptor alpha (*PDGFRA*), as well as markers such as fibroblast activation protein alpha (*FAP*) and podoplanin (*PDPN*), contained secretory/matrix-remodeling cancer-associated fibroblasts (sCAFs). Although a previous study identified these cells as CAFs [3], we introduced the term “sCAFs” on the basis of the cells’ transcriptomic functions [6–9] (Also see S2F-2H Fig).

Spatial transcriptome analysis case descriptions

Patient HUH001 (T2N3bM0, stage IVB)

Patient HUH001, a 48-year-old woman, was diagnosed with squamous cell carcinoma of the tongue (left side) after a biopsy was conducted at a general hospital. There was no previous history of malignancy and no concurrent cancer according to an upper gastrointestinal tract examination. Positron emission tomography and computed tomography (CT) findings showed fludeoxyglucose accumulation in several areas, including the left lateral tongue and specific lymph nodes (Fig 5B). Contrast-enhanced CT and magnetic resonance imaging scans revealed a 2.2-cm contrast effect on the tongue's left lateral border and rim-enhanced lymph nodes (approximately 2 cm; Fig 5B). After surgery, histopathological examination of the surgical specimens confirmed moderately differentiated squamous cell carcinoma with pattern of invasion type 4 and negative resection margins (S3A Fig). LNM was present (submental lymph nodes, n = 0/3; submandibular lymph nodes, n = 0/5; upper internal jugular lymph nodes, n =1/7; middle internal jugular lymph nodes, n = 2/3; lower internal jugular lymph nodes, n = 0/5; accessory nerve nodes, n = 0/5).

Patient HUH002 (T2N0M0, stage II)

Patient HUH002, 61-year-old woman, sought medical help due to pain on the right lingual border. A subsequent biopsy confirmed squamous cell carcinoma of the tongue's right side. Positron emission tomography and CT scans revealed fludeoxyglucose accumulation at the right lateral tongue, while contrast-enhanced CT and magnetic resonance imaging scans revealed enlarged right lymph nodes (Fig 5B). After surgery, histopathological examination of the surgical

specimens identified moderately differentiated squamous cell carcinoma with pattern of invasion type 3 and negative resection margins (S3B Fig). There was no LNM (submental lymph nodes, *n* = 0/2; submandibular lymph nodes, *n* = 0/5; upper internal jugular lymph nodes, *n* = 0/6; middle internal jugular lymph nodes, *n* = 0/2).

Spatial validation cohort processing

To validate our findings, the spatial transcriptomics dataset was re-analyzed (source: GSE208253 [10]). Initially, cell-type deconvolution of spatial spots was performed using Tangram ([11,12]; mode='constrained', learning_rate=0.1). This process used an OSCC scRNA-seq dataset (GSE103322) as a reference, from which signature markers for 10 principal cell types were derived (Scanpy v1.7.1 [4], tl.rank_genes_groups, Wilcoxon method) and from which we identified 338 common genes for mapping. Spots predicted by Tangram deconvolution to contain at least 1 OSCC cell were selected as tumor regions for further analysis. Sample-specific batch effects within these tumor regions were corrected using scVI [13] (scvi-tools v0.14.5 [14]) on raw count data. Cellular and genetic filtering was applied (Scanpy pp.filter_cells: min_counts=500; pp.filter_genes: min_cells=3). Subsequently, unsupervised Leiden clustering (Scanpy tl.leiden, resolution=0.5) was performed on the scVI-integrated data. The resultant clusters were then characterized and annotated as tumor core, intermediate, or invasive tumor front via examination of the expression of the established DEGs specific to these regions, as reported in the original study [10].

Tongue SCC co-culture RNAseq data processing

Publicly available bulk RNA-sequencing data (source: GSE279481 [15]) from co-cultures of human HPV-negative tongue squamous cell carcinoma (tongue SCC; Cal33, Cal27) and cancer-associated fibroblast (CAF; 61137, 61162) cell lines were analyzed. Gene-expression values were processed using a log_2[TPM+1] scale where applicable (e.g., myCAF marker confirmation; *ACTA2, TAGLN, MYL9, TPM2, MCAM*; see S2H Fig for further details). *COL1A1* expression in CAFs (mono-culture vs. co-culture with tongue SCC cells) and *CD44* expression in tongue SCC cells (mono-culture vs. co-culture with CAFs) were compared. For correlation analysis, 17 ECM core genes were examined (*COL1A1, COL1A2, COL3A1, COL4A1, COL4A2, COL5A1, COL5A2, COL5A3, COL6A1, COL6A2, COL6A3, COL7A1, COL13A1, FN1, LAMA3, LAMB1, LAMB3*). These were selected based on the literature [15–28] and confirmed expression in myCAFs (e.g., log_2[TPM+1] > 1.0). After aggregating gene expression by condition, log_2 fold changes (FCs) were calculated for these ECM genes in myCAFs (co-culture vs. mono-culture) and for *CD44* in tongue SCC cells (co-culture vs. mono-culture). Pearson correlation coefficients (rs) were then determined between the FCs of ECM genes in myCAFs and the FCs of *CD44* in tongue SCC cells.

Hierarchical Bayesian modeling of the ECM-CD44 axis

To quantitatively assess the overall relationship between myCAF-derived ECM gene expression and *CD44* expression in OSCC cells, while accounting for variability across individual ECM genes and experimental conditions (cell line pairings), a hierarchical Bayesian model was applied to the GSE279481 co-culture dataset [15]. ECM gene expression in myCAFs (denoted as

x) and *CD44* expression in OSCC cells (denoted as *y*) from mono- and co-culture experiments were standardized prior to modeling their relationship. The data was structured in a long format suitable for the model, including indicators for each specific ECM gene and experimental pair. The hierarchical Bayesian model structure was defined (see S4R Fig for further details). The model was implemented using PyMC [29] v5.16.2. Posterior distributions of all parameters, including the population-level mean slope, gene-specific slopes, and pair-specific slopes, were estimated using the No-U-Turn Sampler (NUTS), typically with 4000 tuning (warm-up) steps followed by 4000 drawing steps per chain (target_accept=0.9). The posterior distribution was used to evaluate the overall relationship between myCAF-derived ECM gene expression and *CD44* levels in OSCC cells.

Mediation analysis of ECM-CD44 axis via the integrin and syndecan pathways

To investigate the indirect effects of myCAF-derived ECM signaling (predictor, X) on OSCC cell *CD44* expression (outcome, Y) via specific receptor pathways, a mediation analysis was conducted using data from the GSE279481 [15]. Variables were constructed from $\log_2$ fold-changes (FC; co-culture vs. mono-culture). The predictor (X) was defined as the first principal component (PC1) of FCs of selected collagen gene expressions in CAFs. The outcome (Y) was the FC of *CD44* expression in OSCC cells. Two mediator pathways in OSCC cells were examined: the integrin pathway (M1), represented by the PC1 of FCs of *ITGAs* and *ITGBs* gene expressions; and the syndecan pathway (M2), represented by the mean of standardized FCs of *SDC1* and *SDC4* gene expressions. All variables (X, Y, M1, M2) were standardized prior to analysis. For each pathway (X→M1→Y and X→M2→Y), the path coefficients (β_{XM} for X→M; β_{MY} for M→Y) were first estimated using an ordinary least squares (OLS) regression by fitting

models of the form M~X and Y~M. The indirect effect for each pathway was calculated as the product $\beta_{XM} \cdot \beta_{MY}$. To assess the statistical significance and stability of these indirect effects, a bootstrap estimation (n = 10,000 resamples) was performed, which yielded mean indirect effects and 95% CIs.

Microarray data processing

Publicly available microarray datasets [30] GSE178153 and GSE178154 were processed. Raw microarray CEL files underwent background correction and normalization using the robust multi-array average (RMA) method. Probes were then annotated to gene symbols using relevant annotation databases (huex10sttranscriptcluster.db and hta20transcriptcluster.db). Probes exhibiting low variance (in the bottom 20%) across samples were removed. Subsequently, batch effects were corrected using the pyComBat algorithm [31,32]. For comparative analyses, gene expression values were Z-score—standardized per gene across all samples.

Spatial pseudotime trajectory analyses

To model cellular progression using spatial transcriptomics data, trajectory analyses were conducted using 2 complementary approaches. For quantitative comparisons and per-cluster pseudotime visualization, pseudotime values for each spatial spot were computed with stLearn[33]. Specifically, the trajectory was rooted in primary tumor cluster 3 (presumed to represent an early state) using stLearn.spatial.trajectory.set_root, and the pseudotime was subsequently calculated via stLearn.spatial.trajectory.pseudotime (using PCA representation with

eps = 50). Separately, for visualizing inter-cluster relationships and the overall trajectory structure, the diffusion pseudotime (DPT) was calculated using Scanpy. This DPT calculation (sc.tl.dpt function, e.g., n_dcs=10) was also rooted in primary tumor cluster 3. Partition-based graph abstraction (PAGA; sc.tl.paga), based on Leiden cluster assignments, was then used to generate and visualize a graph of cluster connectivities overlaid with these DPT values.

Risk score validation

We identified 221 cases of oral cancer in The Cancer Genome Atlas (TCGA, RRID:SCR_003193) according to the GDC sample sheet from the bulk RNA-seq analysis, and 220 patients were selected (S6A Fig). We then excluded 5 patients with the pathological diagnosis of basaloid squamous cell carcinoma (TCGA-CQ-A4CI, TCGA-QK-A8Z7, TCGA-UF-A71E, TCGA-HD-8314, and TCGA-P3-A5QE). The exclusion criteria were perioperative death (within 30 days post-operation), undergoing neoadjuvant chemoradiotherapy, positive margins, and recurrent lesions. Among the 215 OSCC patients in TCGA, 1 was excluded due to perioperative death (TCGA-CV-A463). Thus, we evaluated the association between 298 upregulated DEGs identified in clusters of metastatic primary tumor sited from the spatial transcriptome analysis of matched LNM samples and OS in 214 patients and between 298 upregulated DEGs and DSS in 209 patients, excluding 5 who died of non-OSCC causes (TCGA-CQ-A4C7, TCGA-CV-7102, TCGA-CV-7406, TCGA-CV-A45V, TCGA-P3-A6T7). For GSE41613, 1 out of 97 OSCC patients was excluded due to perioperative death (GSM1020187). Thus, we evaluated OS in 96 patients and DSS in 76 patients, excluding 20 who died of non-OSCC causes (GSM1020102, GSM1020111, GSM1020115, GSM1020116, GSM1020117,

GSM1020122, GSM1020126, GSM1020128, GSM1020129, GSM1020135, GSM1020137, GSM1020143, GSM1020152, GSM1020167, GSM1020178, GSM1020181, GSM1020182, GSM1020183, GSM1020184, GSM1020190). For GSE42743, we analyzed 71 OSCC patients from 103 samples. Among the 32 non-OSCC samples, 29 were from normal tissue (GSM1049106, GSM1049108, GSM1049110, GSM1049112, GSM1049113, GSM1049115, GSM1049118, GSM1049123, GSM1049126, GSM1049127, GSM1049130, GSM1049132, GSM1049135, GSM1049138, GSM1049140, GSM1049142, GSM1049144, GSM1049148, GSM1049152, GSM1049153, GSM1049154, GSM1049157, GSM1049159, GSM1049161, GSM1049163, GSM1049165, GSM1049166, GSM1049168, GSM1049170), and 3 were from the oropharynx (GSM1049173, GSM1049175, GSM1049179). We excluded 21 patients from the 71 OSCC patients: 5 due to perioperative death (GSM1049121, GSM1049131, GSM1049134, GSM1049160, GSM1049172), 4 who received neoadjuvant chemoradiotherapy (GSM1049081, GSM1049089, GSM1049100, GSM1049101), and 12 with recurrence (GSM1049082, GSM1049085, GSM1049104, GSM1049107, GSM1049109, GSM1049120, GSM1049128, GSM1049136, GSM1049137, GSM1049145, GSM1049147, GSM1049150). Consequently, OS was assessed in 50 patients and DSS was assessed in 37 patients, excluding 13 who died of non-OSCC causes (GSM1049079, GSM1049084, GSM1049087, GSM1049088, GSM1049090, GSM1049096, GSM1049097, GSM1049111, GSM1049129, GSM1049155, GSM1049158, GSM1049174, GSM1049181).

Supplementary references

1. Sabatini ME, Chiocca S. Human papillomavirus as a driver of head and neck cancers. Br J Cancer. 2020;122: 306–314.

2. Leemans CR, Snijders PJF, Brakenhoff RH. The molecular landscape of head and neck cancer. Nat Rev Cancer. 2018;18: 269–282.

3. Puram SV, Tirosh I, Parikh AS, Patel AP, Yizhak K, Gillespie S, et al. Single-Cell Transcriptomic Analysis of Primary and Metastatic Tumor Ecosystems in Head and Neck Cancer. Cell. 2017;171: 1611-1624.e24.

4. Wolf FA, Angerer P, Theis FJ. SCANPY: large-scale single-cell gene expression data analysis. Genome Biol. 2018;19: 15.

5. Elsafadi M, Manikandan M, Dawud RA, Alajez NM, Hamam R, Alfayez M, et al. Transgelin is a TGFβ-inducible gene that regulates osteoblastic and adipogenic differentiation of human skeletal stem cells through actin cytoskeleston organization. Cell Death Dis. 2016;7: e2321.

6. Li H, Courtois ET, Sengupta D, Tan Y, Chen KH, Goh JJL, et al. Reference component analysis of single-cell transcriptomes elucidates cellular heterogeneity in human colorectal tumors. Nat Genet. 2017;49: 708–718.

7. Elyada E, Bolisetty M, Laise P, Flynn WF, Courtois ET, Burkhart RA, et al. Cross-species single-cell analysis of pancreatic ductal adenocarcinoma reveals antigen-presenting cancer-associated fibroblasts. Cancer Discov. 2019;9: 1102–1123.

8. Lavie D, Ben-Shmuel A, Erez N, Scherz-Shouval R. Cancer-associated fibroblasts in the single-cell era. Nat Cancer. 2022;3: 793–807.

9. McAndrews KM, Chen Y, Darpolor JK, Zheng X, Yang S, Carstens JL, et al. Identification of functional heterogeneity of carcinoma-associated fibroblasts with distinct IL6-mediated therapy resistance in pancreatic cancer. Cancer Discov. 2022;12: 1580–1597.

10. Arora R, Cao C, Kumar M, Sinha S, Chanda A, McNeil R, et al. Spatial transcriptomics reveals distinct and conserved tumor core and edge architectures that predict survival and targeted therapy response. Nat Commun. 2023;14: 5029.

11. Biancalani T, Scalia G, Buffoni L, Avasthi R, Lu Z, Sanger A, et al. Deep learning and alignment of spatially resolved single-cell transcriptomes with Tangram. Nat Methods. 2021;18: 1352–1362.

12. Li H, Zhou J, Li Z, Chen S, Liao X, Zhang B, et al. A comprehensive benchmarking with practical guidelines for cellular deconvolution of spatial transcriptomics. Nat Commun. 2023;14: 1548.

13. Lopez R, Regier J, Cole MB, Jordan MI, Yosef N. Deep generative modeling for single-cell transcriptomics. Nat Methods. 2018;15: 1053–1058.

14. Gayoso A, Lopez R, Xing G, Boyeau P, Valiollah Pour Amiri V, Hong J, et al. A Python library for probabilistic analysis of single-cell omics data. Nat Biotechnol. 2022;40: 163–166.

15. Waas M, Karamboulas C, Wu BZ, Khan S, Poon S, Meens J, et al. Molecular correlates for HPV-negative head and neck cancer engraftment prognosticate patient outcomes. Nat Commun. 2024;15: 10869.

16. Choi J-H, Lee B-S, Jang JY, Lee YS, Kim HJ, Roh J, et al. Single-cell transcriptome profiling of the stepwise progression of head and neck cancer. Nat Commun. 2023;14: 1055.

17. Sun Y, Chen Y, Zhao H, Wang J, Liu Y, Bai J, et al. Lactate-driven type I collagen deposition facilitates cancer stem cell-like phenotype of head and neck squamous cell carcinoma. iScience. 2024;27: 109340.

18. Chen C, Zhao S, Karnad A, Freeman JW. The biology and role of CD44 in cancer progression: therapeutic implications. J Hematol Oncol. 2018;11. doi:10.1186/s13045-018-0605-5

19. Knutson JR, Iida J, Fields GB, McCarthy JB. CD44/chondroitin sulfate proteoglycan and alpha 2 beta 1 integrin mediate human melanoma cell migration on type IV collagen and invasion of basement membranes. Mol Biol Cell. 1996;7: 383–396.

20. Hagedorn H, Schreiner M, Wiest I, Tubel J, Schleicher ED, Nerlich AG. Defective basement membrane in laryngeal carcinomas with heterogeneous loss of distinct components. Hum Pathol. 1998;29: 447–454.

21. Lim YC, Oh S-Y, Kim H. Cellular characteristics of head and neck cancer stem cells in type IV collagen-coated adherent cultures. Exp Cell Res. 2012;318: 1104–1111.

22. Liang X, Osman TA-H, Sapkota D, Neppelberg E, Lybak S, Liavaag PG, et al. Rapid adherence to collagen IV enriches for tumour initiating cells in oral cancer. Eur J Cancer. 2014;50: 3262–3270.

23. Fejza A, Camicia L, Poletto E, Carobolante G, Mongiat M, Andreuzzi E. ECM remodeling in squamous cell carcinoma of the aerodigestive tract: Pathways for cancer dissemination and emerging biomarkers. Cancers (Basel). 2021;13: 2759.

24. Wang H, Zhou H, Ni H, Shen X. COL11A1-driven epithelial-mesenchymal transition and stemness of pancreatic cancer cells induce cell migration and invasion by modulating the AKT/GSK-3β/Snail pathway. Biomolecules. 2022;12: 391.

25. Kumar KV, Hema KN. Extracellular matrix in invasion and metastasis of oral squamous cell carcinoma. J Oral Maxillofac Pathol. 2019;23: 10–16.

26. Patankar SR, Wankhedkar DP, Tripathi NS, Bhatia SN, Sridharan G. Extracellular matrix in oral squamous cell carcinoma: Friend or foe? Indian J Dent Res. 2016;27: 184–189.

27. Yang Y, Sun H, Yu H, Wang L, Gao C, Mei H, et al. Tumor-associated-fibrosis and active collagen-CD44 axis characterize a poor-prognosis subtype of gastric cancer and contribute to tumor immunosuppression. J Transl Med. 2025;23: 123.

28. Dong C, Zhao Y, Yang S, Jiao X. LINC00173 blocks GATA6-mediated transcription of COL5A1 to affect malignant development of oral squamous cell carcinoma. J Oral Pathol Med. 2023;52: 493–503.

29. Abril-Pla O, Andreani V, Carroll C, Dong L, Fonnesbeck CJ, Kochurov M, et al. PyMC: a modern, and comprehensive probabilistic programming framework in Python. PeerJ Comput Sci. 2023;9: e1516.

30. Wiechec E, Magan M, Matic N, Ansell-Schultz A, Kankainen M, Monni O, et al. Cancer-associated fibroblasts modulate transcriptional signatures involved in proliferation, differentiation and metastasis in head and neck squamous cell carcinoma. Cancers (Basel). 2021;13: 3361.

31. Johnson WE, Li C, Rabinovic A. Adjusting batch effects in microarray expression data using empirical Bayes methods. Biostatistics. 2007;8: 118–127.

32. pycombat: Python implementation of Combat for data harmonisation, allowing also to remove unwanted effects. Github;

33. Pham D, Tan X, Balderson B, Xu J, Grice LF, Yoon S, et al. Robust mapping of spatiotemporal trajectories and cell-cell interactions in healthy and diseased tissues. Nat Commun. 2023;14: 7739.
