# Supplementary material for: Spatial colocalization and molecular crosstalk of myofibroblastic CAFs and tumor cells shape lymph node metastasis in oral squamous cell carcinoma
Source: PLoS Genet. 2025 Sep 4;21(9):e1011791. doi: 10.1371/journal.pgen.1011791 (PMC12410789; doi:10.1371/journal.pgen.1011791)
Supplement: S1 Table — The percentages in each category are based on the total number of cases with sufficient information. The significance of continuous variables was evaluated using the two-sided Mann–Whitney U test, while the two-sided Fisher exact test was used for categorical variables. Abbreviations: IQR, interquartile range; LNM, lymph node metastasis; OSCC, oral squamous cell carcinoma. (PDF) [file pgen.1011791.s002.pdf]

S1 Table. Clinical characteristics of 201 patients with OSCC (related to Figs 2 and S1).

Characteristic		LNM (-) (*N* = 86)		LNM (+) (*N* = 115)		*P*-value*
**Median age at diagnosis, years (IQRs)**		59	(52–65)	60	(51–67)	0.86
**Sex, *n* (%)**	Women	33	(38)	27	(23)	0.03
	Men	53	(62)	88	(77)	
**Pathologic T stage, n (%)**	T1	10	(12)	5	(4)	0.05
	T2	32	(37)	34	(30)	
	T3	15	(17)	35	(30)	
	T4	29	(34)	41	(36)	
**Pathologic N stage, n (%)**	N0	86	(100)	0	(0)	
	N1	0	(0)	33	(29)	
	N2	0	(0)	80	(69)	
	N3	0	(0)	2	(2)	
**Tissue origin, n (%)**	Tongue	47	(55)	65	(57)	0.93

Floor of mouth	17	(20)	26	(23)
Cheek mucosa	7	(8)	10	(9)
Mouth	8	(9)	8	(7)
Gum	5	(6)	4	(3)
Palate	2	(2)	2	(2)

Table Legend

The percentages in each category are based on the total number of cases with sufficient information.

[*] The significance of continuous variables was evaluated using the two-sided Mann–Whitney *U* test, while the two-sided Fisher exact test was used for categorical variables.

Abbreviations: IQR, interquartile range; LNM, lymph node metastasis; OSCC, oral squamous cell carcinoma.
