# Supplementary material for: Spatial colocalization and molecular crosstalk of myofibroblastic CAFs and tumor cells shape lymph node metastasis in oral squamous cell carcinoma
Source: PLoS Genet. 2025 Sep 4;21(9):e1011791. doi: 10.1371/journal.pgen.1011791 (PMC12410789; doi:10.1371/journal.pgen.1011791)
Supplement: S2 Table — P-values lower than 0.05 were considered to indicate statistical significance. Abbreviations: CI, confidence interval; LNM, lymph node metastasis; OSCC, oral squamous cell carcinoma; OR, odds ratio; SE, standard error of the coefficient. (PDF) [file pgen.1011791.s003.pdf]

**S2 Table.** Multiple logistic regression for mutated genes and LNM in patients with OSCC (related to Fig 2B).

| | **Estimate** | **SE** | **z** | ***P*** | **OR** | **Lower 95% CI** | **Upper 95% CI** |
|---|---|---|---|---|---|---|---|
| **(Intercept)** | 0.59 | 0.39 | 1.52 | 0.13 | 1.80 | 0.84 | 3.87 |
| ***TP53*** | 0.05 | 0.38 | 0.14 | 0.89 | 1.05 | 0.50 | 2.21 |
| ***TTN*** | 0.28 | 0.33 | 0.86 | 0.39 | 1.33 | 0.69 | 2.54 |
| ***FAT1*** | −0.23 | 0.37 | -0.61 | 0.54 | 0.80 | 0.39 | 1.64 |
| ***CDKN2A*** | 0.31 | 0.38 | 0.81 | 0.42 | 1.36 | 0.65 | 2.86 |
| ***NOTCH1*** | −0.06 | 0.42 | −0.13 | 0.90 | 0.95 | 0.41 | 2.16 |
| ***MUC16*** | −0.80 | 0.43 | −1.87 | 0.06 | 0.45 | 0.19 | 1.04 |
| ***CASP8*** | −0.59 | 0.46 | −1.26 | 0.21 | 0.56 | 0.23 | 1.38 |
| ***SYNE1*** | −0.58 | 0.42 | −1.39 | 0.16 | 0.56 | 0.25 | 1.27 |
| ***PIK3CA*** | −0.79 | 0.41 | −1.94 | 0.05 | 0.46 | 0.21 | 1.01 |

**Table Legend**

*P*-values lower than 0.05 were considered to indicate statistical significance.

Abbreviations: CI, confidence interval; LNM, lymph node metastasis; OSCC, oral squamous cell carcinoma; OR, odds ratio; SE, standard error of the coefficient.
