# Supplementary material for: Spatial colocalization and molecular crosstalk of myofibroblastic CAFs and tumor cells shape lymph node metastasis in oral squamous cell carcinoma
Source: PLoS Genet. 2025 Sep 4;21(9):e1011791. doi: 10.1371/journal.pgen.1011791 (PMC12410789; doi:10.1371/journal.pgen.1011791)
Supplement: S3 Table — All patient data including Destination, age, sex, pathologic T stage, pathologic N stage, tissue origin, primary tumor site, and grade were obtained from a previous study [7]. Abbreviation: OSCC, oral squamous cell carcinoma. (PDF) [file pgen.1011791.s004.pdf]

S3 Table. Characteristics of 17 patients with OSCC derived from single-cell transcriptome data (related to Figs 3 and S2).

Sample ID	Destination	Age	Sex	Pathologic T stage	Pathologic N stage	Tissue origin	Primary tumor site	Grade
**OSCC5**	MEEI5	69	F	T2	N1	Tongue	Left lateral tongue	2
**OSCC6**	MEEI6	88	F	T4	N2	Floor of mouth	Right floor of mouth	1
**OSCC7**	MEEI7	71	F	T1	N2	Floor of mouth	Right floor of mouth	3
**OSCC8**	MEEI8	82	F	T4	N0	Palate	Right hard palate	1
Data not available	MEEI9	77	F	T1	N0	Tongue	Right lateral tongue	2
**OSCC10**	MEEI10	76	M	T4	N2	Gum	Right retromolar trigone	2
**OSCC12**	MEEI12	80	M	T4	N0	Gum	Left retromolar trigone	2
**OSCC13**	MEEI13	52	F	T3	N1	Tongue	Left lateral tongue	2
**OSCC16**	MEEI16	63	F	T2	N0	Tongue	Left lateral tongue	1
**OSCC17**	MEEI17	59	M	T4	N0	Gum	Right alveolar ridge	2
**OSCC18**	MEEI18	41	M	T3	N1	Tongue	Left lateral tongue	2
**OSCC20**	MEEI20	53	M	T4	N2	Floor of mouth	Right floor of mouth	2
**OSCC22**	MEEI22	77	M	T1	N0	Cheek mucosa	Left buccal mucosa	2
**OSCC23**	MEEI23	56	M	T3	N1	Gum	Right retromolar trigone	2
**OSCC24**	MEEI24	78	F	T4	N2	Gum	Right alveolar ridge	2
**OSCC25**	MEEI25	76	F	T3	N1	Tongue	Left lateral tongue	2
**OSCC26**	MEEI26	51	M	T4	N2	floor of mouth	Left floor of mouth	3
**OSCC28**	MEEI28	58	M	T2	N2	Tongue	Right lateral tongue	1

Table Legend

All patient data including Destination, age, sex, pathologic T stage, pathologic N stage, tissue origin, primary tumor site, and grade were obtained from a previous study [1].

Abbreviation: OSCC, oral squamous cell carcinoma.

References

1. Puram SV, Tirosh I, Parikh AS, Patel AP, Yizhak K, Gillespie S, et al. Single-Cell Transcriptomic Analysis of Primary and Metastatic Tumor Ecosystems in Head and Neck Cancer. Cell. 2017;171: 1611-1624.e24. doi:10.1016/j.cell.2017.10.044
