# Supplementary material for: Spatial colocalization and molecular crosstalk of myofibroblastic CAFs and tumor cells shape lymph node metastasis in oral squamous cell carcinoma
Source: PLoS Genet. 2025 Sep 4;21(9):e1011791. doi: 10.1371/journal.pgen.1011791 (PMC12410789; doi:10.1371/journal.pgen.1011791)
Supplement: S4 Table — This table includes all 20 patients in the clinical cohort. Patients HUH001 and HUH002 were used for spatial transcriptomics analysis only. Abbreviations: OSCC, oral squamous cell carcinoma; POI, pattern of invasion. (PDF) [file pgen.1011791.s005.pdf]

S4 Table. Characteristics of 20 patients with OSCC from a clinical cohort (related to Figs 5-9 and S3-S6).

Patient ID	Age	Sex	Pathologic T stage	Pathologic N stage	Tissue origin	Primary tumor site	Grade	POI
**HUH001**	48	F	T2	N3b	Tongue	Left lateral tongue	2	4
**HUH002**	61	F	T2	N0	Tongue	Right lateral tongue	2	3
**HUH003**	64	M	T2	N2	Tongue	Right lateral tongue	3	4
**HUH004**	80	F	T2	N1	Tongue	Left lateral tongue	2	4
**HUH005**	78	M	T2	N1	Tongue	Left lateral tongue	1	3
**HUH006**	45	M	T2	N1	Tongue	Right lateral tongue	2	4
**HUH007**	57	F	T2	N1	Tongue	Left lateral tongue	1	4
**HUH008**	68	F	T2	N0	Tongue	Left lateral tongue	2	4
**HUH009**	58	M	T2	N0	Tongue	Left lateral tongue	1	2
**HUH010**	59	M	T2	N0	Tongue	Base of tongue	2	2
**HUH011**	64	M	T2	N0	Tongue	Right lateral tongue	2	4
**HUH012**	34	M	T2	N0	Tongue	Left lateral tongue	2	4
**HUH013**	46	M	T2	N1	Tongue	Left lateral tongue	3	5
**HUH014**	79	M	T2	N1	Gum	Left alveolar ridge	3	3
**HUH015**	57	M	T2	N1	Cheek mucosa	Left buccal mucosa	2	4
**HUH016**	58	M	T2	N1	Tongue	Left lateral tongue	2	3
**HUH017**	63	F	T2	N0	Tongue	Left lateral tongue	1	3
**HUH018**	56	M	T2	N0	Tongue	Left lateral tongue	2	2
**HUH019**	71	M	T2	N0	Tongue	Left lateral tongue	1	3
**HUH020**	79	F	T2	N0	Tongue	Left lateral tongue	2	2

Table Legend

This table includes all 20 patients in the clinical cohort. Patients HUH001 and HUH002 were used for spatial transcriptomic analysis only.

Abbreviations: OSCC, oral squamous cell carcinoma; POI, pattern of invasion.
