# Supplementary material for: Spatial colocalization and molecular crosstalk of myofibroblastic CAFs and tumor cells shape lymph node metastasis in oral squamous cell carcinoma
Source: PLoS Genet. 2025 Sep 4;21(9):e1011791. doi: 10.1371/journal.pgen.1011791 (PMC12410789; doi:10.1371/journal.pgen.1011791)
Supplement: S5 Table — HUH001_met_1 and HUH001_met_2 were prepared from the same FFPE block. HUH001_met_1 was used for all subsequent analyses. Abbreviations: Conc., concentrated; DV200, percentage of RNA fragments longer than or equal to 200 nucleotides; FFPE, formalin-fixed, paraffin-embedded. (PDF) [file pgen.1011791.s006.pdf]

S5 Table. RNA quality evaluation with DV200 to predict FFPE assay performance (related to the Methods).

	From [nt]	To [nt]	Average Size [nt]	Conc. [pg/µL]	Region Molarity [pmol/L]	% of Total	DV200 [%]
**HUH001–P1**	200	1,296,279	35,912,0	6,570	53.8	95.33	95.33
**HUH001–P2**	200	1,296,279	35,933,0	2,770	22.7	93.77	93.77
**HUH002–P**	200	1,296,279	34,189,2	2,950	25.4	95.7	95.7
**HUH001–met_1**	200	1,296,279	19,156,7	8,010	123	93.44	93.44
**HUH001–met_2**	200	1,296,279	52,151,1	736	4.15	80.6	80.6

Table Legend

HUH001_met_1 and HUH001_met_2 were prepared from the same FFPE block. HUH001_met_1 was used for all subsequent analyses.

Abbreviations: Conc., concentrated; DV200, percentage of RNA fragments longer than or equal to 200 nucleotides; FFPE, formalin-fixed, paraffin-embedded.
