# Supplementary material for: Spatial colocalization and molecular crosstalk of myofibroblastic CAFs and tumor cells shape lymph node metastasis in oral squamous cell carcinoma
Source: PLoS Genet. 2025 Sep 4;21(9):e1011791. doi: 10.1371/journal.pgen.1011791 (PMC12410789; doi:10.1371/journal.pgen.1011791)
Supplement: S6 Table — Abbreviations: LNM, lymph node metastasis; UMI, Unique Molecular Identifiers. (PDF) [file pgen.1011791.s007.pdf]

S6 Table. Quality of the sequence and characteristics of the spatial transcriptome analysis (related to the Methods).

		**HUH001–P1** (LNM +)	**HUH001–P2** (LNM +)	**HUH001–met** Metastatic site	**HUH002–P** (LNM −)
**Spots for whole-transcriptome**	Number of spots under tissue	2,899	3,000	2,872	3,234
	Mean reads per spot	49,400	54,678	53,585	44,404
	Median genes per spot	1,046	1,052	4,054	2,654
**Sequencing**	Number of reads	143,210,704	164,034,393	153,894,971	143,601,425
	Valid barcodes	98.00%	97.80%	97.90%	97.70%
	Valid UMIs	100.00%	100.00%	100.00%	100.00%
	Sequencing saturation	75.50%	83.30%	71.60%	81.60%
	Q30 bases in barcode	93.40%	93.30%	93.20%	93.30%
	Q30 bases in probe read	88.50%	88.50%	88.80%	88.70%
	Q30 bases in UMI	90.90%	90.90%	90.90%	90.80%
**Mapping**	Reads mapped to probe set	95.70%	95.50%	95.50%	94.80%
	Reads mapped confidently to probe set	95.00%	94.50%	94.70%	90.60%
	Reads mapped confidently to the filtered probe set	76.00%	75.10%	72.90%	68.00%
**Spots**	Fraction reads in spots under tissue	98.40%	98.00%	96.80%	98.90%
	Mean reads per spot	49,400	54,678	53,585	44,404
	Mean reads under tissue per spot	47,637	52,368	50,765	42,836
	Median UMI counts per spot	1,720	1,765	11,414	6,372
	Median genes per spot	1,046	1,052	4,054	2,654
	Genes detected	17,84	17,825	17,834	17,861

Table Legend
Abbreviations: LNM, lymph node metastasis; UMI, Unique Molecular Identifiers.
