# Supplementary material for: Spatial colocalization and molecular crosstalk of myofibroblastic CAFs and tumor cells shape lymph node metastasis in oral squamous cell carcinoma
Source: PLoS Genet. 2025 Sep 4;21(9):e1011791. doi: 10.1371/journal.pgen.1011791 (PMC12410789; doi:10.1371/journal.pgen.1011791)
Supplement: S7 Table — All data including Sample ID, patient ID, age, pathologic T stage, pathologic N stage, tissue origin, primary tumor site, and grade were obtained from a previous study [25]. Abbreviation: OSCC, oral squamous cell carcinoma. (PDF) [file pgen.1011791.s008.pdf]

S7 Table. Characteristics of 12 samples with OSCC derived from validation spatial transcriptome data (related to Figs 9G, S4L-S4P, and S5K).

Sample ID	Patient ID	Age, years	Pathologic T stage	Pathologic N stage	Tissue origin	Primary tumor site	Grade
**S1**	P1	50-59	T2	N2	Tongue	Anterior	2
**S2**	P2	50-59	T4	N1	Floor of mouth	Posterior floor of mouth	2
**S3**	P3	50-59	T1	N0	Tongue	Left lateral tongue	1
**S4**	P4	30-39	T2	N2	Tongue	Left lateral tongue	1
**S5**	P5	60-69	T2	N0	Tongue	Right lateral tongue	2
**S6**	P6	40-49	T2	N0	Tongue	Left lateral tongue	2
**S7**	P7	70-79	T4	N0	Tongue	Left retromolar tongue	3
**S8**	P7	70-79	T4	N0	Tongue	Left lateral tongue	3
**S9**	P8	50-59	T4	N1	Tongue	Left lateral tongue	2
**S10**	P8	50-59	T4	N1	Floor of mouth	Anterior floor of mouth	2
**S11**	P9	60-69	T2	N0	Floor of mouth	Floor of mouth	2
**S12**	P10	60-69	T2	N2	Floor of mouth	Floor of mouth	2

Table Legend

All data including Sample ID, patient ID, age, pathologic T stage, pathologic N stage, tissue origin, primary tumor site, and grade were obtained from a previous study [1].

Abbreviation: OSCC, oral squamous cell carcinoma.

References

1. Arora R, Cao C, Kumar M, Sinha S, Chanda A, McNeil R, et al. Spatial transcriptomics reveals distinct and conserved tumor core and edge architectures that predict survival and targeted therapy response. Nat Commun. 2023;14: 5029. doi:10.1038/s41467-023-40271-4
