# Supplementary material for: Spatial colocalization and molecular crosstalk of myofibroblastic CAFs and tumor cells shape lymph node metastasis in oral squamous cell carcinoma
Source: PLoS Genet. 2025 Sep 4;21(9):e1011791. doi: 10.1371/journal.pgen.1011791 (PMC12410789; doi:10.1371/journal.pgen.1011791)
Supplement: S8 Table — All data including Geo accession, sample ID, cell line, cell type, and treatment condition were obtained from the Gene Expression Omnibus (GEO) dataset GSE279481 [26]. Abbreviations: CAF, cancer-associated fibroblast; HPV, human papilloma virus; OSCC, oral squamous cell carcinoma; SCC, squamous cell carcinoma. (PDF) [file pgen.1011791.s009.pdf]

S8 Table. Characteristics of HPV-negative OSCC and CAF co-culture samples (related to S4Q-S4S Fig).

Geo accession	Sample	Cell line	Cell type	Treatment
GSE279481	GSM8573232	Cal33	Tongue SCC	Control
GSE279481	GSM8573233	61137	CAFs	Control
GSE279481	GSM8573234	Cal33	Tongue SCC	Co-cultured with 61137
GSE279481	GSM8573235	61137	CAFs	Co-cultured with Cal33
GSE279481	GSM8573236	Cal33	Tongue SCC	Control
GSE279481	GSM8573237	61162	CAFs	Control
GSE279481	GSM8573238	Cal33	Tongue SCC	Co-cultured with 61162
GSE279481	GSM8573239	61162	CAFs	Co-cultured with Cal33
GSE279481	GSM8573240	Cal27	Tongue SCC	Control
GSE279481	GSM8573241	61137	CAFs	Control
GSE279481	GSM8573242	61162	CAFs	Control
GSE279481	GSM8573243	Cal27	Tongue SCC	Co-cultured with 61137
GSE279481	GSM8573244	61137	CAFs	Co-cultured with Cal27
GSE279481	GSM8573245	Cal27	Tongue SCC	Co-cultured with 61137
GSE279481	GSM8573246	61162	CAFs	Co-cultured with Cal27

Table Legend

All data including Geo accession, sample ID, cell line, cell type, and treatment condition were obtained from the Gene Expression Omnibus (GEO) dataset GSE279481 [1].

Abbreviations: CAF, cancer-associated fibroblast; HPV, human papilloma virus; OSCC, oral squamous cell carcinoma; SCC, squamous cell carcinoma.

References

1. Waas M, Karamboulas C, Wu BZ, Khan S, Poon S, Meens J, et al. Molecular correlates for HPV-negative head and neck cancer engraftment prognosticate patient outcomes. Nat Commun. 2024;15: 10869. doi:10.1038/s41467-024-55203-z
