# Supplementary material for: Spatial colocalization and molecular crosstalk of myofibroblastic CAFs and tumor cells shape lymph node metastasis in oral squamous cell carcinoma
Source: PLoS Genet. 2025 Sep 4;21(9):e1011791. doi: 10.1371/journal.pgen.1011791 (PMC12410789; doi:10.1371/journal.pgen.1011791)
Supplement: S9 Table — All data including Geo accession, sample ID, age, sex, cell line, experiment type, tumor location, treatment condition, and stage were obtained from the Gene Expression Omnibus (GEO) datasets GSE178153/GSE178154 [27]. Abbreviations: CAF, cancer-associated fibroblast; HNSCC, head and neck squamous cell carcinoma. (PDF) [file pgen.1011791.s010.pdf]

S9 Table. Characteristics of patient-derived HNSCC and CAF co-culture samples (related to S4T Fig).

Geo accession	Sample	Age, years	Sex	Cell line	Experiment	Location	Treatment	Stage
GSE178153	GSM5381030	74	M	LK0824	2D	Tongue	Control	T2N1M0
GSE178153	GSM5381031	38	F	LK0858	2D	Tongue	Control	T3N0M0
GSE178153	GSM5381032	79	F	LK0923	2D	Larynx	Control	T1N0M0
GSE178153	GSM5381033	71	M	LK0942	2D	Larynx	Control	T4N0M0
GSE178153	GSM5381034	62	M	LK0949	2D	Tongue	Control	T2N0M0
GSE178153	GSM5381035	74	M	LK0824	2D	Tongue	Co-cultured with CAF	T2N1M0
GSE178153	GSM5381036	38	F	LK0858	2D	Tongue	Co-cultured with CAF	T3N0M0
GSE178153	GSM5381037	79	F	LK0923	2D	Larynx	Co-cultured with CAF	T1N0M0
GSE178153	GSM5381038	71	M	LK0942	2D	Larynx	Co-cultured with CAF	T4N0M0
GSE178153	GSM5381039	62	M	LK0949	2D	Tongue	Co-cultured with CAF	T2N0M0
GSE178154	GSM5381040	68	F	LK0902	3D	Tongue	Control	T1N0M0
GSE178154	GSM5381041	79	M	LK0917	3D	Gingiva	Control	T4N1M1
GSE178154	GSM5381042	65	F	LK1108	3D	Hypopharynx	Control	T2N0M0
GSE178154	GSM5381043	68	F	LK0902	3D	Tongue	Co-cultured with CAF	T1N0M0
GSE178154	GSM5381044	79	M	LK0917	3D	Gingiva	Co-cultured with CAF	T4N1M1
GSE178154	GSM5381045	65	F	LK1108	3D	Hypopharynx	Co-cultured with CAF	T2N0M0

Table Legend

All data including Geo accession, sample ID, age, sex, cell line, experiment type, tumor location, treatment condition, and stage were obtained from the Gene Expression Omnibus (GEO) datasets GSE178153/GSE178154 [1].

Abbreviations: CAF, cancer-associated fibroblast; HNSCC, head and neck squamous cell carcinoma.

References

1. Wiechec E, Magan M, Matic N, Ansell-Schultz A, Kankainen M, Monni O, et al. Cancer-associated fibroblasts modulate transcriptional signatures involved in proliferation, differentiation and metastasis in head and neck squamous cell carcinoma. Cancers (Basel). 2021;13: 3361. doi:10.3390/cancers13133361
