# Supplementary material for: Spatial colocalization and molecular crosstalk of myofibroblastic CAFs and tumor cells shape lymph node metastasis in oral squamous cell carcinoma
Source: PLoS Genet. 2025 Sep 4;21(9):e1011791. doi: 10.1371/journal.pgen.1011791 (PMC12410789; doi:10.1371/journal.pgen.1011791)
Supplement: S10 Table — Cluster numbers are from the analysis of our Visium spatial transcriptome dataset. (PDF) [file pgen.1011791.s011.pdf]

S10 Table. *CD44* expression and pseudotime in integrated spatial transcriptome clusters (related to S5H Fig).

Cluster	*CD44* expression					Pseudotime		
	Median	25th percentile	75th percentile	***P***	Adjusted ***P***	Median	25th percentile	75th percentile
1	1.59	1.34	1.78	0.008	0.012	0.28	0.26	0.30
2	1.67	1.32	1.95	<0.001	<0.001	0.37	0.35	0.38
3	1.65	1.51	1.79	<0.001	<0.001	0.16	0.13	0.20
4	1.44	1.13	1.67	0.307	0.338	0.34	0.31	0.37
5	1.99	1.76	2.20	<0.001	<0.001	0.26	0.21	0.31
6	1.50	1.12	1.90	0.571	0.571	0.48	0.45	0.52
7	1.67	1.42	1.92	<0.001	<0.001	0.32	0.30	0.33
8	1.61	1.24	1.87	0.052	0.063	0.40	0.35	0.52
9	1.86	1.62	2.09	<0.001	<0.001	0.20	0.18	0.24
10	1.87	1.44	2.20	<0.001	<0.001	0.39	0.35	0.41
11	1.34	0.00	1.72	0.013	0.018	0.43	0.37	0.57
12	1.46	1.17	1.71			0.33	0.32	0.36

Table Legend

Cluster numbers are from the analysis of our Visium spatial transcriptome dataset.
