# Supplementary material for: Spatial colocalization and molecular crosstalk of myofibroblastic CAFs and tumor cells shape lymph node metastasis in oral squamous cell carcinoma
Source: PLoS Genet. 2025 Sep 4;21(9):e1011791. doi: 10.1371/journal.pgen.1011791 (PMC12410789; doi:10.1371/journal.pgen.1011791)
Supplement: S11 Table — Cluster numbers, top differentially expressed genes (DEGs), spatial transcriptome-based cluster names, cluster classifications (metastatic or primary tumor), and relevant references are from the analysis of our Visium spatial transcriptome dataset. Abbreviations: GC, Germinal Center; myCAF, myofibroblastic cancer-associated fibroblast; OSCC, oral squamous cell carcinoma. (PDF) [file pgen.1011791.s012.pdf]

S11 Table. Molecular characteristics and top DEGs of integrated spatial transcriptome clusters (related to S5H Fig).

Cluster	Top DEGs each integrated spatial transcriptome cluster					Spatial transcriptome-based cluster name	Cluster classification	Reference
1	DES	TNNI1	CKM	SOX11	COL11A1	Contractile myCAFs with OSCC	Metastatic	[1,2]
2	IGHD	NCCRP1	SPIB	CD22	XIRP2	Naïve B cell with OSCC	Metastatic	[3,4]
3	FLG	MFAP5	KRTAP2-3	CPA4	CNTNAP2	Keratin-high OSCC	Primary tumor	[5]
4	PAEP	CCL21	NLRP14	HMGA2	ACTN2	CCL21-driven migratory OSCC	Metastatic	[5–9]
5	PI3	SERPINB3	S100A9	TMPRSS11D	MSLN	SERPINB3-high invasive OSCC	Metastatic	[10–13]
6	VPREB3	DUXA	EIF5AL1	XCL1	LIPJ	Pre-/GC B-cell with OSCC	Metastatic	[14]
7	IGLV9-49	IGHG4	IGLV3-1	IGHA1	IGHV5-10-1	Mature plasma cell with OSCC	Metastatic	[15]
8	IGLV6-57	KRT13	IGKC	IGHG2	MPPED2	Mature plasma cell with OSCC	Metastatic	[15]
9	SPINK6	CXCL14	IGHV6-1	PTPRZ1	KRT1	$CXCL14^{+}$ basal-like OSCC	Metastatic	[16,17]
10	STRIP2	FGFBP1	BLNK	CLCA2	LRRC15	$LRRC15^{+}$ CAFs with OSCC	Metastatic	[18,19]
11	SAMD11	KRT4	CYP7A1	CCR5	OR4N2	CCR5-high mucosal-type OSCC	Metastatic	[20]
12	TCAP	ACTC1	KLHL40	TRIM72	MYLPF	Myogenic OSCC	Primary tumor	[21]

Table Legend

Cluster numbers, top differentially expressed genes (DEGs), spatial transcriptome-based cluster names, cluster classifications (metastatic or primary tumor), and relevant references are from the analysis of our Visium spatial transcriptome dataset.

Abbreviations: GC, Germinal Center; myCAF, myofibroblastic cancer-associated fibroblast; OSCC, oral squamous cell carcinoma.

References

1. Feng Q-S, Shan X-F, Yau V, Cai Z-G, Xie S. Facilitation of tumor stroma-targeted therapy: Model difficulty and co-culture organoid method. Pharmaceuticals (Basel). 2025;18. doi:10.3390/ph18010062

2. Arebro J, Lee C-M, Bennewith KL, Garnis C. Cancer-associated fibroblast heterogeneity in malignancy with focus on oral squamous cell carcinoma. Int J Mol Sci. 2024;25: 1300. doi:10.3390/ijms25021300

3. Su B, Sun R, Zhang L, Zhang J, Feng X, Cao Z, et al. Single-cell RNA sequencing-based immunological feature analysis of a COVID-19 patient with recurrent positive SARS-CoV-2 RNA. Chin Med J (Engl). 2022;135: 1000–1002. doi:10.1097/CM9.0000000000001956

4. Verstegen NJM, Pollastro S, Unger P-PA, Marsman C, Elias G, Jorritsma T, et al. Single-cell analysis reveals dynamics of human B cell differentiation and identifies novel B and antibody-secreting cell intermediates. Elife. 2023;12. doi:10.7554/eLife.83578

5. Takahashi K, Podyma-Inoue KA, Saito M, Sakakitani S, Sugauchi A, Iida K, et al. TGF-β generates a population of cancer cells residing in G1 phase with high motility and metastatic potential via KRTAP2-3. Cell Rep. 2022;40: 111411. doi:10.1016/j.celrep.2022.111411

6. Qin Y, He LD, Sheng ZJ, Yong MM, Sheng YS, Wei Dong X, et al. Increased CCL19 and CCL21 levels promote fibroblast ossification in ankylosing spondylitis hip ligament tissue. BMC Musculoskelet Disord. 2014;15: 316. doi:10.1186/1471-2474-15-316

7. Chen Z, Zhou L, Liu L, Hou Y, Xiong M, Yang Y, et al. Single-cell RNA sequencing highlights the role of inflammatory cancer-associated fibroblasts in bladder urothelial carcinoma. Nat Commun. 2020;11: 5077. doi:10.1038/s41467-020-18916-5

8. Kieffer Y, Hocine HR, Gentric G, Pelon F, Bernard C, Bourachot B, et al. Single-cell analysis reveals fibroblast clusters linked to immunotherapy resistance in cancer. Cancer Discov. 2020;10: 1330–1351. doi:10.1158/2159-8290.CD-19-1384

9. Cords L, Tietscher S, Anzeneder T, Langwieder C, Rees M, de Souza N, et al. Cancer-associated fibroblast classification in single-cell and spatial proteomics data. Nat Commun. 2023;14: 4294. doi:10.1038/s41467-023-39762-1

10. Chen L, Shi V, Wang S, Sun L, Freeman R, Yang J, et al. SCCA1/SERPINB3 suppresses antitumor immunity and blunts therapy-induced T cell responses via STAT-dependent chemokine production. J Clin Invest. 2023;133. doi:10.1172/JCI163841

11. Hu Y, Han Y, He M, Zhang Y, Zou X. S100 proteins in head and neck squamous cell carcinoma (Review). Oncol Lett. 2023;26: 362. doi:10.3892/ol.2023.13948

12. Raffat MA, Hadi NI, Hosein M, Mirza S, Ikram S, Akram Z. S100 proteins in oral squamous cell carcinoma. Clin Chim Acta. 2018;480: 143–149. doi:10.1016/j.cca.2018.02.013

13. Qu ZF, Ma H, Duan XF, Wu R, Zou Y. The expression and significance of S100A9 in oral squamous cell carcinoma. Lin Chuang Er Bi Yan Hou Tou Jing Wai Ke Za Zhi. 2017;31: 219–222. doi:10.13201/j.issn.1001-1781.2017.03.013

14. Rodig SJ, Kutok JL, Paterson JC, Nitta H, Zhang W, Chapuy B, et al. The pre-B-cell receptor associated protein VpreB3 is a useful diagnostic marker for identifying c-MYC translocated lymphomas. Haematologica. 2010;95: 2056–2062. doi:10.3324/haematol.2010.025767

15. Kurkalang S, Roy S, Acharya A, Mazumder P, Mazumder S, Patra S, et al. Single-cell transcriptomic analysis of gingivo-buccal oral cancer reveals two dominant cellular programs. Cancer Sci. 2023;114: 4732–4746. doi:10.1111/cas.15979

16. Meyer-Hoffert U, Wu Z, Kantyka T, Fischer J, Latendorf T, Hansmann B, et al. Isolation of SPINK6 in human skin. J Biol Chem. 2010;285: 32174–32181. doi:10.1074/jbc.m109.091850

17. Nakayama R, Arikawa K, Bhawal UK. The epigenetic regulation of CXCL14 plays a role in the pathobiology of oral cancers. J Cancer. 2017;8: 3014–3027. doi:10.7150/jca.21169

18. Krishnamurty AT, Shyer JA, Thai M, Gandham V, Buechler MB, Yang YA, et al. LRRC15+ myofibroblasts dictate the stromal setpoint to suppress tumour immunity. Nature. 2022;611: 148–154. doi:10.1038/s41586-022-05272-1

19. Dominguez CX, Müller S, Keerthivasan S, Koeppen H, Hung J, Gierke S, et al. Single-cell RNA sequencing reveals stromal evolution into LRRC15+ myofibroblasts as a determinant of patient response to cancer immunotherapy. Cancer Discov. 2020;10: 232–253. doi:10.1158/2159-8290.CD-19-0644

20. Domingueti C-B, Janini J-B-M, Paranaíba L-M-R, Lozano-Burgos C, Olivero P, González-Arriagada W-A. Prognostic value of immunoexpression of CCR4, CCR5, CCR7 and CXCR4 in squamous cell carcinoma of tongue and floor of the mouth. Med Oral Patol Oral Cir Bucal. 2019;24: e354–e363. doi:10.4317/medoral.22904

21. Kim SC, Kellett T, Wang S, Nishi M, Nagre N, Zhou B, et al. TRIM72 is required for effective repair of alveolar epithelial cell wounding. Am J Physiol Lung Cell Mol Physiol. 2014;307: L449-59. doi:10.1152/ajplung.00172.2014
