# Supplementary material for: Spatial colocalization and molecular crosstalk of myofibroblastic CAFs and tumor cells shape lymph node metastasis in oral squamous cell carcinoma
Source: PLoS Genet. 2025 Sep 4;21(9):e1011791. doi: 10.1371/journal.pgen.1011791 (PMC12410789; doi:10.1371/journal.pgen.1011791)
Supplement: S12 Table — Genes labeled “unfavorable” with a positive LASSO weight act as poor-prognosis biomarkers, whereas “favorable” genes with a negative weight act as good-prognosis biomarkers. Abbreviation: myCAF, myofibroblastic cancer-associated fibroblast. (PDF) [file pgen.1011791.s013.pdf]

S12 Table. Spatially-resolved 23 core signature genes are ranked by Spearman correlation with myCAF proportion (related to Figs 9 and S6).

Gene symbol	Coefficient	Gene weight	Spearman with myCAF proportion	Adjusted *P*	Description
**SFRP2**	Unfavorable	0.318	0.648	7.31E-25	Secreted frizzled related protein 2
**NES**	Favorable	−0.162	0.634	1.35E-23	Nestin
**MRC2**	Unfavorable	0.364	0.592	4.80E-20	Mannose receptor C type 2
**COL15A1**	Favorable	−0.407	0.529	1.47E-15	Collagen type XV alpha 1 chain
**CCN2**	Unfavorable	0.315	0.498	1.21E-13	Cellular communication network factor 2
**MFGE8**	Favorable	−0.408	0.412	3.53E-09	Milk fat globule EGF and factor V/VIII domain-containing
**PTX3**	Unfavorable	0.131	0.399	1.34E-08	Pentraxin 3
**S100A4**	Favorable	−0.511	0.368	2.42E-07	S100 calcium binding protein A4
**C3**	Unfavorable	0.191	0.333	4.50E-06	Complement C3
**UBXN6**	Favorable	−0.18	0.293	7.57E-05	UBX domain protein 6
**DES**	Unfavorable	0.374	0.289	9.81E-05	Desmin
**CORO1A**	Favorable	−0.243	0.238	0.002	Coronin 1A
**CFD**	Unfavorable	0.242	0.214	0.007	Complement factor D
**NOM1**	Unfavorable	0.213	0.192	0.018	Nucleolar protein with MIF4G domain 1
**NTMT1**	Unfavorable	0.214	0.192	0.018	N-terminal Xaa-Pro-Lys N-methyltransferase 1
**MRFAP1L1**	Favorable	−0.338	0.15	0.083	Morf4 family associated protein 1 like 1
**C4orf3**	Unfavorable	0.122	0.146	0.093	Chromosome 4 open reading frame 3
**ABHD16A**	Unfavorable	0.235	0.101	0.297	Abhydrolase domain containing 16A, phospholipase
**LUC7L3**	Favorable	−0.28	0.074	0.475	LUC7-like 3 pre-mrna splicing factor
**PIM3**	Unfavorable	0.289	0.063	0.561	Pim-3 proto-oncogene, serine/threonine kinase
**OLR1**	Unfavorable	0.227	0.055	0.62	Oxidized low density lipoprotein receptor 1
**IFNGR2**	Unfavorable	0.245	0.038	0.749	Interferon gamma receptor 2
**TXNDC17**	Unfavorable	0.166	−0.139	0.117	Thioredoxin domain containing 17

Table Legend

Genes labeled “unfavorable” with a positive LASSO weight act as poor-prognosis biomarkers, whereas “favorable” genes with a negative weight act as good-prognosis biomarkers.

Abbreviation: myCAF, myofibroblastic cancer-associated fibroblast.
