# Supplementary material for: Spatial colocalization and molecular crosstalk of myofibroblastic CAFs and tumor cells shape lymph node metastasis in oral squamous cell carcinoma
Source: PLoS Genet. 2025 Sep 4;21(9):e1011791. doi: 10.1371/journal.pgen.1011791 (PMC12410789; doi:10.1371/journal.pgen.1011791)
Supplement: S13 Table — Abbreviations: BRCA, breast invasive carcinoma; CRC, colorectal cancer; cSCC, cutaneous squamous cell carcinoma; GBM, glioblastoma multiforme; HCC, hepatocellular carcinoma; HNSCC, head and neck squamous cell carcinoma; ICI, immune checkpoint inhibitor; OSCC, oral squamous cell carcinoma; PDX, patient-derived xenograft; PMN-MDSC, polymorphonuclear myeloid derived suppressor cells. (PDF) [file pgen.1011791.s014.pdf]

S13 Table. Biological and clinical relevance of 23 spatially-resolved signature genes (related to Figs 9 and S6).

Gene symbol	Key biological role	Current or emerging application	Reference
**SFRP2**	Wnt antagonist;<br>tumor angiogenesis	Stool DNA test (CRC);<br>anti-SFRP2 mAb	[1–5]
**NES**	Stem-cell filament;<br>neo-angiogenesis	IHC prognostic marker in OSCC, BRCA, GBM	[6–10]
**MRC2 (Endo180)**	Collagen uptake; invasion	Endo180-ADC in sarcoma PDX; myCAF targeting boosts immunotherapy.	[11–15]
**COL15A1**	Non-fibrillar collagen;<br>vascular niche	Immune–stromal prognostic marker;<br>MSI/TMB correlate	[16–18]
**CCN2 (CTGF)**	ECM remodelling; fibrosis	Pamrevlumab (FG-3019, NCT03941093, PDAC)	[19–22]
**MFGE8**	PS bridging; efferocytosis	IHC marker of local recurrence in OSCC	[23,24]
**PTX3**	Long pentraxin; complement tuner	Plasma prognostic biomarker;<br>immune-modulation target	[25–27]
**S100A4**	Metastasin;<br>EMT & MMPs	IHC predictor of nodal metastasis;<br>S100A4-RAGE inhibitors (pre-cl.)	[28–31]
**C3 (CPAMD1)**	Central complement node	Serum C3a/C5a correlate with OSCC stage;<br>C3-inhibitors (AMY-101)	[32–34]
**UBXN6 (UBXD1)**	p97 adaptor;<br>IFN-STAT stabilizer	Low UBXN6 predicts relapse (gastric CA)	[35–37]
**DES (CSM1)**	Muscle filament;<br>myofibre marker	Pathology aid for muscle invasion depth	[38,39]
**CORO1A (p57)**	Actin regulator;<br>immune cell motility	High in LOX-1$^+$ PMN-MDSC;<br>target to curb metastasis	[32,40,41]
**CFD (ADIPSIN)**	Trigger of complement alternative pathway	Danicopan inhibits CFD;<br>high CFD in cSCC	[33,42,43]
**NOM1 (SGD1)**	Nucleolar ribosome biogenesis	New targets; Part of high-proliferation gene hubs in OSCC	[44–47]
**NTMT1**	N-terminal methyltransferase;<br>genome stability	High NTMT1 → poor OS;<br>radiosensitisation when inhibited	[48–55]
**MRFAP1L1 (PP784)**	Chromatin remodel associate	Candidate prognostic marker in RCC	[56]
**C4orf3 (ARLN)**	ER membrane protein;<br>Ca^{2+} homeostasis	Exploratory;<br>potential ER-stress modulator	[57,58]
**ABHD16A**	Lyso-PS synthase;<br>immune suppression	ABHD16A inhibitors enhance ICI in mice	[32,59,60]
**LUC7L3**	Pre-mRNA splicing factor	Potential HCC prognostic biomarker	[45,61]
**PIM3**	Ser/Thr kinase;<br>survival & mTOR	PIM inhibitors (AZD1208, SGI-1776) in early trials	[37,62–68]

**OLR1 (LOX-1)**	Scavenger receptor; MDSC marker	Anti-LOX-1 depletes PMN-MDSC; prognostic in HNSCC	[32,69–71]
**IFNGR2**	IFN-γ receptor β-chain; Ligand activates JAK/STAT	Loss/mutation → ICI resistance biomarker	[72–75]
**TXNDC17 (TRP14)**	Redox enzyme;	High TXNDC17 → taxane resistance (ovarian CA)	[76–78]

Table Legend

Abbreviations: BRCA, breast invasive carcinoma; CRC, colorectal cancer; cSCC, cutaneous squamous cell carcinoma; GBM, glioblastoma multiforme; HCC, hepatocellular carcinoma; HNSCC, head and neck squamous cell carcinoma; ICI, immune checkpoint inhibitor; OSCC, oral squamous cell carcinoma; PDX, patient-derived xenograft; PMN-MDSC, polymorphonuclear myeloid derived suppressor cells.

References

1. Xiao C, Wang L, Zhu L, Zhang C, Zhou J. Secreted frizzled-related protein 2 is epigenetically silenced and functions as a tumor suppressor in oral squamous cell carcinoma. Mol Med Rep. 2014;10: 2293–2298. doi:10.3892/mmr.2014.2542

2. Yang Q, Huang T, Ye G, Wang B, Zhang X. Methylation of SFRP2 gene as a promising noninvasive biomarker using feces in colorectal cancer diagnosis: a systematic meta-analysis. Sci Rep. 2016;6: 33339. doi:10.1038/srep33339

3. Garcia D, Nasarre P, Bonilla IV, Hilliard E, Peterson YK, Spruill L, et al. Development of a novel humanized monoclonal antibody to secreted frizzled-related protein-2 that inhibits triple-negative breast cancer and angiosarcoma growth in vivo. Ann Surg Oncol. 2019;26: 4782–4790. doi:10.1245/s10434-019-07800-2

4. Fane ME, Ecker BL, Kaur A, Marino GE, Alicea GM, Douglass SM, et al. SFRP2 supersedes VEGF as an age-related driver of angiogenesis in melanoma, affecting response to anti-VEGF therapy in older patients. Clin Cancer Res. 2020;26: 5709–5719. doi:10.1158/1078-0432.CCR-20-0446

5. van Loon K, Huijbers EJM, Griffioen AW. Secreted frizzled-related protein 2: a key player in noncanonical Wnt signaling and tumor angiogenesis. Cancer Metastasis Rev. 2021;40: 191–203. doi:10.1007/s10555-020-09941-3

6. Matsuda Y, Hagio M, Ishiwata T. Nestin: a novel angiogenesis marker and possible target for tumor angiogenesis. World J Gastroenterol. 2013;19: 42–48. doi:10.3748/wjg.v19.i1.42

7. Ravindran G, Devaraj H. Prognostic significance of neural stem cell markers, Nestin and Musashi-1, in oral squamous cell carcinoma: expression pattern of Nestin in the precancerous stages of oral squamous epithelium. Clin Oral Investig. 2015;19: 1251–1260. doi:10.1007/s00784-014-1341-z

8. Asleh K, Won JR, Gao D, Voduc KD, Nielsen TO. Nestin expression in breast cancer: association with prognosis and subtype on 3641 cases with long-term follow-up. Breast Cancer Res Treat. 2018;168: 107–115. doi:10.1007/s10549-017-4583-z

9. Krüger K, Wik E, Knutsvik G, Nalwoga H, Klingen TA, Arnes JB, et al. Expression of Nestin associates with BRCA1 mutations, a basal-like phenotype and aggressive breast cancer. Sci Rep. 2017;7. doi:10.1038/s41598-017-00862-w

10. Prosniak M, Kenyon LC, Hooper DC. Glioblastoma contains topologically distinct proliferative and metabolically defined subpopulations of Nestin- and Glut1-expressing cells. J Neuropathol Exp Neurol. 2021;80: 674–684. doi:10.1093/jnen/nlab044

11. Melander MC, Jürgensen HJ, Madsen DH, Engelholm LH, Behrendt N. The collagen receptor uPARAP/Endo180 in tissue degradation and cancer (Review). Int J Oncol. 2015;47: 1177–1188. doi:10.3892/ijo.2015.3120

12. Chen J, Yang J, Li H, Yang Z, Zhang X, Li X, et al. Single-cell transcriptomics reveal the intratumoral landscape of infiltrated T-cell subpopulations in oral squamous cell carcinoma. Mol Oncol. 2021;15: 866–886. doi:10.1002/1878-0261.12910

13. Evans RJ, Perkins DW, Selfe J, Kelsey A, Birch GP, Shipley JM, et al. Endo180 (MRC2) antibody-drug conjugate for the treatment of sarcoma. Mol Cancer Ther. 2023;22: 240–253. doi:10.1158/1535-7163.MCT-22-0312

14. Gopalakrishnan KV, Kannan B, Pandi C, Jayaseelan VP, Arumugam P. Prognostic and clinicopathological significance of MRC2 expression in head and neck squamous cell carcinoma. J Stomatol Oral Maxillofac Surg. 2023;124: 101617. doi:10.1016/j.jormas.2023.101617

15. Liu Y, Liang J, Zhang Y, Guo Q. Drug resistance and tumor immune microenvironment: An overview of current understandings (Review). Int J Oncol. 2024;65. doi:10.3892/ijo.2024.5684

16. Zhu L, Jiang Q, Meng J, Zhao H, Lin J. Pan-cancer analysis of COL15A1: an immunological and prognostic biomarker. Discov Oncol. 2024;15: 325. doi:10.1007/s12672-024-01200-z

17. Gao Y, Li J, Cheng W, Diao T, Liu H, Bo Y, et al. Cross-tissue human fibroblast atlas reveals myofibroblast subtypes with distinct roles in immune modulation. Cancer Cell. 2024;42: 1764-1783.e10. doi:10.1016/j.ccell.2024.08.020

18. Liu Y-T, Liu H-M, Ren J-G, Zhang W, Wang X-X, Yu Z-L, et al. Immune-featured stromal niches associate with response to neoadjuvant immunotherapy in oral squamous cell carcinoma. Cell Rep Med. 2025;6: 102024. doi:10.1016/j.xcrm.2025.102024

19. Jia Q, Xu B, Zhang Y, Ali A, Liao X. CCN family proteins in cancer: Insight into their structures and coordination role in tumor microenvironment. Front Genet. 2021;12: 649387. doi:10.3389/fgene.2021.649387

20. Moritani NH, Kubota S, Nishida T, Kawaki H, Kondo S, Sugahara T, et al. Suppressive effect of overexpressed connective tissue growth factor on tumor cell growth in a human oral squamous cell carcinoma-derived cell line. Cancer Lett. 2003;192: 205–214. doi:10.1016/s0304-3835(02)00718-8

21. Yang W-Y, Tang C-H, Chuang J-Y. Abstract 1392: CTGF inhibits cell motility in oral cancer cells through reducing COX-2 expression. Cancer Res. 2011;71: 1392–1392. doi:10.1158/1538-7445.am2011-1392

22. Wu Y-L, Li H-Y, Zhao X-P, Jiao J-Y, Tang D-X, Yan L-J, et al. Mesenchymal stem cell-derived CCN2 promotes the proliferation, migration and invasion of human tongue squamous cell carcinoma cells. Cancer Sci. 2017;108: 897–909. doi:10.1111/cas.13202

23. Yamazaki M, Maruyama S, Abé T, Essa A, Babkair H, Cheng J, et al. MFG-E8 expression for progression of oral squamous cell carcinoma and for self-clearance of apoptotic cells. Lab Invest. 2014;94: 1260–1272. doi:10.1038/labinvest.2014.108

24. Okamoto A, Sakakura K, Takahashi H, Motegi S-I, Kaira K, Yokobori-Kuwabara Y, et al. Immunological and clinicopathological significance of MFG-E8 expression in patients with oral squamous cell carcinoma. Pathol Oncol Res. 2020;26: 1263–1268. doi:10.1007/s12253-019-00692-3

25. Jung H, Kang J, Han K-M, Kim H. Prognostic value of Pentraxin3 protein expression in human malignancies: A systematic review and meta-analysis. Cancers (Basel). 2024;16. doi:10.3390/cancers16223754

26. Chang W-C, Wu S-L, Huang W-C, Hsu J-Y, Chan S-H, Wang J-M, et al. PTX3 gene activation in EGF-induced head and neck cancer cell metastasis. Oncotarget. 2015;6: 7741–7757. doi:10.18632/oncotarget.3482

27. Guo Y, Pan WK, Wang ZW, Su WH, Xu K, Jia H, et al. Identification of novel biomarkers for predicting prognosis and immunotherapy response in head and neck squamous cell carcinoma based on ceRNA network and immune infiltration analysis. Biomed Res Int. 2021;2021: 4532438. doi:10.1155/2021/4532438

28. Natarajan J, Hunter K, Mutalik VS, Radhakrishnan R. Overexpression of S100A4 as a biomarker of metastasis and recurrence in oral squamous cell carcinoma. J Appl Oral Sci. 2014;22: 426–433. doi:10.1590/1678-775720140133

29. Moriyama-Kita M, Endo Y, Yonemura Y, Heizmann CW, Schäfer BW, Sasaki T, et al. Correlation of S100A4 expression with invasion and metastasis in oral squamous cell carcinoma. Oral Oncol. 2004;40: 496–500. doi:10.1016/j.oraloncology.2003.10.003

30. Zhang J, Nakamura T, Ito R, Ohbayashi C, Maeda S. The evaluation of both metastasis and prognosis of oral squamous cell carcinoma by S100A4 and E-cadherin immunostaining. Oral Med Pathol. 2004;9: 67–74. doi:10.3353/omp.9.67

31. Park W-Y, Gray JM, Holewinski RJ, Andresson T, So JY, Carmona-Rivera C, et al. Apoptosis-induced nuclear expulsion in tumor cells drives S100a4-mediated metastatic outgrowth through the RAGE pathway. Nat Cancer. 2023;4: 419–435. doi:10.1038/s43018-023-00524-z

32. Condamine T, Dominguez GA, Youn J-I, Kossenkov AV, Mony S, Alicea-Torres K, et al. Lectin-type oxidized LDL receptor-1 distinguishes population of human polymorphonuclear myeloid-derived suppressor cells in cancer patients. Sci Immunol. 2016;1. doi:10.1126/sciimmunol.aaf8943

33. Gallenkamp J, Spanier G, Wörle E, Englbrecht M, Kirschfink M, Greslechner R, et al. A novel multiplex detection array revealed systemic complement activation in oral squamous cell carcinoma. Oncotarget. 2018;9: 3001–3013. doi:10.18632/oncotarget.22963

34. Jing F, Mu J, Liu J, Hu C, Wu F, Gao Q. Senescent vascular endothelial cells promote oral squamous cell carcinoma progression through complement C3 activation. Arch Oral Biol. 2025;174: 106242. doi:10.1016/j.archoralbio.2025.106242

35. Rezvani K. UBXD proteins: A family of proteins with diverse functions in cancer. Int J Mol Sci. 2016;17: 1724. doi:10.3390/ijms17101724

36. Ketkar H, Harrison AG, Graziano VR, Geng T, Yang L, Vella AT, et al. UBX domain protein 6 positively regulates JAK-STAT1/2 signaling. J Immunol. 2021;206: 2682–2691. doi:10.4049/jimmunol.1901337

37. Broutian TR, Jiang B, Li J, Akagi K, Gui S, Zhou Z, et al. Human papillomavirus insertions identify the PIM family of serine/threonine kinases as targetable driver genes in head and neck squamous cell carcinoma. Cancer Lett. 2020;476: 23–33. doi:10.1016/j.canlet.2020.01.012

38. Etemad-Moghadam S, Khalili M, Tirgary F, Alaeddini M. Evaluation of myofibroblasts in oral epithelial dysplasia and squamous cell carcinoma. J Oral Pathol Med. 2009;38: 639–643. doi:10.1111/j.1600-0714.2009.00768.x

39. Hnia K, Ramspacher C, Vermot J, Laporte J. Desmin in muscle and associated diseases: beyond the structural function. Cell Tissue Res. 2015;360: 591–608. doi:10.1007/s00441-014-2016-4

40. Kros JM, Zeneyedpour L, Pedrosa RMSM, Belcaid Z, Dik WA, Luider TM, et al. T cell induced expression of Coronin-1A facilitates blood-brain barrier transmigration of breast cancer cells. Sci Rep. 2024;14: 31516. doi:10.1038/s41598-024-83301-x

41. Oku T, Kaneko Y, Murofushi K, Seyama Y, Toyoshima S, Tsuji T. Phorbol ester-dependent phosphorylation regulates the association of p57/coronin-1 with the actin cytoskeleton. J Biol Chem. 2008;283: 28918–28925. doi:10.1074/jbc.M709990200

42. Rahmati Nezhad P, Riihilä P, Knuutila JS, Viiklepp K, Peltonen S, Kallajoki M, et al. Complement factor D is a novel biomarker and putative therapeutic target in cutaneous squamous cell carcinoma. Cancers (Basel). 2022;14: 305. doi:10.3390/cancers14020305

43. Ezure T, Sugahara M, Amano S. Senescent dermal fibroblasts negatively influence fibroblast extracellular matrix-related gene expression partly via secretion of complement factor D. Biofactors. 2019;45: 556–562. doi:10.1002/biof.1512

44. You P, Wang D, Liu Z, Guan S, Xiao N, Chen H, et al. Knockdown of RFC4 inhibits cell proliferation of oral squamous cell carcinoma in vitro and in vivo. FEBS Open Bio. 2025;15: 346–358. doi:10.1002/2211-5463.13929

45. Hou Y, Wang S, Zhang Y, Huang X, Zhang X, He F, et al. Proteomics identifies LUC7L3 as a prognostic biomarker for hepatocellular carcinoma. Curr Issues Mol Biol. 2024;46: 4004–4020. doi:10.3390/cimb46050247

46. Solomon-Zemler R, Pozniak Y, Geiger T, Werner H. Identification of nucleolar protein NOM1 as a novel nuclear IGF1R-interacting protein. Mol Genet Metab. 2019;126: 259–265. doi:10.1016/j.ymgme.2019.01.002

47. Simmons HM, Ruis BL, Kapoor M, Hudacek AW, Conklin KF. Identification of NOM1, a nucleolar, eIF4A binding protein encoded within the chromosome 7q36 breakpoint region targeted in cases of pediatric acute myeloid leukemia. Gene. 2005;347: 137–145. doi:10.1016/j.gene.2004.12.027

48. Dong C, Dong G, Li L, Zhu L, Tempel W, Liu Y, et al. An asparagine/glycine switch governs product specificity of human N-terminal methyltransferase NTMT2. Commun Biol. 2018;1: 183. doi:10.1038/s42003-018-0196-2

49. Tan L, Li W, Su Q. The comprehensive analysis of the prognostic and functional role of N-terminal methyltransferases 1 in pan-cancer. PeerJ. 2023;11: e16263. doi:10.7717/peerj.16263

50. Chen P, Huang R, Hazbun TR. Unlocking the mysteries of alpha-N-terminal methylation and its diverse regulatory functions. J Biol Chem. 2023;299: 104843. doi:10.1016/j.jbc.2023.104843

51. Zhao C, Yu M, Li Y. Pan-cancer analysis reveals the pro-oncogenic role of N6-methyladenosine (m6A)-regulated NTMT1 in head and neck squamous cell carcinoma. J Biochem Mol Toxicol. 2024;38: e23603. doi:10.1002/jbt.23603

52. Bonsignore LA, Butler JS, Klinge CM, Schaner Tooley CE. Loss of the N-terminal methyltransferase NRMT1 increases sensitivity to DNA damage and promotes mammary oncogenesis. Oncotarget. 2015;6: 12248–12263. doi:10.18632/oncotarget.3653

53. Shields KM, Tooley JG, Petkowski JJ, Wilkey DW, Garbett NC, Merchant ML, et al. Select human cancer mutants of NRMT1 alter its catalytic activity and decrease N-terminal trimethylation. Protein Sci. 2017;26: 1639–1652. doi:10.1002/pro.3202

54. Faughn JD, Dean WL, Schaner Tooley CE. The N-terminal methyltransferase homologs NRMT1 and NRMT2 exhibit novel regulation of activity through heterotrimer formation. Protein Sci. 2018;27: 1585–1599. doi:10.1002/pro.3456

55. Petkowski JJ, Schaner Tooley CE, Anderson LC, Shumilin IA, Balsbaugh JL, Shabanowitz J, et al. Substrate specificity of mammalian N-terminal α-amino methyltransferase NRMT. Biochemistry. 2012;51: 5942–5950. doi:10.1021/bi300278f

56. Shen C, Han C, Li Z, Yan Y, Li C, Chen H, et al. Construction and validation of a prognostic model based on pyroptosis-related genes in bladder cancer. Comb Chem High Throughput Screen. 2024;27: 2335–2349. doi:10.2174/0113862073256363230929200157

57. Wang X-W, Madeddu L, Spirohn K, Martini L, Fazzone A, Becchetti L, et al. Assessment of community efforts to advance network-based prediction of protein-protein interactions. Nat Commun. 2023;14: 1582. doi:10.1038/s41467-023-37079-7

58. Huttlin EL, Bruckner RJ, Navarrete-Perea J, Cannon JR, Baltier K, Gebreab F, et al. Dual proteome-scale networks reveal cell-specific remodeling of the human interactome. Cell. 2021;184: 3022-3040.e28. doi:10.1016/j.cell.2021.04.011

59. Kamat SS, Camara K, Parsons WH, Chen D-H, Dix MM, Bird TD, et al. Immunomodulatory lysophosphatidylserines are regulated by ABHD16A and ABHD12 interplay. Nat Chem Biol. 2015;11: 164–171. doi:10.1038/nchembio.1721

60. Yan J, Zhang C, Xu Y, Huang Z, Ye Q, Qian X, et al. GPR34 is a metabolic immune checkpoint for ILC1-mediated antitumor immunity. Nat Immunol. 2024;25: 2057–2067. doi:10.1038/s41590-024-01973-z

61. Zhang X, Guo J, Shi X, Zhou X, Chen Q. LUC7L3 is a downstream factor of SRSF1 and prevents genomic instability. Cell Insight. 2024;3: 100170. doi:10.1016/j.cellin.2024.100170

62. Li Y-Y, Mukaida N. Pathophysiological roles of Pim-3 kinase in pancreatic cancer development and progression. World J Gastroenterol. 2014;20: 9392–9404. doi:10.3748/wjg.v20.i28.9392

63. Atalay P, Ozpolat B. PIM3 kinase: A promising novel target in solid cancers. Cancers (Basel). 2024;16. doi:10.3390/cancers16030535

64. Choudhury R, Bahadi CK, Ray IP, Dash P, Pattanaik I, Mishra S, et al. PIM1 kinase and its diverse substrate in solid tumors. Cell Commun Signal. 2024;22: 529. doi:10.1186/s12964-024-01898-y

65. Cervantes-Gomez F, Stellrecht CM, Ayres ML, Keating MJ, Wierda WG, Gandhi V. PIM kinase inhibitor, AZD1208, inhibits protein translation and induces autophagy in primary chronic lymphocytic leukemia cells. Oncotarget. 2019;10: 2793–2809. doi:10.18632/oncotarget.26876

66. Bellon M, Nicot C. Targeting Pim kinases in hematological cancers: molecular and clinical review. Mol Cancer. 2023;22: 18. doi:10.1186/s12943-023-01721-1

67. Luszczak S, Kumar C, Sathyadevan VK, Simpson BS, Gately KA, Whitaker HC, et al. PIM kinase inhibition: co-targeted therapeutic approaches in prostate cancer. Signal Transduct Target Ther. 2020;5: 7. doi:10.1038/s41392-020-0109-y

68. Cortes J, Tamura K, DeAngelo DJ, de Bono J, Lorente D, Minden M, et al. Phase I studies of AZD1208, a proviral integration Moloney virus kinase inhibitor in solid and haematological cancers. Br J Cancer. 2018;118: 1425–1433. doi:10.1038/s41416-018-0082-1

69. Murdocca M, De Masi C, Pucci S, Mango R, Novelli G, Di Natale C, et al. LOX-1 and cancer: an indissoluble liaison. Cancer Gene Ther. 2021;28: 1088–1098. doi:10.1038/s41417-020-00279-0

70. Wu L, Liu Y, Deng W, Wu T, Bu L, Chen L. OLR1 is a pan-cancer prognostic and immunotherapeutic predictor associated with EMT and cuproptosis in HNSCC. Int J Mol Sci. 2023;24. doi:10.3390/ijms241612904

71. Zhang P, Zhao Y, Xia X, Mei S, Huang Y, Zhu Y, et al. Expression of OLR1 gene on tumor-associated macrophages of head and neck squamous cell carcinoma, and its correlation with clinical outcome. Oncoimmunology. 2023;12: 2203073. doi:10.1080/2162402X.2023.2203073

72. Castro F, Cardoso AP, Gonçalves RM, Serre K, Oliveira MJ. Interferon-gamma at the Crossroads of tumor immune surveillance or evasion. Front Immunol. 2018;9: 847. doi:10.3389/fimmu.2018.00847

73. Ding H, Wang G, Yu Z, Sun H, Wang L. Role of interferon-gamma (IFN-γ) and IFN-γ receptor 1/2 (IFNγR1/2) in regulation of immunity, infection, and cancer development: IFN-γ-dependent or independent pathway. Biomed Pharmacother. 2022;155: 113683. doi:10.1016/j.biopha.2022.113683

74. Rosenzweig SD, Schwartz OM, Brown MR, Leto TL, Holland SM. Characterization of a dipeptide motif regulating IFN-gamma receptor 2 plasma membrane accumulation and IFN-gamma responsiveness. J Immunol. 2004;173: 3991–3999. doi:10.4049/jimmunol.173.6.3991

75. Soh J, Donnelly RJ, Kotenko S, Mariano TM, Cook JR, Wang N, et al. Identification and sequence of an accessory factor required for activation of the human interferon gamma receptor. Cell. 1994;76: 793–802. doi:10.1016/0092-8674(94)90354-9

76. Zhang S-F, Wang X-Y, Fu Z-Q, Peng Q-H, Zhang J-Y, Ye F, et al. TXNDC17 promotes paclitaxel resistance via inducing autophagy in ovarian cancer. Autophagy. 2015;11: 225–238. doi:10.1080/15548627.2014.998931

77. Zhang Z, Wang A, Li H, Zhi H, Lu F. RETRACTED: STAT3-dependent TXNDC17 expression mediates Taxol resistance through inducing autophagy in human colorectal cancer cells. Gene. 2016;584: 75–82. doi:10.1016/j.gene.2016.03.012

78. Kocatürk B. Identification of thioredoxin domain containing family members' expression pattern and prognostic value in diffuse gliomas via in silico analysis. Cancer Med. 2023;12: 3830–3844. doi:10.1002/cam4.5169
