# Supplementary material for: Spatial colocalization and molecular crosstalk of myofibroblastic CAFs and tumor cells shape lymph node metastasis in oral squamous cell carcinoma
Source: PLoS Genet. 2025 Sep 4;21(9):e1011791. doi: 10.1371/journal.pgen.1011791 (PMC12410789; doi:10.1371/journal.pgen.1011791)
Supplement: S14 Table — The percentages in each category are based on the total number of cases with sufficient information. The significance of continuous variables was evaluated using the 2-sided Mann–Whitney U test, while the 2-sided Fisher exact test was used for categorical variables. Abbreviations: IQR, interquartile range; OSCC, oral squamous cell carcinoma. (PDF) [file pgen.1011791.s015.pdf]

S14 Table. Clinical characteristics of 214 patients with OSCC (related to Fig 9).

**Characteristic**		Low score (N = 107)		High score (N = 107)		P^*
**Median age at diagnosis, years (IQR)**		60	(53–67)	60	(52–69)	0.32
**Sex, *n* (%)**	Female	29	(27)	36	(34)	0.45
	Male	78	(73)	71	(66)	
**Pathologic T stage, n (%)**	T1	14	(13)	4	(4)	<0.001
	T2	47	(44)	21	(20)	
	T3	24	(22)	31	(29)	
	T4	22	(21)	51	(47)	
**Pathologic N stage, n (%)**	N0	53	(50)	37	(35)	<0.001
	N1	25	(23)	10	(9)	
	N2a-b	26	(24)	40	(37)	
	N2c and N3	3	(3)	20	(19)	
**Tissue origin, n (%)**	Tongue	6	(63)	54	(50)	0.47
	Floor of mouth	22	(20)	24	(22)	
	Cheek mucosa	6	(6)	11	(10)	
	Mouth	6	(6)	10	(9)	
	Gum	4	(4)	6	(6)	
	Palate	2	(2)	2	(2)	

Table Legend

The percentages in each category are based on the total number of cases with sufficient information.

* The significance of continuous variables was evaluated using the 2-sided Mann–Whitney *U* test, while the 2-sided Fisher exact test was used for categorical variables.

Abbreviations: IQR, interquartile range; OSCC, oral squamous cell carcinoma.
