# Supplementary material for: Spatial colocalization and molecular crosstalk of myofibroblastic CAFs and tumor cells shape lymph node metastasis in oral squamous cell carcinoma
Source: PLoS Genet. 2025 Sep 4;21(9):e1011791. doi: 10.1371/journal.pgen.1011791 (PMC12410789; doi:10.1371/journal.pgen.1011791)
Supplement: S15 Table — Abbreviations: DSS, disease-specific survival; OS, overall survival; TCGA, The Cancer Genome Atlas. (PDF) [file pgen.1011791.s016.pdf]

S15 Table. Comparison of five-year survival rates between low and high score groups across all cohorts (related to Fig 9).

			Median score	Five-year survival rate		*P* values by log-rank tests
				Low score	High score	
**TCGA**	OS	(*n* = 214)	0.173	0.8	0.14	<0.001
	DSS	(*n* = 209)	0.172	0.81	0.28	<0.001
**GSE41613**	OS	(*n* = 96)	−0.057	0.66	0.37	0.002
	DSS	(*n* = 76)	−0.050	0.79	0.42	<0.001
**GSE42743**	OS	(*n* = 50)	0.087	0.6	0.32	0.326
	DSS	(*n* = 37)	0.077	0.94	0.4	0.021
**Combined datasets**	OS	(*n* = 146)	−0.015	0.65	0.35	0.002
	DSS	(*n* = 113)	−0.035	0.78	0.45	<0.001

Table Legend

Abbreviations: DSS, disease-specific survival; OS, overall survival; TCGA, The Cancer Genome Atlas.
