# Supplementary material for: Spatial colocalization and molecular crosstalk of myofibroblastic CAFs and tumor cells shape lymph node metastasis in oral squamous cell carcinoma
Source: PLoS Genet. 2025 Sep 4;21(9):e1011791. doi: 10.1371/journal.pgen.1011791 (PMC12410789; doi:10.1371/journal.pgen.1011791)

Supporting Figure 1

A

Bulk WES & RNA-seq analysis

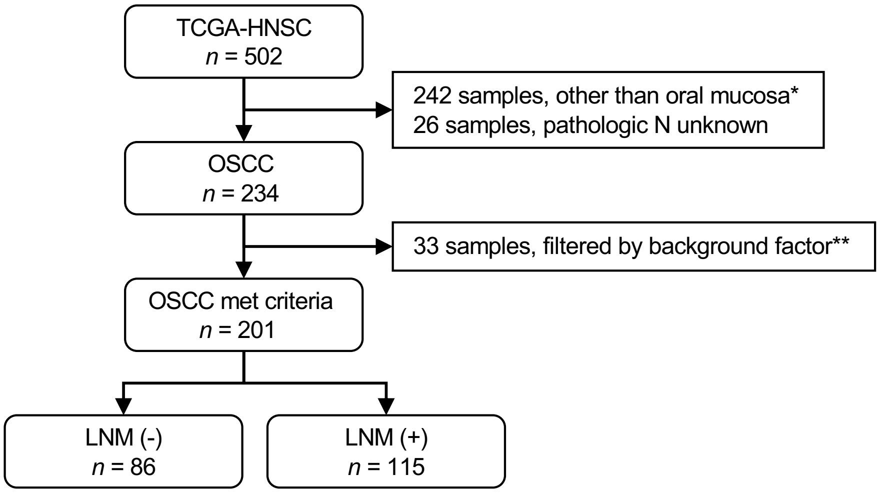

B

Characteristic		LNM (-) (*n* = 86)		LNM (+) (*n* = 115)		*P*-value*
Median age at diagnosis, years (IQR)		59	52 - 65	60	51 - 67	0.86
Sex, n (%)	Female	33	(38)	27	(23)	0.03
	Male	53	(62)	88	(77)	
Pathologic T stage, n (%)	T1	10	(12)	5	(4)	0.05
	T2	32	(37)	34	(30)	
	T3	15	(17)	35	(30)	
	T4	29	(34)	41	(36)	
Pathologic N stage, n (%)	N0	86	(100)	0	(0)	
	N1	0	(0)	33	(29)	
	N2	0	(0)	80	(69)	
	N3	0	(0)	2	(2)	
Tissue origin, n (%)	Tongue	47	(55)	65	(57)	0.93
	Floor of mouth	17	(20)	26	(23)	
	Cheek mucosa	7	(8)	10	(9)	
	Mouth	8	(9)	8	(7)	
	Gum	5	(6)	4	(3)	
	Palate	2	(2)	2	(2)	

Abbreviation: LNM, lymph node metastasis.
Percentages in each given category are based on the total number of cases with sufficient information.
*The significance of continuous variables was evaluated using the two-sided Mann–Whitney *U* test, while the two-sided Fisher's exact test was utilized for categorical variables.

Supporting Figure 1 (Continued)

C

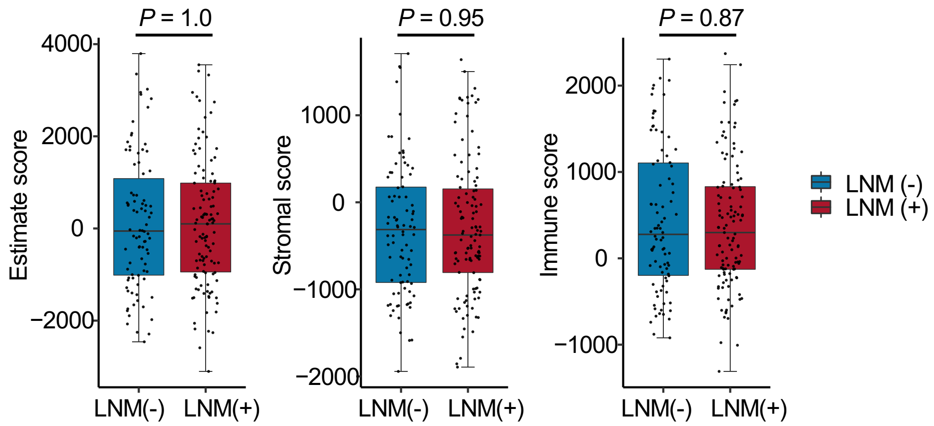

Supplement: S1 Fig — (A) Schematic representation of the patient selection process for bulk whole-exome sequencing (WES) and RNA sequencing (RNA-seq) analysis, excluding the larynx, tonsil, hypopharynx, oropharynx, lip, bones, joints, articular cartilage of other and undefined sites, and ill-defined sites in the lip and oral cavity. Specific exclusions included basaloid squamous cell carcinoma samples, human papilloma virus (HPV)-positive samples, and samples from patients with prior malignancies. (B) Clinical characteristics of a set of 201 patients with OSCC. The significance of the continuous variables was evaluated using the 2-sided Mann-Whitney U test, and the significance of the categorical variables was evaluated using the 2-sided Fisher exact test. (C) Boxplots denoting the Estimate score, Stromal score and Immune score of 201 patients with OSCC, with LNM (red) and without LNM (blue). Black circles represent individual samples. The center lines represent the medians, the box borders represent the interquartile ranges (IQRs), and the whiskers represent ± 1.5 × IQRs. Statistical significance (P < 0.05) was evaluated using the 2-sided Mann-Whitney U test. (PDF) [file pgen.1011791.s017.pdf]
