# Supplementary material for: Spatial colocalization and molecular crosstalk of myofibroblastic CAFs and tumor cells shape lymph node metastasis in oral squamous cell carcinoma
Source: PLoS Genet. 2025 Sep 4;21(9):e1011791. doi: 10.1371/journal.pgen.1011791 (PMC12410789; doi:10.1371/journal.pgen.1011791)

Supporting Figure 2

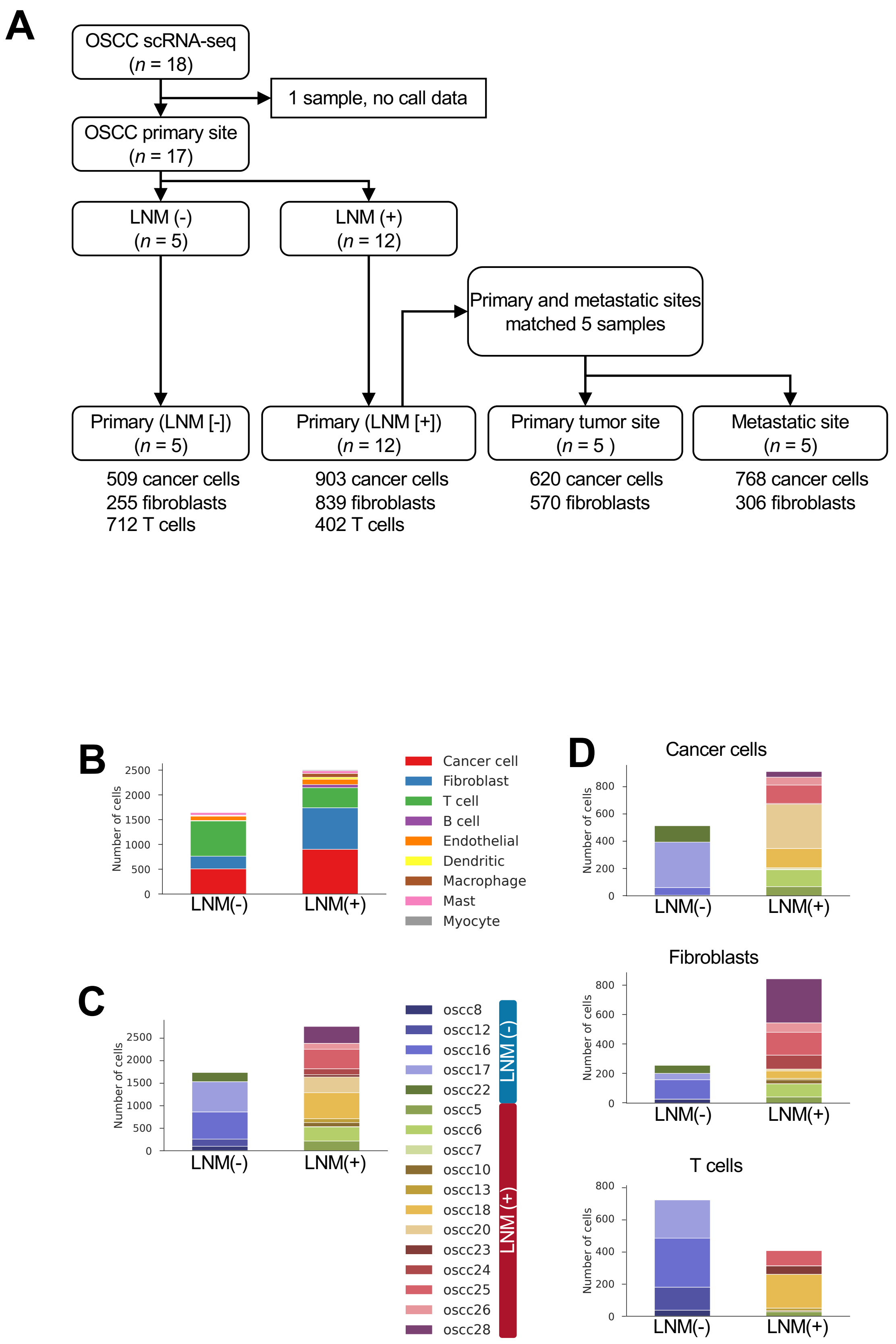

Supporting Figure 2 (continued)

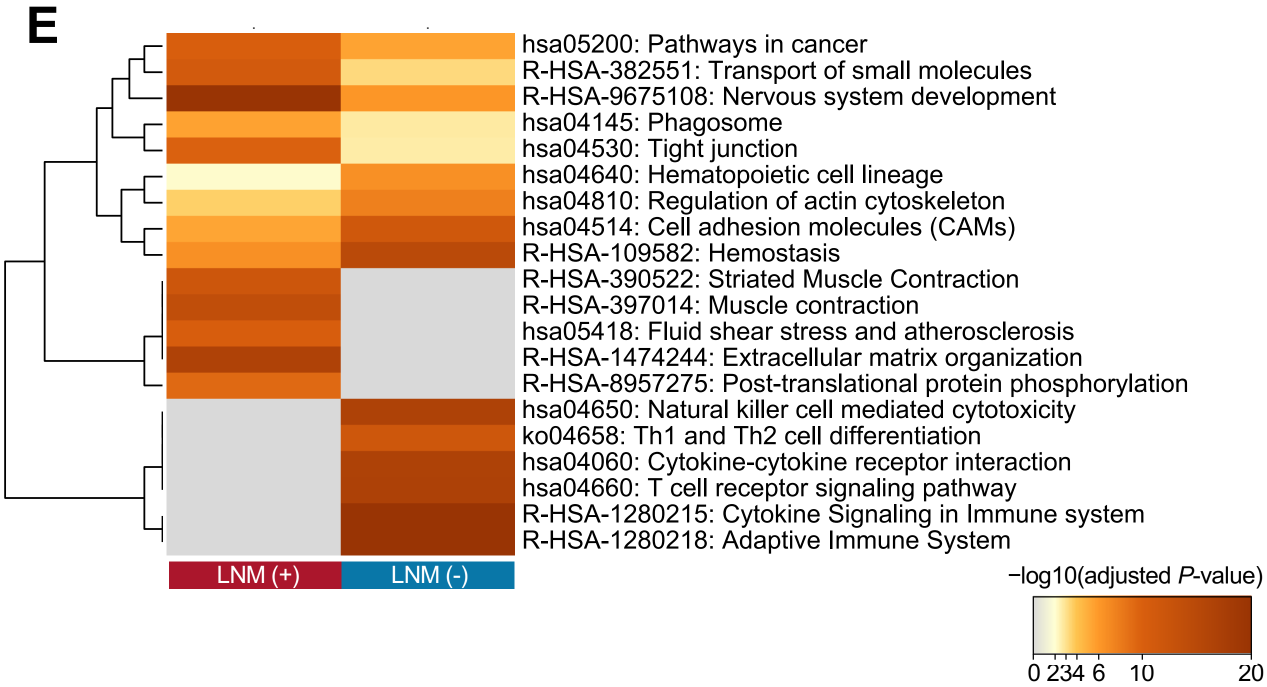

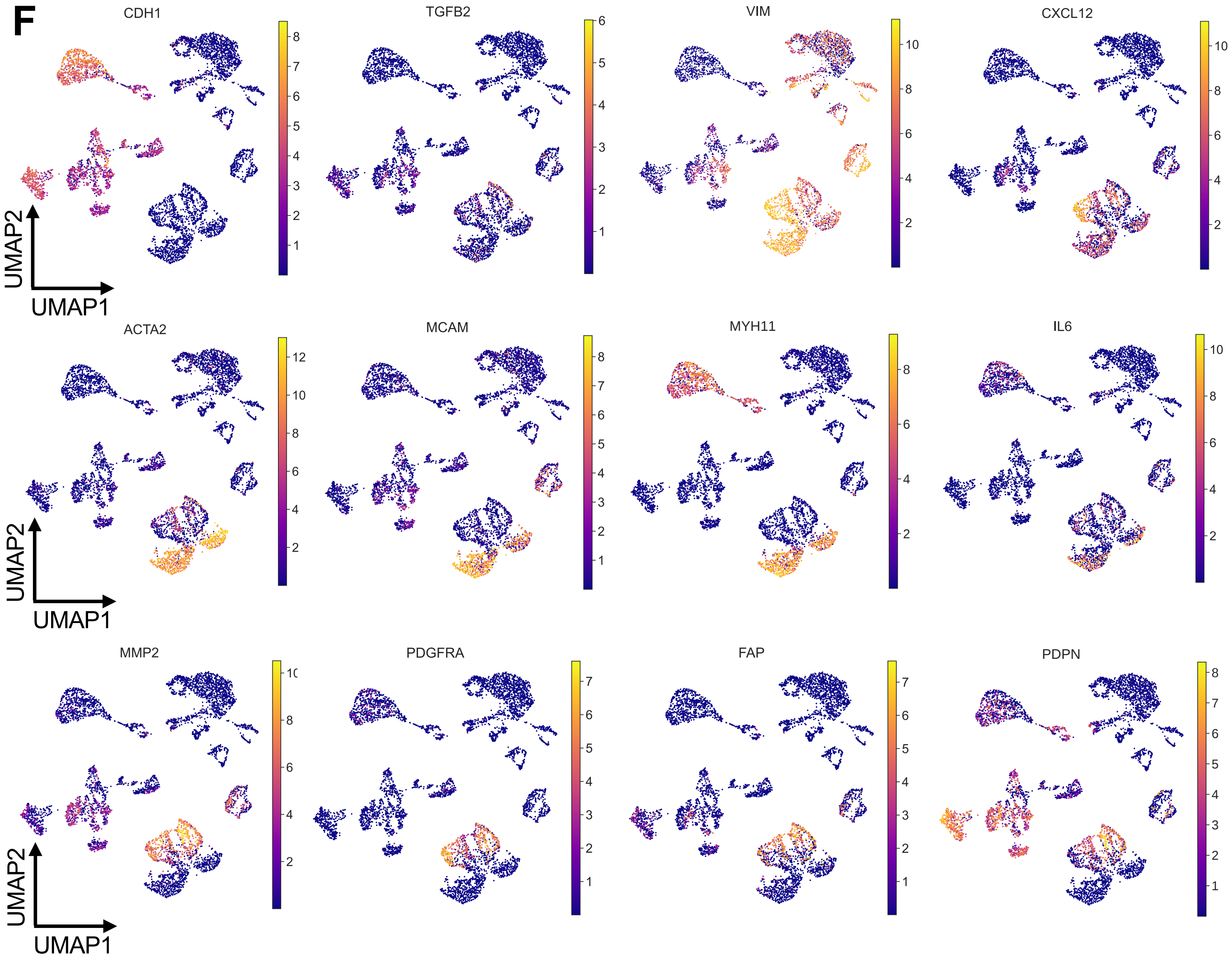

Supporting Figure 2 (continued)

G

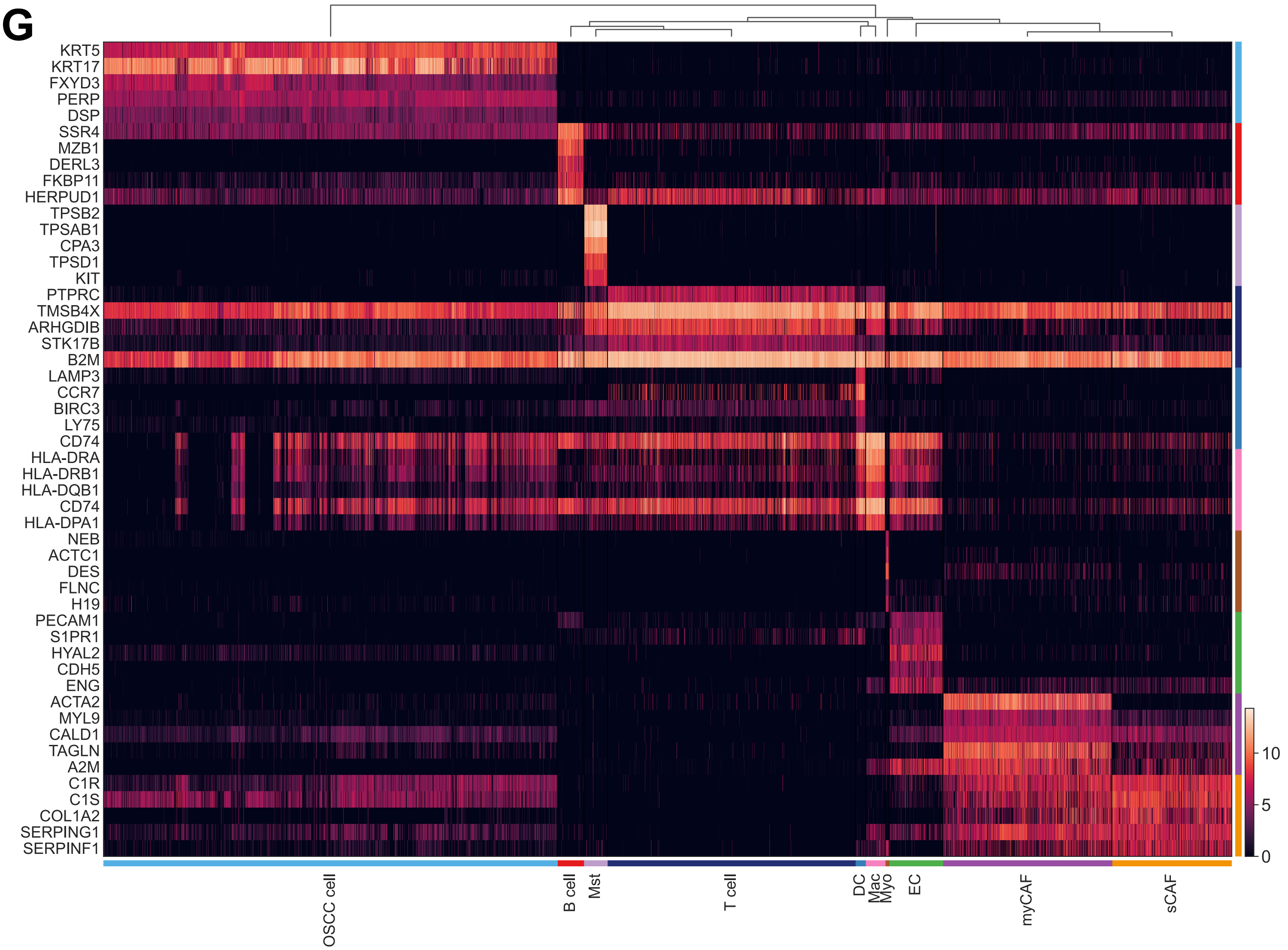

H

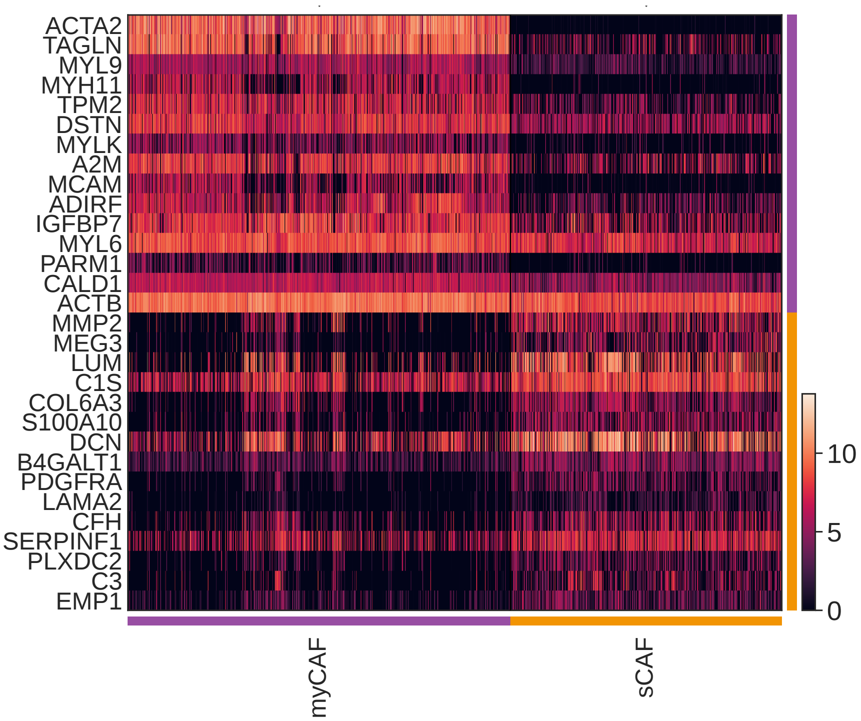

Supporting Figure 2 (continued)

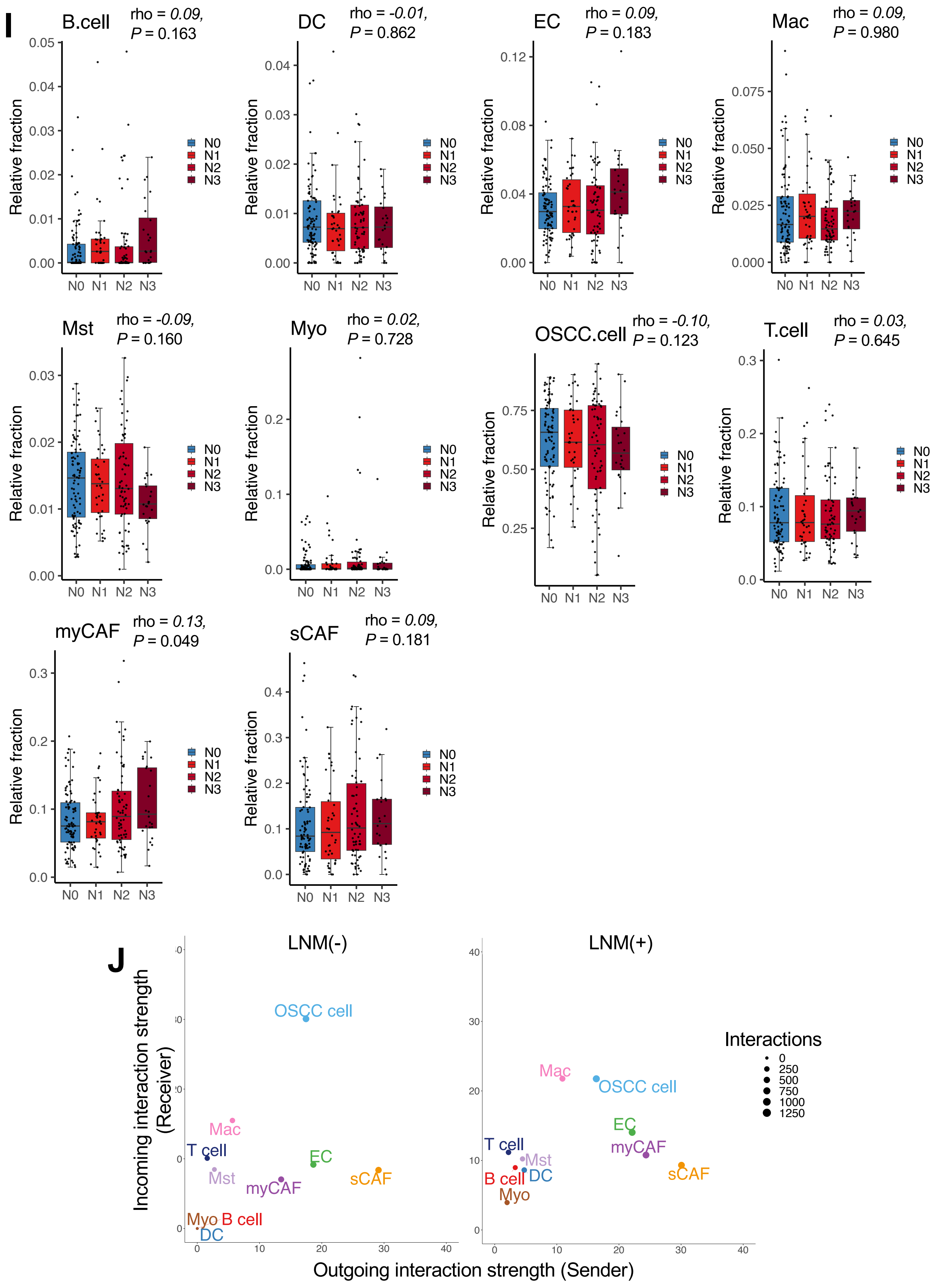

Supporting Figure 2 (continued)

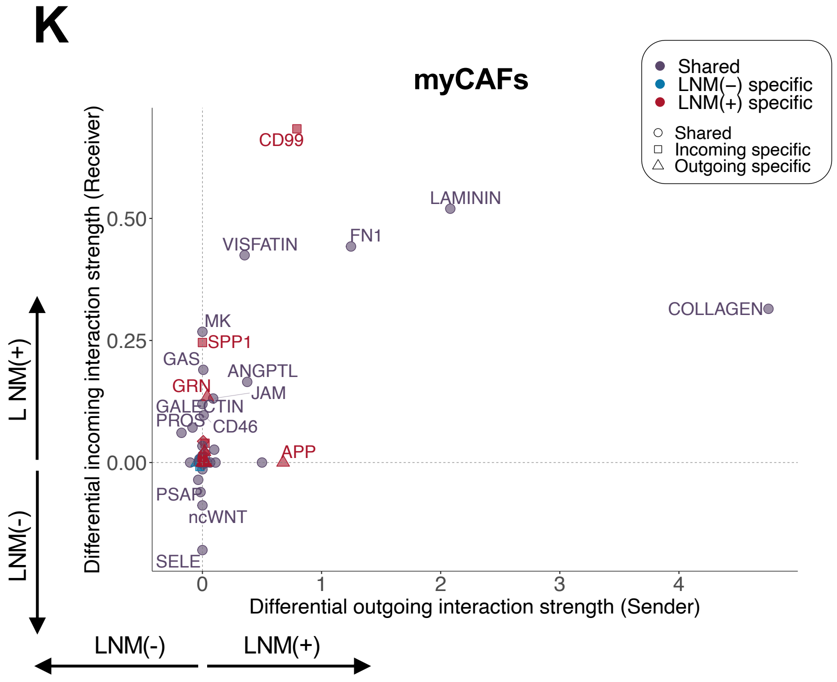

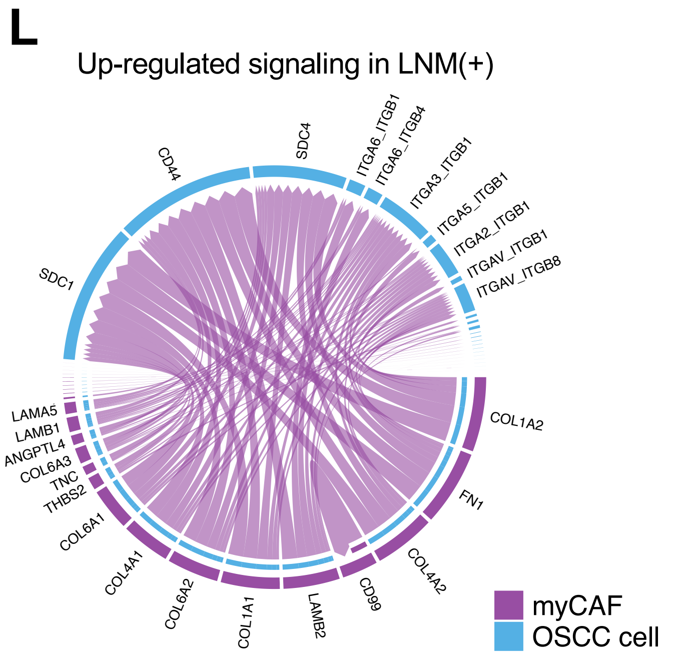

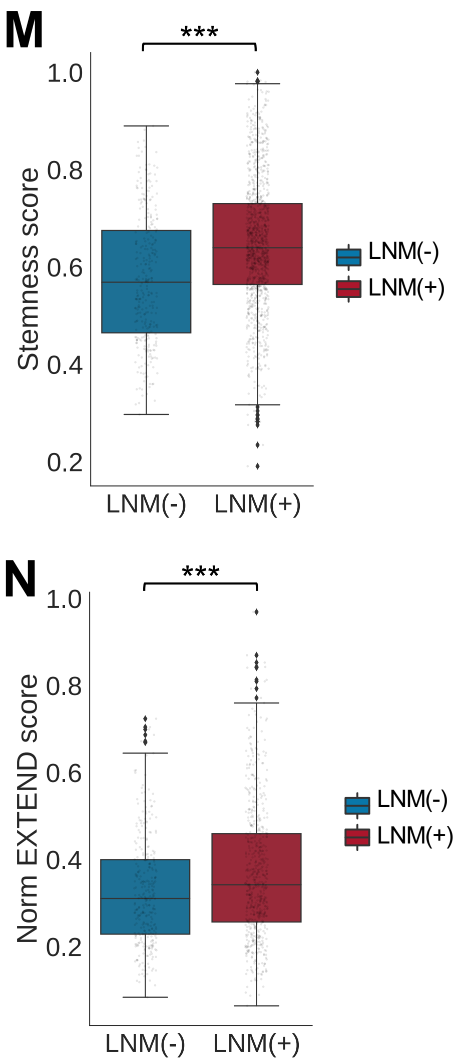

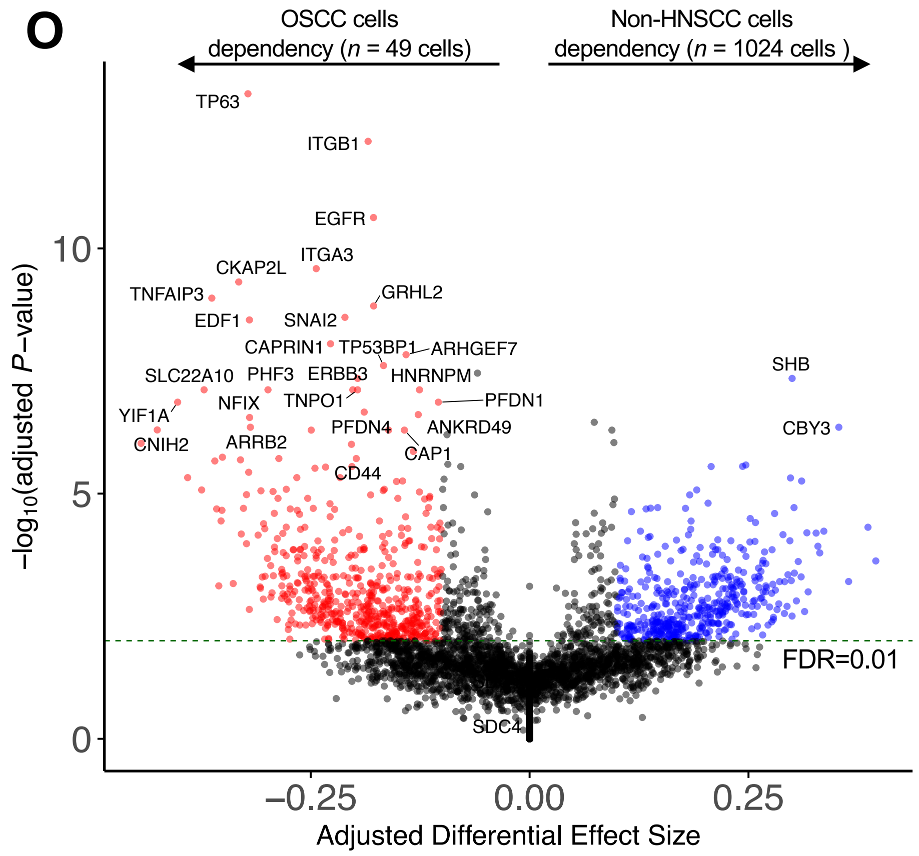

Supplement: S2 Fig — (A) Overview of patients included in the single-cell RNA-seq (scRNA-seq) analysis. (B-D) Comparison of cell numbers in lymph node metastasis (LNM) and non-LNM groups based on the oral squamous cell carcinoma (OSCC) single-cell RNA sequencing (scRNA-seq) data. The comparison involved (B) cell types, (C) sample IDs, and (D) the number of cancer cells, fibroblasts, and T cells. (E) Heatmap illustrating the statistically significant pathways enriched in differentially expressed genes (DEGs) between the LNM and non-LNM groups. Colors denote adjusted P-values. (F) Uniform manifold approximation and projection (UMAP) visualizations represent 5,884 cells from 17 OSCC samples analyzed through scRNA-seq and show the expression of CDH1, TGFβ2, VIM, CXCL12, ACTA2, MCAM, MYH11, IL6, MMP2, PDGFRA, FAP, and PDPN. (G) Heatmap displaying the top 5 DEGs for each cell type. The cell types are as follows: B cells, dendritic cells (DCs), endothelial cells (ECs), macrophages (Macs), mast cells (Msts), myocytes (Myos), OSCC (oral squamous cell carcinoma) cells, T cells, myofibroblastic cancer-associated fibroblasts (myCAFs), and secretory/matrix-remodeling cancer-associated fibroblasts (sCAFs). (H) Heatmap depicting the top 15 DEGs between myCAFs and sCAFs. (I) Relative fractions of the 10 cell types within each sample from 201 patients with OSCC were compared across different LNM stages. The fractions were estimated using a CIBERSORTx analysis. The fractions for the OSCC cells, myCAFs, sCAFs, and T cells are also shown in Fig 3G. (J) Scatter plot comparing interaction strengths for each cell type in LNM-positive vs. LNM-negative samples. Refer to Fig 4B. (K) Scatter plot illustrating differential interaction strengths in myCAFs in LNM-positive vs. LNM-negative samples. (L) Circos plot depicting upregulated outgoing signaling from myCAFs to OSCC cells in LNM-positive samples. (M and N) Box plots illustrate the cancer stemness score [21] and expression-based telomerase enzymatic activity d [file pgen.1011791.s018.pdf]
