# Supplementary material for: Spatial colocalization and molecular crosstalk of myofibroblastic CAFs and tumor cells shape lymph node metastasis in oral squamous cell carcinoma
Source: PLoS Genet. 2025 Sep 4;21(9):e1011791. doi: 10.1371/journal.pgen.1011791 (PMC12410789; doi:10.1371/journal.pgen.1011791)

Supporting Figure 3

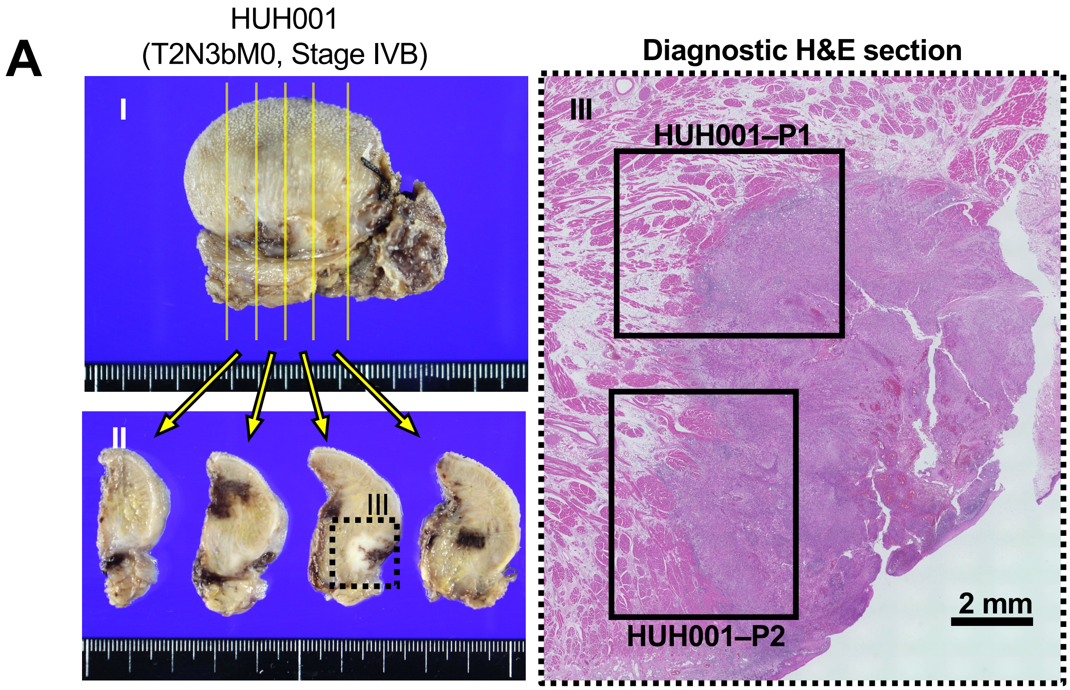

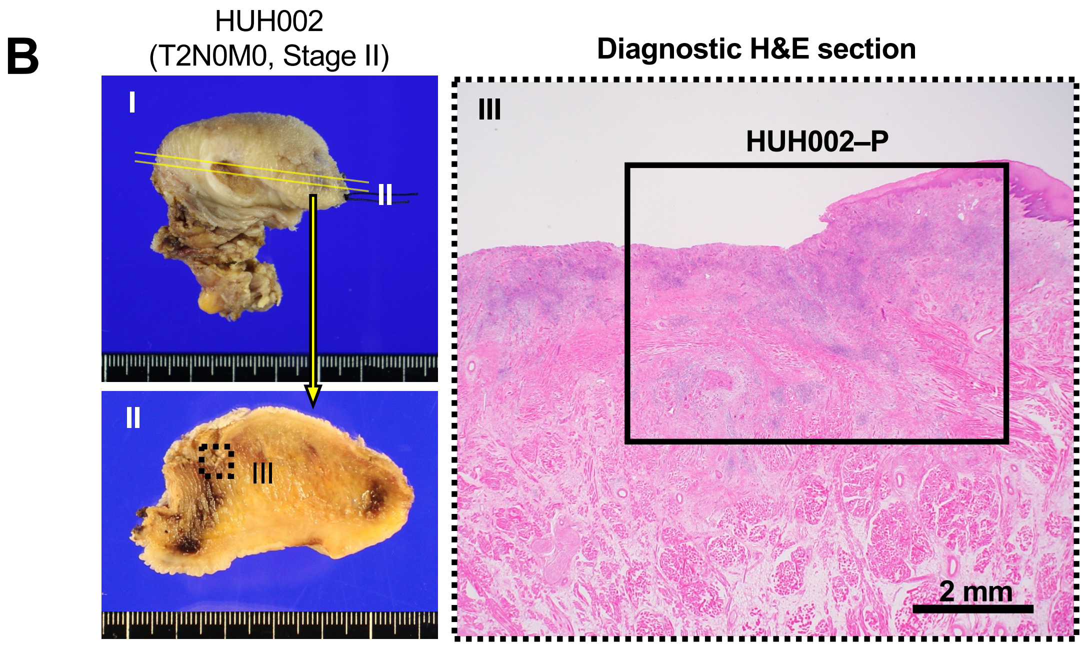

Supporting Figure 3 (continued)

C

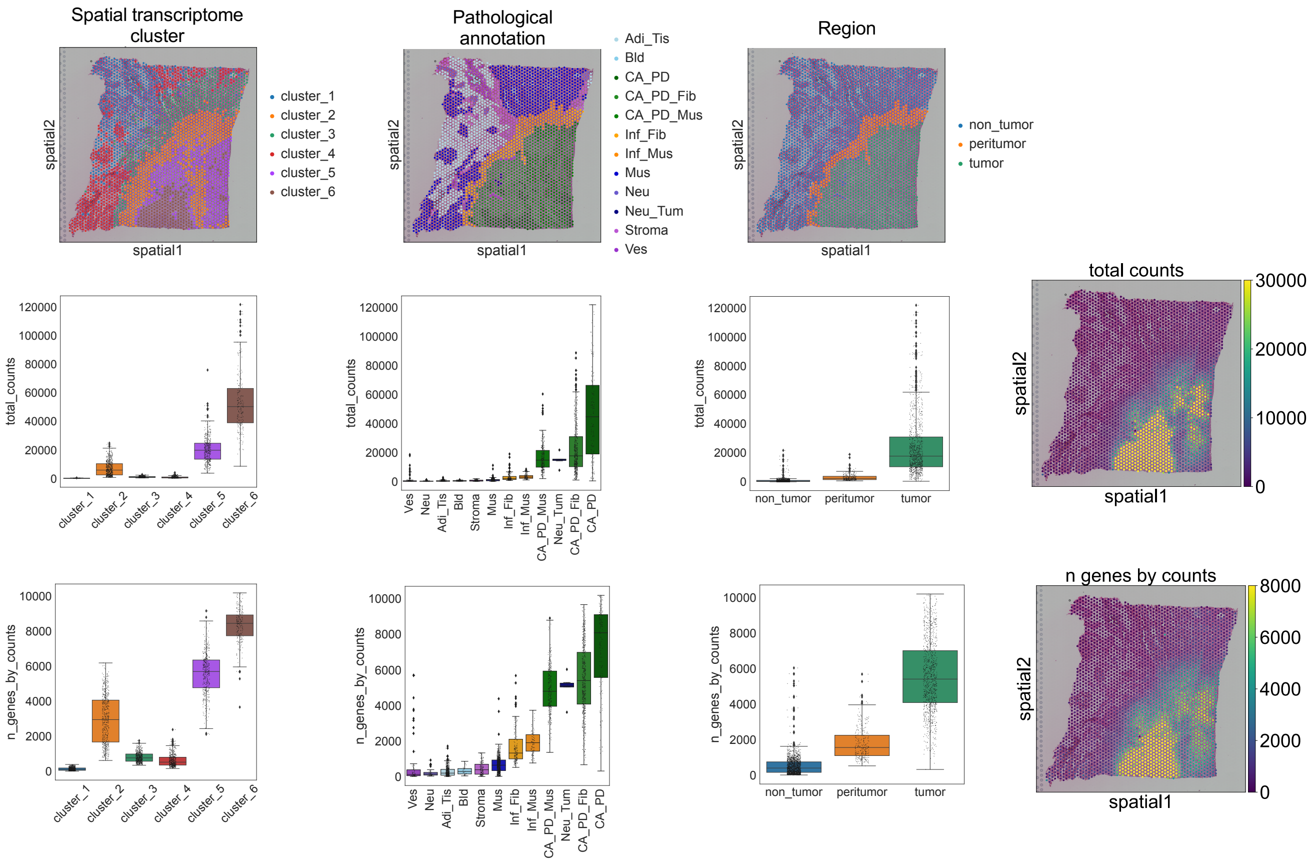

D

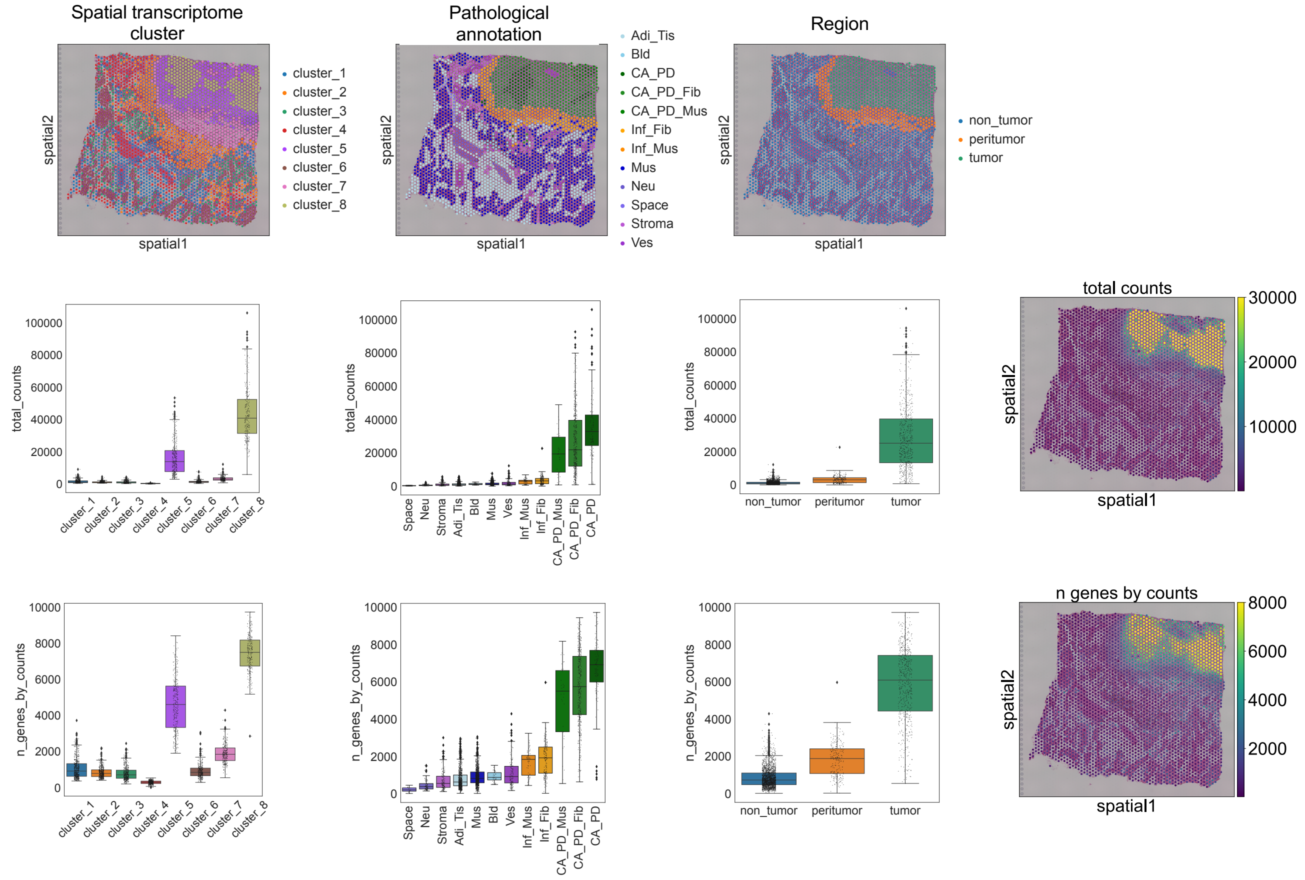

Supporting Figure 3 (continued)

E

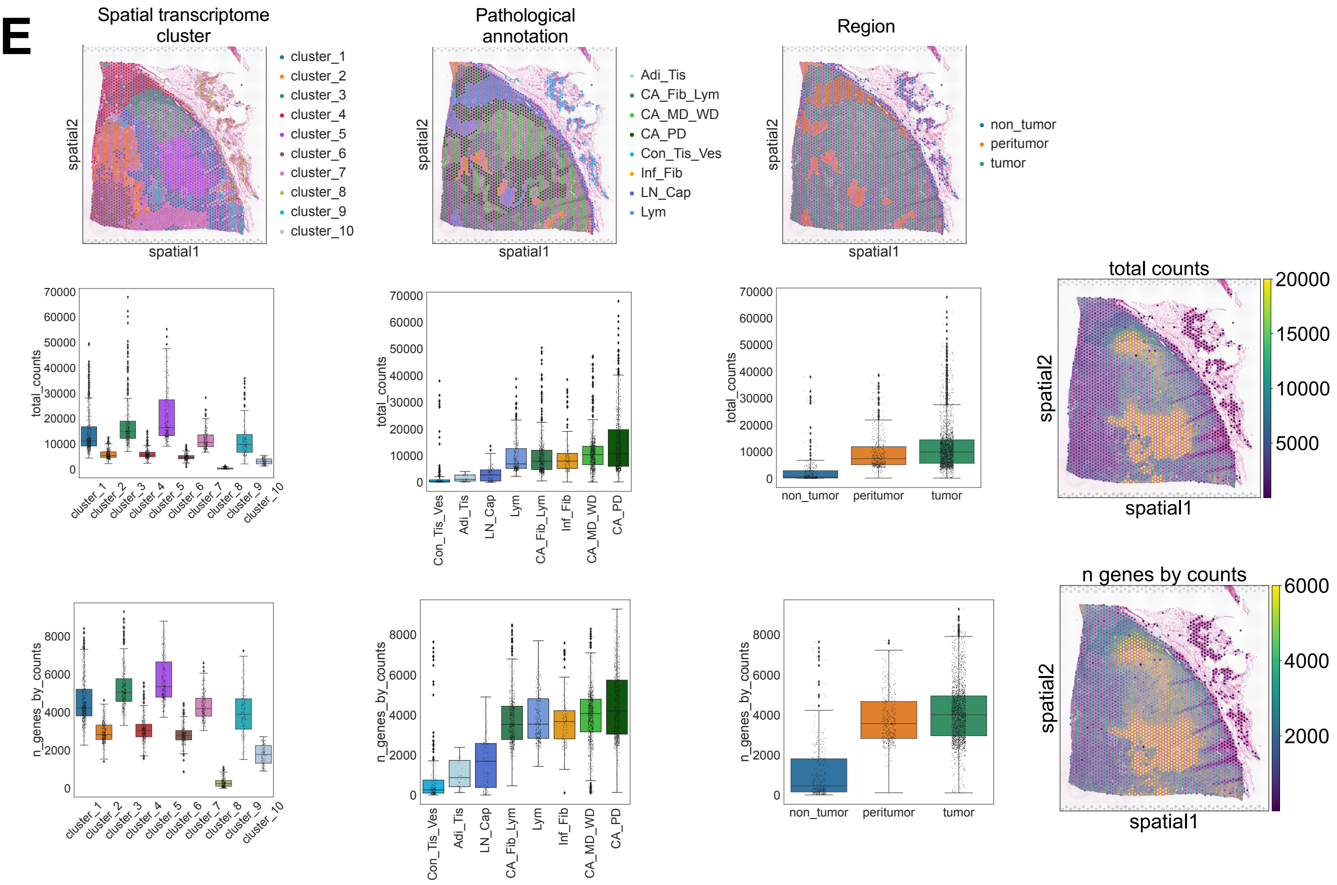

F

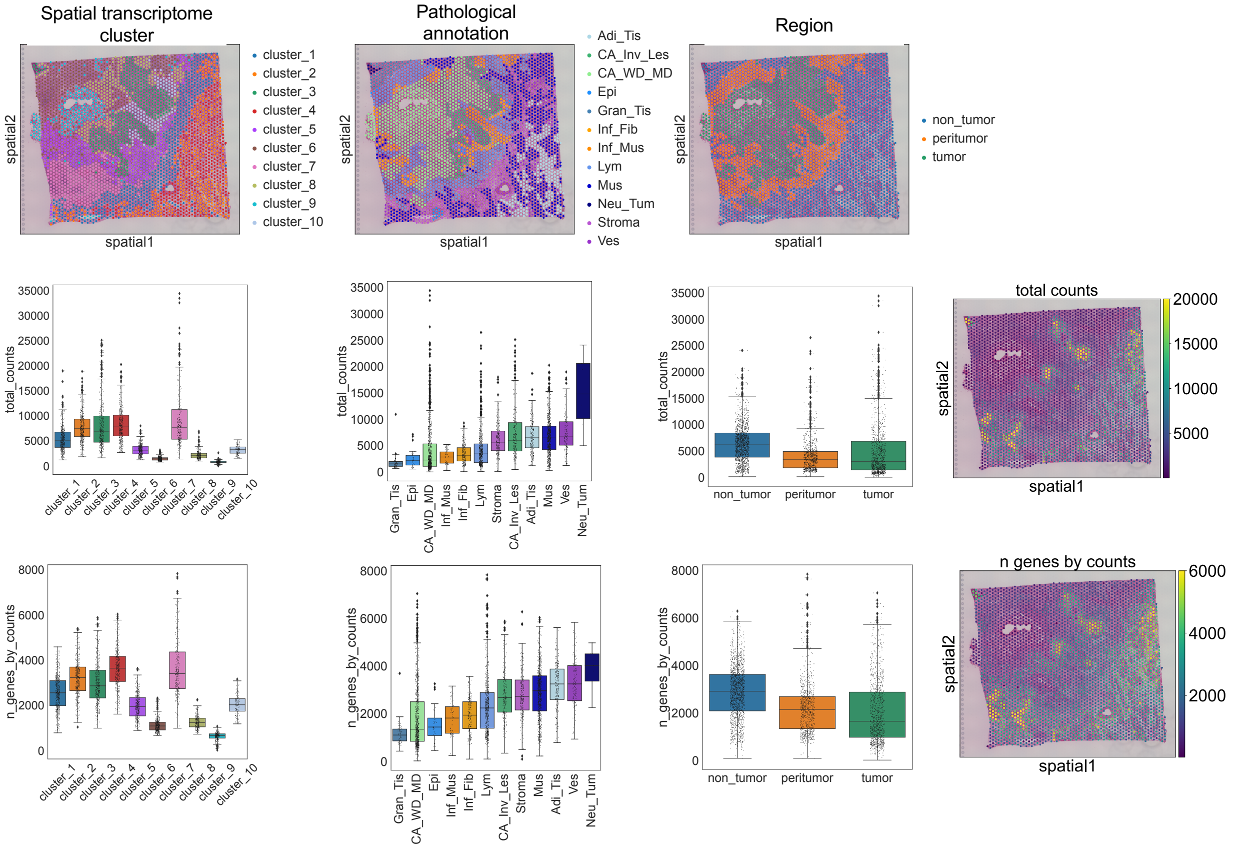

Supporting Figure 3 (continued)

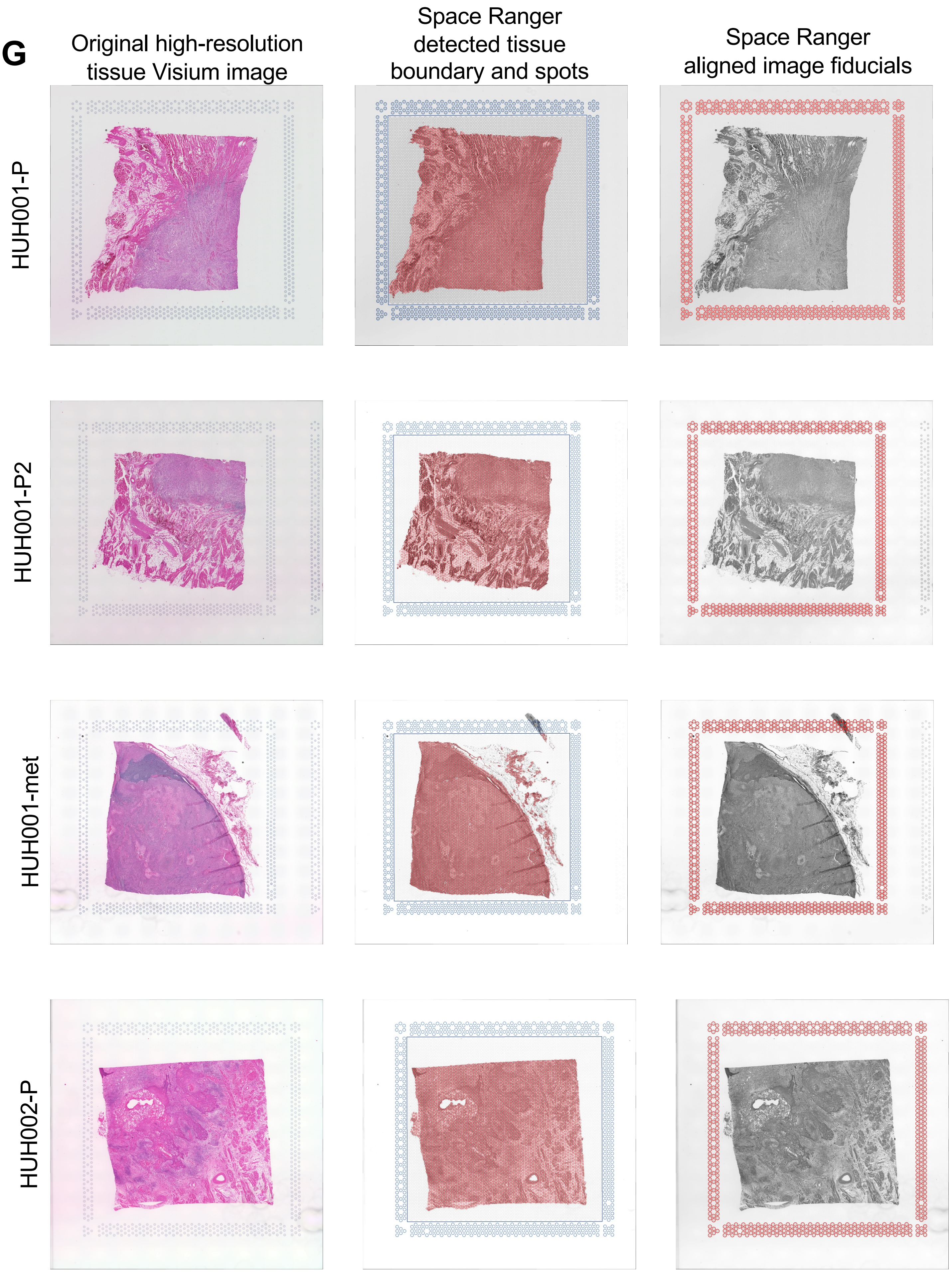

Supplement: S3 Fig — (A-B) Formalin-fixed, paraffin-embedded (FFPE) sample sections were btained from the largest cross-sections of tumors. (A) Two primary tissues (HUH001-P1 and HUH001-P2) from patient HUH001 with LNM. (B) One primary tissue from patient HUH002 without LNM (HUH002-P). Refer to Fig 5B. The adjacent sections from the same specimen were used for diagnostic hematoxylin and eosin (H&E)/ immunohistochemical (IHC) images and Visium images, respectively (see also FFPE sample preparation and H&E-stained images for spatial transcriptome analysis in the Methods). (C-F) Spatial transcriptomes for each sample are displayed. On the top left, unsupervised clustering based on spatial transcriptomes are provided. On the top central, histopathological annotations by specialists are provided and detail tissue states and compositions: CA_WD_MD, cancer, well-differentiated/moderately differentiated; CA_MD_WD, cancer, moderately differentiated/well-differentiated; CA_Inv_Les, cancer, invasive lesions; CA_Fib_Lym, cancer, fibrosis, lymphocytes; CA_PD_Fib, cancer, poorly differentiated fibrosis; CA_PD_Mus, cancer, poorly differentiated muscle; CA_PD, cancer, poorly differentiated; Inf_Fib, inflammation, fibrosis; Inf_Mus, inflammation, muscle; Adi_Tis, adipose tissue; Bld, blood; Con_Tis_Ves, connective tissue, vessels; Epi, epithelium; Gran_Tis, granulation, tissue; LN_Cap, lymph node capillaries; Lym, lymphocytes; Mus, muscle; Neu, neurons; Neu_Tum, neuron tumor; Uncat, uncategorised; and Ves, vessels. These histopathological annotations are broadly characterized into shades of green, representing tumor regions; shades of yellow, indicating peritumor regions; and shades of blue, indicating nontumor regions. On the top right, broad histopathological categorizations for tumor (green), peritumor (yellow), and nontumor (blue) regions are exhibited. The upper right panel displays the total unique molecular identifiers (UMIs), and lower right panel showcases gene expression by UMI counts. The box [file pgen.1011791.s019.pdf]
