# Supplementary material for: Spatial colocalization and molecular crosstalk of myofibroblastic CAFs and tumor cells shape lymph node metastasis in oral squamous cell carcinoma
Source: PLoS Genet. 2025 Sep 4;21(9):e1011791. doi: 10.1371/journal.pgen.1011791 (PMC12410789; doi:10.1371/journal.pgen.1011791)

Supporting Figure 4

A HUH001–P1 (Primary tumor site, patient HUH001 with LNM)

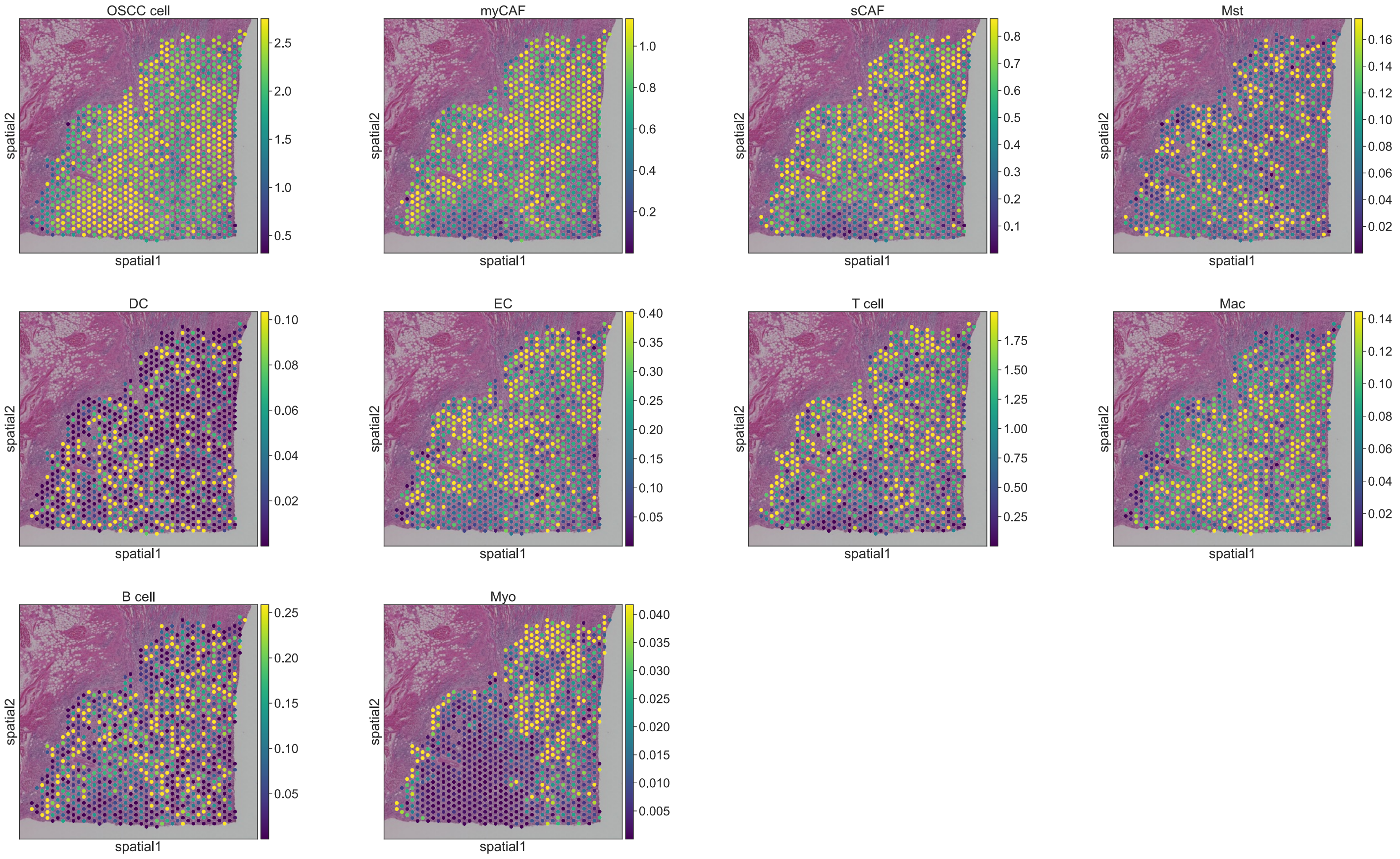

B HUH001–P2 (Primary tumor site, patient HUH001 with LNM)

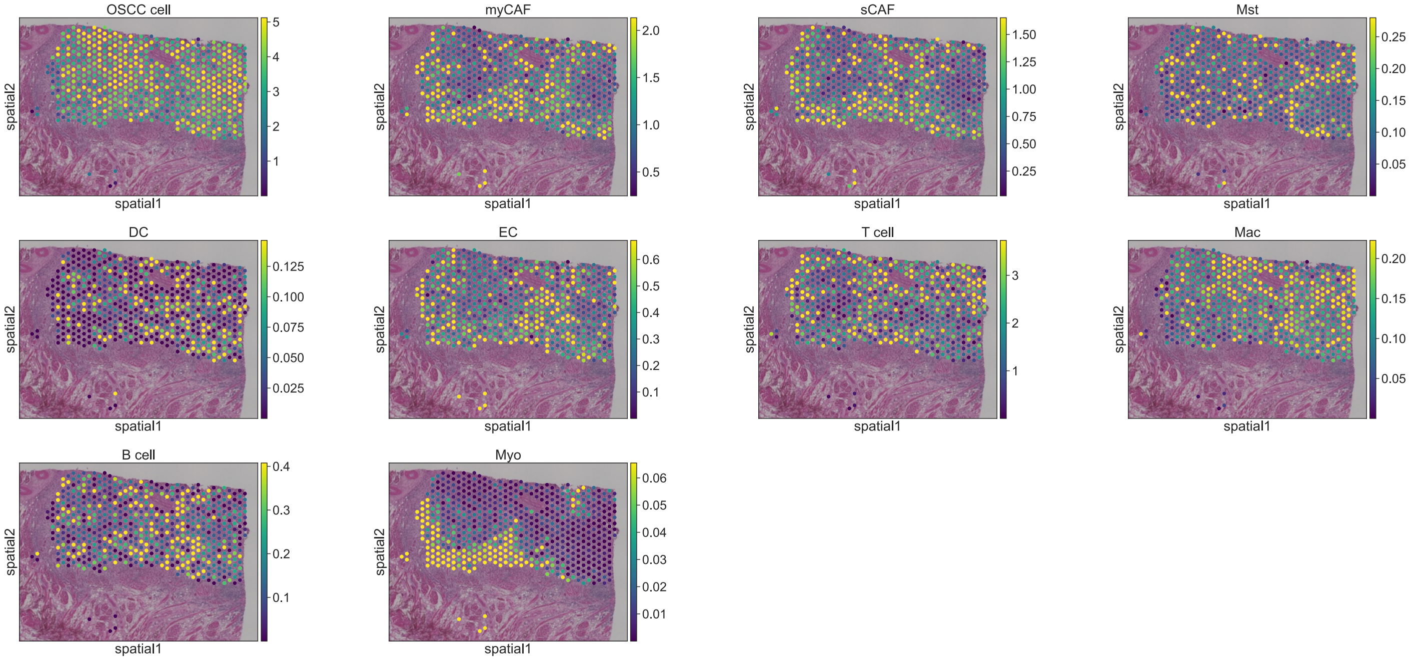

Supporting Figure 4 (continued)

C HUH001–met (Metastatic site, patient HUH001 with LNM)

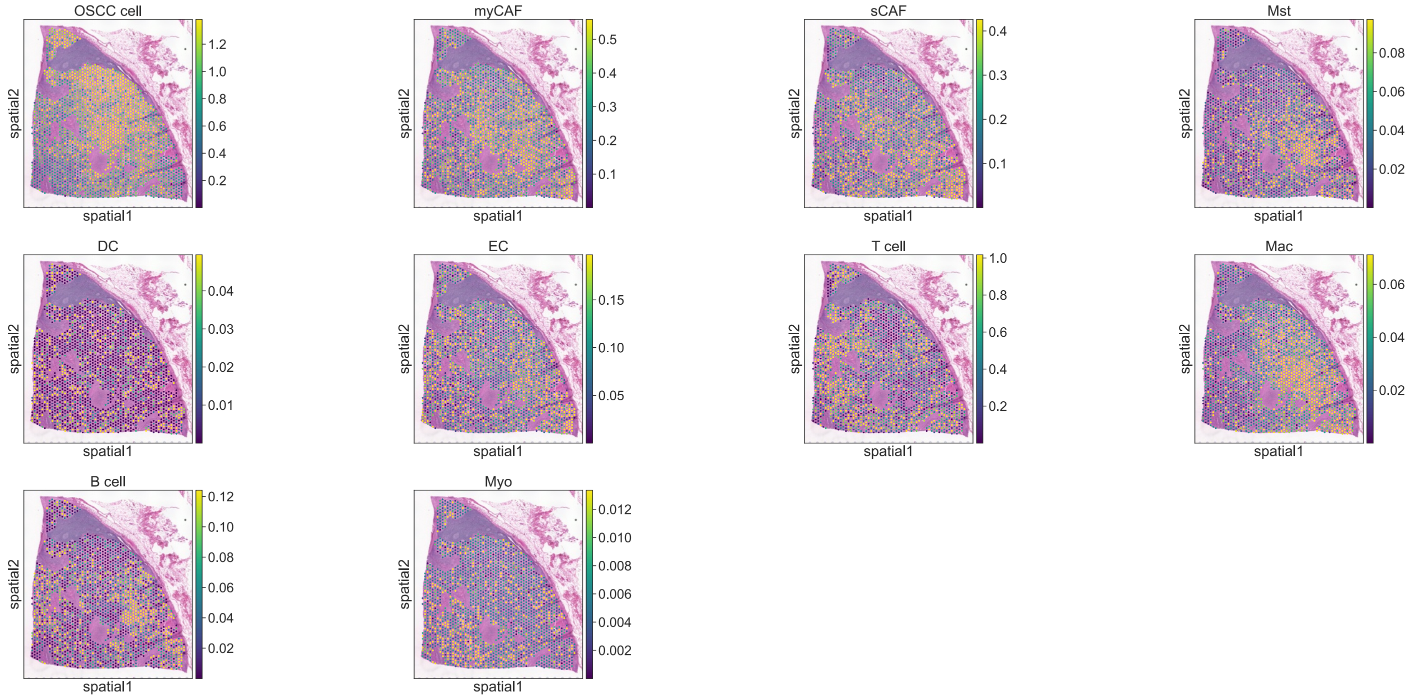

D HUH002–P (Primary tumor site, patient HUH002 without LNM)

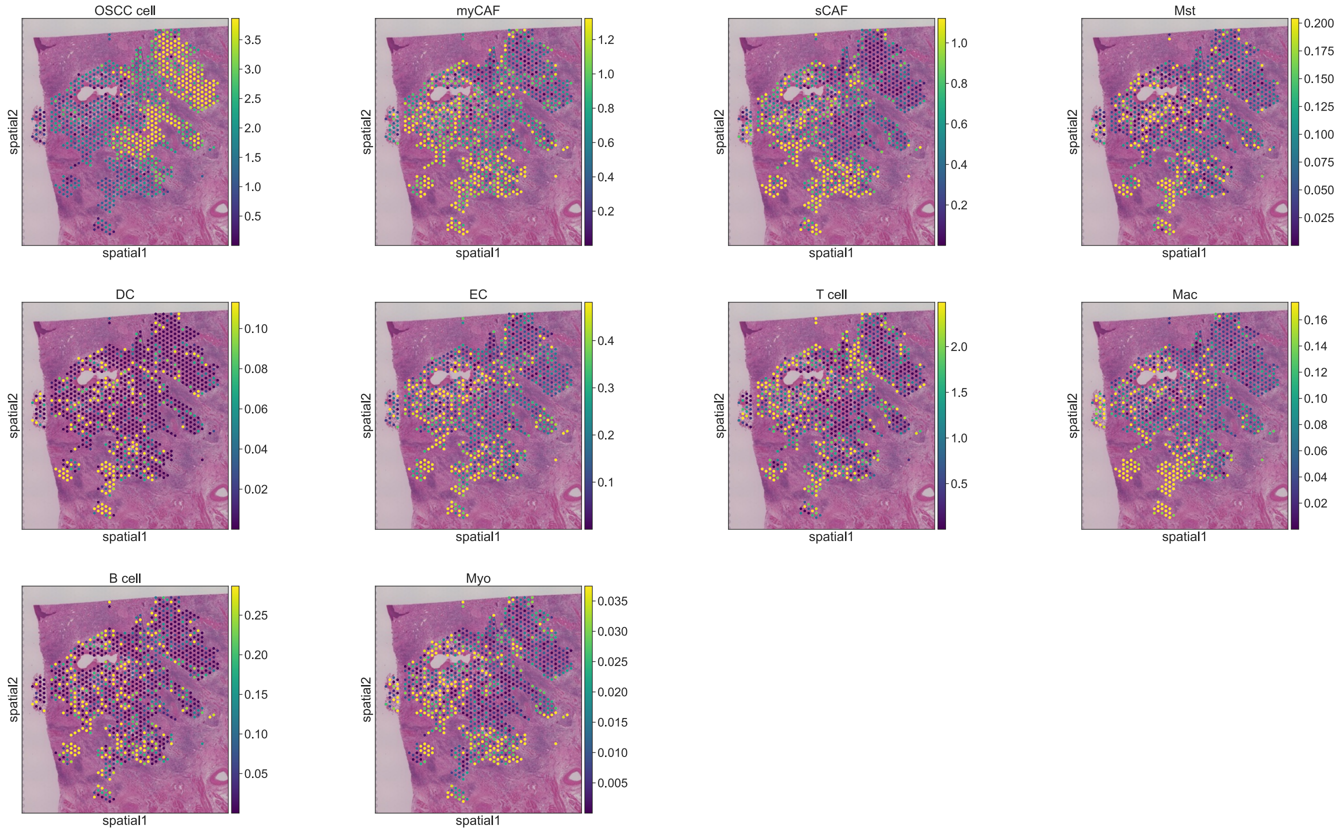

Supporting Figure 4 (continued)

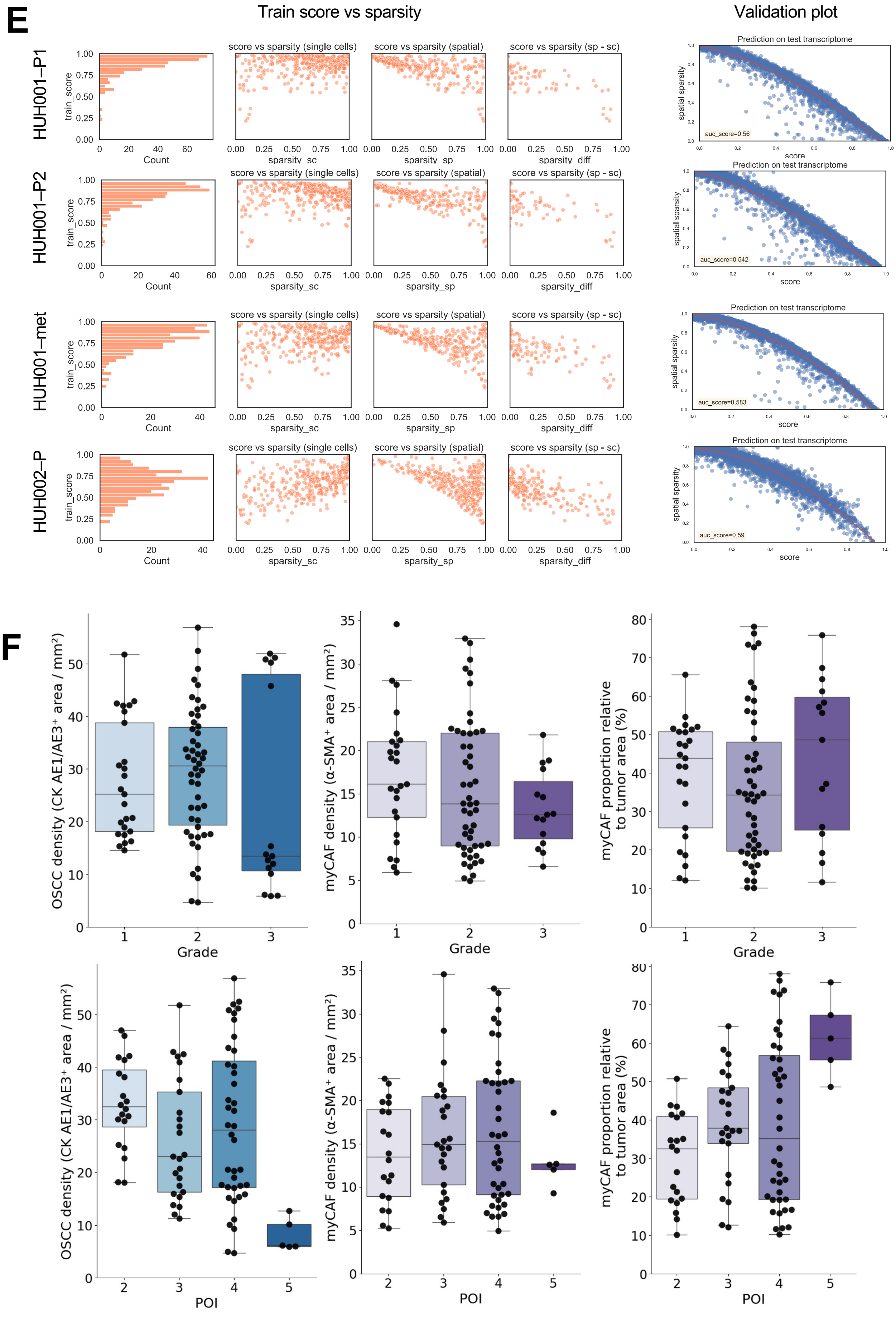

Supporting Figure 4 (continued)

G

HUH001–P1 (Primary tumor site, patient HUH001 with LNM)

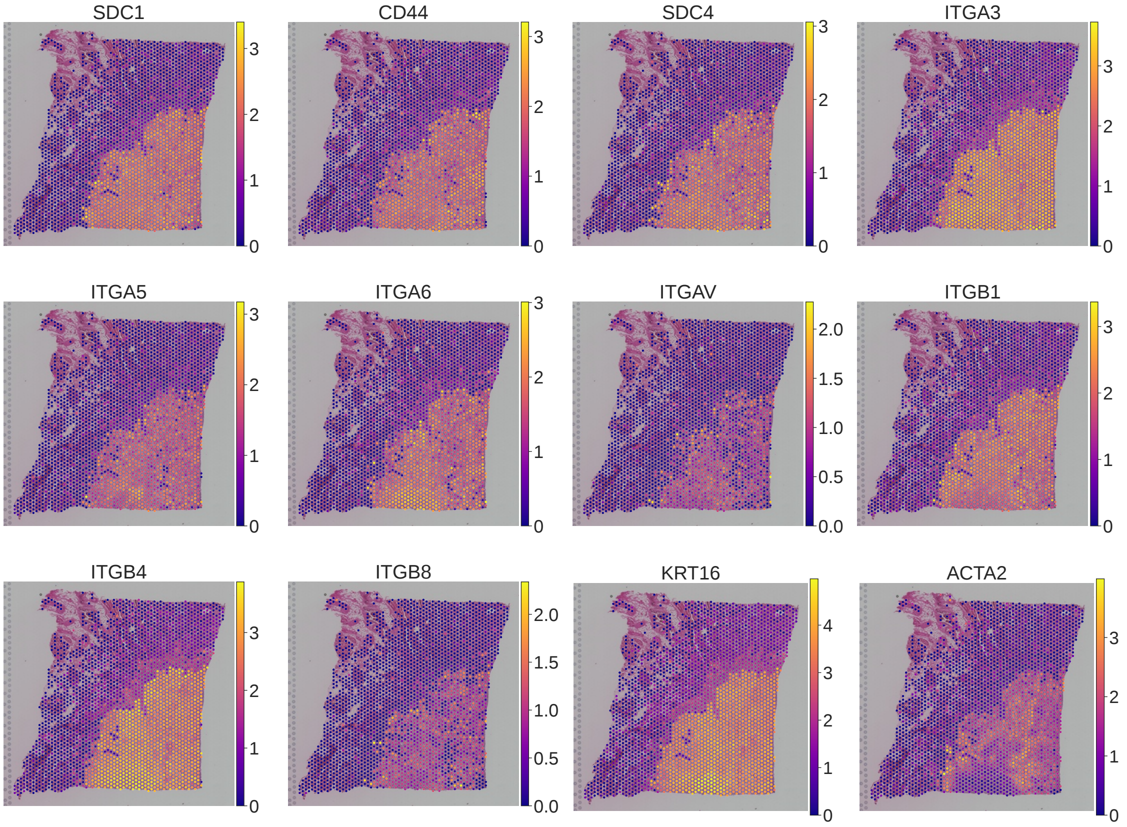

H

HUH001–P2 (Primary tumor site, patient HUH001 with LNM)

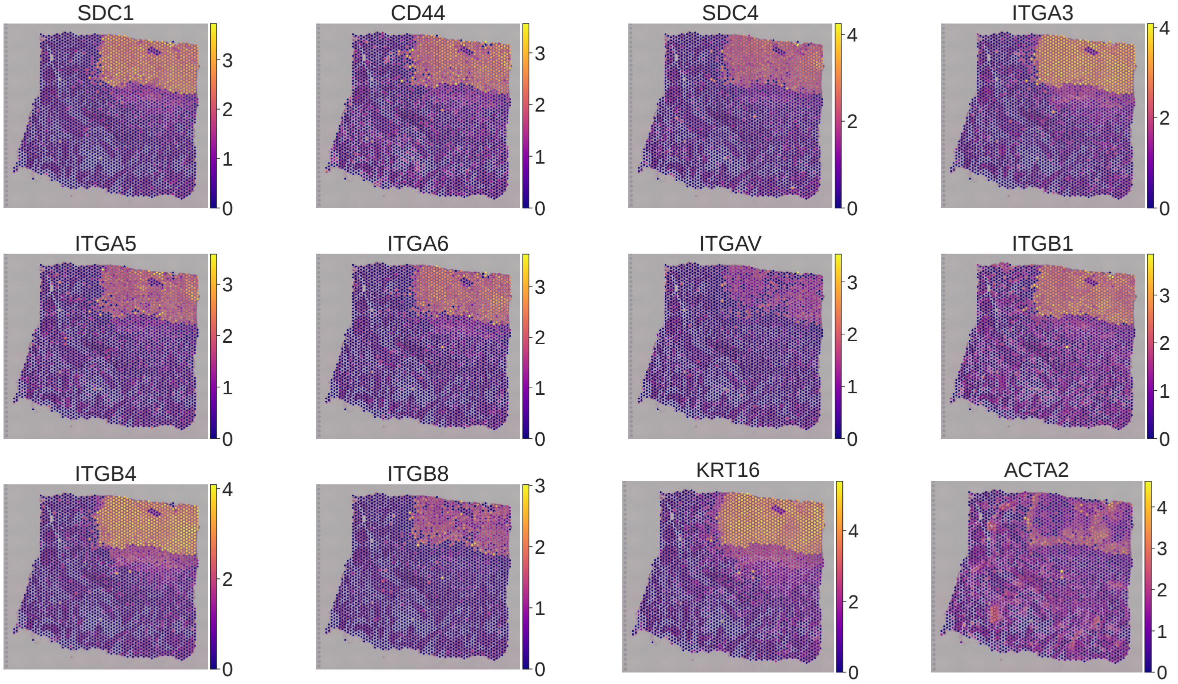

Supporting Figure 4 (continued)

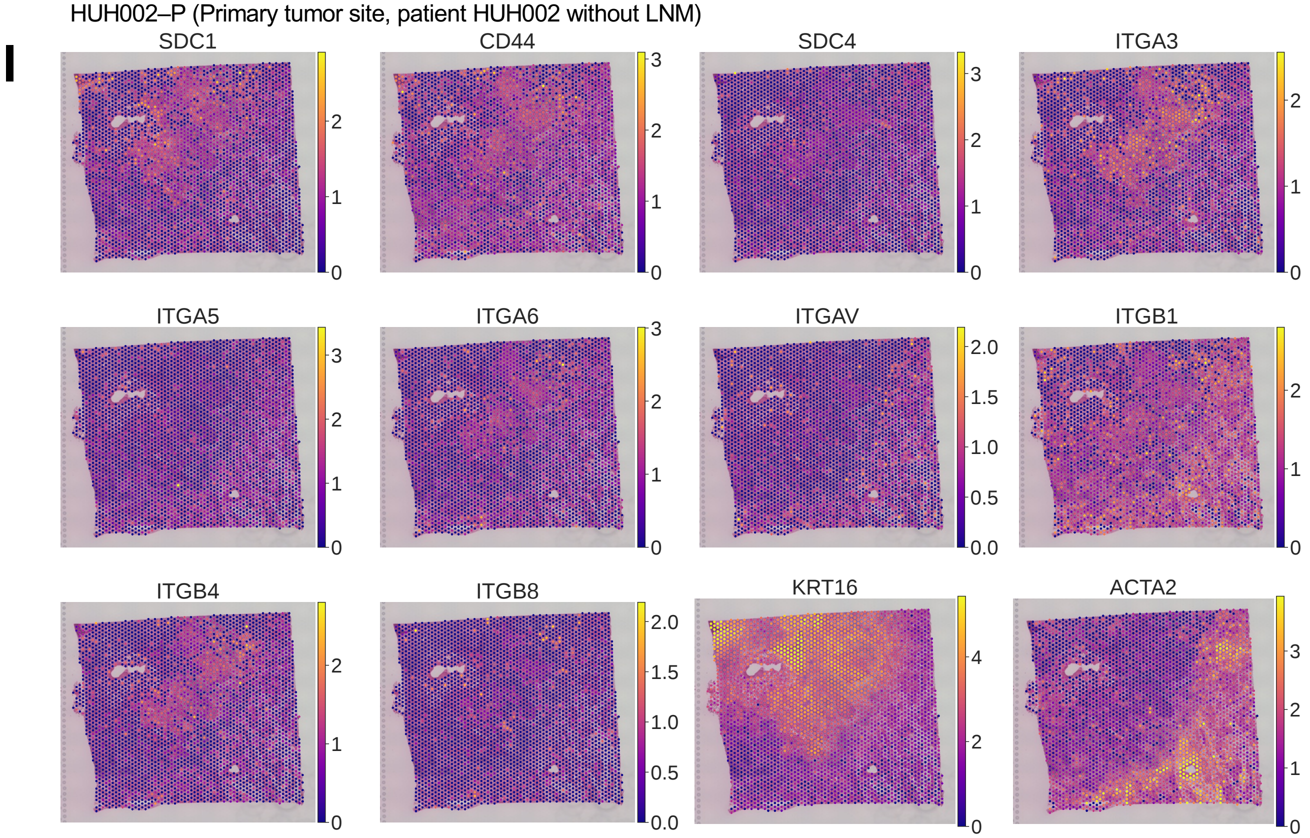

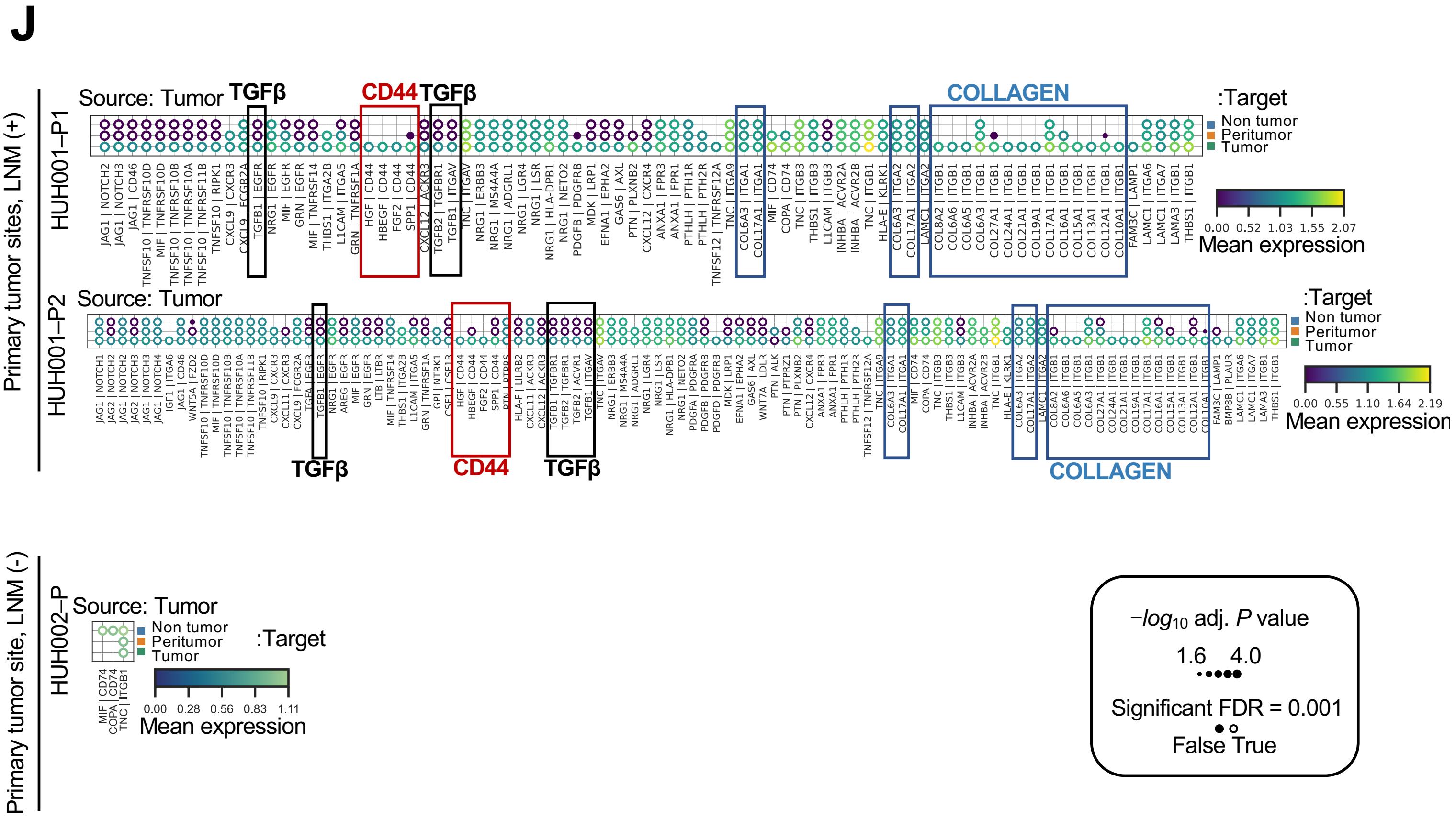

Supporting Figure 4 (continued)

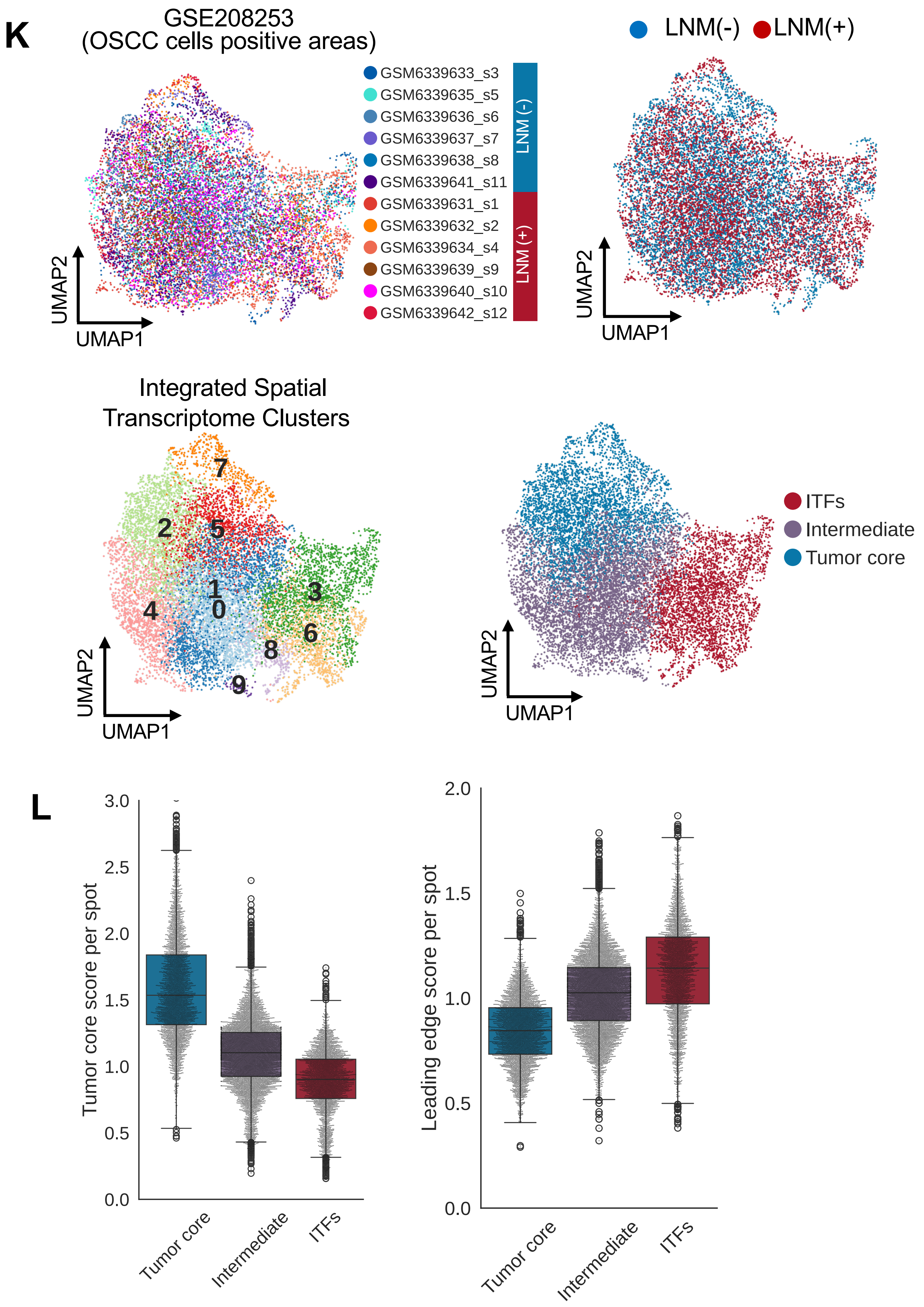

Supporting Figure 4 (continued)

M

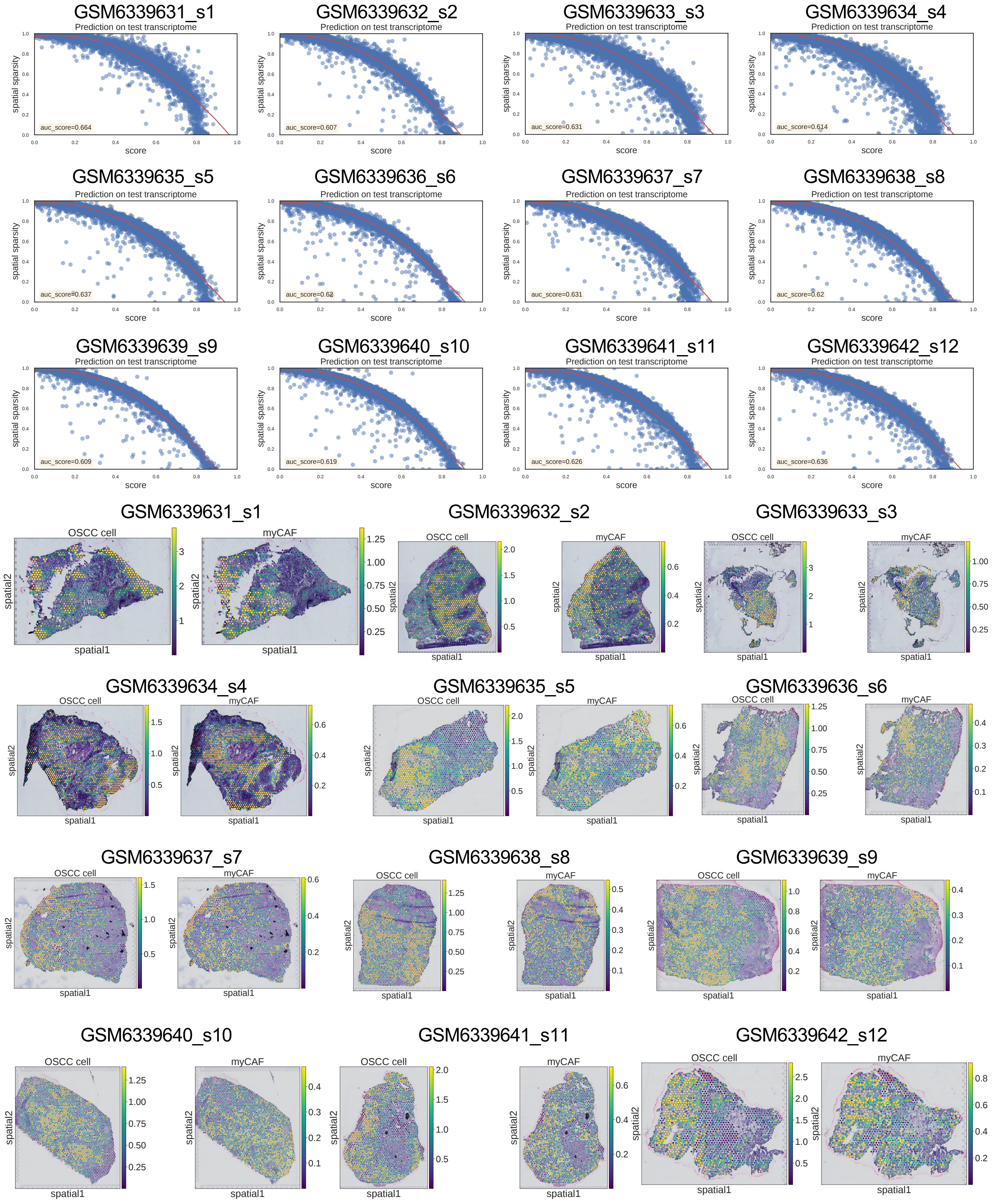

Supporting Figure 4 (continued)

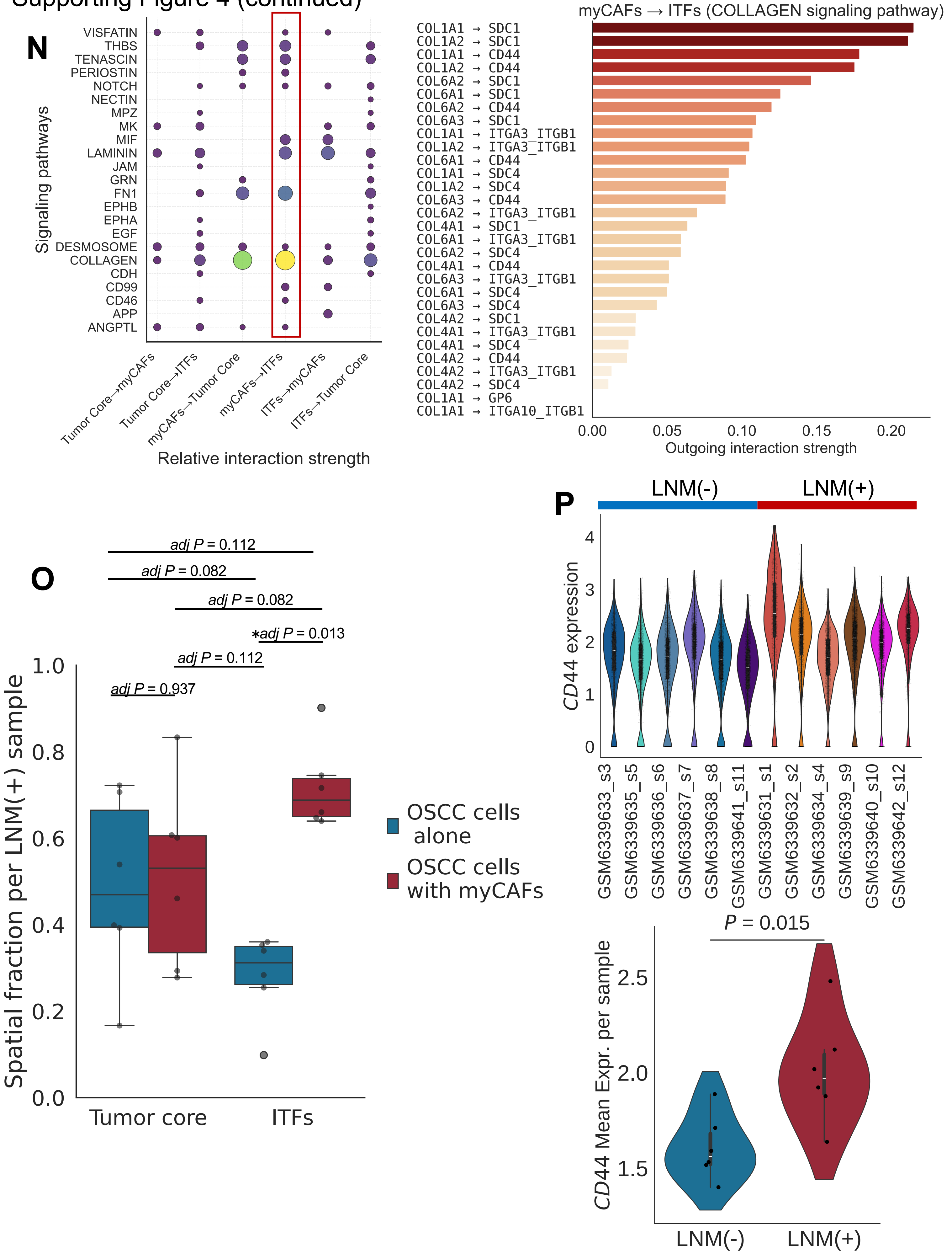

Supporting Figure 4 (continued)

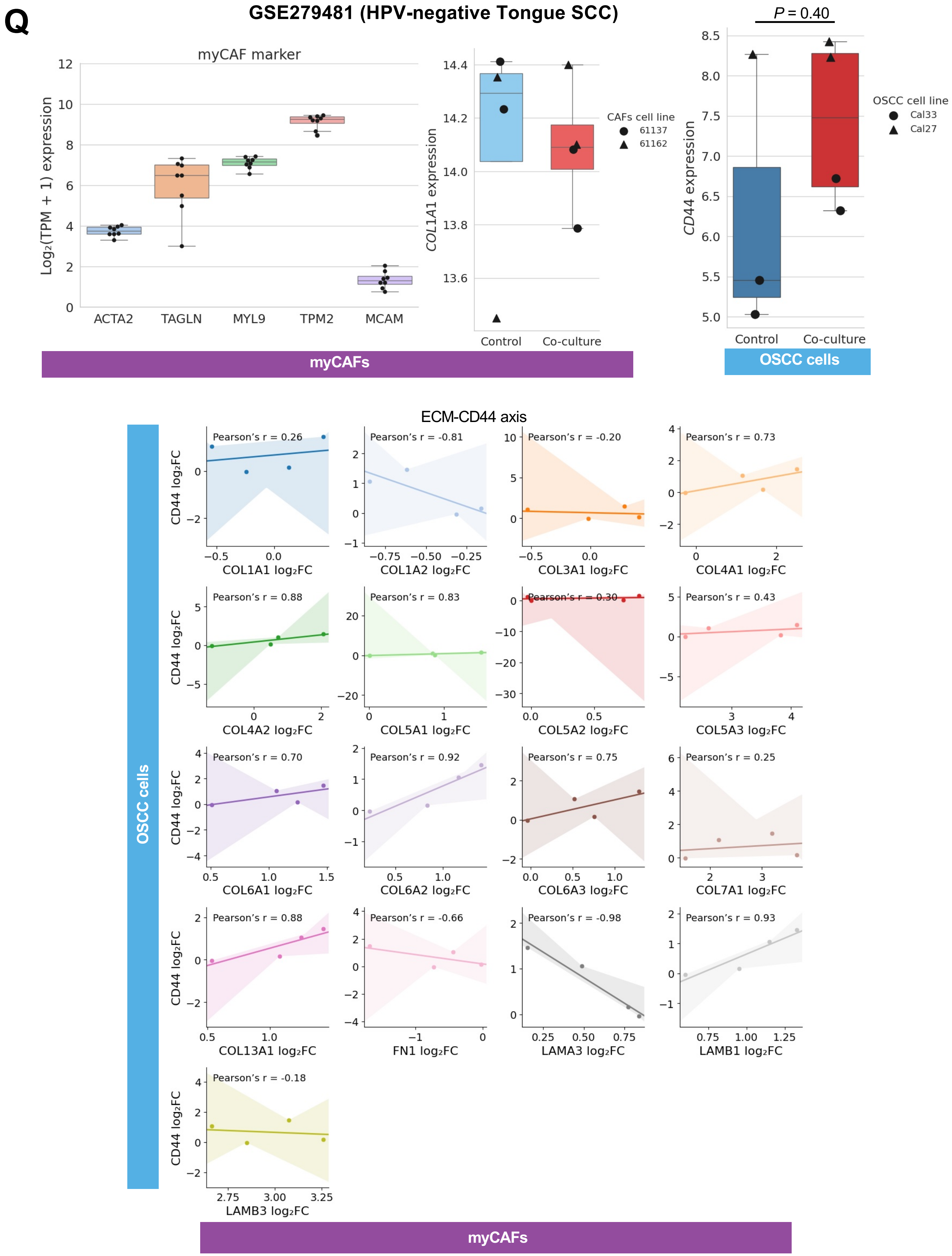

Supporting Figure 4 (continued)

R

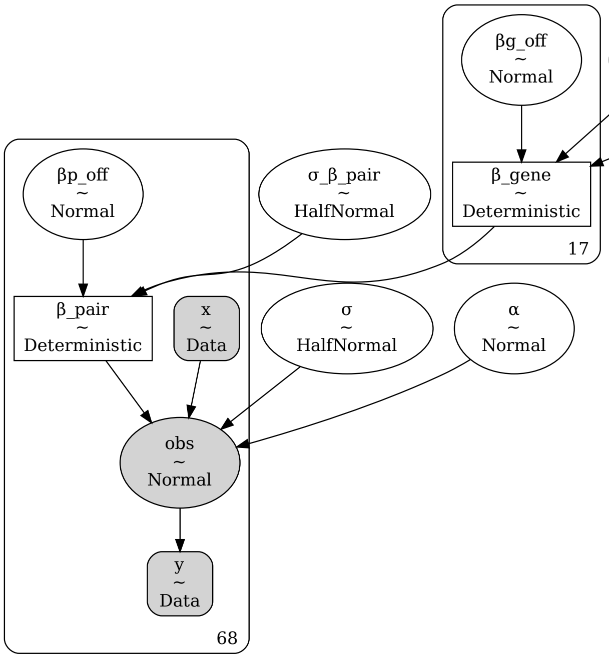

Symbol	Description
x	Standardized ECM gene expression (myCAFs)
y	Standardized *CD44* expression (OSCC cells)
μ_β	Population-level mean of slopes
σ_β_gene	SD of gene-level slope variation
β_gene	Gene-specific slope coefficient
σ_β_pair	SD of pair-specific slope variation
β_pair	Pair-specific slope coefficient
α	Intercept
σ	Residual SD
obs	Observed response variable

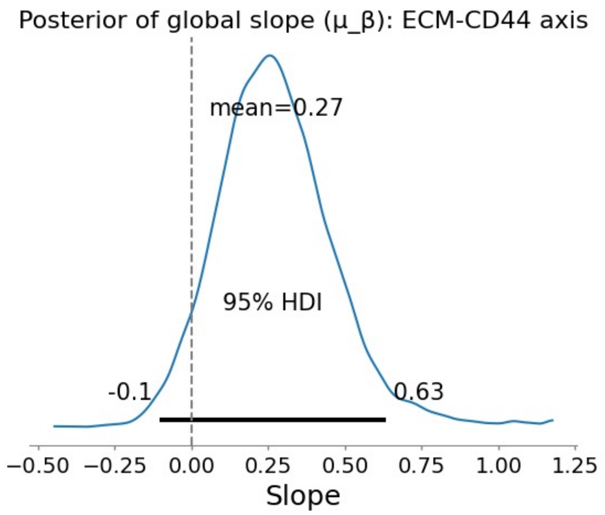

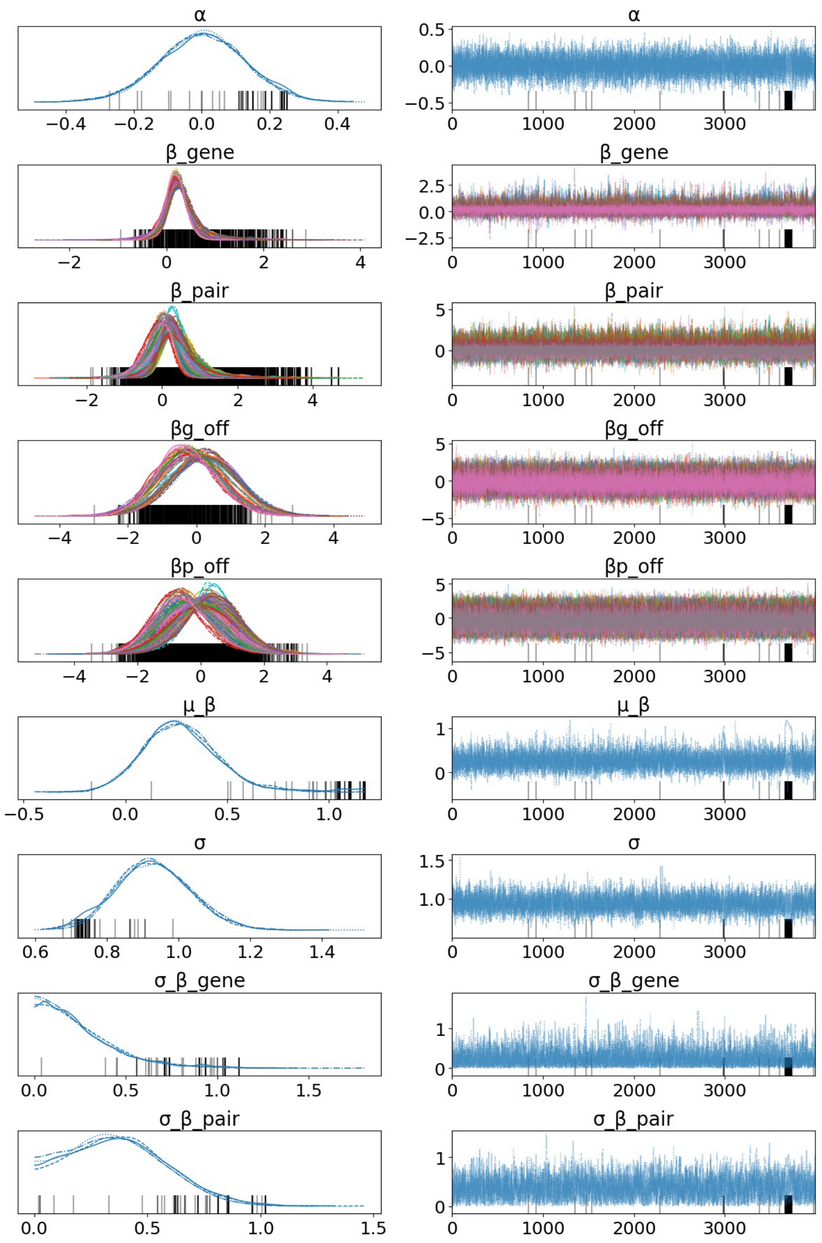

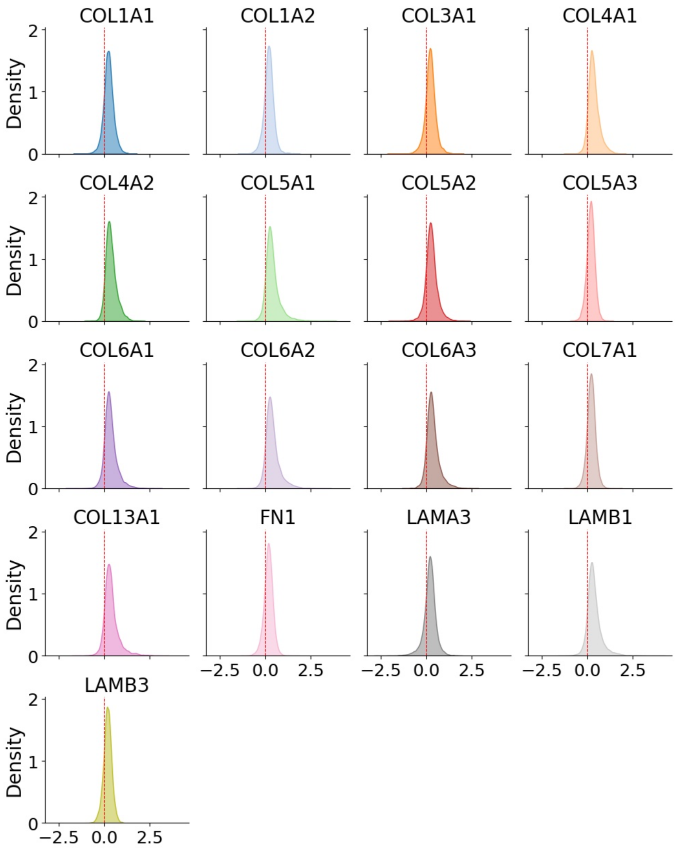

Supporting Figure 4 (continued)

R

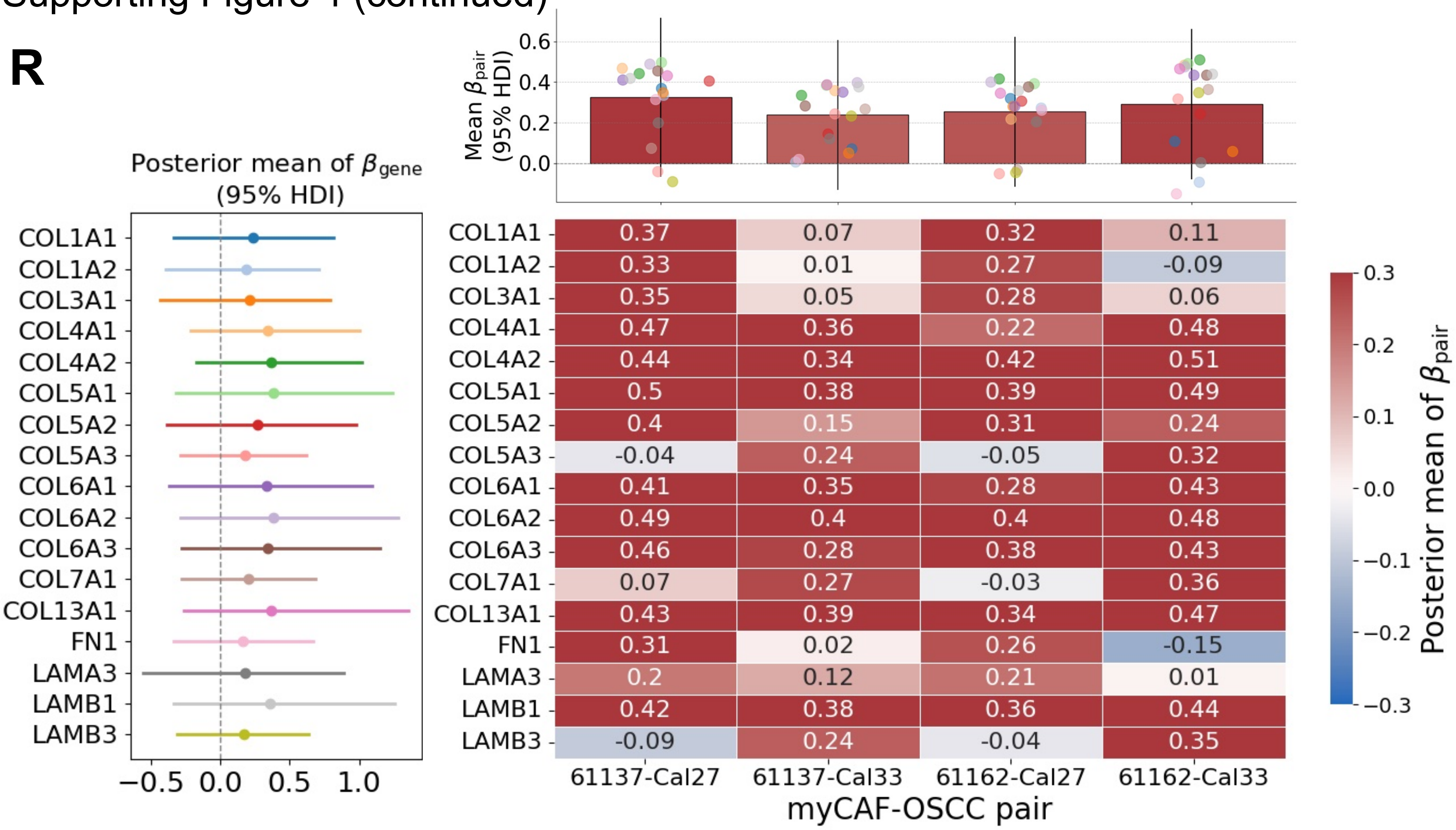

S

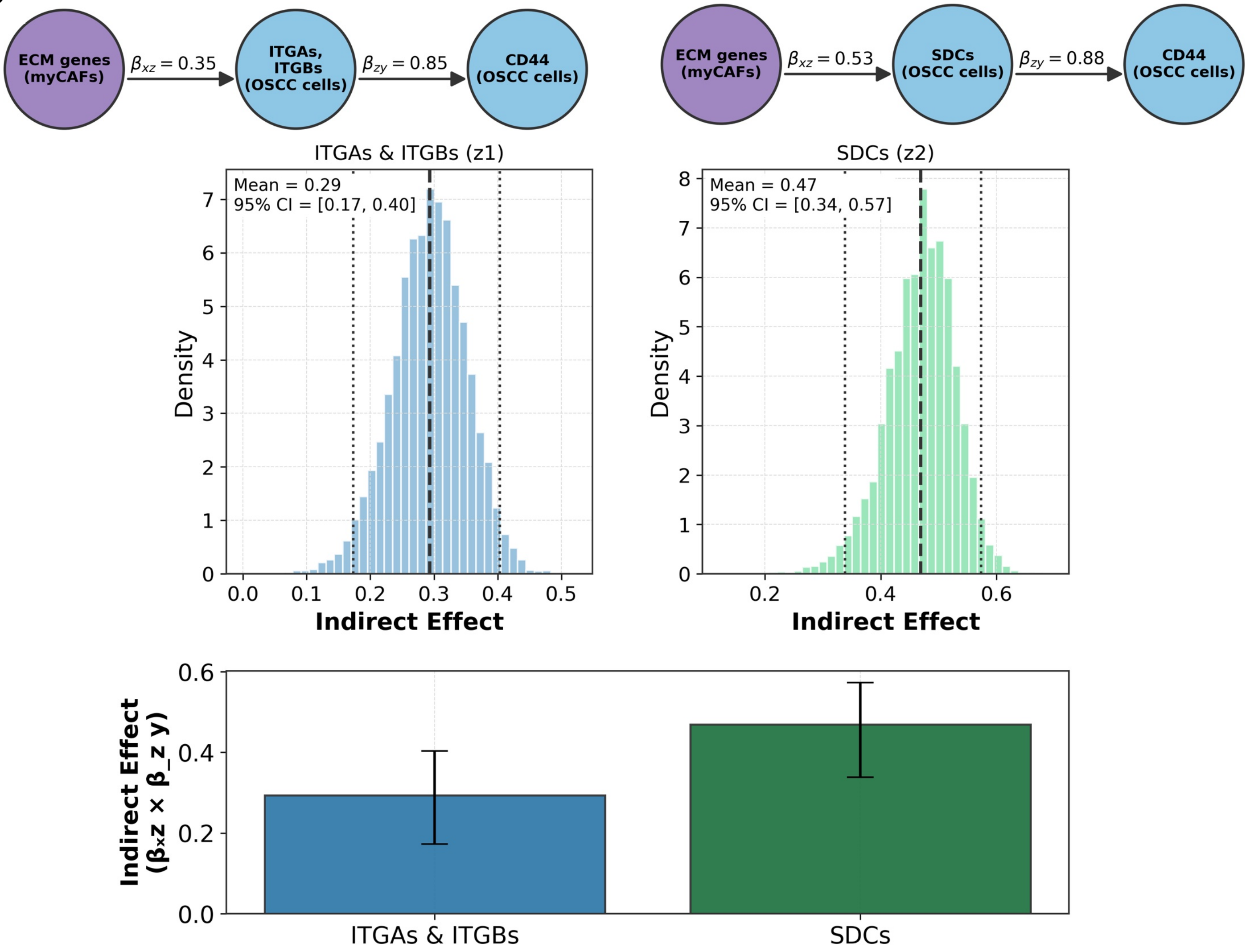

Supporting Figure 4 (continued)

T

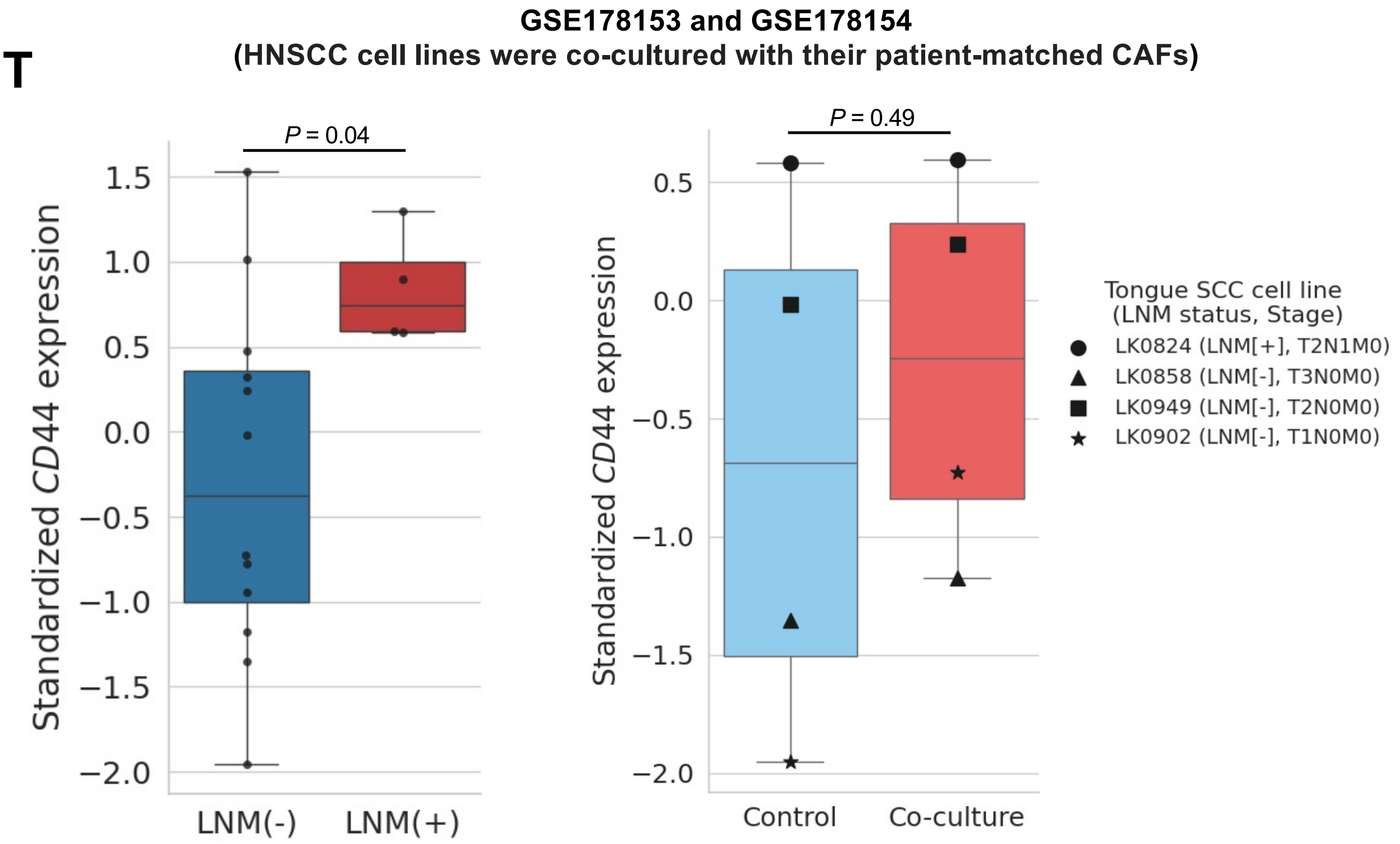

Supplement: S4 Fig — (A-D) Spatial localization at the single-cell level in the primary tumor tissues of patient HUH001 with LNM (HUH001-P1 and HUH001-P2) and of patient HUH002 without LNM (HUH002-P) was performed using spatial RNA-seq (spRNA-seq). The legends indicate the estimated cell numbers. Data for the oral squamous cell carcinoma (OSCC) cells and myofibroblastic cancer-associated fibroblasts (myCAFs) are presented in Fig 6A. (A) HUH001-P1, (B) HUH001-P2, (C) HUH001-met, and (D) HUH002-P. (E) Spatial deconvolution training score and validation plots. (F) Box plots comparing 3 different metrics in OSCC samples from ITF areas. The metrics are absolute OSCC density (CK AE1/AE3-positive area per square millimeter), absolute myCAF density (α-SMA-positive area per square millimeter), and relative myCAF proportion (α-SMA-positive area relative to the sum of α-SMA- and CK AE1/AE3-positive areas). In all box plots, center lines represent medians, boxes represent interquartile ranges (IQRs), and whiskers extend to ± 1.5 × IQRs. Dots represent individual ITF areas. Within this panel, box plots are grouped to show comparisons with the pathological differentiation grade (Grade 1, 2, and 3) in the upper section and with the pattern of invasion (POI) classification in the lower section [80]. Differences across grades and POI classifications for each metric were evaluated using the Kruskal–Wallis and Spearman correlations. (G-I) Spatial gene expression mapping of inferred receptor genes in OSCC cells via scRNAseq, including SDC1, CD44, SDC4, ITGA3, ITGA5, ITGA6, ITGAV, ITGB1, ITGB4, ITGB8, KRT16, and ACTA2. The color-coded regions represent the expression levels of these genes. (G) HUH001-P1, (H) HUH001-P2, and (I) HUH002-P. (J) Ligand-receptor interaction analysis for HUH001-P1, HUH001-P2, and HUH002-P shows tumor-to-tumor, peritumor, and nontumor regions, highlighting a subset of significant ligand-receptor pairs. Visualization filters were applied to receptor pairs (FDR < 0.001; means_range > [file pgen.1011791.s020.pdf]
