# Supplementary material for: Spatial colocalization and molecular crosstalk of myofibroblastic CAFs and tumor cells shape lymph node metastasis in oral squamous cell carcinoma
Source: PLoS Genet. 2025 Sep 4;21(9):e1011791. doi: 10.1371/journal.pgen.1011791 (PMC12410789; doi:10.1371/journal.pgen.1011791)

Supporting Figure 5

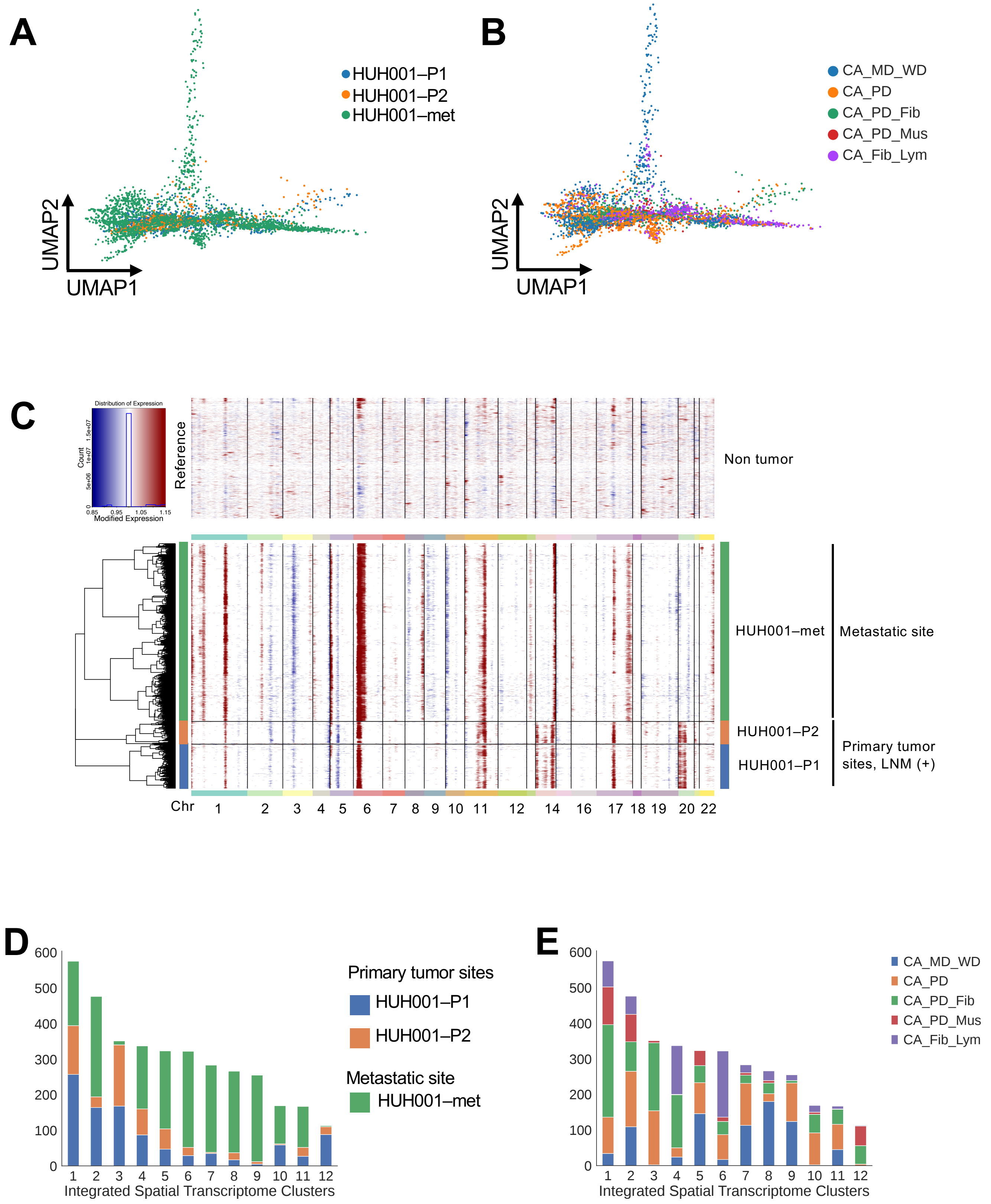

Supporting Figure 5 (continued)

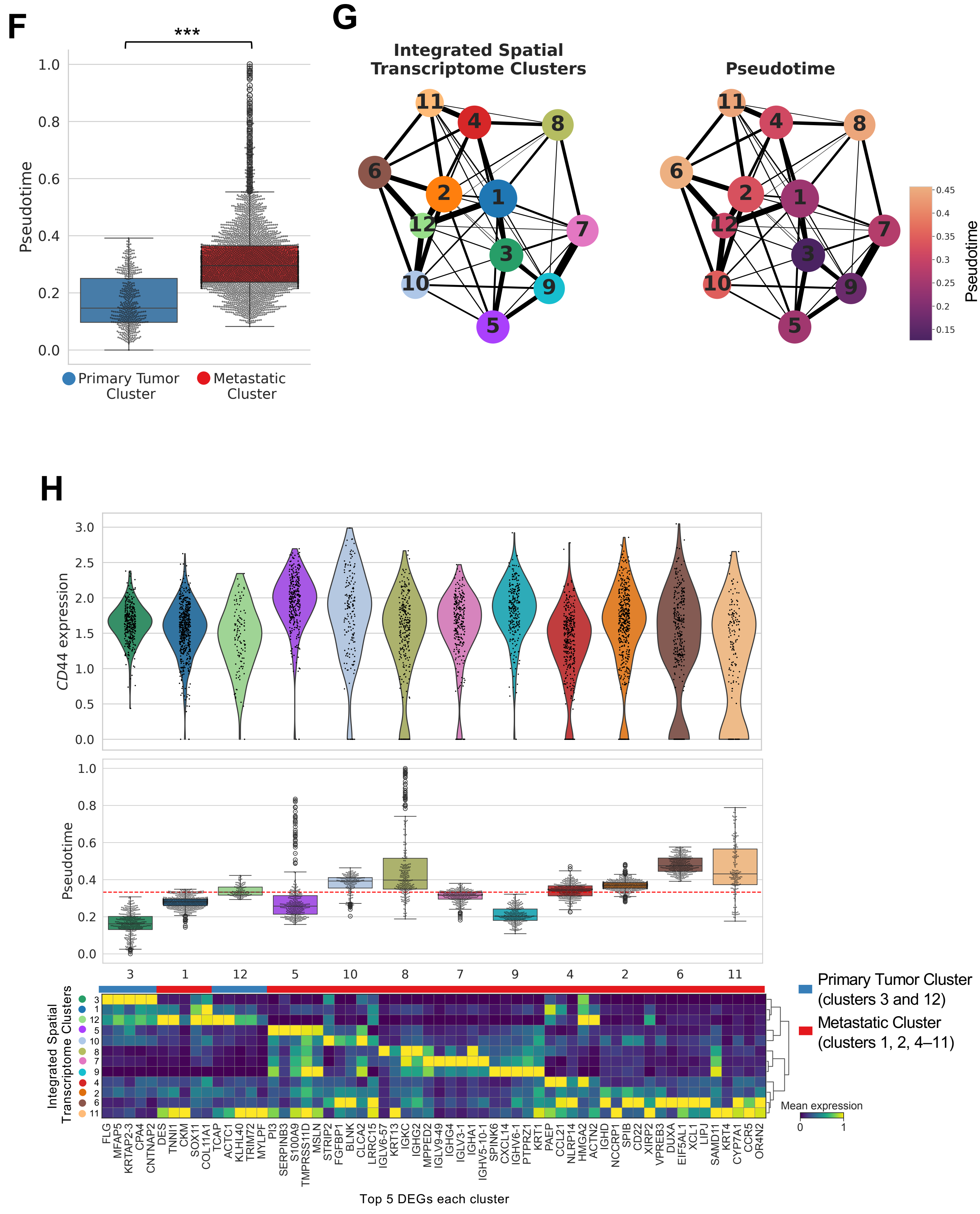

Supporting Figure 5 (continued)

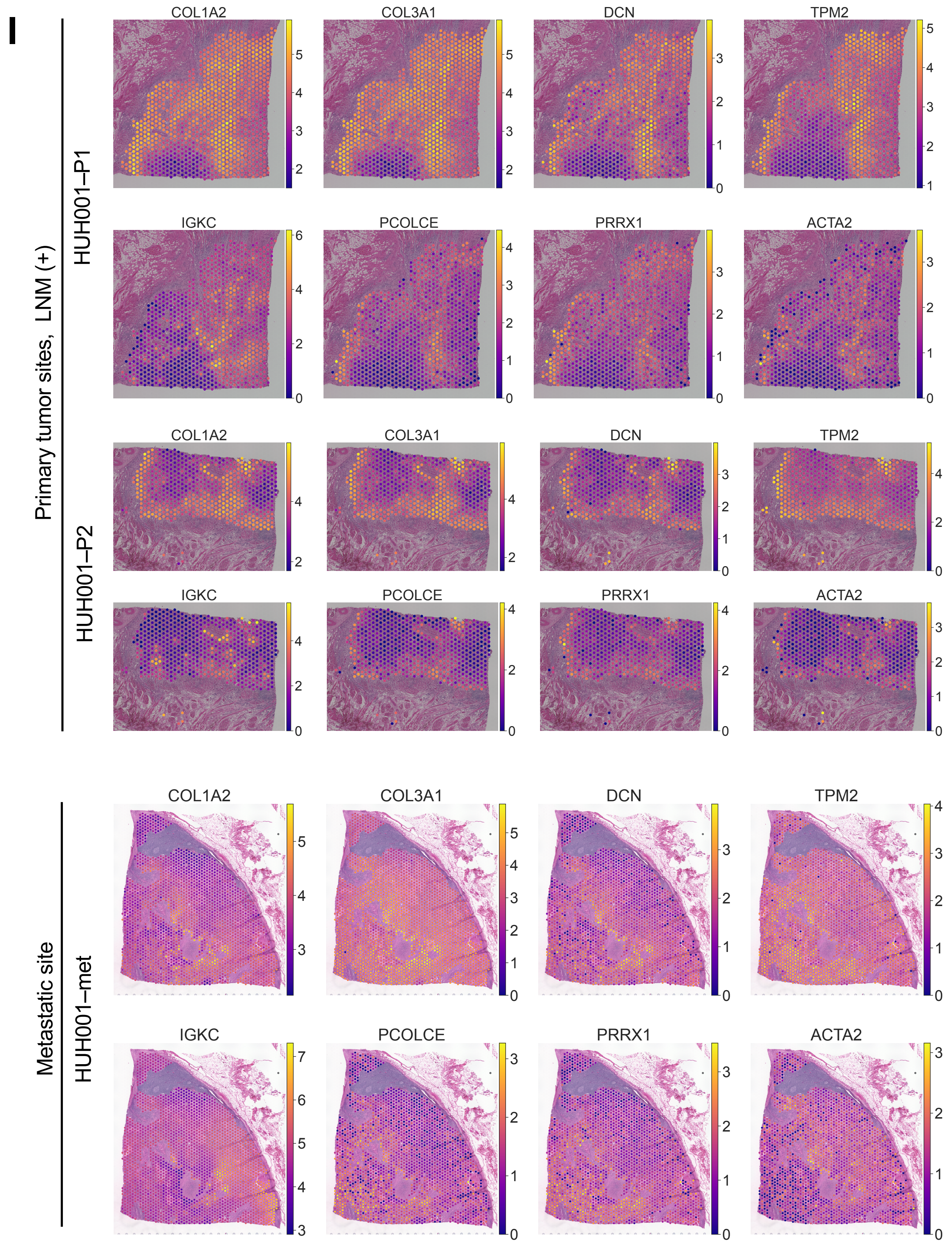

Supporting Figure 5 (continued)

J

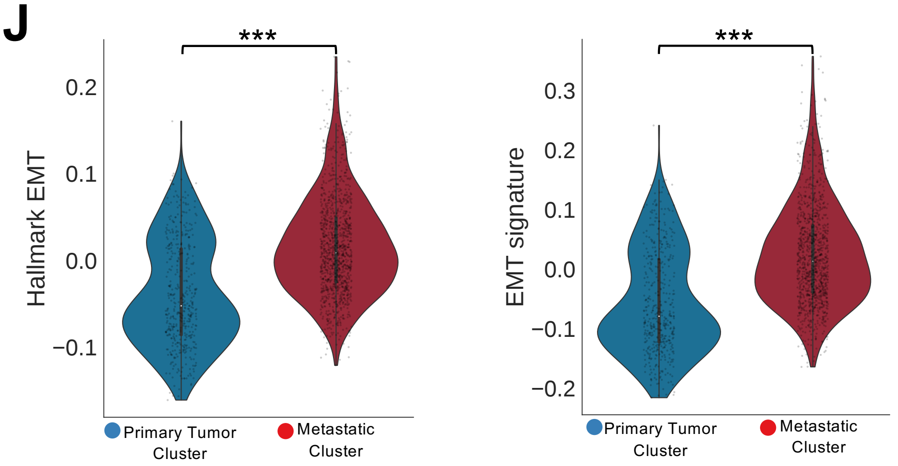

K

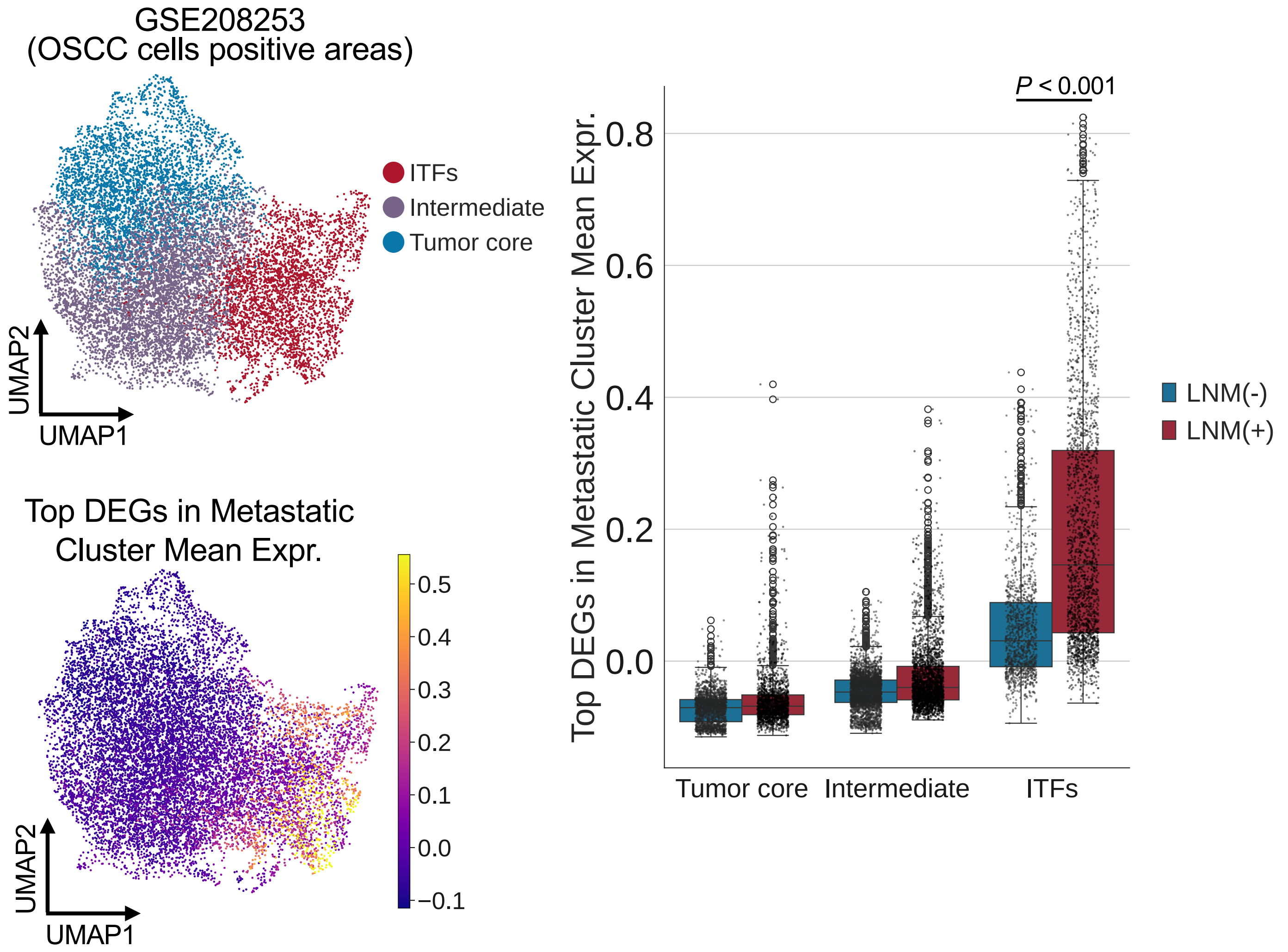

Supplement: S5 Fig — (A and B) Uniform manifold approximation and projection (UMAP) visualization demonstrating the integrated spatial transcriptome data from 2 primary tumor sites (HUH001-P1 and HUH001-P2) and 1 metastatic site (HUH001-met) from patient HUH001, who had lymph node metastasis (LNM). Labels on the left represent samples; annotations on the right (CA_MD_WD, cancer, moderately differentiated/well-differentiated CA_PD, cancer, poorly differentiated; CA_PD_Fib, cancer, poorly differentiated fibrosis; CA_PD_Mus, cancer, poorly differentiated muscle; and CA_Fib_Lym, cancer, fibrosis, lymphocytes) pertain to histopathology. (C) Copy number variations within the integrated spatial transcriptome in 2 primary tumor sites (HUH001-P1 and HUH001-P2) and 1 metastatic site (HUH001-met) from patient HUH001 with LNM. (D and E) Stacked bar graphs show the sample distributions in spatial transcriptome clusters, with (D) HUH001-P1 in blue, HUH001-P2 in orange, and HUH001-met in green. (E) Histopathological annotations within the clusters. (F) Metastatic tumor regions exhibited significantly longer pseudotimes in the spatial trajectory analysis, suggesting an advanced developmental state. Box plots compare stLearn-derived pseudotime values between aggregated Primary Tumor Clusters (blue) and Metastatic Clusters (red) from the spatial transcriptomics data (median pseudotimes: metastatic clusters = 0.34 [95% bootstrap CI, 0.33-0.34] vs. primary tumor clusters = 0.19 [95% bootstrap CI, 0.17-0.20]). Black circles represent individual spots. The center lines represent the medians, the box borders represent the interquartile ranges (IQRs), and the whiskers represent ± 1.5 × IQRs. Statistical significance was assessed using a 2-sided Mann-Whitney U test: ***P < 0.001. (G) Spatial trajectory inference reveals potential progression paths between integrated saptial transcriptome clusters using partition-based graph abstraction (PAGA) and diffusion pseudotime (DPT). Left panel: PAGA graph illustrating t [file pgen.1011791.s021.pdf]
