# Supplementary material for: Spatial colocalization and molecular crosstalk of myofibroblastic CAFs and tumor cells shape lymph node metastasis in oral squamous cell carcinoma
Source: PLoS Genet. 2025 Sep 4;21(9):e1011791. doi: 10.1371/journal.pgen.1011791 (PMC12410789; doi:10.1371/journal.pgen.1011791)

Supporting Figure 6

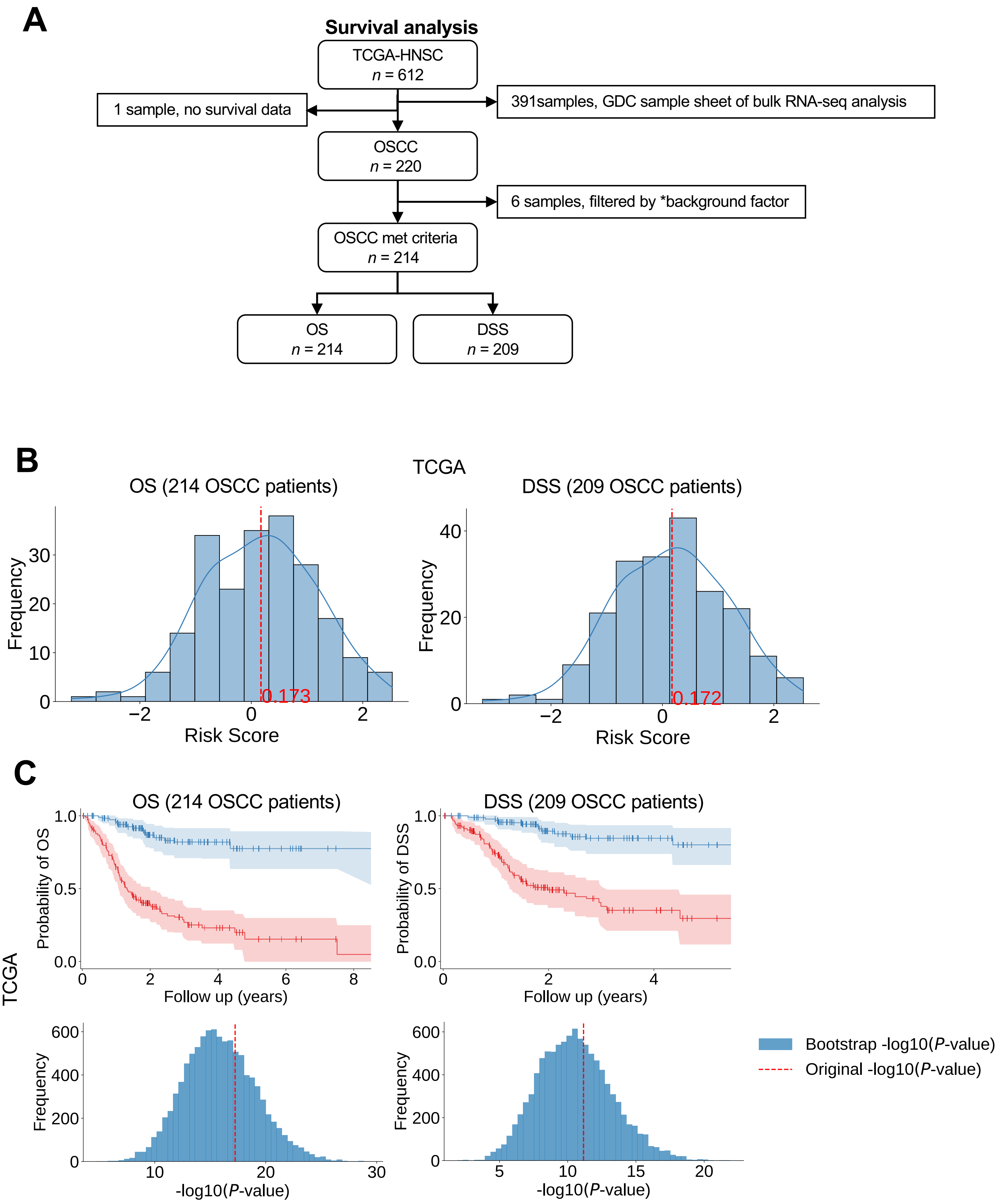

Supporting Figure 6 (continued)

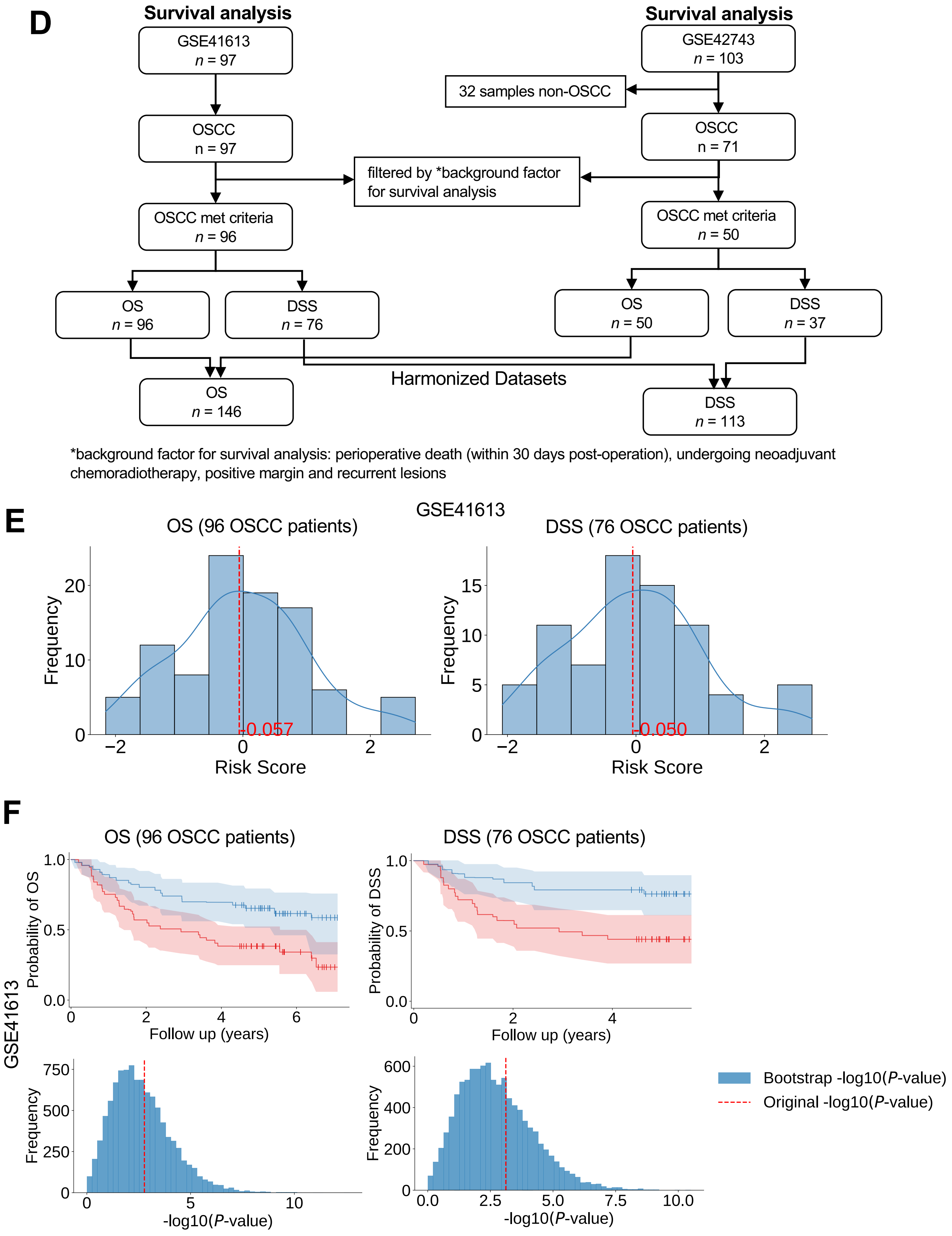

Supporting Figure 6 (continued)

G

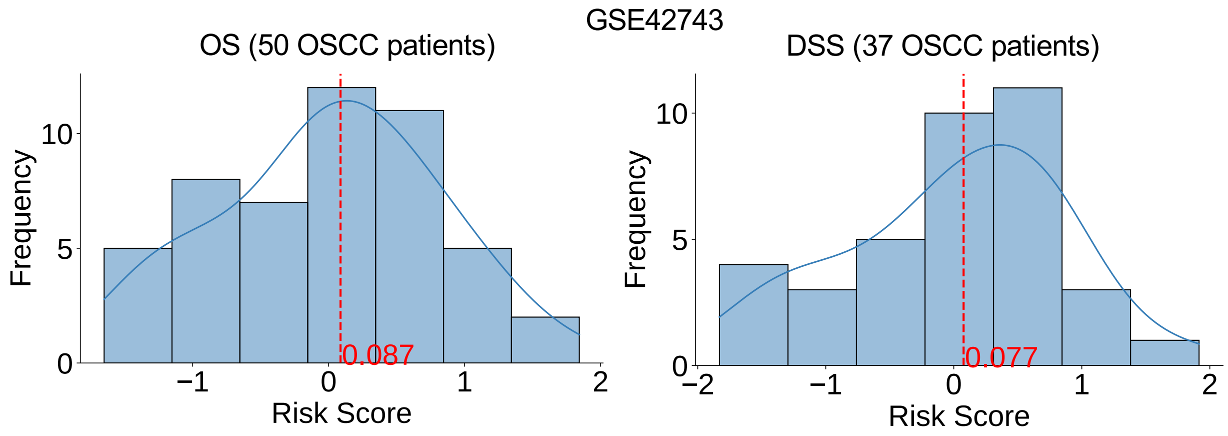

H

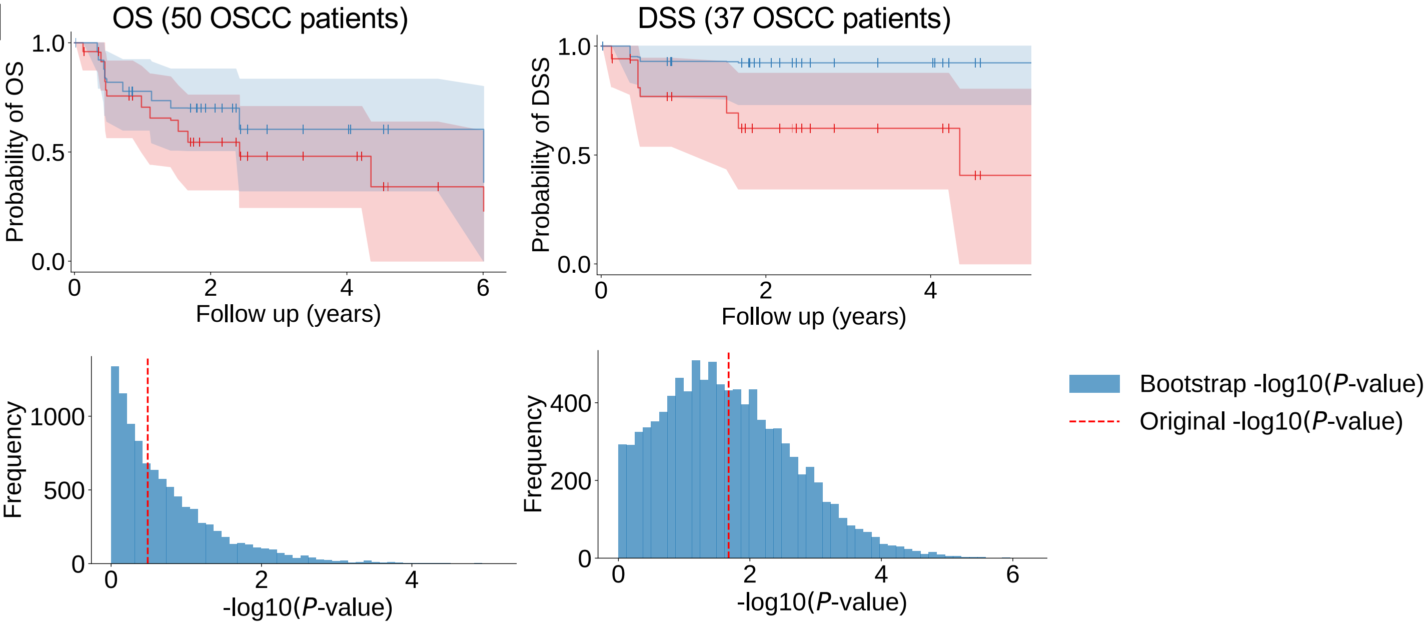

Supporting Figure 6 (continued)

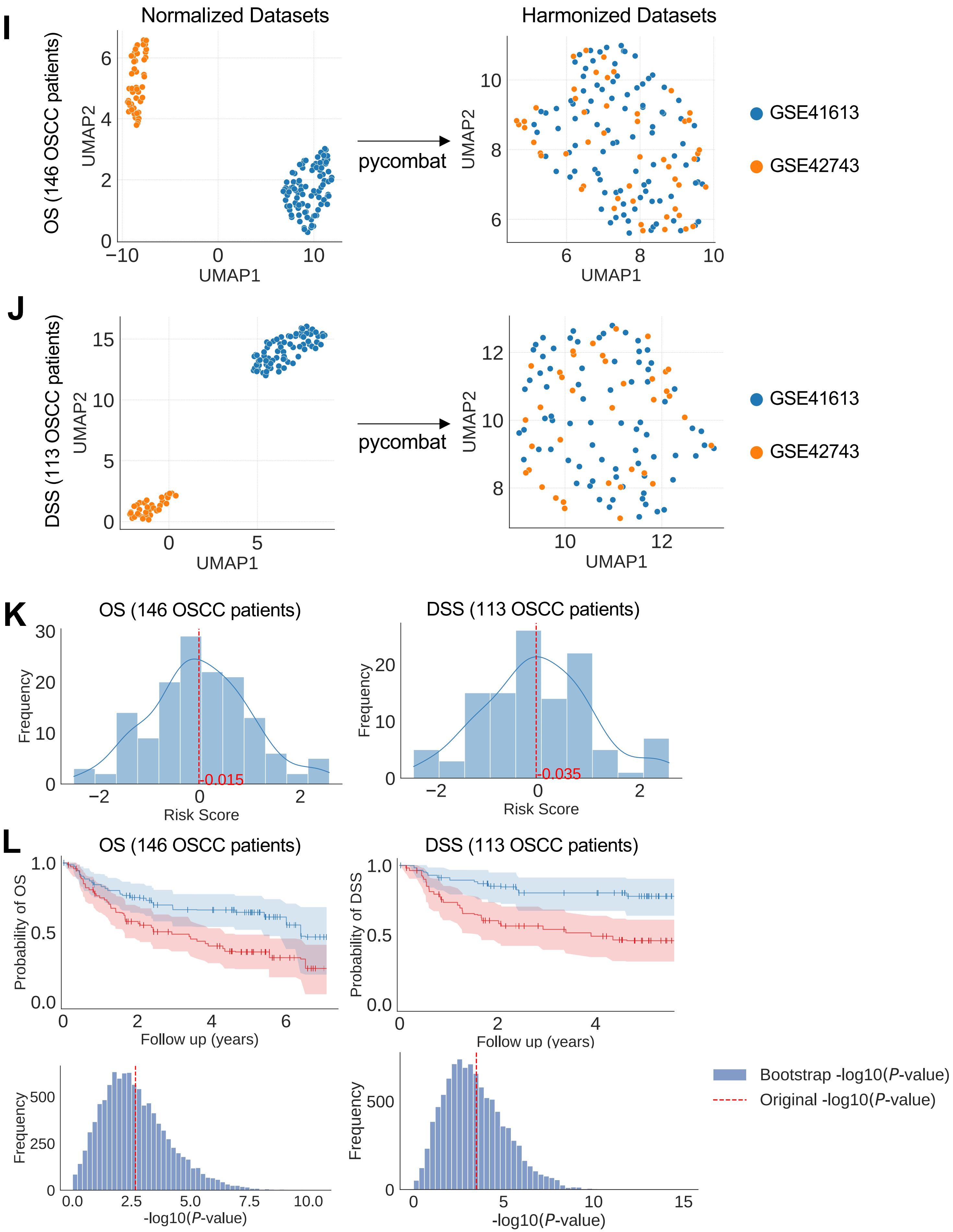

Supplement: S6 Fig — (A) Overview of patients used in the survival analysis using The Cancer Genome Atlas (TCGA) data. (B) Bar charts show the distribution of TCGA training group risk scores. Median scores are indicated for 214 patients for overall survival (OS) and 209 patients for disease-specific survival (DSS), respectively. (C) Kaplan-Meier curves, averaged using 10,000 bootstrap resamples with 95% CI, display OS and DSS on the basis of the risk scores from 23 signature genes. Patients were grouped as having high (blue) or low (red) risk scores on the basis of the median scores (OS for 214 TCGA patients and DSS for 209 TCGA patients). The P-value distributions from the log-rank test for the bootstrap samples are represented by blue bars, and the P-values from the log-rank test of the original data are depicted using red dotted lines. Refer to 9B and 9C. (D) Overview of the cohorts from the microarray datasets GSE41613 and GSE42743, which were used for the validation analysis. (E-H) Bar charts depict the risk score distributions in the GSE41613 and GSE42743 datasets. Median scores are highlighted. Kaplan-Meier curves, produced from 10,000 bootstrap resamples with a 95% CI, display OS and DSS outcomes based on these scores. Patients were grouped as having high (blue) or low (red) risk scores on the basis of the median scores. The P-value distributions from the log-rank test for the bootstrap samples are represented by blue bars, and the P-values from the log-rank test of the original data are depicted using red dotted lines. (E, F) For GSE41613: the OS was determined using 96 patients, and the DSS was determined using 76 patients. For details using the original data, refer to Fig 9H and 9I. (G, H) For GSE42743: the OS was determined using 50 patients, and the DSS was determined using 37 patients. For details using the original data, refer to Fig 9J and 9K. (I, J) Uniform manifold approximation and projection (UMAP) visualizations from the 2 harmonized microarray datasets (GSE41613 an [file pgen.1011791.s022.pdf]
