# Supplementary material for: Spatial colocalization and molecular crosstalk of myofibroblastic CAFs and tumor cells shape lymph node metastasis in oral squamous cell carcinoma
Source: PLoS Genet. 2025 Sep 4;21(9):e1011791. doi: 10.1371/journal.pgen.1011791 (PMC12410789; doi:10.1371/journal.pgen.1011791)
Supplement: Attachment — Submitted filename: Response to reviewers(reduce size).pdf [file pgen.1011791.s024.pdf]

We would like to first thank the reviewers for their critical assessment of our manuscript and their constructive feedback. We are most grateful for the comments, which we believe have helped us improve the manuscript considerably. Our point-by-point response is outlined below. We thank you in advance for your attention and hope you will find our manuscript suitable for publication in *PLOS Genetics.*

Reviewers' comments for PGENETICS-D-25-00169:

Reviewer's Responses to Questions

Reviewer #1: This study provides a comprehensive investigation into the role of myCAFs in promoting lymph node metastasis (LNM) in OSCC. While the findings are compelling, several issues require clarification or expansion:

RE: We greatly appreciate the reviewer's positive evaluation and valuable suggestion.

1.In spatial transcriptomic analysis part, can the author supply the clear HE results (without any label) of every sample to clarify the location of tumor cells and myCAFs? Because in the HUH002-P, the location of OSCC cells seems not very accurate based on the picture (left).

RE: Thank you for raising this question. We believe the apparent discrepancy the reviewer observed, particularly in the HUH002-P visualization compared to your reference diagnostic images, stems primarily from the use of adjacent sections prepared from different FFPE blocks. As detailed in the Methods ("FFPE sample preparation and hematoxylin and eosin—stained images for spatial transcriptome analysis"), the Visium platform requires specific tissue dimensions (a 6.5 mm × 6.5 mm or smaller tissue section). Therefore, for each surgical specimen, we prepared two adjacent FFPE blocks: one for routine diagnostic purposes (including H&E and IHC, such as **S3A and S3B**) and one specifically dedicated to Visium analysis. While these adjacent blocks capture the same overall tissue area, subtle differences in cellular morphology and distribution between sections originating from separate blocks are expected. To clarity this, we have added a figure legend to **Supporting Figure s S3A and S3B** (**Letter Figure L1A**) explaining that they show 'Diagnostic H&E sections'.

To comprehensively address the reviewer's concern and enhance the clarity of our spatial analysis presentation, we have taken several steps. In direct response to your request, we have now provided the clear, unlabeled, original high-resolution H&E-stained images of the Visium sections for all samples (HUH001-P, HUH001-P2, HUH001-met, and HUH002-P) in a **new Supporting Figure S3G** (**Letter Figure L1B**). This figure also includes the corresponding Space Ranger processed images (showing tissue detection, alignment, and spots) and aligned fiducial markers, allowing you to directly assess the tissue morphology on the Visium slide and verify the processing steps.

We next reexamined the annotation of HUH002-P. Specifically concerning the HUH002-P sample, three board-certified pathologists (T.Y., S.G., H.K.) independently re-examined and confirmed the tumor region annotation directly on the H&E-stained Visium image using Loupe Browser v6.0.0 (as described in the updated Methods: "Spatial RNA-seq data processing and

pathological annotation"). They verified that the annotation accurately reflects the tissue morphology visible within that particular Visium section. Space Ranger v1.3.0 processing for HUH002-P also showed robust spot detection and tissue alignment (3,234 spots under tissue, 98.9% reads in spots under tissue).

Our approach involves annotating broad tissue regions based on the Visium H&E and then using Tangram[1,2], a deep learning method integrating scRNA-seq data, to estimate the spatial distribution and composition of cell types, including OSCC cells and myCAFs, within each Visium spot. While Tangram offers high spatial resolution estimation[3], we acknowledge that this computational projection may differ slightly from the micro-anatomy of an adjacent diagnostic section. To validate the spatial localization of OSCC cells derived from this estimation, we compared the spatial expression pattern of the OSCC marker Keratin 16 (KRT16) on the Visium slides with the immunohistochemical staining pattern of the pan-cytokeratin marker CK AE1/AE3 on the adjacent diagnostic. The updated **Supporting Figure S4F-S4H** (**Letter Figure L1C**). displays the spatial expression patterns of KRT16 and ACTA2 overlaid on the Visium H&E. Based on our comparison, the spatial distribution of KRT16-high spots shows a high degree of concordance (approximately >90% overlap) with CK AE1/AE3 positive regions on the adjacent diagnostic sections, strongly supporting the accuracy of our tumor region localization in the spatial data. Similarly, the spatial pattern of the myCAF marker Actin Alpha 2, Smooth Muscle (ACTA2) aligns well with stromal regions identified on the H&E.

For complete details on the Space Ranger processing and quality metrics, we have included links to the interactive Web Summaries for each sample. The overall summary is available at https://kenflab.github.io/oscc_metastasis/summary.html, and specific links for each sample are as follows:
• **HUH001-P**:
https://kenflab.github.io/oscc_metastasis/data/spatial_transcriptome_data_summary/A2_web_summary.html
• **HUH001-P2**:
https://kenflab.github.io/oscc_metastasis/data/spatial_transcriptome_data_summary/B2_web_summary.html
• **HUH001-met**:
https://kenflab.github.io/oscc_metastasis/data/spatial_transcriptome_data_summary/C2_web_summary.html
• **HUH002-P**:
https://kenflab.github.io/oscc_metastasis/data/spatial_transcriptome_data_summary/D2_web_summary.html

We are confident that the comprehensive information provided, including the clear, unlabeled Visium H&E images (**Letter Figure L1B** [**new Supporting Figure S3G**]), the re-confirmed annotation of HUH002-P based on the Visium section, and the demonstration of marker gene spatial expression patterns (KRT16 in **updated Supporting Figure S4G-S4I [Letter Figure L1C]**) which showed high concordance with adjacent CK AE1/AE3 IHC, along with the explicit

clarifications in the text and legends, will comprehensively address your concern regarding the precise localization of OSCC cells and myCAFs within our spatial transcriptomic data.

Letter Figure L1A (updated Supplemental Figure S3A and S3B)

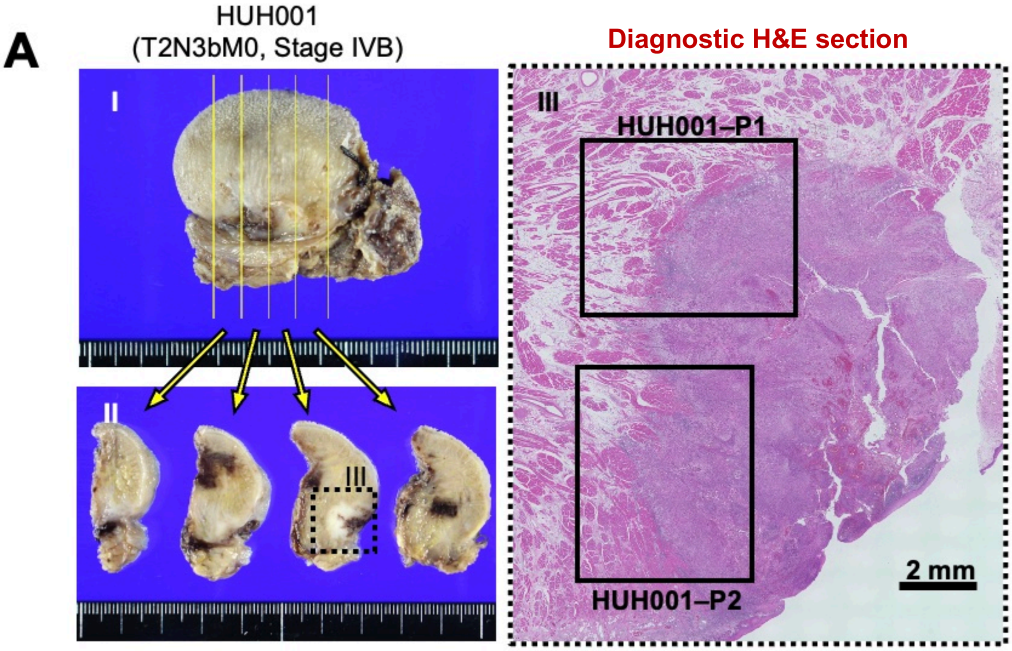

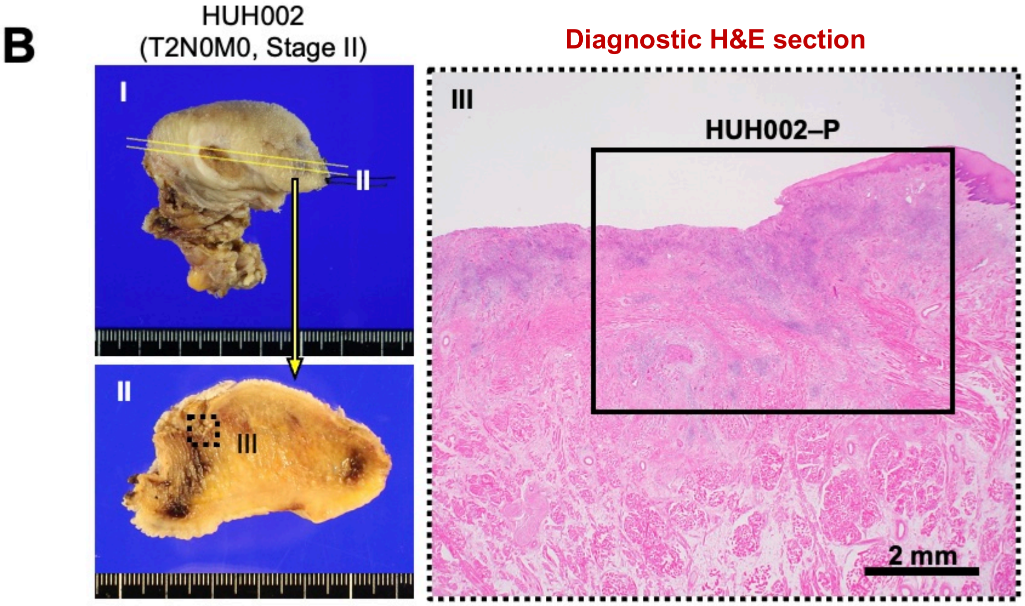

Letter Figure L1B (new Supporting Figure S3G)

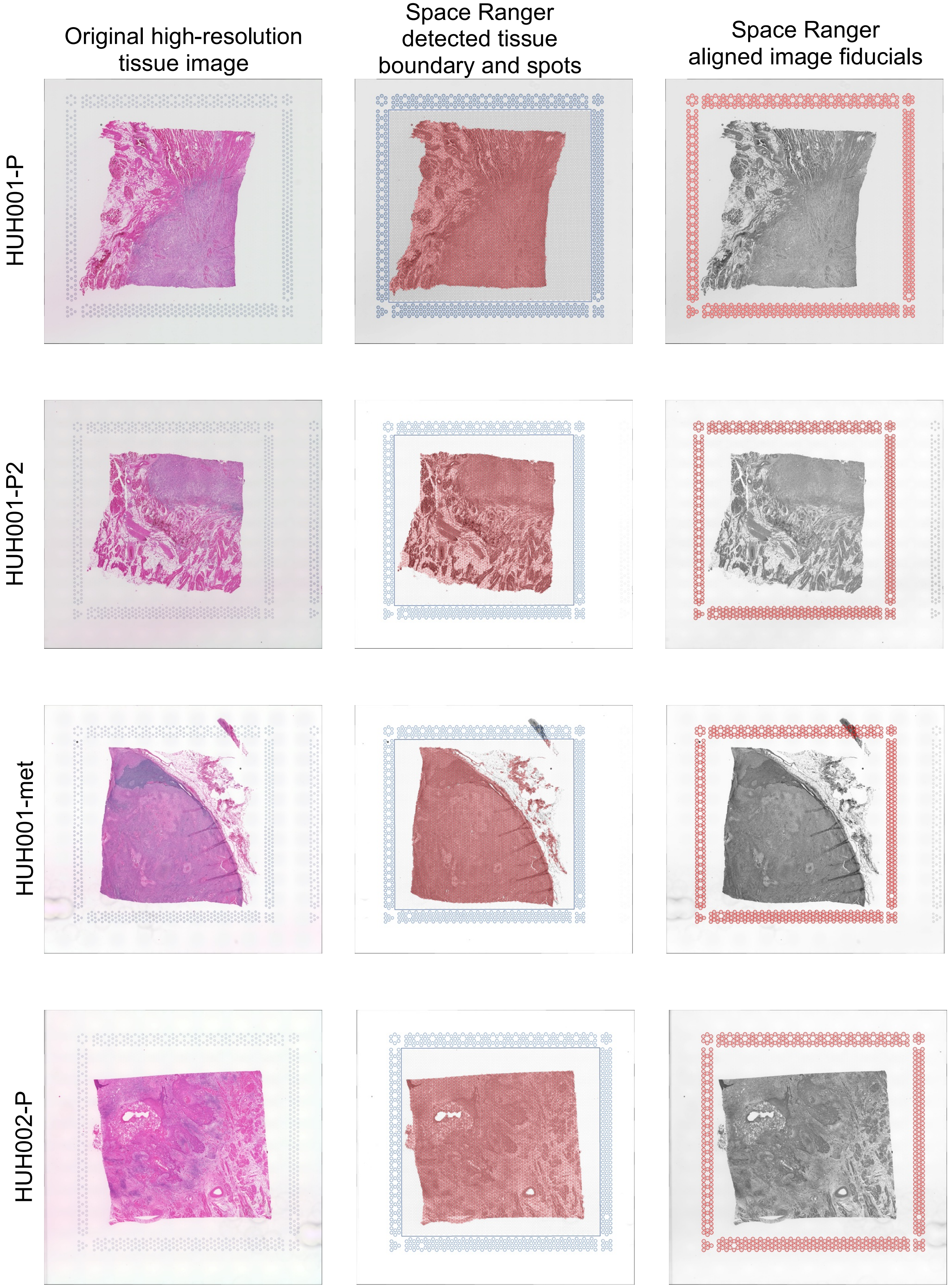

Letter Figure L1C (updated Supporting Figure S4G-S4I)

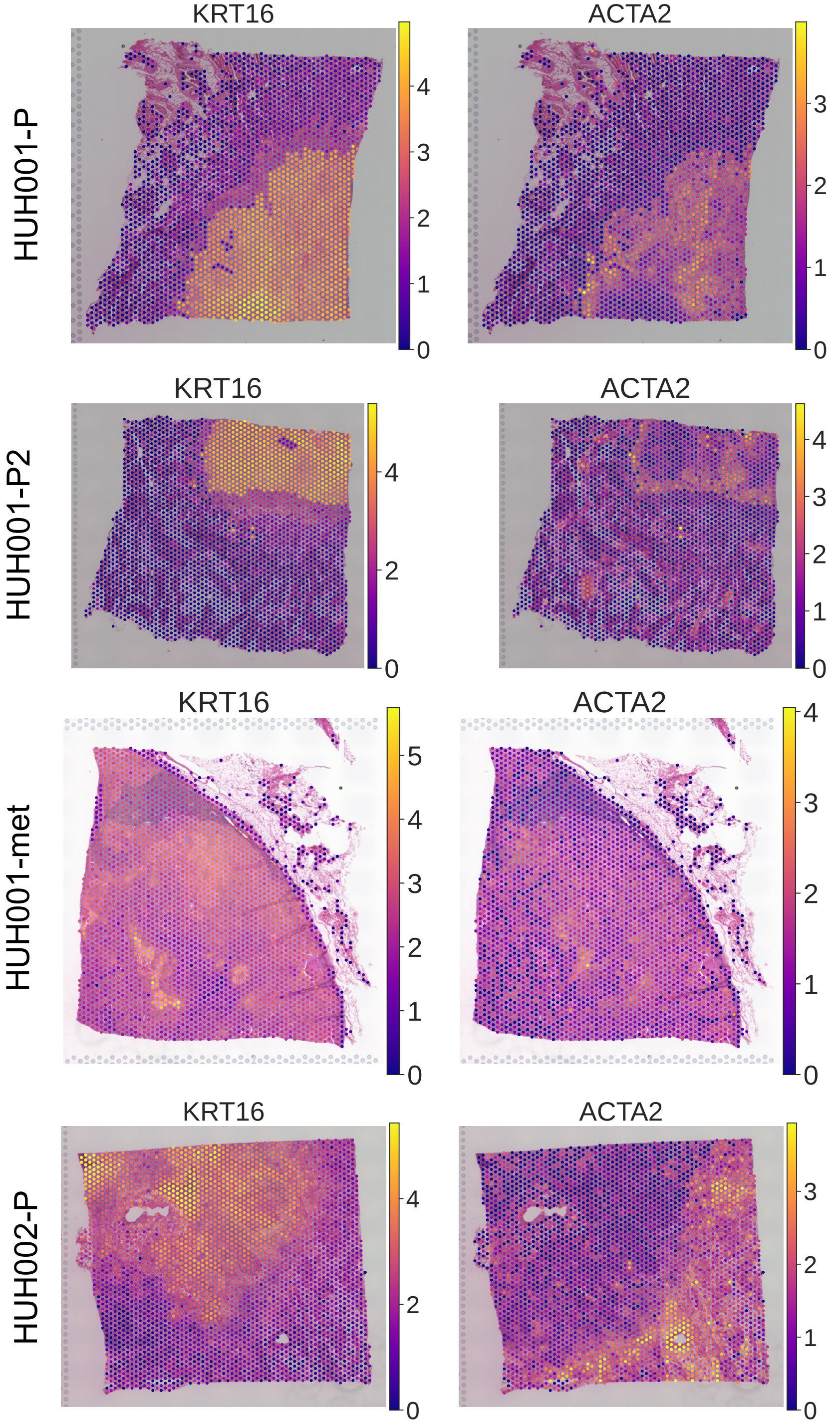

Revised Figures: **Supporting Figure S3A and S3B, new Supporting Figure S3G, and Supplementary Fig. S4G-S4I**

Revised text: Methods section ("FFPE sample preparation and hematoxylin and eosin—stained images for spatial transcriptome analysis" subsection).

2.Through spatial transcriptomic analysis, the author summarized the difference of myCAFS localization between LNM+sample and LNM-sample. Is the difference also related with the morphological characteristics of SCC nests (such as nest-like, lobulated, etc.) or with pathological grade?

RE: Thank you for the insightful comment regarding the potential relationship between myCAF localization, tumor morphology, and pathological grade. We agree that this is an important consideration for interpreting our spatial findings.

To address this comprehensively, we initially quantified myCAF accumulation using the absolute α-SMA-positive area per square millimeter (myCAF density). Additionally, we calculated the relative proportion of α-SMA-positive area to the sum of α-SMA- and CK AE1/AE3-positive areas (myCAF proportion relative to tumor area, **Letter Figure L2A;** 50 samples from 10 patients), to adjust for local differences in tumor–stromal balance, particularly in regions with irregular architecture wit in the invasive tumor fronts (ITFs).

Letter Figure L2A

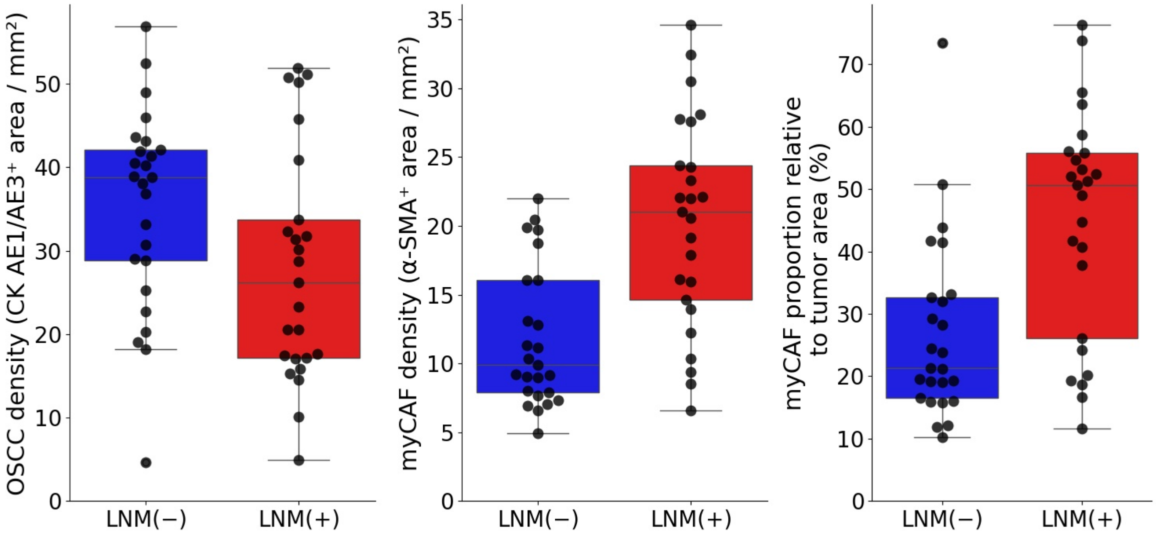

To further explore the relationship between myCAF distribution, tumor morphology, and pathological grade, we performed a systematic histopathological analysis on our initial cohort (50 samples from 10 patients). We specifically assessed the association between myCAF density and both the Pattern of Invasion (POI)[4] and pathological differentiation grades—well-differentiated (Grade 1), moderately differentiated (Grade 2), and poorly differentiated (Grade 3). Our analysis at this time revealed a statistically significant negative correlation between myCAF density and increasing pathological grade (Kruskal–Wallis, $P = 0.033$, **Letter Figure L2B**), with median values of 19.88% (95% bootstrap CI: 16.12–24.4) in Grade 1; 11.25% (95% bootstrap CI: 9.06–16.06) in Grade 2; 12.22% (95% bootstrap CI: 6.59–17.90) in Grade 3. In addition, Spearman's rank correlation analysis (Spearman's $\rho = -0.36$, $P = 0.01$), indicating a significant negative association between pathological grade and myCAF density. As a point of caution, the trend of negative association between myCAF density and pathological grade may reflect the more conservative selection of sample areas or that only one patient was the poorly differentiated OSCC (Grade 3; see also **Letter Table L1**).

Letter Figure L2B

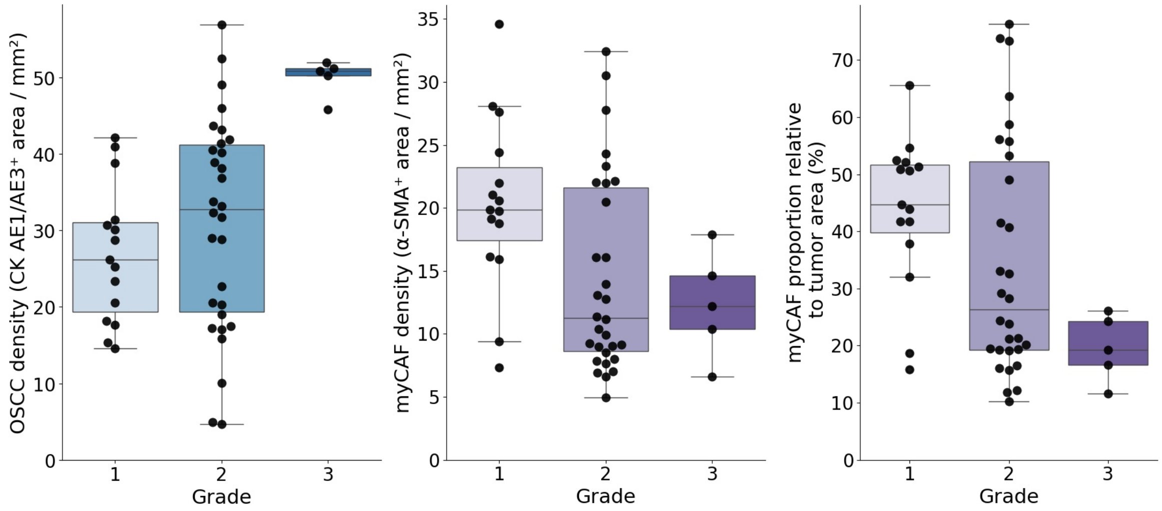

In contrast, no significant association was observed between myCAF density and POI classification (**Letter Figure L2C**).

Letter Figure L2C

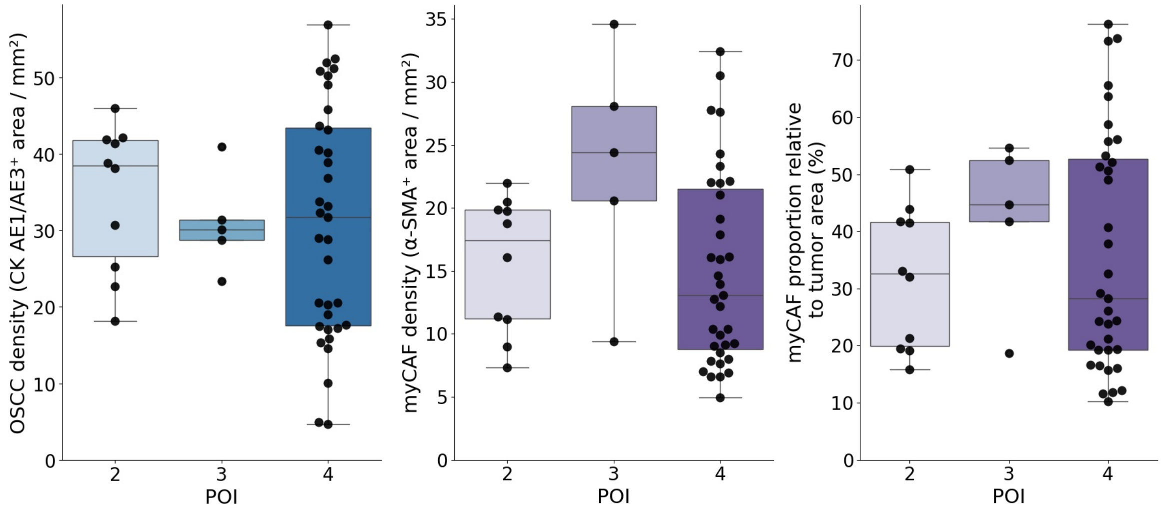

To independently evaluate their associations with lymph node metastasis (LNM) using this initial dataset, we conducted univariate logistic regression analyses, which identified myCAF density ($P = 0.001$; odds ratio (OR) =1.22; 95% CI, 1.09-1.36) and POI ($P = 0.015$; OR =2.90; 95% CI, 1.23-6.87) as significantly associated with LNM, whereas pathological grade and OSCC density were not significantly associated ($P = 0.058$, and $P = 1.0$, respectively; **Letter Figure L2D**).

Letter Figure L2D

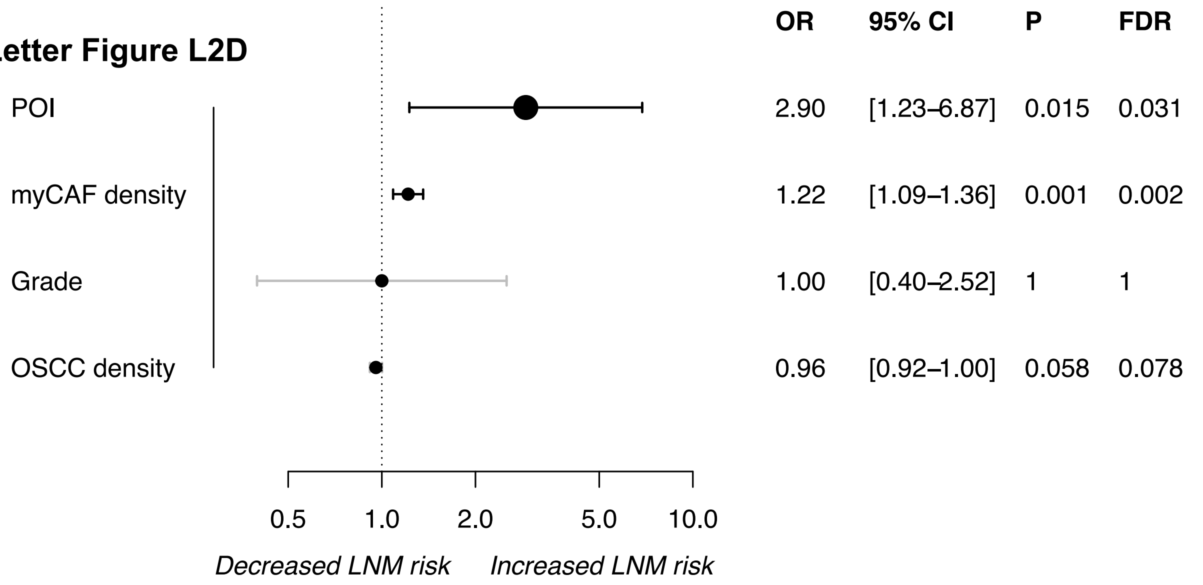

To assess whether the association between each variable and LNM persisted after adjustment for potential confounding factors, we performed multivariate logistic regression on this initial cohort. The model included myCAF density, POI, and pathological grade demonstrated that myCAF density remained an independent predictor of LNM ($P = 0.001$; OR = 1.44, 95% CI: 1.15–1.80; **Letter Figure L2E**). This suggested that the effect of myCAFs on LNM is not solely explained by morphological features or pathological grade.

Letter Figure L2E

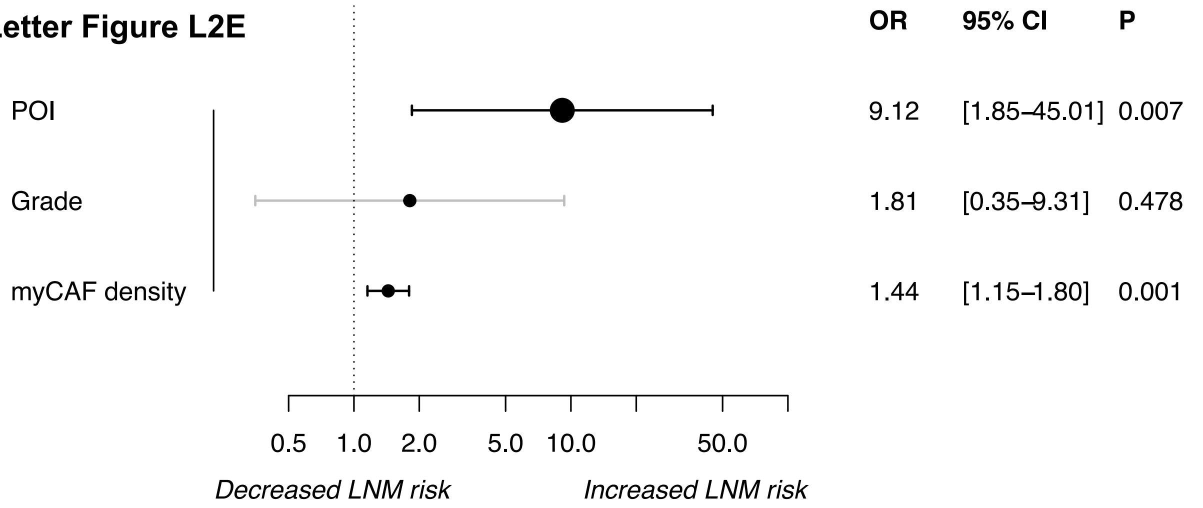

To further explore this relationship, we tested interaction terms between myCAF density and both POI and pathological grade. Neither interaction was statistically significant (myCAF density × POI: OR = 1.251, 95% CI: 0.956–1.638, $P = 0.103$; myCAF density × pathological grade: OR = 0.809, 95% CI: 0.568–1.153, $P = 0.242$), suggesting that the effect of myCAFs on LNM is not substantially modified by these histological features in this cohort. All statistical analyses were performed using absolute myCAF density (absolute α-SMA-positive area per square millimeter), which offers a standardized and interpretable measure of CAF burden across samples.

Based on these initial analyses with the smaller cohort, our understanding at that stage was that while myCAF accumulation appeared to be correlated with pathological grade, its significant association with LNM risk was independent of tumor morphological characteristics (POI) and differentiation status. The figures and tables supporting these particular observations were prepared accordingly for that version of our supplementary information."

Furthermore, in response to the reviewer's subsequent comment (Comment 3) regarding the sample size for IHC validation, we expanded our IHC cohort to 90 samples from 18 patients. The comprehensive re-analyses based on this larger dataset, including an updated assessment of the relationship between myCAF density and pathological grade, are detailed in our response to that specific comment (Comment 3) and have been fully incorporated into the current revised manuscript (please see your Comment 3's response, **Abstract, Results, Discussion, Methods, Supporting Table S4, and relevant Figure 7A-7C and Supporting Figure S4F).**"

Letter Table L1. Characteristics of 12 patients with OSCC from a clinical cohort, Related to Methods

Patient ID	Age	Sex	Pathologic T stage	Pathologic N stage	Tissue origin	Primary tumor site	Grade	POI
**HUH001**	48	F	T2	N3b	Tongue	Left lateral tongue	2	4
**HUH002**	61	F	T2	N0	Tongue	Right lateral tongue	2	3
**HUH003**	64	M	T2	N2	Tongue	Right lateral tongue	3	4
**HUH004**	80	F	T2	N1	Tongue	Left lateral tongue	2	4
**HUH005**	78	M	T2	N1	Tongue	Left lateral tongue	1	3
**HUH006**	45	M	T2	N1	Tongue	Right lateral tongue	2	4
**HUH007**	57	F	T2	N1	Tongue	Left lateral tongue	1	4
**HUH008**	68	F	T2	N0	Tongue	Left lateral tongue	2	4
**HUH009**	58	M	T2	N0	Tongue	Left lateral tongue	1	2
**HUH010**	59	M	T2	N0	Tongue	Base of tongue	2	2
**HUH011**	64	M	T2	N0	Tongue	Right lateral tongue	2	4
**HUH012**	34	M	T2	N0	Tongue	Left lateral tongue	2	4

3. The sample size in this study is too small to rule out the influence of the pathological morphology of the sample tissues on the results in figure 4. Although 50 additional IHC samples were used, but only from 10 patients, why not use 50 samples from 50 patients (one sample per patient) considering the different samples from one person have similar or same differentiation?

RE: Thank you for the comment regarding the sample size and our strategy of analyzing multiple invasive tumor front (ITF) samples per patient in the immunohistochemical (IHC) validation cohort. We appreciate the opportunity to clarify our approach. We agree that the total number of patients is a critical factor for generalizability. The rationale for sampling multiple ITF areas per patient was to capture the significant intra-patient heterogeneity inherent in the OSCC microenvironment (**Letter Figure 3A**).

Letter Figure L3A

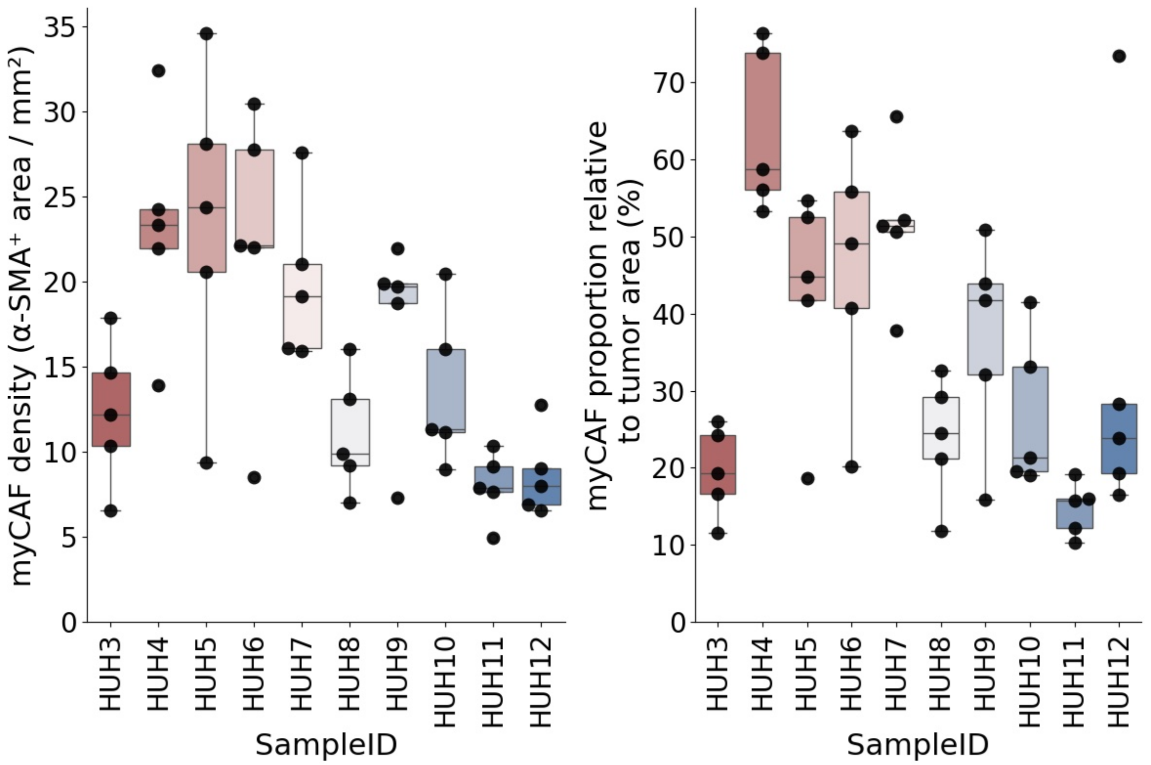

To quantify the degree of similarity or variability among these five ITF areas within each patient, we calculated the intraclass correlation coefficient (ICC) across the five ITF areas obtained per patient. Indeed, our analysis demonstrated significant intra-patient heterogeneity, as shown by the single-rater ICC was 0.55 (95% CI 0.28-0.83), indicating that approximately 45% of the variance resides within patients (**Letter Table L2**). This suggests a substantial degree of intra-patient heterogeneity in myCAF-related measures, highlighting the importance of sampling multiple ITF areas per patient to capture the full spectrum of the OSCC microenvironment.

Letter Table L2 Intraclass correlation coefficient (ICC) across the five ITF areas obtained per patient

ICC model	Measurement assumption	ICC estimate	F statistic	df_1	df_2	*P*-value	Lower 95% CI	Upper 95% CI
ICC (1, 1)	Single rater, absolute agreement	0.553	7.178	9	40	0.000004	0.28	0.83
ICC (2, 1)	Single rater, random raters	0.551	6.97	9	36	0.000009	0.27	0.83
ICC (3, 1)	Single rater, fixed raters	0.544	6.97	9	36	0.000009	0.26	0.82
ICC (1, k)	Average rater, absolute agreement	0.861	7.178	9	40	0.000004	0.66	0.96
ICC (2, k)	Average rater, random raters	0.86	6.97	9	36	0.000009	0.65	0.96
ICC (3, k)	Average rater, fixed raters	0.857	6.97	9	36	0.000009	0.64	0.96

Abbreviations: ICC, intraclass correlation coefficient; df, degrees of freedom; CI, confidence interval. Lower than 0.05 were considered to indicate statistical significance.

To account for the non-independence of multiple ITF measurements within each patient, we then performed a generalized estimating equation (GEE) analysis using Patient ID as the clustering variable (**Letter Table L1**). This yielded a positive, borderline-significant association between myCAF fraction and lymph node metastasis (LNM, $\beta = 0.0006 \pm 0.0003$, $P = 0.062$; OR per 10% increase = 1.006), suggesting that higher myCAF fractions may be associated with an increased risk of LNM, even after accounting for within-patient clustering.

Because effect estimates can be scale-dependent, we performed a sensitivity analysis using one randomly selected ITF area per patient and repeating the logistic regression 10,000 times. The median odds ratio was 1.08 (95% CI 1.01–1.30; **Letter Figure L3B)**, suggesting that the association between myCAF density and LNM is robust to variability in ITF area selection and that our findings remain stable even when accounting for the limited sample size of 10 patients.

Letter Figure L3B

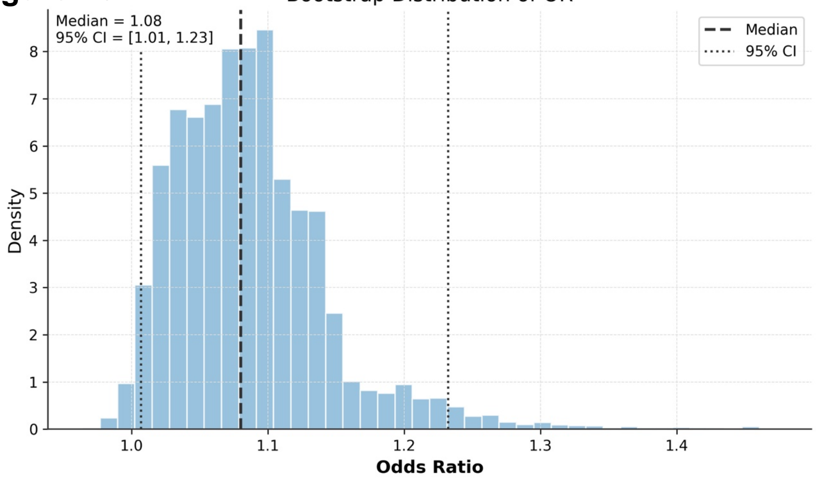

To address the reviewer's concern about the overall patient number, we expanded the IHC cohort to include 90 ITF samples from 18 OSCC patients with T2 primary tumors (9 with LNM, 9 without LNM), increasing the overall patient number compared to our initial analyses (**Letter Figure L3C [updated Figure 7A-7B]; Letter Table L4[updated Supporting Table S4]; Supporting Figure S4F-S4G**). These analyses, including the quantification of intra-patient heterogeneity, the use of appropriate statistical methods for clustered data, a comprehensive sensitivity analysis, and the increase in patient numbers, rigorously support our sampling strategy and the robustness of our conclusions regarding the association between myCAF density and LNM. We have updated the Methods section and relevant Supporting Figures/Tables to fully describe these approaches and present the supporting data.

Letter Figure L3C (updated Figure 7)

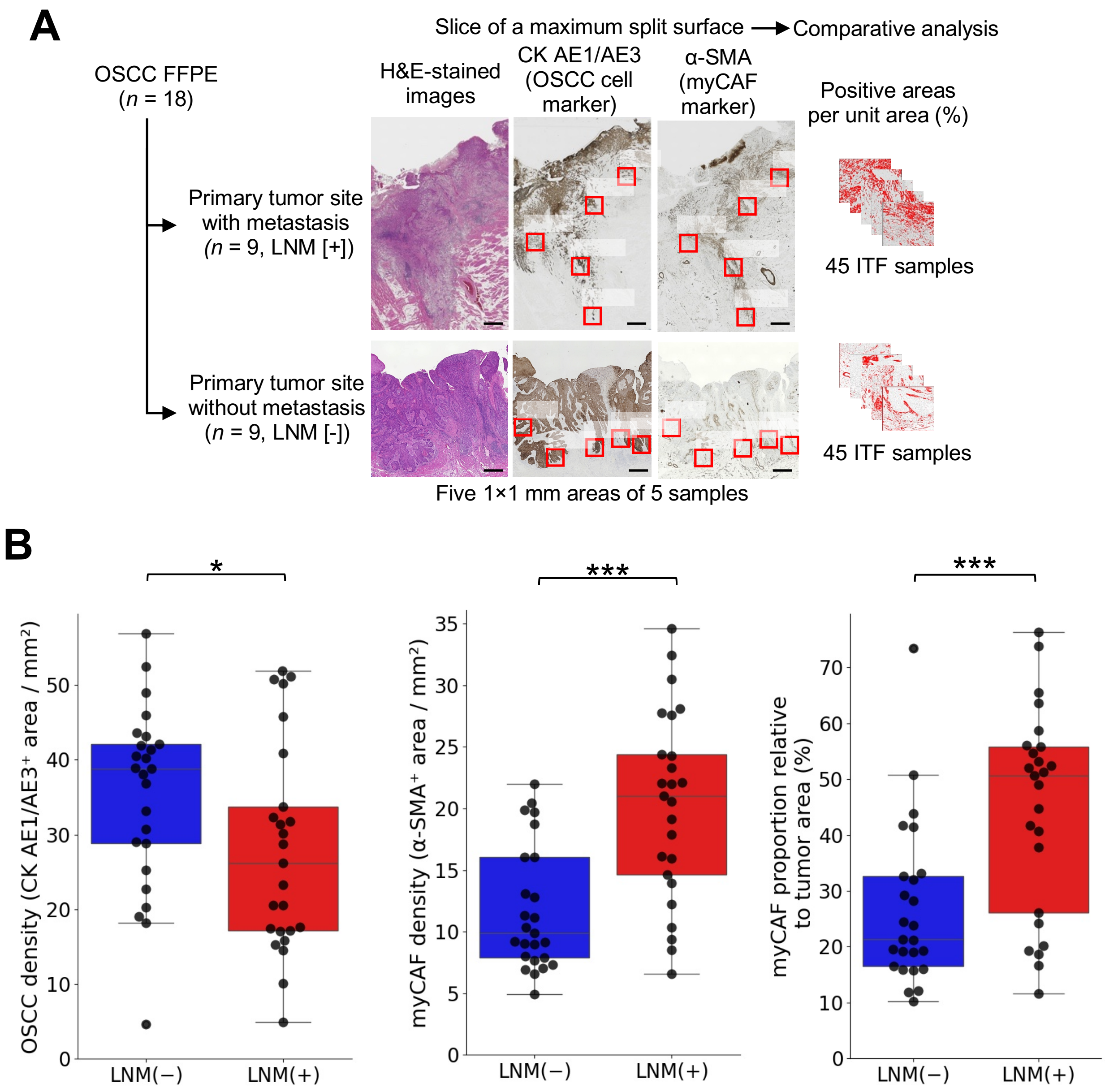

Letter Table L3 (new Supporting Table S4). Characteristics of 20 patients with OSCC from a clinical cohort.

**Patient ID**	**Age**	**Sex**	**Pathologic T stage**	**Pathologic N stage**	**Tissue origin**	**Primary tumor site**	**Grade**	**POI**
**HUH001**	48	F	T2	N3b	Tongue	Left lateral tongue	2	4
**HUH002**	61	F	T2	N0	Tongue	Right lateral tongue	2	3
**HUH003**	64	M	T2	N2	Tongue	Right lateral tongue	3	4
**HUH004**	80	F	T2	N1	Tongue	Left lateral tongue	2	4
**HUH005**	78	M	T2	N1	Tongue	Left lateral tongue	1	3
**HUH006**	45	M	T2	N1	Tongue	Right lateral tongue	2	4
**HUH007**	57	F	T2	N1	Tongue	Left lateral tongue	1	4
**HUH008**	68	F	T2	N0	Tongue	Left lateral tongue	2	4
**HUH009**	58	M	T2	N0	Tongue	Left lateral tongue	1	2
**HUH010**	59	M	T2	N0	Tongue	Base of tongue	2	2
**HUH011**	64	M	T2	N0	Tongue	Right lateral tongue	2	4
**HUH012**	34	M	T2	N0	Tongue	Left lateral tongue	2	4
**HUH013**	46	M	T2	N1	Tongue	Left lateral tongue	3	5
**HUH014**	79	M	T2	N1	Gum	Left alveolar ridge	3	3
**HUH015**	57	M	T2	N1	Cheek mucosa	Left buccal mucosa	2	4
**HUH016**	58	M	T2	N1	Tongue	Left lateral tongue	2	3
**HUH017**	63	F	T2	N0	Tongue	Left lateral tongue	1	3
**HUH018**	56	M	T2	N0	Tongue	Left lateral tongue	2	2
**HUH019**	71	M	T2	N0	Tongue	Left lateral tongue	1	3
**HUH020**	79	F	T2	N0	Tongue	Left lateral tongue	2	2

new Supporting Figure S4

F

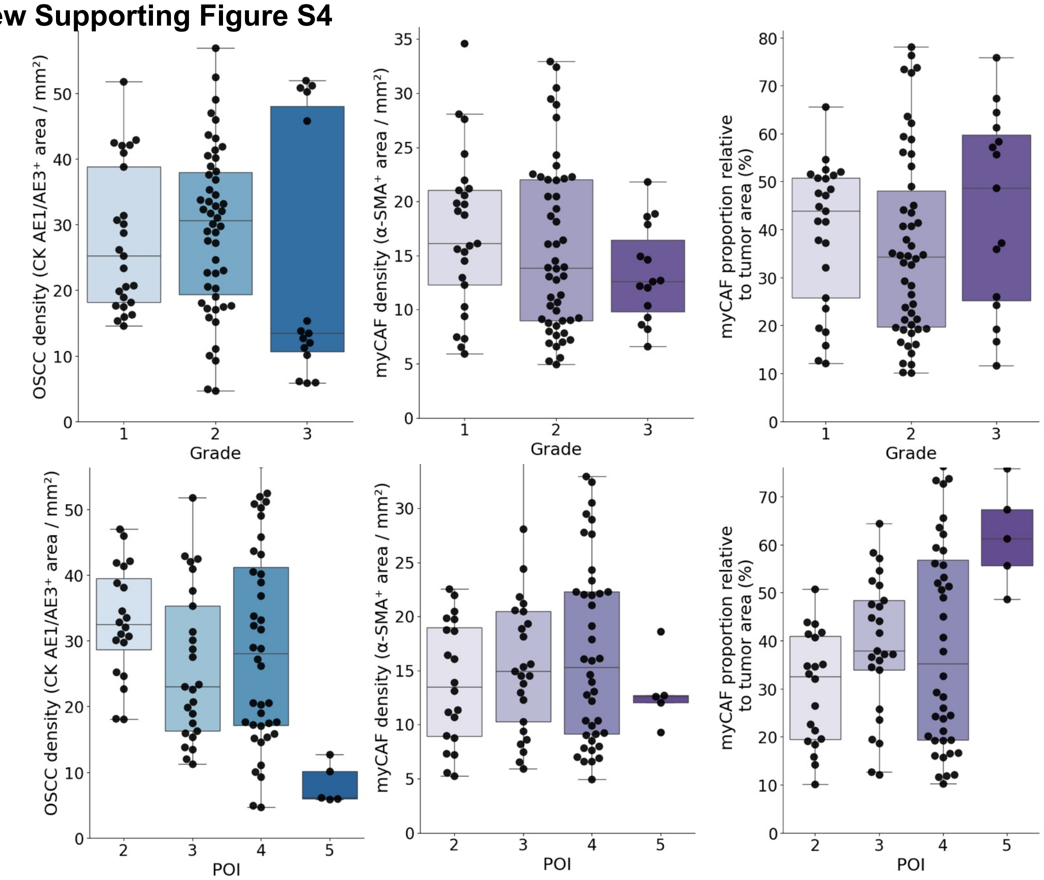

new Figure 7

C

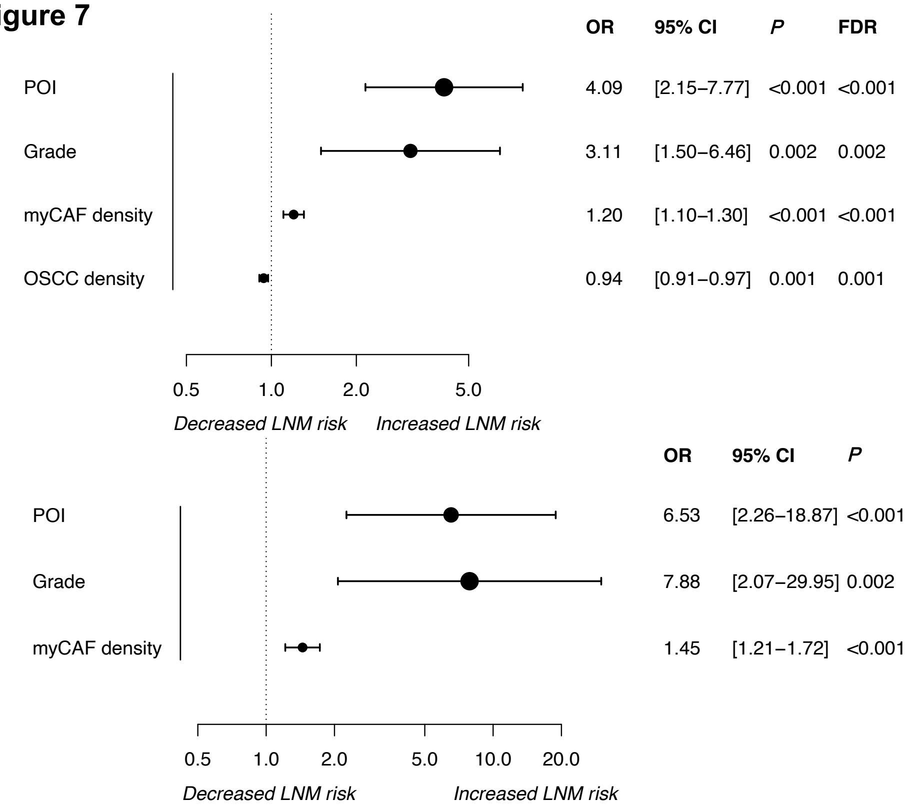

UPDATED ABSTRACT: Lymph node metastasis (LNM) is a critical prognostic factor for patients with oral squamous cell carcinoma (OSCC). Previous research has implicated the partial epithelial-to-mesenchymal transition of tumor cells and myofibroblastic cancer-associated fibroblasts (myCAFs) in the LNM process. However, the underlying molecular mechanisms remain poorly understood. Here, we conducted a comprehensive molecular analysis integrating original and publicly available OSCC data from bulk genome and transcriptome, single-cell transcriptome, and spatial transcriptome analyses. We found that myCAFs were quantitatively and functionally activated in LNM-positive samples and spatially colocalized with OSCC cells within the invasive tumor front (ITF), providing a niche that may facilitate LNM. Immunohistochemical validation in 90 ITF samples confirmed significantly higher myCAF density in LNM-positive samples, and this density remained an independent predictor of LNM when adjusted for pathological grade and the pattern of invasion. In LNM-positive samples, myCAFs provided increased extracellular matrix signals, upregulating stemness-related genes such as *CD44* in OSCC cells. The functional importance of this myCAF-driven ECM-CD44 axis was further supported by our validation analysis of expanded, publicly available spatial transcriptome and experimental in vitro coculture data. We also extracted a spatially resolved, 23-gene signature from the metastatic ITF where OSCC and myCAFs colocalize. This signature predicted LNM status and poor overall survival in OSCC patients. Our findings provide novel insight into the molecular myCAF/OSCC crosstalk that facilitates LNM and identify potential prognostic biomarkers and therapeutic targets for OSCC patients.

UPDATED RESULTS To validate these findings in a larger cohort, we then conducted an IHC analysis of 90 additional samples of ITFs from 18 patients with OSCC (9 with LNM and 9 without LNM; Figure 7A). All selected patients had stage T2 primary tumors as determined by the tumor sizes and locations (Table S4). Consistent with the findings from the spatial transcriptomic analysis, significantly higher numbers of the absolute α-SMA-positive area per square millimeter (myCAF density) were detected in the ITFs of samples from patients with LNM compared with those of samples from patients without LNM (median : 19.14% [95% CI, 15.92%-21.99%] vs. 10.70% [95% CI, 9.06%-13.12%] , respectively; P <0.001; Figure 7B). Moreover, histopathological analyses were conducted. myCAF density did not significantly correlate with pathological grade or POI (Spearman correlation = −0.16, P = 0.14, Spearman correlation = 0.06, P = 0.60; respectively; Figure S4F). Importantly, in multivariable logistic regression adjusted for pathological grade and POI, myCAF density was identified as an independent predictor of LNM (OR = 1.45, 95% CI: 1.21–1.72, P<0.001; Figure 7C), underscoring the association between myCAF presence within the ITFs and LNM risk.

UPDATED DISCUSSION: A limitation of this study was the small sample size in the spatial transcriptomic analysis. To overcome this, we validated our findings regarding the spatial colocalization of myCAFs and OSCC cells using publicly available 12 more spatial transcriptomic OSCC samples and 90 additional IHC samples (see Table S4 for details). These analyses confirmed a significant association between increased myCAF density at the ITFs and LNM. Building on this, multivariable analysis of these 90 IHC samples confirmed myCAF density as an independent LNM predictor, even when controlling for pathological grade and POI. Since myCAF density itself did not significantly correlate with them, it offers distinct information for predicting LNM beyond these established clinicopathological parameters. Supportive In vitro data from other studies also provided mechanistic support for our observed

ECM-CD44 axis. Additionally, the prognostic significance of the 23-gene signature that we extracted from the spatial analysis was validated in separate clinical TCGA cohorts and 2 other microarray datasets. We believe that these efforts successfully addressed the limitations regarding the small sample size in the spatial transcriptomic analysis.

UPDATED METHODS: **IHC analysis of 90 ITF samples from T2 OSCC patients**
To measure the rates of CK AE1/AE3 positivity and αSMA positivity in ITF samples, we performed an IHC analysis of 90 ITF samples from 18 patients with T2 primary tumors: 9 patients (45 samples) had LNM and 9 patients (45 samples) did not (Table S4). FFPE tissue sections representative of tumor cross-sections underwent standard immunostaining procedures. Images of the tumor sections (stained H&E, CK AE1/AE3, and α-SMA) were acquired using an all-in-one BZ-X800 fluorescence microscope (Keyence). We randomly selected 5 ITF areas (each 1.0 × 1.0 mm^2) from a wide tumor area. The same regions were extracted from the specimens stained with α-SMA. We binarized the images using ImageJ software (RRID: SCR_003070, Java 1.6.0_24 [64 bit]; National Institutes of Health, Bethesda, MD, USA). The binarized images showed either positive or negative immunostaining. We calculated the percentage of CK AE1/AE3–positive cells (OSCC cells) and α-SMA–positive cells (myCAFs) per unit area (1.0 × 1.0 mm^2).

Statistics and reproducibility Statistical analyses were performed using R v3.6.2. No statistical method was used to predetermine the sample size for the bulk genome, transcriptome, single-cell transcriptome, and spatial transcriptome analyses, as these were exploratory studies in which the effect sizes of factors related to LNM were not predetermined. The sample size for the IHC analysis estimated using G*Power v3.1.9.7 [69] (RRID:SCR_013726) (2-sided Mann-Whitney U test; significance level [α] = 0.05; target power [1 − β] = 0.8; allocation ratio N2/N1 = 1.0; effect size [d] = 0.8). Univariate logistic regression analyses were initially performed. Subsequently, multivariable logistic regression was used to determine the independent predictive value of myCAF density for LNM, after adjusting for pathological grade and Pattern of Invasion (POI), in a clinical cohort (refer to Table S4 for patient characteristics and the specific cohort used for this analysis).

Revised text**: Abstract, Result, Discussion, Methods** section ("**IHC analysis of 90 ITF samples from T2 OSCC patients**" and "**Statistics and reproducibility" subsection**).
Revised Figures**: Figure 7A-7C; Supporting Figure S4F.**
Revised Table**: Supporting Table S4**.

4.The clinical application potential of the 23 gene markers should be described or discussed in more depth.

RE: We thank the reviewer for this critical point. We have clarified the prognostic polarity (Favorable/Unfavorable) of each gene within the 23-gene signature in the updated Supporting Table S12 (previously Letter Table L4). Furthermore, we have compiled a new Supporting Table S13 (previously Letter Table L5) summarizing the key biological roles and current or emerging clinical applications (diagnostic, prognostic, predictive, or therapeutic) for each of these 23 genes. Importantly, building on this, we have expanded the Discussion to address the potential

clinical utility of this 23-gene signature as a prognostic tool, including the prospect of developing a multi-gene assay for OSCC.

First, in the revised manuscript we have clarified the prognostic polarity of each gene and have provided the potential clinical applications of 23 gene signature. Genes with a positive LASSO coefficient increase the predicted hazard and are therefore classified as **Unfavorable** prognostic biomarkers, whereas those with a negative coefficient reduce risk and are designated **Favorable**. Among the 23 genes, 15 are **Unfavorable**, and 8 are **Favorable**, underscoring the detrimental impact of a myCAF-rich microenvironment. We have added this information to the footnote in **the updated Supporting Table S12 (Letter Table L4)**, which orders the genes by Spearman correlation with myCAF density and explicitly lists the direction and magnitude of the LASSO weight.

Letter Table L4 (updated Supporting Table S12.) Spatially-resolved 23 core signature genes are ranked by spearman correlation with myCAFs proportion.

Gene Symbol	Coefficient	Gene Weight	Spearman with myCAFs	Adjusted *P*-value	Description
**SFRP2**	Unfavorable	0.318	0.648	7.31E-25	secreted frizzled related protein 2
**NES**	Favorable	-0.162	0.634	1.35E-23	nestin
**MRC2**	Unfavorable	0.364	0.592	4.80E-20	mannose receptor C type 2
**COL15A1**	Favorable	-0.407	0.529	1.47E-15	collagen type XV alpha 1 chain
**CCN2**	Unfavorable	0.315	0.498	1.21E-13	cellular communication network factor 2
**MFGE8**	Favorable	-0.408	0.412	3.53E-09	milk fat globule EGF and factor V/VIII domain containing
**PTX3**	Unfavorable	0.131	0.399	1.34E-08	pentraxin 3
**S100A4**	Favorable	-0.511	0.368	2.42E-07	S100 calcium binding protein A4
**C3**	Unfavorable	0.191	0.333	4.50E-06	complement C3
**UBXN6**	Favorable	-0.18	0.293	7.57E-05	UBX domain protein 6
**DES**	Unfavorable	0.374	0.289	9.81E-05	Desmin
**CORO1A**	Favorable	-0.243	0.238	0.002	coronin 1A
**CFD**	Unfavorable	0.242	0.214	0.007	complement factor D
**NOM1**	Unfavorable	0.213	0.192	0.018	nucleolar protein with MIF4G domain 1
**NTMT1**	Unfavorable	0.214	0.192	0.018	N-terminal Xaa-Pro-Lys N-methyltransferase 1
**MRFAP1L1**	Favorable	-0.338	0.15	0.083	Morf4 family associated protein 1 like 1
**C4orf3**	Unfavorable	0.122	0.146	0.093	chromosome 4 open reading frame 3
**ABHD16A**	Unfavorable	0.235	0.101	0.297	abhydrolase domain containing 16A, phospholipase
**LUC7L3**	Favorable	-0.28	0.074	0.475	LUC7 like 3 pre-mRNA splicing factor
**PIM3**	Unfavorable	0.289	0.063	0.561	Pim-3 proto-oncogene, serine/threonine kinase
**OLR1**	Unfavorable	0.227	0.055	0.62	oxidized low density lipoprotein receptor 1
**IFNGR2**	Unfavorable	0.245	0.038	0.749	interferon gamma receptor 2
**TXNDC17**	Unfavorable	0.166	-0.139	0.117	thioredoxin domain containing 17

Genes labeled ‘Unfavorable’ with a positive LASSO weight act as poor-prognosis biomarkers, whereas ‘Favorable’ genes with a negative weight act as good-prognosis biomarkers.

Second, to illustrate potential clinical applications, we have created a **new Supporting Table 13** (**Letter Table L5**). For each gene we summarized the key biological role, current or emerging clinical applications—diagnostic, prognostic, predictive, or therapeutic—and representative references. Examples include the anti-SFRP2 monoclonal antibody currently entering first-in-human trials for solid tumors, pamrevlumab (FG-3019) targeting CCN2 (CTGF) in a phase III study for pancreatic cancer, and several PIM-kinase inhibitors (e.g., AZD1208) that have completed phase I evaluations. In addition, OLR1 (LOX-1) has recently been validated as a surface marker for polymorphonuclear myeloid-derived suppressor cells (PMN-MDSC); anti-LOX-1 antibodies deplete these cells and enhance e checkpoint blockade in pre-clinical models. We trust this focused summary addresses the reviewer's request.

Letter Table L5 (**new Supporting Table S13**.) Spatially-resolved 23 signature genes: biology & clinical relevance.

Gene Symbol	Key Biological Role	Current or Emerging Application	Reference
**SFRP2**	Wnt antagonist; tumor angiogenesis	Stool DNA test (CRC); anti-SFRP2 mAb	[5–9]
**NES**	Stem-cell filament; neo-angiogenesis	IHC prognostic marker in OSCC, BRCA, GBM	[10–14]
**MRC2(Endo180)**	Collagen uptake; invasion	Endo180-ADC in sarcoma PDX; myCAF targeting boosts immunotherapy.	[15–19]
**COL15A1**	Non-fibrillar collagen; vascular niche	Immune–stromal prognostic marker; MSI/TMB correlate	[20–22]
**CCN2(CTGF)**	ECM remodelling; fibrosis	Pamrevlumab (FG-3019, NCT03941093, PDAC)	[23–26]
**MFGE8**	PS bridging; efferocytosis	IHC marker of local recurrence in OSCC	[27,28]
**PTX3**	Long pentraxin; complement tuner	Plasma prognostic biomarker; immune-modulation target	[29–31]
**S100A4**	Metastasin; EMT & MMPs	IHC predictor of nodal metastasis; S100A4-RAGE inhibitors (pre-cl.)	[32–35]
**C3(CPAMD1)**	Central complement node	Serum C3a/C5a correlate with OSCC stage; C3-inhibitors (AMY-101)	[36–38]
**UBXN6(UBXD1)**	p97 adaptor; IFN-STAT stabilizer	Low UBXN6 predicts relapse (gastric CA)	[39–41]
**DES(CSM1)**	Muscle filament; myofibre marker	Pathology aid for muscle invasion depth	[42,43]
**CORO1A(p57)**	Actin regulator; immune cell motility	High in LOX-1^{+} PMN-MDSC; target to curb metastasis	[36,44,45]
**CFD(ADIPSIN)**	Trigger of complement alternative pathway	Danicopan inhibits CFD; high CFD in cSCC	[37,46,47]
**NOM1(SGD1)**	Nucleolar ribosome biogenesis	New targets; Part of high-proliferation gene hubs in OSCC	[48–51]
**NTMT1**	N-terminal methyltransferase; genome stability	High NTMT1 → poor OS; radiosensitisation when inhibited	[52–59]

**MRFAP1L1(PP784)**	Chromatin remodel associate	Candidate prognostic marker in RCC	[60]
**C4orf3(ARLN)**	ER membrane protein; Ca^{2+} homeostasis	Exploratory; potential ER-stress modulator	[61,62]
**ABHD16A**	Lyso-PS synthase; immune suppression	ABHD16A inhibitors enhance ICI in mice	[36,63,64]
**LUC7L3**	Pre-mRNA splicing factor	Potential HCC prognostic biomarker	[49,65]
**PIM3**	Ser/Thr kinase; survival & mTOR	PIM inhibitors (AZD1208, SGI-1776) in early trials	[41,66–72]
**OLR1(LOX-1)**	Scavenger receptor; MDSC marker	Anti-LOX-1 depletes PMN-MDSC; prognostic in HNSCC	[36,73–75]
**IFNGR2**	IFN-γ receptor β-chain; Ligand activates JAK/STAT	Loss/mutation → ICI resistance biomarker	[76–79]
**TXNDC17(TRP14)**	Redox enzyme;	High TXNDC17 → taxane resistance (ovarian CA)	[80–82]

CRC, colorectal cancer; OSCC, oral squamous cell carcinoma; BRCA, breast invasive carcinoma; GBM, glioblastoma multiforme; PDX, patient-derived xenograft; cSCC, cutaneous squamous cell carcinoma; ICI, iommune checkpoint inhibitor; HCC, hepatocellular carcinoma; PMN-MDSC, polymorphonuclear myeloid-derived suppressor cells; HNSCC, head and neck squamous cell carcinoma.

UPDATED DISCUSSION:

Beyond providing insights into myCAF/OSCC crosstalk facilitating LNM, our 23-gene signature offers prognostic value. While gene expression profiling is not currently standard for OSCC prognosis, our findings suggest that this 23-gene signature, reflecting myCAF activity at the ITF, has the potential to be developed into a clinically relevant gene transcription assay. Similar to the Oncotype DX Breast Recurrence Score test, which uses 21 genes in early-stage breast cancer to assess recurrence risk and predict chemotherapy benefit, this assay could significantly improve risk stratification and clinical management for patients with OSCC. Furthermore, individual components of this signature highlight potential therapeutic avenues (Supporting Table S13). For instance, unfavorable signature genes such as *SFRP2* (a Wnt modulator with antibodies in early trials), *CCN2* (targeted by pamrevlumab), *MRC2* (target for Endo180-ADC platforms), *OLR1* (target for depleting immunosuppressive cells), and *PIM3* (target for PIM inhibitors like AZD1208) are already under investigation. While the clinical utility of this 23-gene signature as a comprehensive assay and the precise roles of its constituent genes within the OSCC microenvironment require rigorous validation, its development could provide valuable prognostic tools and novel therapeutic targets.

Revised text: **Discussion**.

Revised Table: Supporting Table S12 and **Supporting Table S13.**

Reviewer #2: In the manuscript, the authors tried to identify the molecular mechanisms behind the development of lymph node metastasis and poor prognosis of OSCC using spatial transcriptomic analyses. To support their findings, they also used public data.
The study showed some interesting points in regard to OSCC by spatial transcriptomic analyses from VISIUM, 10x genomics. myCAFs were found spatially colocalized with OSCC cells in the invasive front of the tumor. These CAFs were found to upregulate ECM-related genes and ECM outgoing signaling from myCAFs to OSCC cells in LNM positive samples. Especially the authors identified the 23-gene signature associated with LNM status that predicted poor overall survival in OSCC patients.

RE: Thank you for the comprehensive summary of our work and highlighting the key findings.

Although the research objectives and results are interesting, there are some areas that could be improved to further clarify the findings:
1. It would be interesting to expand the study by identifying a specific tumor cluster associated with metastasis to the lymph nodes. For example, analyzing the developmental trajectories of tumor cells could be a valuable addition.

RE: Thank you for your insightful suggestion to identify specific tumor clusters associated with lymph node metastasis (LNM) and explore cellular trajectories. We agree that this analysis enhances our understanding of the metastatic process. To address this, we applied pseudotime trajectory analysis to our spatial transcriptomics data using stLearn[83], which integrates spatial context with gene expression. This analysis aimed to identify spatial transitions and states associated with the invasive front and potential metastatic progression. Our analysis revealed that Metastatic Clusters (Cluster 1, 2, and 4-11) exhibited significantly longer pseudotime compared to Primary Tumor Clusters (median: 0.34 [95% bootstrap CI, 0.33-0.34] vs 0.19 [95% bootstrap CI, 0.17-0.20], respectively; P <0.001), suggesting an advanced developmental state in Metastatic Clusters (**Letter Figure L4A [Supporting Figure S5F-S5G]**). Because tumor progress towards the ITFs, we set Cluster 3 (Primary Tumor Cluster) as the trajectory root.

Letter Figure L4A (Supporting Figure S5F-S5G)

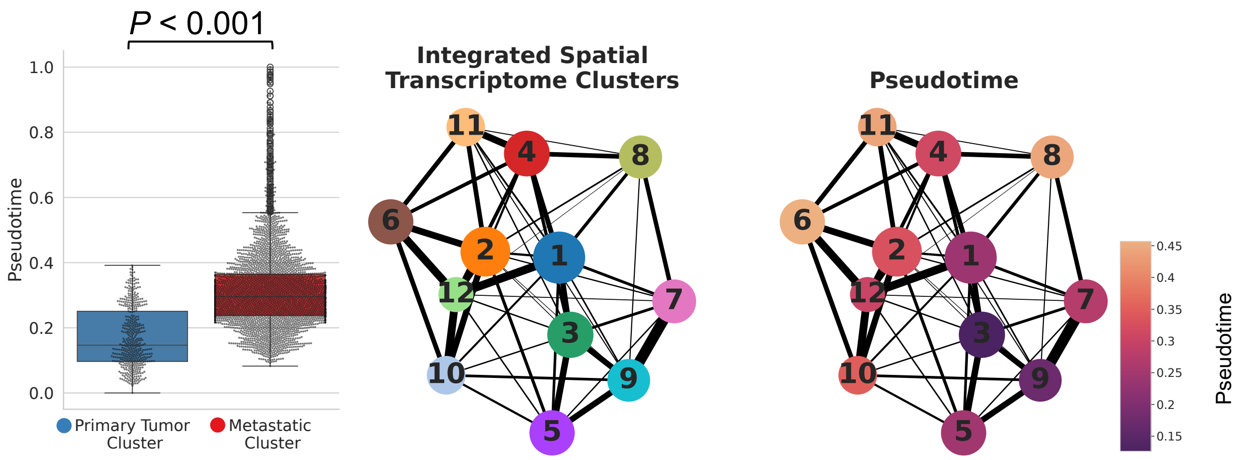

We identified several spatial clusters with distinct molecular profiles and pseudotime characteristics. While the interpretation of pseudotime in spatial data can be complex, our analysis highlighted specific clusters (e.g., Clusters 1, 5, 7, and 9) that demonstrated shorter pseudotime than Primary Tumor Cluster 12, coupled with elevated *CD44* expression. These findings indicate increased expression of stemness markers and exhibiting features potentially associated with metastatic capabilities (**Letter Table L6[new Supporting Table S10]; Letter Figure L4B [new Supplemental 5H]**).

Letter Table L6(new Supporting Table S10)

Integrated Spatial Transcriptome Cluster	CD44 expression					Pseudotime		
	median	25th percentile	75th percentile	P	adj P	median	25th percentile	75th percentile
1	1.59	1.34	1.78	0.008	0.012	0.28	0.26	0.30
2	1.67	1.32	1.95	<0.001	<0.001	0.37	0.35	0.38
3	1.65	1.51	1.79	<0.001	<0.001	0.16	0.13	0.20
4	1.44	1.13	1.67	0.307	0.338	0.34	0.31	0.37
5	1.99	1.76	2.20	<0.001	<0.001	0.26	0.21	0.31
6	1.50	1.12	1.90	0.571	0.571	0.48	0.45	0.52
7	1.67	1.42	1.92	<0.001	<0.001	0.32	0.30	0.33
8	1.61	1.24	1.87	0.052	0.063	0.40	0.35	0.52
9	1.86	1.62	2.09	<0.001	<0.001	0.20	0.18	0.24
10	1.87	1.44	2.20	<0.001	<0.001	0.39	0.35	0.41
11	1.34	0.00	1.72	0.013	0.018	0.43	0.37	0.57
12	1.46	1.17	1.71			0.33	0.32	0.36

Letter Figure L4B (new Supporting Figure S5H)

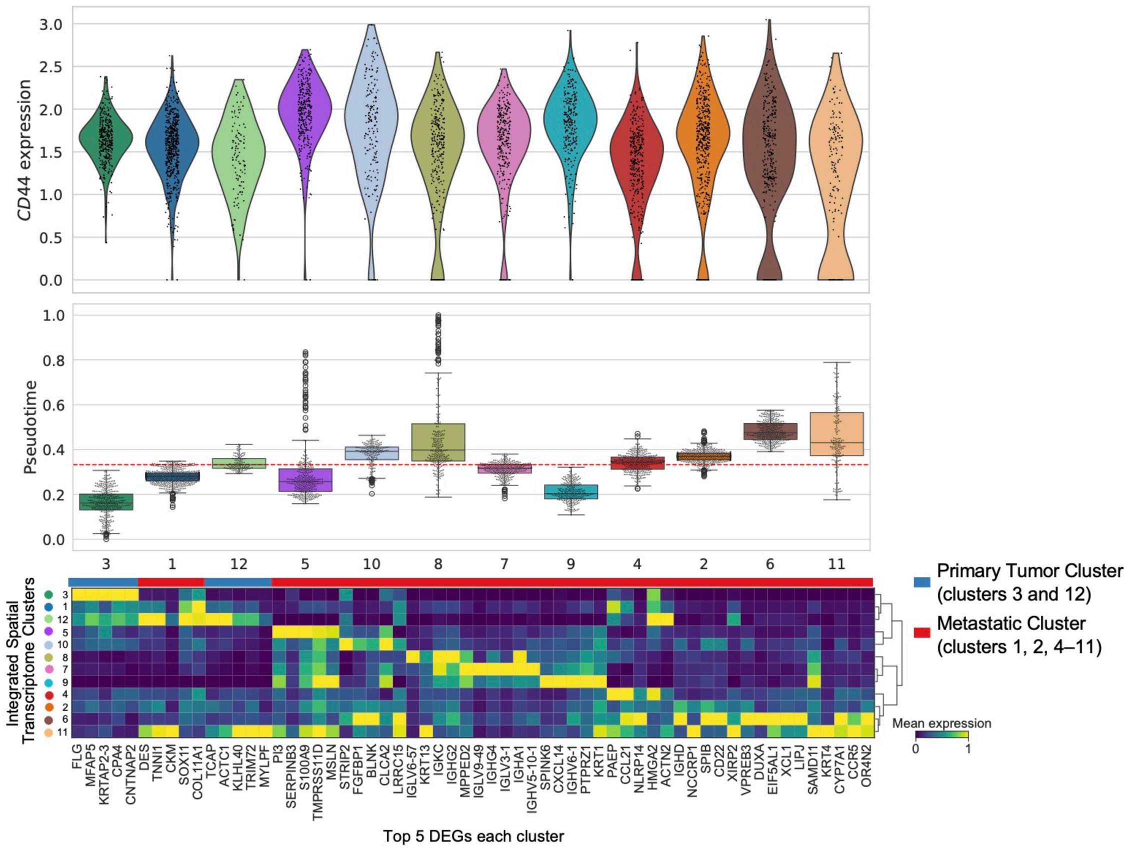

Differential gene expression analysis revealed unique molecular signatures for each Metastatic Cluster (**Letter Table L7 [new Supporting Table S11]**): Cluster 1 showed enrichment of myCAF-like program, including Desmin (*DES*), Troponin I1 *(TNNI1)*, and Collagen Type XI Alpha 1 Chain *(COL11A1)*. Cluster 5 was characterized by elevated Serpin Family B Member 3 (*SERPINB3)*, S100 Calcium Binding Protein A9 *(S100A9)*, and Mesothelin *(MSLN)* expression, indicating high invasive tumor cells. Cluster 7 demonstrated high expression of markers class-switched immunoglobulins**,** notably Immunoglobulin Heavy Constant Gamma 4 **(***IGHG4),* Immunoglobulin Heavy Constant Alpha 1 *(IGHA1),* and Immunoglobulin Lambda Variable 3-1 *(IGLV3-1)*. Cluster 9 was distinguished by markers typically associated with CXCL14^{+} basal-like epithelial cells*, including* C-X-C Motif Chemokine Ligand 14 *(CXCL14),* Serine Peptidase Inhibitor Kazal Type 6 (*SPINK6), and* Keratin 1 *(KRT1).* Of particular note, Cluster 1, which had the highest proportion of cells from primary tumor sites among Metastatic Clusters, showed enrichment of myCAFs and activated *COL11A1*, suggesting the molecular cross talk though the ECM-CD44 axis. These analyses provide spatial context for the heterogeneous states and microenvironmental interactions associated with the transition towards a metastatic phenotype at the invasive front. We have incorporated these findings and corresponding figures and tables into the revised Results section (refer to Figure 5A-5C for cluster locations, and new Supporting Figure s and Tables for pseudotime analysis results, cluster characteristics, and DEGs).

Letter Table L7 (new Supporting Table S11)

Cluster	Top DEGs each Integrated Spatial Transcriptome Cluster					Spatial transcriptome-based cluster name	Cluster classification	Reference
1	DES	TNNI1	CKM	SOX11	COL11A1	Contractile myCAFs with OSCC	Metastatic	[84,85]
2	IGHD	NCCRP1	SPIB	CD22	XIRP2	Naïve B cell with OSCC	Metastatic	[86,87]
3	FLG	MFAP5	KRTAP2-3	CPA4	CNTNAP2	Keratin-high OSCC	Primary Tumor	[88]
4	PAEP	CCL21	NLRP14	HMGA2	ACTN2	CCL21-driven migratory OSCC	Metastatic	[88–92]
5	PI3	SERPINB3	S100A9	TMPRSS11D	MSLN	SERPINB3-high invasiveOSCC	Metastatic	[93–96]
6	VPREB3	DUXA	EIF5AL1	XCL1	LIPJ	Pre-/GC-Bcell with OSCC	Metastatic	[97]
7	IGLV9-49	IGHG4	IGLV3-1	IGHA1	IGHV5-10-1	Mature plasma cell with OSCC	Metastatic	[98]
8	IGLV6-57	KRT13	IGKC	IGHG2	MPPED2	Mature plasma cell with OSCC	Metastatic	[98]
9	SPINK6	CXCL14	IGHV6-1	PTPRZ1	KRT1	CXCL14^{+} Basal-like OSCC	Metastatic	[99,100]
10	STRIP2	FGFBP1	BLNK	CLCA2	LRRC15	LRRC15^{+} CAFs with OSCC	Metastatic	[101,102]
11	SAMD11	KRT4	CYP7A1	CCR5	OR4N2	CCR5-high mucosal-type OSCC	Metastatic	[103]
12	TCAP	ACTC1	KLHL40	TRIM72	MYLPF	Myogenic OSCC	Primary Tumor	[104]

UPDATED RESULTS: To explore tumor clusters associated with LNM, we conducted pseudotime trajectory analysis using stLearn, rooted in the Primary Tumor Cluster 3. Metastatic Clusters 1, 5, 7, and 9 exhibited shorter pseudotime compared to Primary Tumor Cluster 12, with elevated *CD44* expression (all adj $P < 0.001$) indicating enhanced stemness. DEGs analysis revealed unique molecular signatures for these spatial clusters. Cluster 1 showed enrichment of myCAF-like program, including Desmin (*DES)*, Troponin I1 (*TNNI1)*, and Collagen Type XI Alpha 1 Chain (*COL11A1*). Cluster 5 was characterized by elevated Serpin Family B Member 3 *(SERPINB3),* S100 Calcium Binding Protein A9 (*S100A9*), and Mesothelin (*MSLN*) expression, indicating high invasive tumor cells. Cluster 7 demonstrated high expression of markers class-switched immunoglobulins, notably Immunoglobulin Heavy Constant Gamma 4 (*IGHG4*), Immunoglobulin Heavy Constant Alpha 1 (*IGHA1)*, and Immunoglobulin Lambda Variable 3-1 (*IGLV3-1*). Cluster 9 was distinguished by markers typically associated with $CXCL14^{+}$ basal-like epithelial cells, including C-X-C Motif Chemokine Ligand 14 (*CXCL14*), Serine Peptidase Inhibitor Kazal Type 6 (*SPINK6*), and Keratin 1 (*KRT1)*. Interestingly, Cluster 1, which had the highest proportion of cells from primary tumor sites among Metastatic Clusters, showed enrichment myCAFs and activated COL11A1, suggesting the molecular cross talk though the ECM-CD44 axis. These data depict a continuum of spatial states at the ITF, reflecting migratory, inflammatory, and stem-like characteristics potentially underpinning LNM in OSCC. (Refer to **Supporting Table S11** for full list of DEGs and cluster characteristics).

Revised text: **Results, Supplementary Methods section ("Spatial pseudotime trajectory analyses"** subsection**).**
Revised Figures: **Figure 7A-7C; Supporting Figure S4F.**
New Tables: **Supporting Table S10** and **Supporting Table S11**.

2. The discussion section should be expanded to reflect all the results obtained in the study. For instance, there is a lack of discussion regarding whole-exome sequencing and bulk transcriptome sequencing data from TCGA project.

RE: Thank you for your valuable suggestion to expand the discussion section to comprehensively reflect all results, specifically including the whole-exome sequencing (WES) and bulk transcriptome sequencing data from the TCGA project. We agree that integrating these findings is essential for a complete understanding of our study.

We have revised the Discussion section to incorporate the results obtained from the TCGA data analyses. We discuss how the lack of significant association between major driver mutations and LNM status in the WES data suggests that genetic events alone may not fully explain metastatic potential in OSCC. We also integrate the finding from the bulk TCGA RNA-seq data regarding the enrichment of a muscle-contraction transcriptional program in LNM-positive samples, interpreting this as an indicator of increased stromal activation potentially contributing to invasion and metastasis, independent of specific genetic drivers.

By including these analyses, our discussion provides a more complete picture, highlighting the potential importance of non-mutational and microenvironment-driven mechanisms in LNM development, complementary to genetic alterations. The Discussion section has been updated accordingly to reflect these important aspects.

UPDATE DISCUSSION: Using publicly available multiomics data and original spatial transcriptomic data, we studied the role of myCAFs in promoting LNM in OSCC. For analyses using HNSCC TCGA data, which includes various head and neck sub-sites, we applied strict inclusion criteria to define a homogeneous OSCC cohort. We focused on cases annotated as OSCC by site and confirmed HPV-negative status. While HPV is a critical prognostic factor in HNSCC, particularly in oropharyngeal tumors, its prevalence and prognostic impact are considerably lower (<10%) and limited in OSCC. Accurately classifying cases and avoiding confounding is challenging in HNSCC cohorts due to anatomical ambiguity (e.g., tongue base or tonsil region) and distinct HPV-positive tumor features. Therefore, filtering by both specific oral cavity site annotation and HPV negativity was crucial. This created a homogeneous HPV-negative OSCC subtype, including cases from relevant boundary regions whose molecular profiles align with primary oral cavity tumors, thereby enhancing study validity. Analysis of this cohort revealed several initial insights. We found no significant association between key driver mutations and LNM status, suggesting that genetic events alone may not fully explain LNM potential. Instead, bulk RNA-seq analysis highlighted a muscle-contraction transcriptional program enriched in LNM-positive samples, indicating increased stromal activation. We also found a quantitative and functional activation of myCAFs in the LNM-positive microenvironment. In LNM-positive samples, myCAFs spatially colocalized with OSCC cells within the ITF, indicating their active role in promoting LNM. The myCAFs also sent ECM-related signals to OSCC cells, which activated key stemness-related genes in the OSCC cells through ECM-related receptors such as CD44. Furthermore, by analyzing the genes specifically expressed in the ITF, where myCAFs and OSCCs colocalize and where metastasis arises, we identified a 23-gene signature that was significantly associated with LNM status and poor OS in patients with OSCC.

Revised text: **Discussion**

Reviewer #3: This study represented by Furudate et al. integrates in-house spatial transcriptomic data, and third party bulk transcriptomic, single-cell RNA-seq data, and dissects the role of myCAFs in OSCC progression. The spatial colocalization of myCAFs and OSCC cells at the invasive tumor front (ITF) and their crosstalk via ECM-related signaling and CD44 stemness pathway offer fresh insights into LNM pathogenesis. The identification of a 23-gene signature with prognostic value is clinically significant. Advanced computational tools, such as CellChat, CIBERSORTx, Tangram, and validation via IHC in 50 additional samples strengthen the conclusions. The survival analyses in TCGA and external cohorts enhance reproducibility. However, several issues require clarification or elaboration to strengthen the manuscript.

RE: We very much appreciate the reviewer's appreciation of our study and positive comments.

Key Concerns:

1. The spatial analysis relies on only 2 patients (1 LNM-positive, 1 LNM-negative). While IHC validation in 50 samples mitigates this, expanding spatial transcriptomics to a larger cohort (at least 3-5 pairs) would improve statistical power and generalizability.

RE: We thank the reviewer for the insightful comment. In response to the recommendation, we have significantly expanded our spatial transcriptome analysis cohort from the original 4 samples (2 patients) to 16 samples with OSCC. Specifically, we leveraged a publicly available OSCC spatial transcriptomic dataset (GSE208253; Arora et al[105]) consisting of 12 Visium spatial transcriptome samples (6 LNM-positive and 6 LNM-negative, **Letter Table 8**［**new Supporting Table S7**］).

We first applied Tangram[1,2,83] for precise cell-type deconvolution, and identified tumor-specific regions where OSCC cells were detected following the methodology described by Arora et al[105] (ITFs and central tumor regions, **Letter Figure L5A-B** [**new Supporting Figure S4K-4N**]). Subsequent analyses of this expanded dataset consistently demonstrated that myCAFs preferentially localized at the invasive tumor fronts (ITFs) in LNM-positive samples, confirming our original findings regarding spatial colocalization and ECM-driven signaling pathways **(Letter Figure L5C** [**new Supporting Figure S4O**]). The expanded validation supported our spatially-resolved 23-gene signature, confirming its significant association with LNM status in patients with OSCC ($P < 0.001$, 95% CI = 0.76-1.45, **new Figure 9G**).

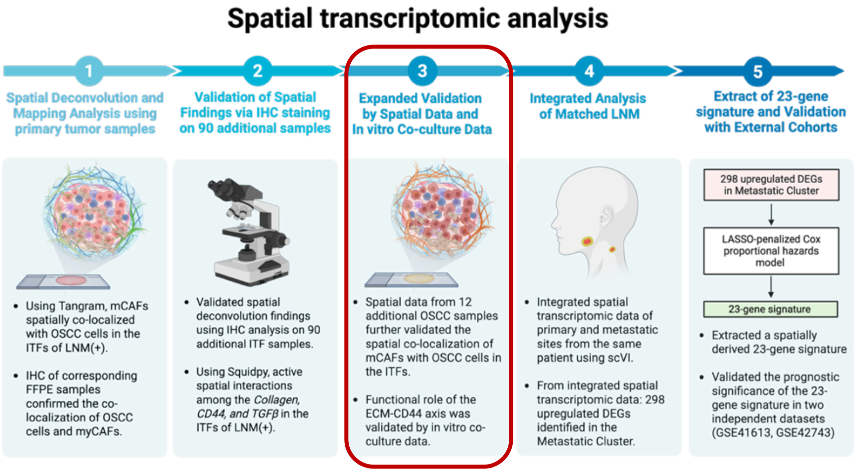

Letter Table 8 (new Supporting Table S7.) Characteristics of 12 samples with OSCC derived from validation spatial transcriptome data.

**Sample ID**	**Patient ID**	**Age**	**Pathologic T stage**	**Pathologic N stage**	**Tissue origin**	**Primary tumor site**	**Grade**
**S1**	P1	50-59	T2	N2	Tongue	Anterior	2
**S2**	P2	50-59	T4	N1	Floor of mouth	Posterior floor of mouth	2
**S3**	P3	50-59	T1	N0	Tongue	Left lateral tongue	1
**S4**	P4	30-39	T2	N2	Tongue	Left lateral tongue	1
**S5**	P5	60-69	T2	N0	Tongue	Right lateral tongue	2
**S6**	P6	40-49	T2	N0	Tongue	Left lateral tongue	2
**S7**	P7	70-79	T4	N0	Tongue	Left retromolar tongue	3
**S8**	P7	70-79	T4	N0	Tongue	Left lateral tongue	3
**S9**	P8	50-59	T4	N1	Tongue	Left lateral tongue	2
**S10**	P8	50-59	T4	N1	Floor of mouth	Anterior floor of mouth	2
**S11**	P9	60-69	T2	N0	Floor of mouth	Floor of mouth	2
**S12**	P10	60-69	T2	N2	Floor of mouth	Floor of mouth	2

Sample ID, Patient ID, age, pathologic T stage, pathologic N stage, tissue origin, primary tumor site, and grade were obtained from a previous study[105]

Letter Figure L5A (new Supporting Figure S4K-4L)

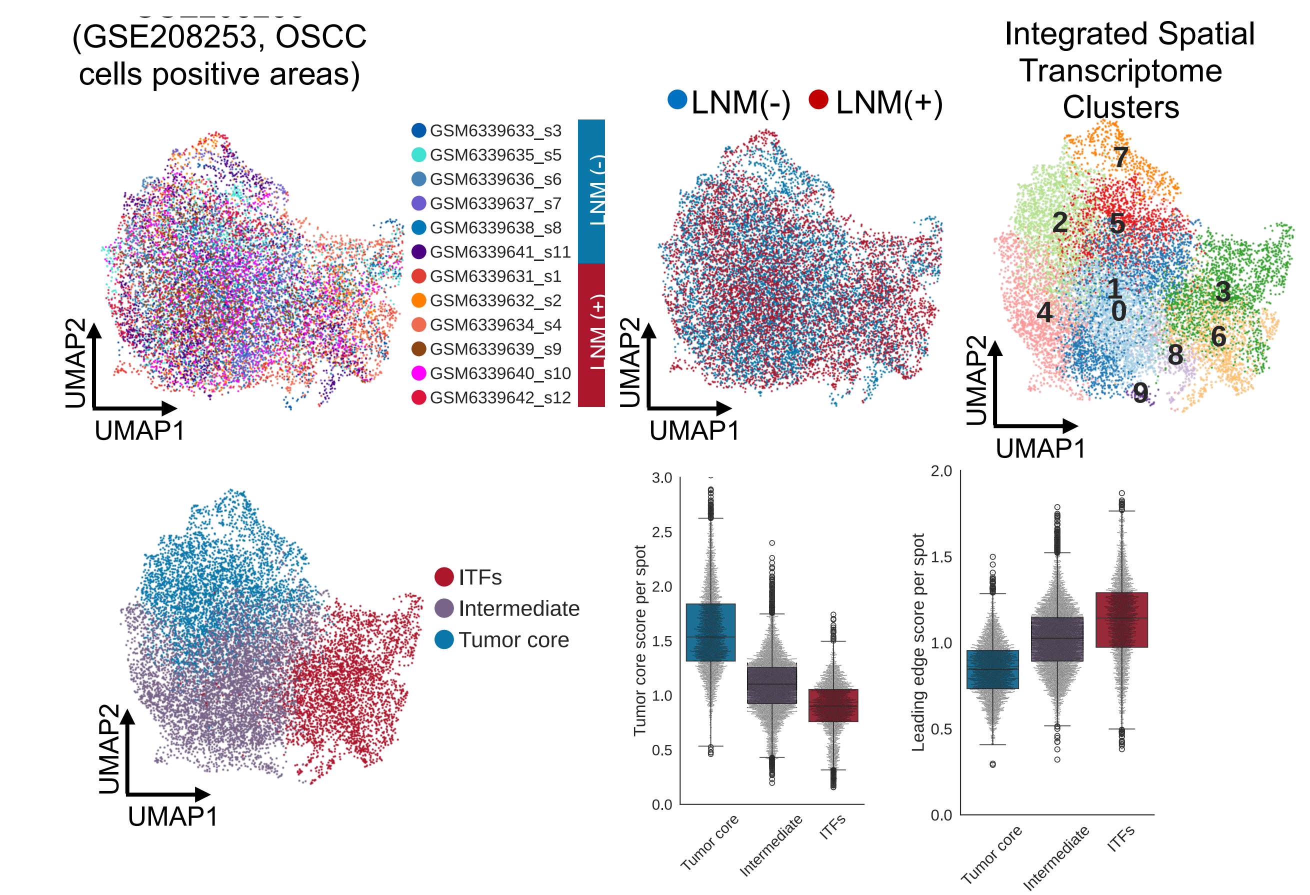

Letter Figure L5B (new Supporting Figure 4M)

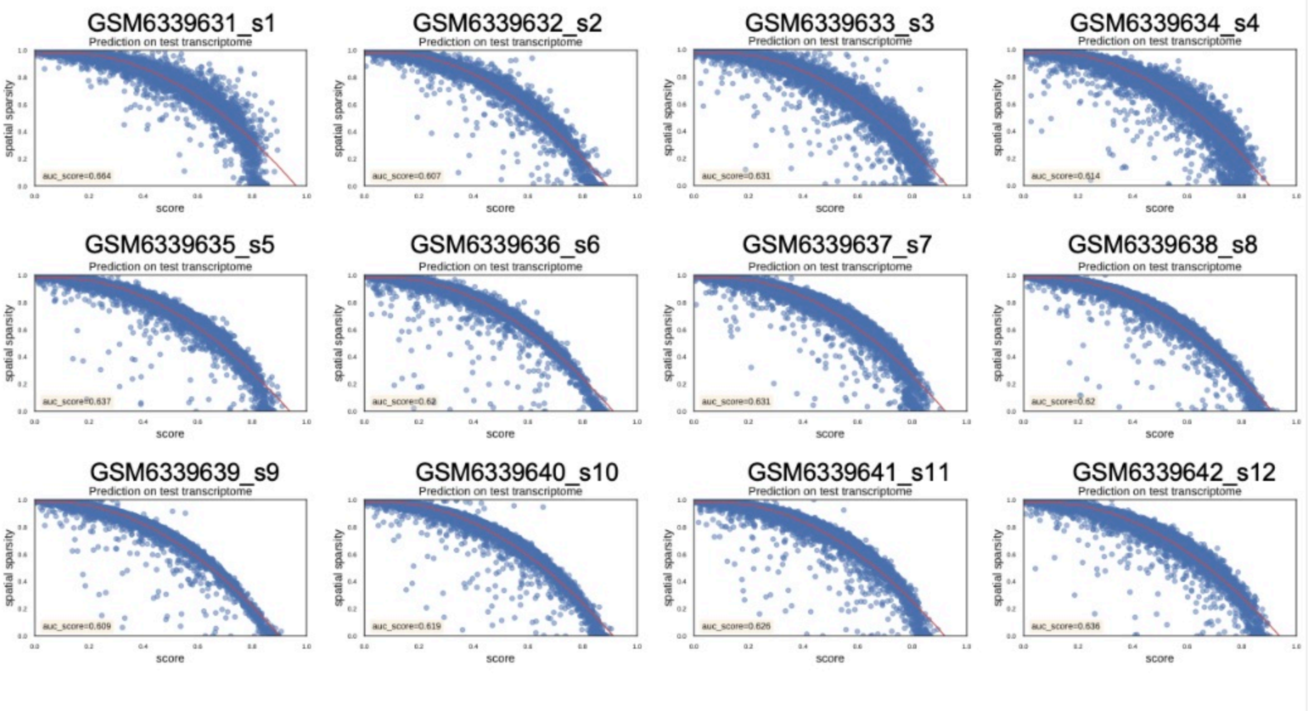

Letter Figure L5B (continue; new Supporting Figure S4M-N)

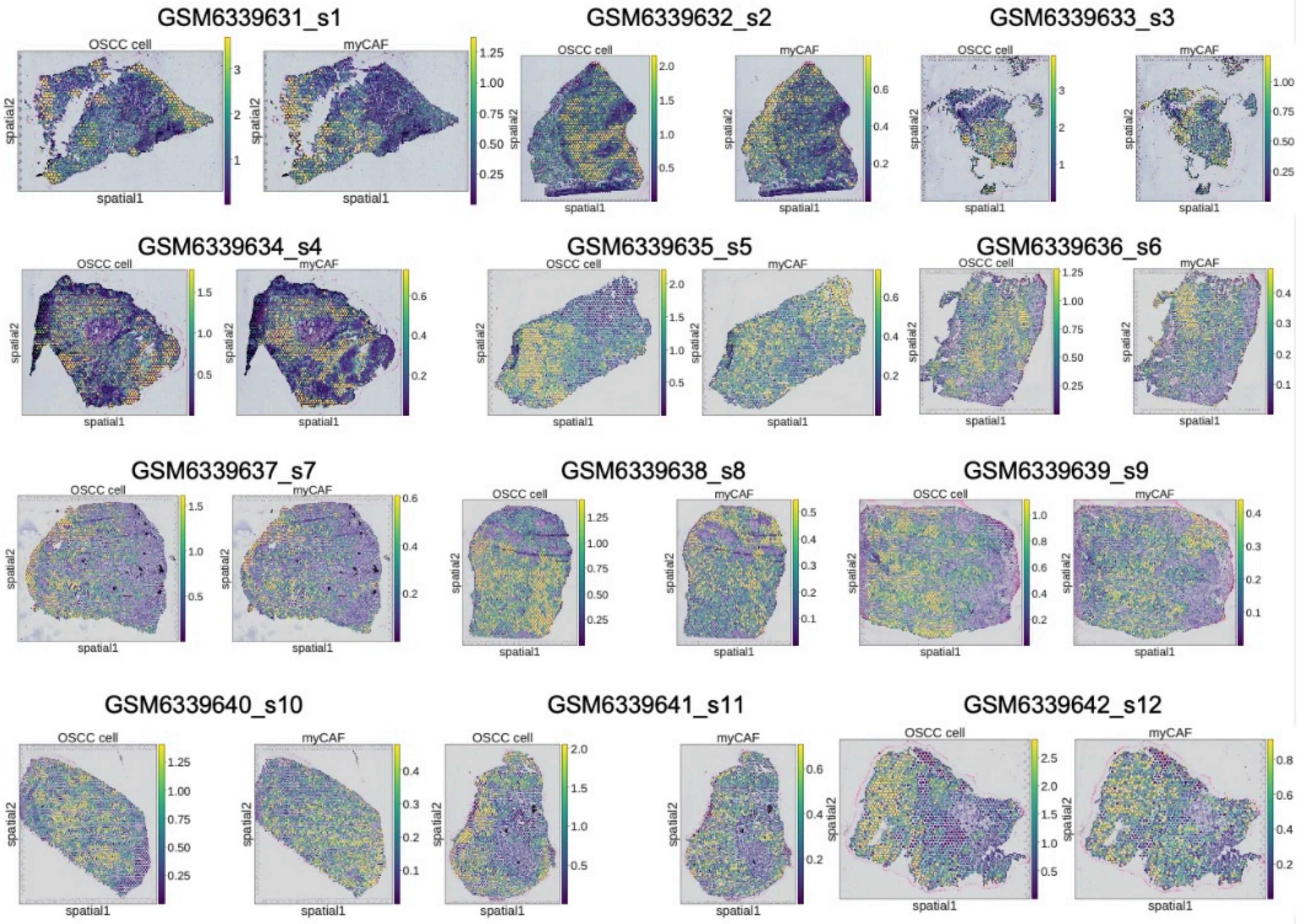

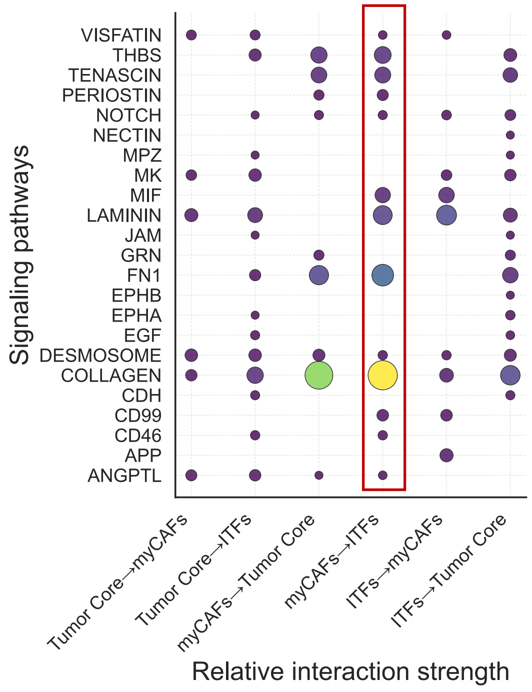

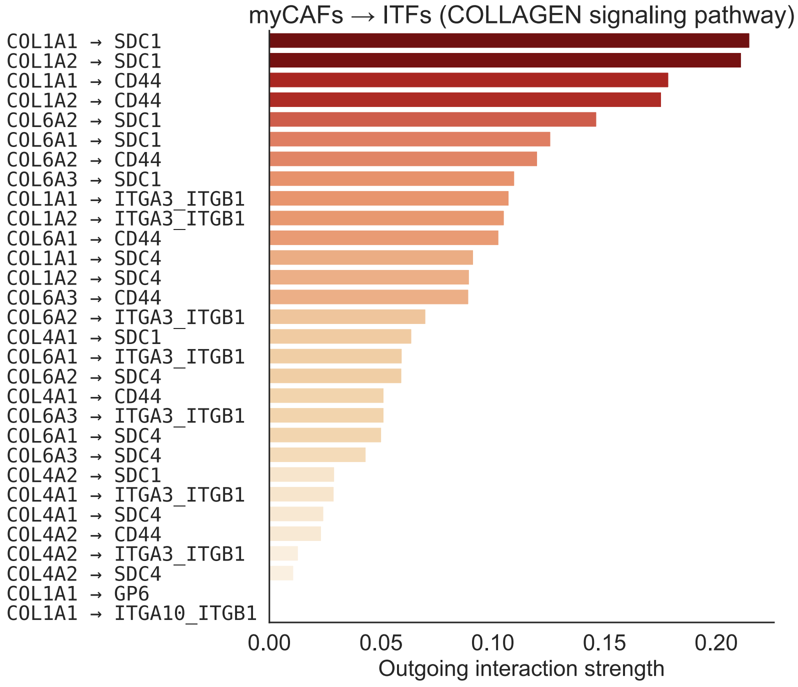

Letter Figure L5C (new Supporting Figure S4O; Figure 6G; new Supporting Figure S5K)

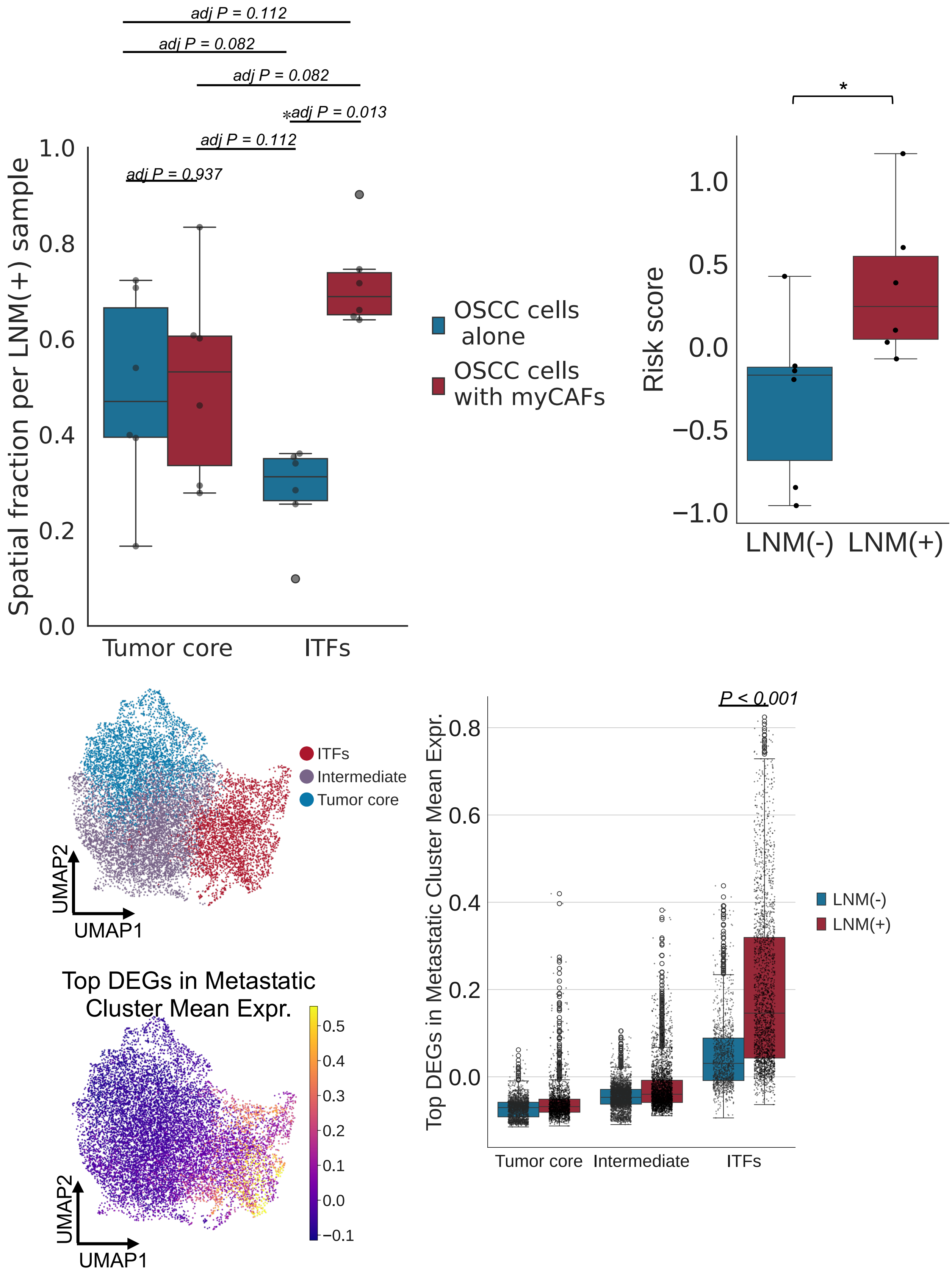

Additionally, we have increased the number of samples validated by immunohistochemistry (IHC) from 50 to 90, further enhancing the robustness and generalizability of our findings (**Letter Figure L5D [updated Figure 7A-7B]; Letter Table L9 [updated Supporting Table S4**). Collectively, these extensive analyses reinforce our conclusion that myCAFs and associated ECM signaling pathways are determinants of LNM in patients with OSCC, substantially improving both statistical power and clinical relevance.

Letter Figure L5D (updated Figure 7A-7B)

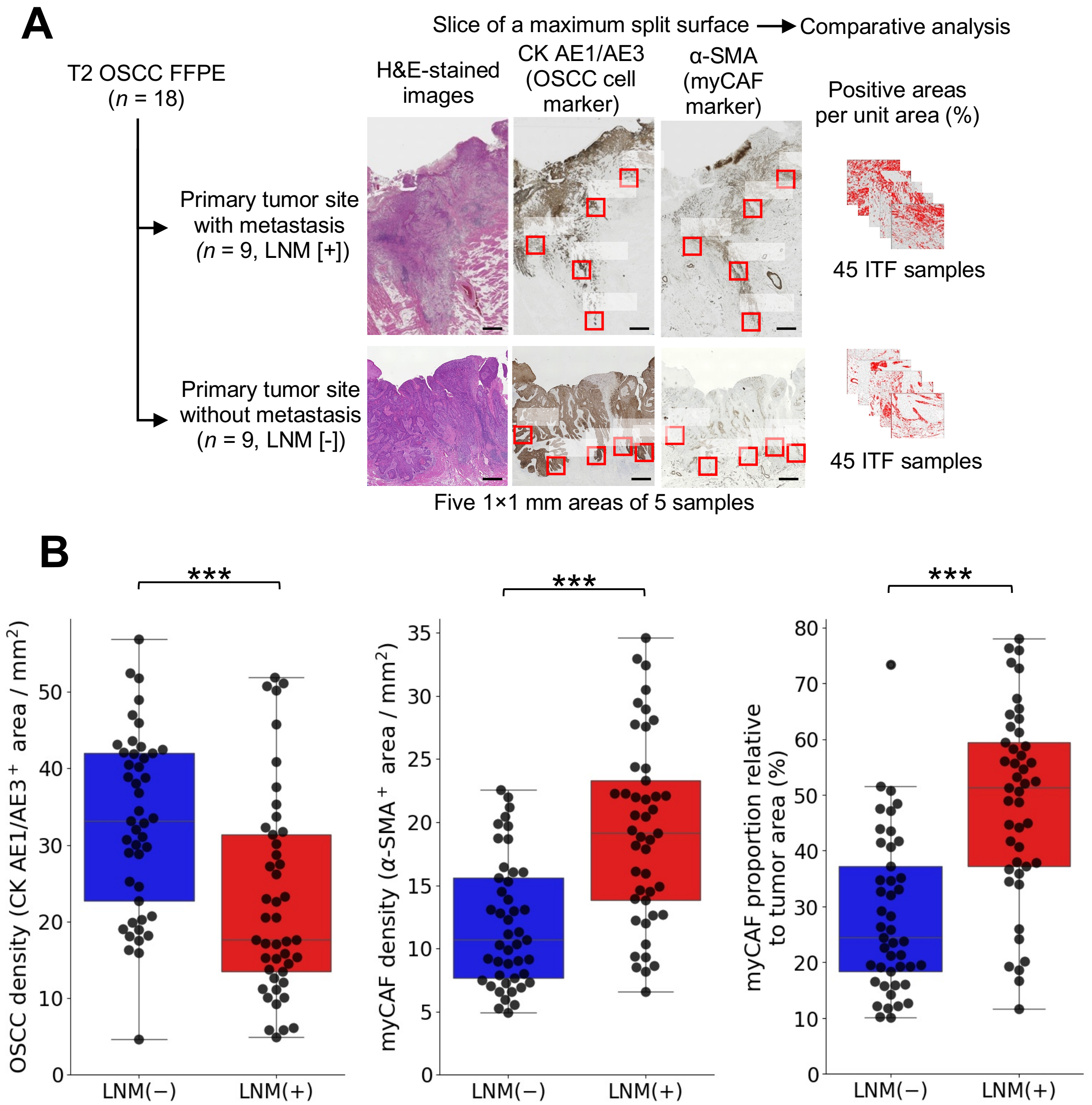

Letter Table L9 (Supporting Table S4.) Characteristics of 20 patients with OSCC from a clinical cohort, Related to Methods

Patient ID	Age	Sex	Pathologic T stage	Pathologic N stage	Tissue origin	Primary tumor site	Grade	POI
**HUH001**	48	F	T2	N3b	Tongue	Left lateral tongue	2	4
**HUH002**	61	F	T2	N0	Tongue	Right lateral tongue	2	3
**HUH003**	64	M	T2	N2	Tongue	Right lateral tongue	3	4
**HUH004**	80	F	T2	N1	Tongue	Left lateral tongue	2	4
**HUH005**	78	M	T2	N1	Tongue	Left lateral tongue	1	3
**HUH006**	45	M	T2	N1	Tongue	Right lateral tongue	2	4
**HUH007**	57	F	T2	N1	Tongue	Left lateral tongue	1	4
**HUH008**	68	F	T2	N0	Tongue	Left lateral tongue	2	4
**HUH009**	58	M	T2	N0	Tongue	Left lateral tongue	1	2
**HUH010**	59	M	T2	N0	Tongue	Base of tongue	2	2
**HUH011**	64	M	T2	N0	Tongue	Right lateral tongue	2	4
**HUH012**	34	M	T2	N0	Tongue	Left lateral tongue	2	4
**HUH013**	46	M	T2	N1	Tongue	Left lateral tongue	3	5
**HUH014**	79	M	T2	N1	Gum	Left alveolar ridge	3	3
**HUH015**	57	M	T2	N1	Cheek mucosa	Left buccal mucosa	2	4
**HUH016**	58	M	T2	N1	Tongue	Left lateral tongue	2	3
**HUH017**	63	F	T2	N0	Tongue	Left lateral tongue	1	3
**HUH018**	56	M	T2	N0	Tongue	Left lateral tongue	2	2
**HUH019**	71	M	T2	N0	Tongue	Left lateral tongue	1	3
**HUH020**	79	F	T2	N0	Tongue	Left lateral tongue	2	2

UPDATE RESULTS: Next, we examined the ligand-receptor pairs (myCAFs and OSCC cells) in the spatial transcriptome data using the scRNA-seq data described above. The LNM-positive samples (HUH001-P1 and HUH001-P2) showed enhanced spatial gene expression of inferred receptors such as ITGA, ITGB, CD44, and SDC in OSCC cells within the ITFs (Figure S4H-J). A further spatial interaction analysis confirmed that these LNM-positive samples exhibited increased spatially organized communication compared with the LNM-negative sample (HUH002-P); the identified signaling pathways involved the collagen gene family, CD44, and transforming growth factor β (TGFβ; Figure S4K).

To validate the proposed myCAF-OSCC cell interactions via the ECM-CD44 axis, we analyzed additional publicly available 12 spatial transcriptomic OSCC samples (source: GSE208253[25]) and in vitro co-culture datasets (sources: GSE279481[26]; GSE178153/GSE178154[27]). The validation spatial cohort consistently showed a spatially organized ECM-CD44 axis. In LNM-positive samples, this reflected active collagen signaling from myCAFs to OSCC within ITFs (Figures S4L-N). This occurred alongside enhanced myCAF-OSCC colocalization ($P = 0.01$) and significantly higher spatial *CD44* expression in LNM-positive samples ($P = 0.02$; Figures S4O-P).

UPDATE DISCUSSION: A limitation of this study was the small sample size in the spatial transcriptomic analysis. To overcome this, we validated our findings regarding the spatial colocalization of myCAFs and OSCC cells using publicly available 12 more spatial transcriptomic OSCC samples and 90 additional IHC samples (see Table S4 and S7 for details). These analyses confirmed a significant association between increased myCAF density at the ITFs and LNM. Building on this, multivariable analysis of these 90 IHC samples confirmed myCAF density as an independent LNM predictor, even when controlling for pathological grade and POI. Since myCAF density itself did not significantly correlate with them, it offers distinct information for predicting LNM beyond these established clinicopathological parameters. Supportive In vitro data from other studies also provided mechanistic support for our observed ECM-CD44 axis. Additionally, the prognostic significance of the 23-gene signature that we extracted from the spatial analysis was validated in separate clinical TCGA cohorts and 2 other microarray datasets. We believe that these efforts successfully addressed the limitations regarding the small sample size in the spatial transcriptomic analysis.

Revised text: **Result**, **Discussion**, **Methods** section ("**IHC analysis of 90 ITF samples from T2 OSCC patients**" and "**Statistics and reproducibility**" subsection), and Supplementary Methods section ("**Spatial validation cohort processing**" subsection).
Revised Figures: **Figure 7A-7C**; **Figure 6G**; **Supporting Figure S4K-4O**, and **Supporting Figure S5K**.
Revised Table: **Supporting Table S4** and **Supporting Table S7**.

2. The proposed ECM-CD44 axis driving stemness in OSCC cells is compelling but requires functional validation. In vitro co-culture experiments (myCAFs + OSCC cells) or in vivo models like xenografts with myCAF depletion could confirm causality. As the DEGs are derived from LNM different samples, it might be better to establish a LNM model in vitro or in vivo and further validate their findings.

RE: We appreciate reviewer's insightful suggestion regarding functional validation of theproposed ECM–CD44 axis driving stemness in OSCC cells. While direct in vitro and in vivo experimental validations are critical (e.g., myCAF depletion in xenograft models), these experiments lie beyond the current scope of our manuscript. Instead, we conducted additional supportive analyses using publicly available co-cultured datasets (GSE279481; Waas et al[106] and GSE178153/GSE178154; Wiechec et al[107]). GSE279481 is the bulk RNA-seq dataset of tongue squamous cell carcinoma cells (Tongue SCC, Cal33 and Cal27) and cancer-associated fibroblast cells (CAFs, 61137 and 61162) in mono-culture or co-culture (**Letter Table L10 [new Supporting Table S8]**).

Letter Table L10 (new Supporting Table S8)

geo accession	Sample	cell line	cell type	treatment
GSE279481	GSM8573232	Cal33	Tongue SCC	Control
GSE279481	GSM8573233	61137	CAFs	Control
GSE279481	GSM8573234	Cal33	Tongue SCC	Co-cultured with 61137
GSE279481	GSM8573235	61137	CAFs	Co-cultured with Cal33
GSE279481	GSM8573236	Cal33	Tongue SCC	Control
GSE279481	GSM8573237	61162	CAFs	Control
GSE279481	GSM8573238	Cal33	Tongue SCC	Co-cultured with 61162
GSE279481	GSM8573239	61162	CAFs	Co-cultured with Cal33
GSE279481	GSM8573240	Cal27	Tongue SCC	Control
GSE279481	GSM8573241	61137	CAFs	Control
GSE279481	GSM8573242	61162	CAFs	Control
GSE279481	GSM8573243	Cal27	Tongue SCC	Co-cultured with 61137
GSE279481	GSM8573244	61137	CAFs	Co-cultured with Cal27
GSE279481	GSM8573245	Cal27	Tongue SCC	Co-cultured with 61137
GSE279481	GSM8573246	61162	CAFs	Co-cultured with Cal27

We first confirmed that 61137 and 61162 expressed the key myCAF markers (ACTA2, TANGLN, MYL9, TPM2, MCAM). Then, we confirmed no difference in COL1A1 expression between mono-cultured and co-cultured (**Letter Figure L6A [new Supporting Figure 4Q]**).

Letter Figure L6A (new Supporting Figure S4Q)

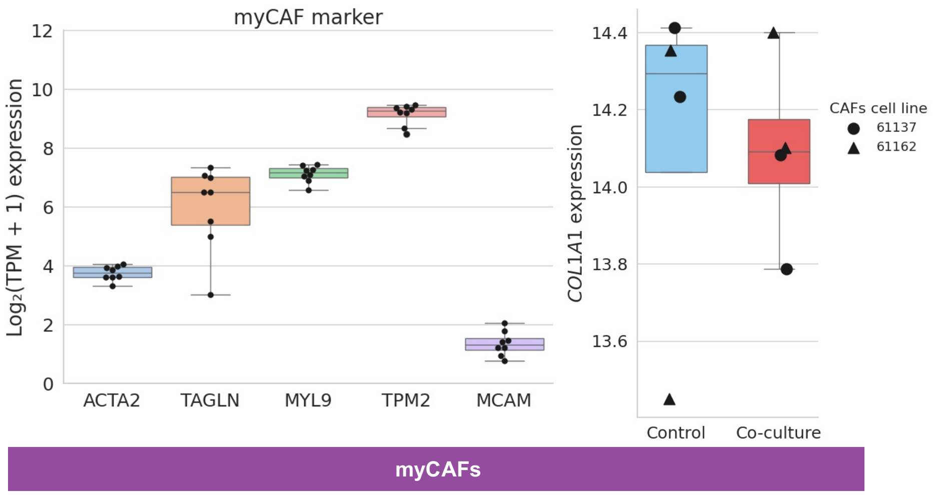

Although the sample size was limited, we observed a higher median *CD44* expression in OSCC cells co-cultured with myCAFs compared to monoculture (**Letter Figure L6B [new Supporting Figure S4Q]**; median [interquartile range (IQR)] in monoculture: 3.78 [3.64–4.76] vs. co-culture: 5.18 [4.59–5.74]). However, the difference was not statistically significant ($P = 0.40$, two sided Mann–Whitney U test), and should be interpreted with caution.

Letter Figure L6B (new Supporting Figure S4Q)

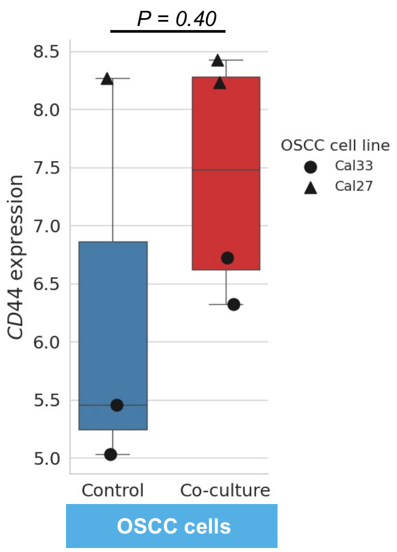

To further refine our selection of collagen-related genes relevant to myCAF–OSCC communication, we initially extracted all genes beginning with "COL" (n = 56) from the expression matrix. We then retained genes with mean expression greater than 1.0 ($\log_2$[TPM+1]) across myCAF samples (combining monoculture and co-culture conditions), resulting in 23 expressed genes. We focused specifically on genes implicated in ECM organization and basement membrane formation. Based on the previous reports[106,108–120], we selected 17 ECM core genes for detailed analysis, including fibrillar collagens (*COL1A1, COL3A1*) and basement membrane components (*COL4A1, COL4A5, LAMA3, LAMB1*).

To investigate the relationship between expression changes of collagen genes in myCAFs and *CD44* expression changes in OSCC cells under co-culture conditions, we aggregated gene expression data by condition, computed $\log_2$ fold-changes for collagen genes in myCAFs and *CD44* in OSCC cells, merged the data, and calculated gene-wise Pearson correlations, visualizing the results using scatter plots. Among the 17 genes analyzed, *COL6A2* and *LAMB1* showed strong positive correlations with *CD44* in co-cultured OSCC cells (r = 0.92, *P* = 0.077; r = 0.93, *P* = 0.068), while *LAMA3* showed a strong negative correlation (r = -0.98, *P* = 0.024, **Letter Figure L6C [new Supporting Figure 4Q]**). These findings suggest that while most ECM genes showed a positive correlation with *CD44* expression in co-cultured OSCC cells, some collagen genes, notably *LAMA3*, showed a negative correlation, highlighting subtype-specific roles.

Letter Figure L6C (new Supporting Figure S4Q)

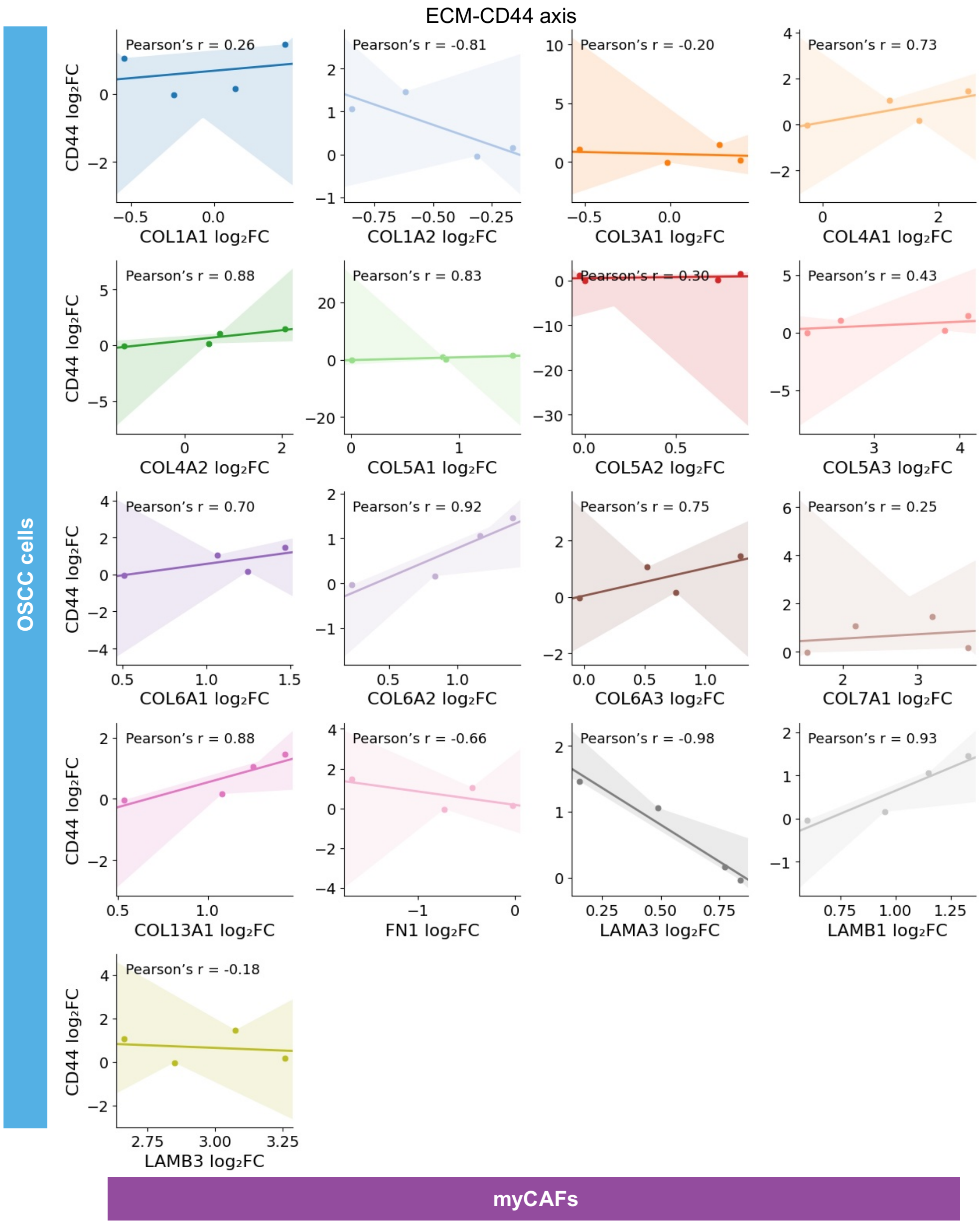

To further investigate the overall relationship between myCAF-derived ECM gene expression and CD44 levels in OSCC cells, we applied hierarchical Bayesian modeling. The advantage of using a hierarchical Bayesian model is that it enables stable estimation even with small datasets, properly accounts for variability across genes and pairs, and provides a unified framework to evaluate both overall trends and individual differences. This approach allowed us to simultaneously estimate the global effect across all genes as well as gene- and pair-specific variations, addressing the limited sample size and inherent variability in the data. Moreover, the hierarchical Bayesian modeling revealed a global positive association between ECM gene expression and CD44 levels (posterior slope mean: 0.27, 95% HDI: 0.01–0.63), supporting the potential biological relevance of the ECM–CD44 axis (**Letter Figure L6D [new Supporting Figure S4R]**).). is positive, individual ECM genes exhibit diverse effects, emphasizing the importance of considering subtype-specific roles.

Letter Figure L6D (new Supporting Figure S4R)

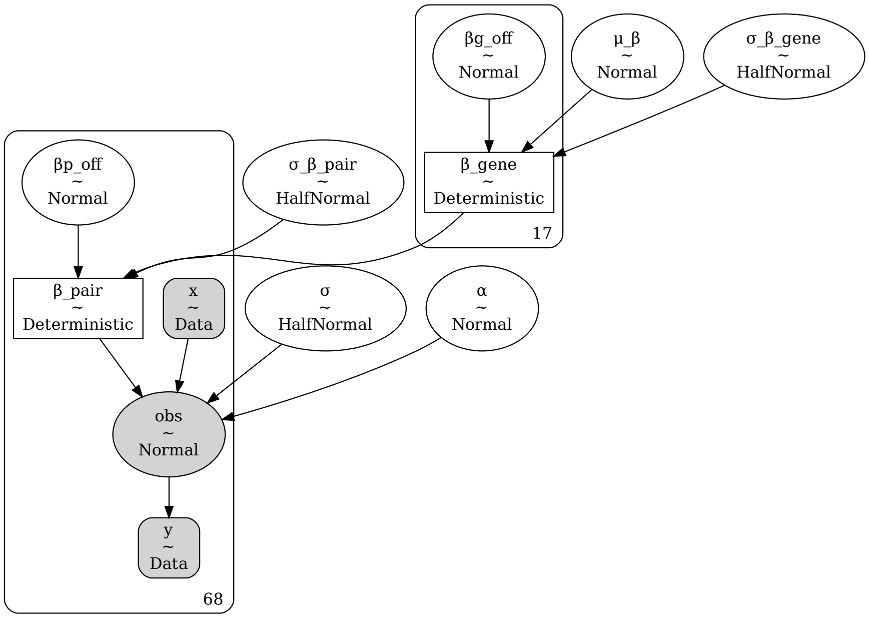

Symbol	Description
x	Standardized ECM gene expression (myCAFs)
y	Standardized *CD44* expression (OSCC cells)
μ_β	Population-level mean of slopes
σ_β_gene	SD of gene-level slope variation
β_gene	Gene-specific slope coefficient
σ_β_pair	SD of pair-specific slope variation
β_pair	Pair-specific slope coefficient
α	Intercept
σ	Residual SD
obs	Observed response variable

Letter Figure L6D (new Supporting Figure S4R)

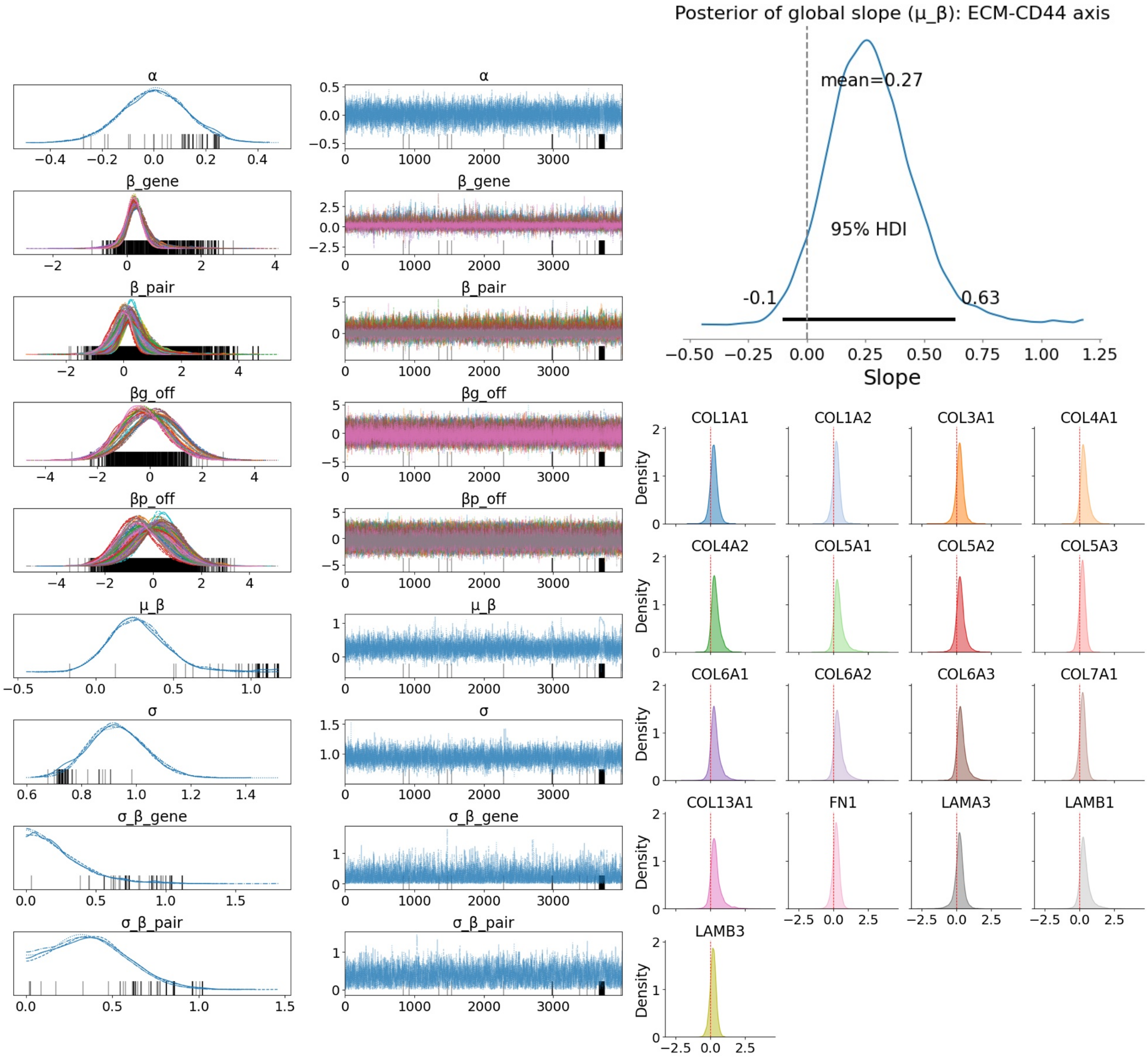

In addition to the global relationship, the model provided gene-specific slope estimates. Notably, several collagen genes, such as COL6A2 (mean: 0.381) and LAMB1 (mean: 0.357), showed relatively strong positive slopes, consistent with the overall trend. By contrast, genes like LAMA3 (mean: 0.177) and LAMB3 (mean: 0.175) had lower mean slopes and wide 95% HDI intervals crossing zero (e.g., LAMA3: -0.550 to 0.889), suggesting substantial uncertainty and potential gene-specific variability. Heatmap showed heterogeneity across experimental pairs, with some genes like COL5A3, FN1, LAMA3, and LAMB3 displaying notable variation across conditions (**Letter Figure L6E [new Supporting Figure 4R]**). Taken together, these results highlight that while the overall ECM–CD44 relationship.

Letter Figure L6E (new Supporting Figure S4R)

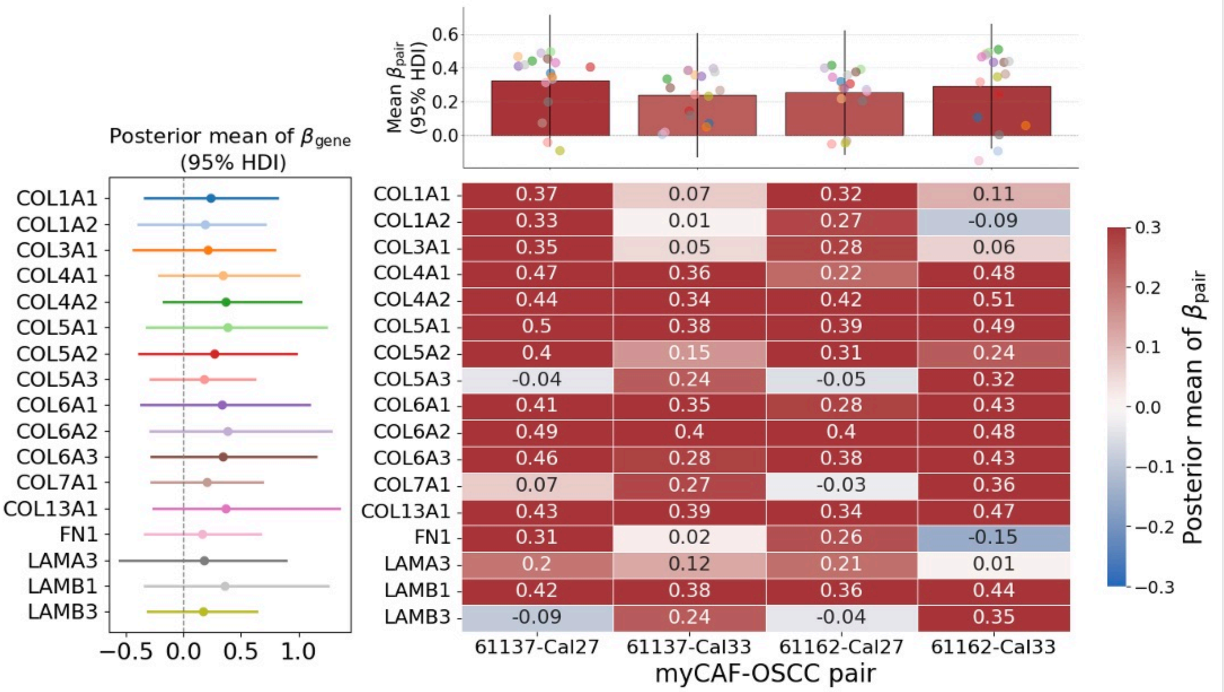

Our scRNA cell–cell communication and spatial interaction analyses revealed potential interactions between myCAF-derived extracellular matrix (ECM) signaling and OSCC cell receptors, particularly integrins (ITGAs, ITGBs) and syndecans (SDC1, SDC4). Based on these observations, we aimed to investigate how myCAF-derived ECM gene expression influences CD44 expression in OSCC cells through these mediators. Using principal component analysis (PCA) and ordinary least squares (OLS) regression, we found that the integrin-mediated pathway exhibited a moderate indirect effect ($\beta = 0.29$), while the syndecan-mediated pathway showed a stronger effect ($\beta = 0.47$), suggesting that both receptor families play important roles in ECM-driven regulation of CD44. Specifically, the integrin pathway (z1) showed a significant effect of collagen on ITGA/ITGB expression ($\beta_{xz} = 0.35$, $p = 0.004$) and a strong effect of ITGA/ITGB on CD44 ($\beta_{zy} = 0.85$, $p < 0.001$), yielding an indirect effect of 0.29. The syndecan pathway (z2) demonstrated an even stronger effect of collagen on SDC expression ($\beta_{xz} = 0.53$, $p < 0.001$) and SDC on CD44 ($\beta_{zy} = 0.88$, $p < 0.001$), with an indirect effect of 0.47(**Letter Figure L6F [new Supporting Figure S4S]**). Further, mediation analysis employing bootstrap sampling (n = 10,000) confirmed these findings, indicating significant indirect effects of ECM genes on CD44 expression in OSCC cells through both ITGA/ITGB and SDC pathways. Specifically, the indirect effect through ITGAs/ITGBs (pathway 1) was $\beta = 0.29$ (95% CI: 0.17–0.40), while the indirect effect via SDCs (pathway 2) was even stronger, at $\beta = 0.47$ (95% CI: 0.34–0.57; **Letter Figure L6F [new Supporting Figure S4S**). These findings statistically and biologically support our proposed model, suggesting that ECM-related signals from myCAFs may enhance OSCC stemness through cell adhesion molecules.

Letter Figure L6F (new Supporting Figure S4S)

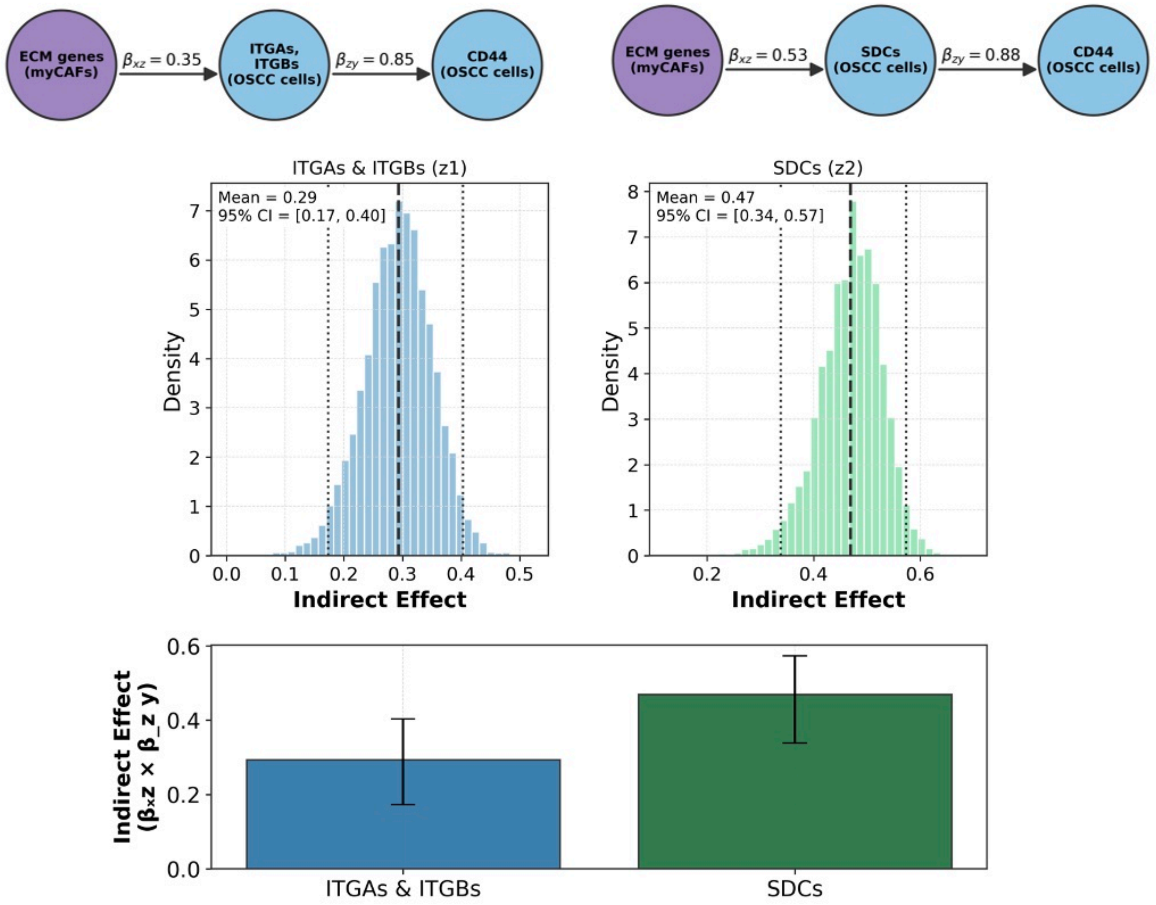

To validate our findings, we next analyzed an independent microarray dataset of head and neck squamous cell carcinoma (HNSCC) (GSE178153, GSE178154). In this dataset, patient-derived HNSCC cell lines (LK0824, LK0858, LK0923, LK0942, LK0949, LK0902, LK0917, LK1108) were cultured either alone (control) or co-cultured with cancer-associated fibroblasts (CAFs) under 2D (GSE178153) and 3D (GSE178154) conditions (**Letter Table L11 [new Supporting Table 9]**). This allowed us to assess the impact of CAFs on ECM-related gene expression and *CD44* levels in an independent experimental system.

Letter Table L11 (new Supporting Table 9)

GSE178153 and GSE178154
(HNSCC cell lines were co-cultured with their patient-matched CAFs)

geo_accession	sample	Title	tissue	age	gender	cell_line	experiment	location	treatment	stage	LNM
GSE178153	GSM5381030	LK0824celllinegrownwithoutCAFin2D	HNSCC	74	M	LK0824	2D	tongue	Control	T2N1M0	1
GSE178153	GSM5381031	LK0858celllinegrownwithoutCAFin2D	HNSCC	38	F	LK0858	2D	tongue	Control	T3N0M0	0
GSE178153	GSM5381032	LK0923celllinegrownwithoutCAFin2D	HNSCC	79	F	LK0923	2D	larynx	Control	T1N0M0	0
GSE178153	GSM5381033	LK0942celllinegrownwithoutCAFin2D	HNSCC	71	M	LK0942	2D	larynx	Control	T4N0M0	0
GSE178153	GSM5381034	LK0949celllinegrownwithoutCAFin2D	HNSCC	62	M	LK0949	2D	tongue	Control	T2N0M0	0
GSE178153	GSM5381035	LK0824celllinegrownwithCAFin2D	HNSCC	74	M	LK0824	2D	tongue	Co-culturedwithCAF	T2N1M0	1
GSE178153	GSM5381036	LK0858celllinegrownwithCAFin2D	HNSCC	38	F	LK0858	2D	tongue	Co-culturedwithCAF	T3N0M0	0
GSE178153	GSM5381037	LK0923celllinegrownwithCAFin2D	HNSCC	79	F	LK0923	2D	larynx	Co-culturedwithCAF	T1N0M0	0
GSE178153	GSM5381038	LK0942celllinegrownwithCAFin2D	HNSCC	71	M	LK0942	2D	larynx	Co-culturedwithCAF	T4N0M0	0
GSE178153	GSM5381039	LK0949celllinegrownwithCAFin2D	HNSCC	62	M	LK0949	2D	tongue	Co-culturedwithCAF	T2N0M0	0
GSE178154	GSM5381040	LK0902celllinegrownwithoutCAFin3D	HNSCC	68	F	LK0902	3D	tongue	Control	T1N0M0	0
GSE178154	GSM5381041	LK0917celllinegrownwithoutCAFin3D	HNSCC	79	M	LK0917	3D	gingiva	Control	T4N1M1	1
GSE178154	GSM5381042	LK1108celllinegrownwithoutCAFin3D	HNSCC	65	F	LK1108	3D	hypopharynx	Control	T2N0M0	0
GSE178154	GSM5381043	LK0902celllinegrownwithCAFin3D	HNSCC	68	F	LK0902	3D	tongue	Co-culturedwithCAF	T1N0M0	0
GSE178154	GSM5381044	LK0917celllinegrownwithCAFin3D	HNSCC	79	M	LK0917	3D	gingiva	Co-culturedwithCAF	T4N1M1	1
GSE178154	GSM5381045	LK1108celllinegrownwithCAFin3D	HNSCC	65	F	LK1108	3D	hypopharynx	Co-culturedwithCAF	T2N0M0	0

To compare the expression of *CD44* in patient-derived HNSCC cells (GSE178153/GSE178154), we first processed the microarray CEL files by applying Robust Multi-Array Average (RMA) normalization and adding gene annotations using the databases (huex10sttranscriptcluster.db and hta20transcriptcluster.db, respectively). We then removed low-variance genes (bottom 20%), corrected batch effects with pyComBat[34,35], and applied Z-score standardization for each gene. We next observed significantly higher CD44 levels in LNM-positive samples compared to LNM-negative samples *(P* = 0.042; median for LNM (-) = -0.373 [IQR, -1.002–0.360] vs. median for LNM (+) = 0.747 [IQR, 0.590–0.999]). Additionally, while the overall comparison between monoculture and co-culture conditions in patient-derived HNSCC cells did not reach statistical significance (*P* = 0.721; median for monoculture = 0.151 [IQR, -0.923–0.690] vs. median for co-culture = 0.359 [IQR, -0.783–0.670]), subgroup analysis revealed distinct patterns (**Letter Figure L6G [new Supporting Figure S4T]**). Specifically, in Tongue SCC cells derived from LNM-positive patients, CD44 expression was already elevated under monoculture conditions and remained largely unchanged upon co-culture with CAFs. This may suggest that, in vivo, the tumor microenvironment of LNM-positive patients was already mimicking conditions similar to co-culture, although this possibility should be interpreted with caution.

Letter Figure L6G (new Supporting Figure S4T)

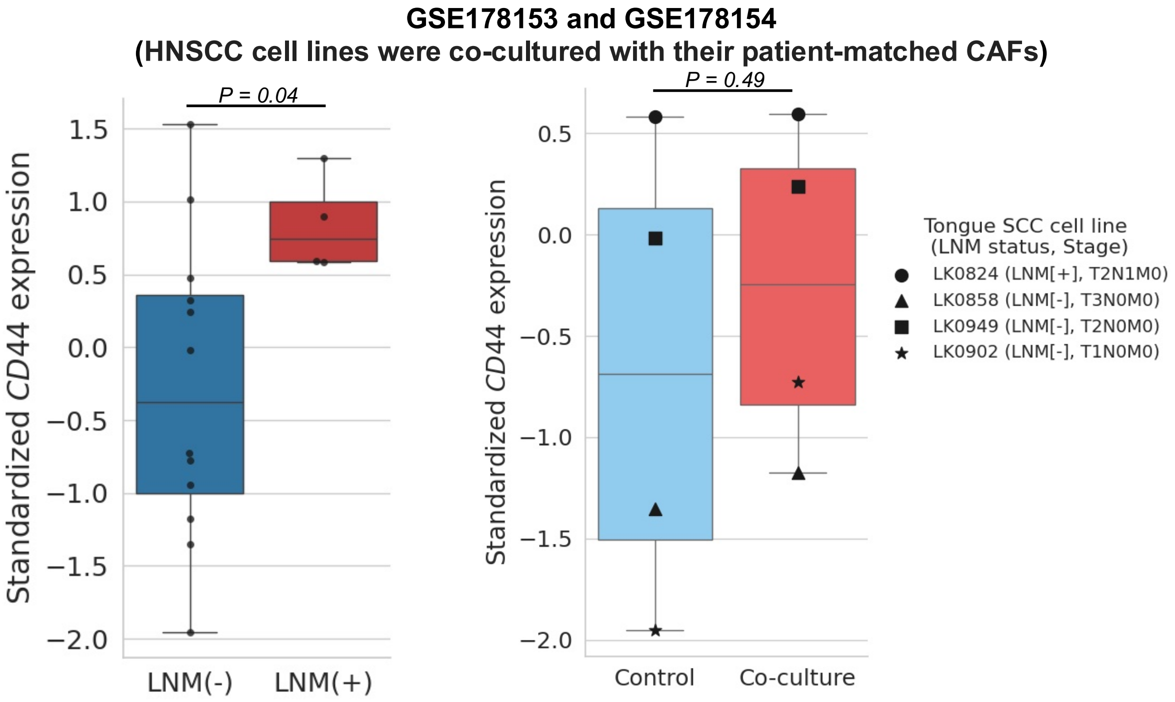

Furthermore, using 12 additional spatial transcriptomic cohort, we confirmed that ECM signaling was most active in myCAFs→ITFs and that *CD44* expression was higher in LNM (+) samples (*P*<0.001, 95% CI= −0.16-−0.13, **Letter Figure L6H [new Supplymental Figure S4N-P]**).

Letter Figure L6H (new Supplymental Figure S4N-P)

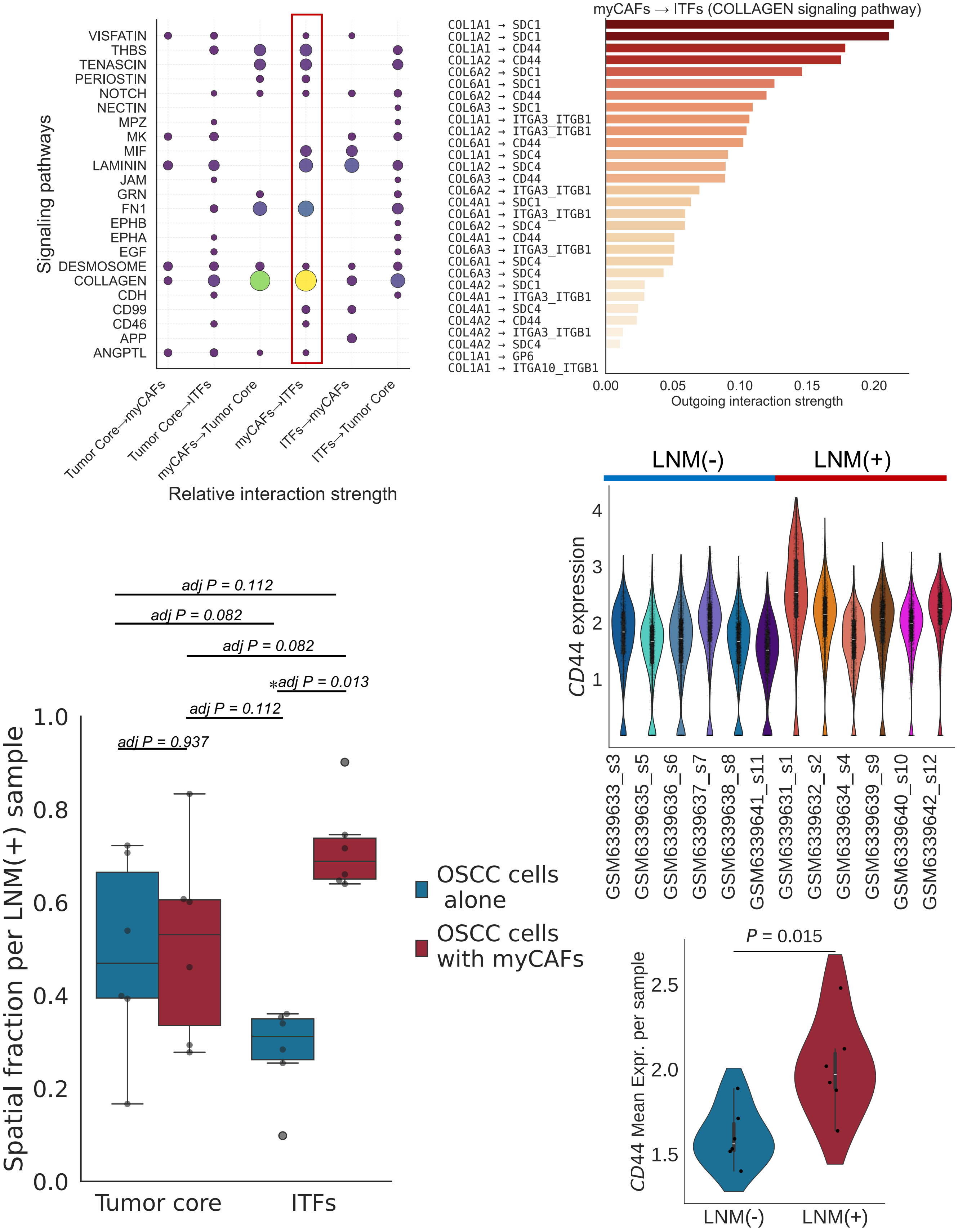

To confirm the proposed ECM-CD44 axis driving stemness in OSCC cells, we also added CD44 expression and pseudotime trajectory analysis to our Visium dataset with stLearn, which integrates spatial gene expression, tissue morphology, and physical distance with a graph-based approach[83]. Our analysis revealed that Metastatic Clusters exhibited significantly longer pseudotime compared to Primary Tumor Clusters (median: 0.34 [95% bootstrap CI, 0.33-0.34] vs 0.19 [95% bootstrap CI, 0.17-0.20], respectively; *P* <0.001), suggesting an advanced developmental state in Metastatic Clusters (**Letter Figure L6I [new Supporting Figure S5F-S5G]**). Because tumor progress towards the ITFs, we set Cluster 3 (Primary Tumor Cluster) as the trajectory root because the trajectory root lies in a Primary Tumor Cluster.

Letter Figure L6I (new Supporting Figure S5F-S5G)

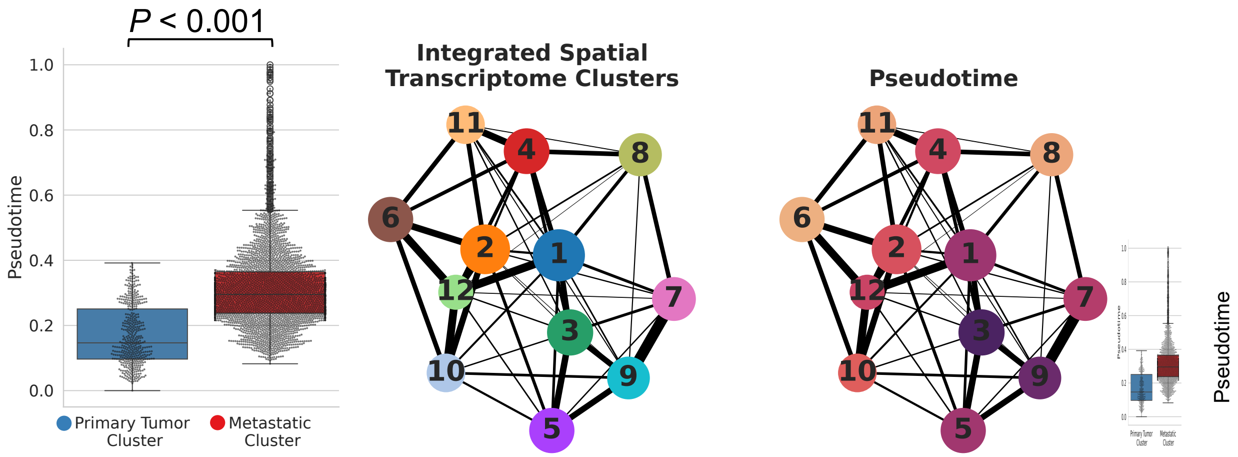

Further comparison of pseudotime across individual clusters highlighted specific Metastatic Clusters (Clusters 1, 5, 7, and 9) that demonstrated shorter pseudotime than primary tumor cluster 12, coupled with elevated *CD44* expression (**Letter Table L12 [new Supporting Table S10]** and **Letter Figure L6J [new Supporting Figure S5H]**). These findings indicate increased stemness characteristics in these metastatic clusters.

Letter Table L12 (new Supporting Table S10)

Integrated Spatial Transcriptome Cluster	CD44 expression					Pseudotime		
	median	25th percentile	75th percentile	P	adj P	median	25th percentile	75th percentile
1	1.59	1.34	1.78	0.008	0.012	0.28	0.26	0.30
2	1.67	1.32	1.95	<0.001	<0.001	0.37	0.35	0.38
3	1.65	1.51	1.79	<0.001	<0.001	0.16	0.13	0.20
4	1.44	1.13	1.67	0.307	0.338	0.34	0.31	0.37
5	1.99	1.76	2.20	<0.001	<0.001	0.26	0.21	0.31
6	1.50	1.12	1.90	0.571	0.571	0.48	0.45	0.52
7	1.67	1.42	1.92	<0.001	<0.001	0.32	0.30	0.33

8	1.61	1.24	1.87	0.052	0.063	0.40	0.35	0.52
9	1.86	1.62	2.09	<0.001	<0.001	0.20	0.18	0.24
10	1.87	1.44	2.20	<0.001	<0.001	0.39	0.35	0.41
11	1.34	0.00	1.72	0.013	0.018	0.43	0.37	0.57
12	1.46	1.17	1.71			0.33	0.32	0.36

Letter Figure L6J (new Supporting Figure S5H)

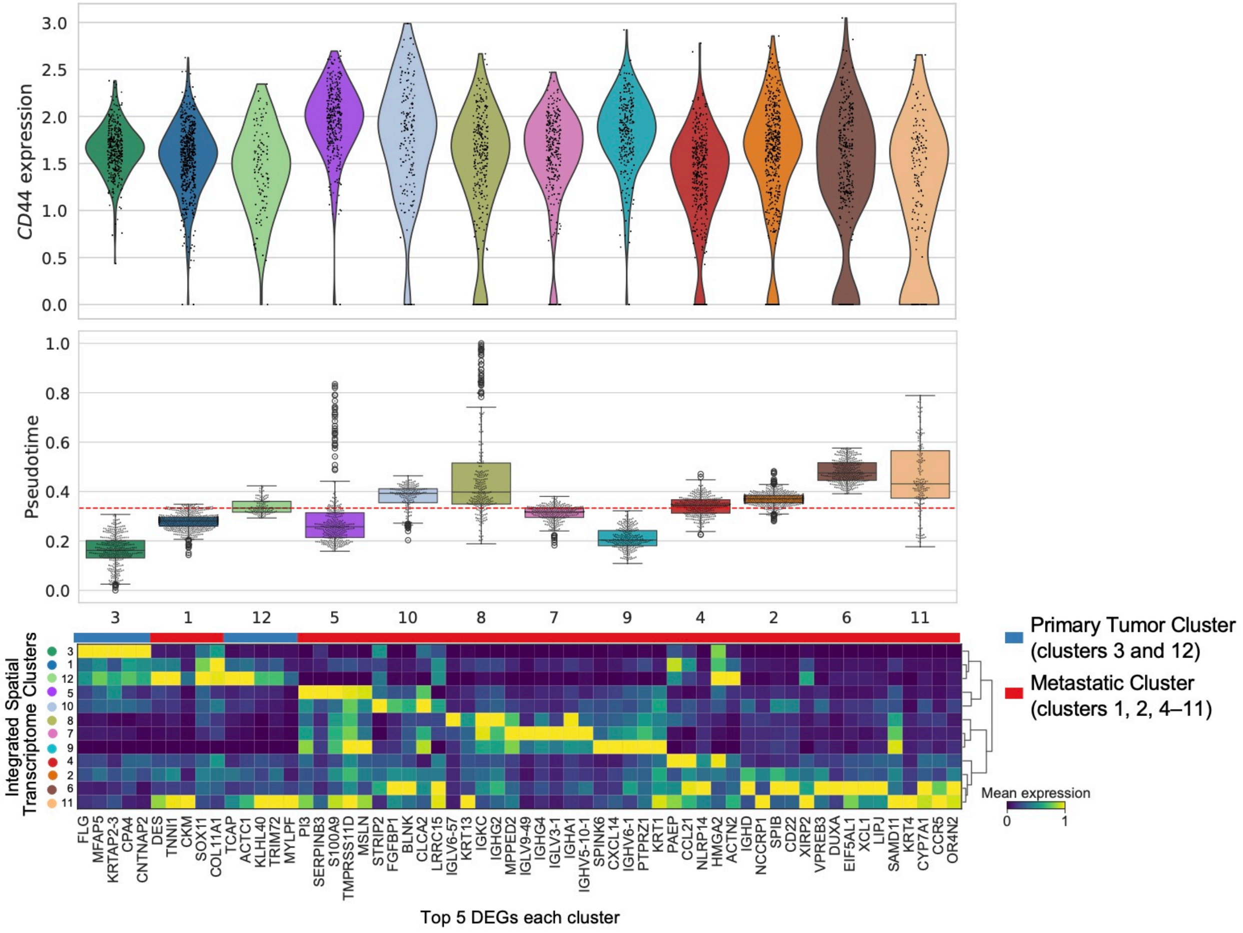

Differential gene expression analysis revealed unique molecular signatures for each Metastatic Cluster (**Letter Table L13 [new Supporting Table S11]** and **Letter Figure L6J**): Cluster 1 showed enrichment of myCAF-like program, including Desmin (*DES*), Troponin I1 *(TNNI1)*, and Collagen Type XI Alpha 1 Chain *(COL11A1)*. Cluster 5 was characterized by elevated Serpin Family B Member 3 (*SERPINB3*), S100 Calcium Binding Protein A9 *(S100A9)*, and Mesothelin *(MSLN)* expression, indicating high invasive tumor cells. Cluster 7 demonstrated high expression of markers class-switched immunoglobulins**,** notably Immunoglobulin Heavy Constant Gamma 4 **(***IGHG4*), Immunoglobulin Heavy Constant Alpha 1 *(IGHA1)*, and Immunoglobulin Lambda Variable 3-1 *(IGLV3-1)*. Cluster 9 was distinguished by markers typically associated with $CXCL14^{+}$ basal-like epithelial cells*, including* C-X-C Motif Chemokine Ligand 14 *(CXCL14)*, Serine Peptidase Inhibitor Kazal Type 6 (*SPINK6*)*, and* Keratin 1 *(KRT1)*.

Of particular note, Cluster 1, which had the highest proportion of cells from primary tumor sites among Metastatic Clusters, showed enrichment myCAFs and activated *COL11A1*, suggesting the molecular cross talk though the ECM-CD44 axis. These data depict a continuum from the Primary Tumor Site to highly
migratory, inflammatory, and stem-like states that underpin LNM in OSCC.

Letter Table L13 (new Supporting Table S11)

Cluster	Top DEGs each Integrated Spatial Transcriptome Cluster					Spatial transcriptome-based cluster name	Cluster classification	Reference
1	DES	TNNI1	CKM	SOX11	COL11A1	Contractile myCAFs with OSCC	Metastatic	[84,85]
2	IGHD	NCCRP1	SPIB	CD22	XIRP2	Naïve B cell with OSCC	Metastatic	[86,87]
3	FLG	MFAP5	KRTAP2-3	CPA4	CNTNAP2	Keratin-high OSCC	Primary Tumor	[88]
4	PAEP	CCL21	NLRP14	HMGA2	ACTN2	CCL21-driven migratory OSCC	Metastatic	[88–92]
5	PI3	SERPINB3	S100A9	TMPRSS11D	MSLN	SERPINB3-high invasiveOSCC	Metastatic	[93–96]
6	VPREB3	DUXA	EIF5AL1	XCL1	LIPJ	Pre-/GC-Bcell with OSCC	Metastatic	[97]
7	IGLV9-49	IGHG4	IGLV3-1	IGHA1	IGHV5-10-1	Mature plasma cell with OSCC	Metastatic	[98]
8	IGLV6-57	KRT13	IGKC	IGHG2	MPPED2	Mature plasma cell with OSCC	Metastatic	[98]
9	SPINK6	CXCL14	IGHV6-1	PTPRZ1	KRT1	CXCL14$^+$ Basal-like OSCC	Metastatic	[99,100]
10	STRIP2	FGFBP1	BLNK	CLCA2	LRRC15	LRRC15$^+$ CAFs with OSCC	Metastatic	[101,102]
11	SAMD11	KRT4	CYP7A1	CCR5	OR4N2	CCR5-high mucosal-type OSCC	Metastatic	[103]
12	TCAP	ACTC1	KLHL40	TRIM72	MYLPF	Myogenic OSCC	Primary Tumor	[104]

We acknowledge that our results alone do not definitively demonstrate causality. Therefore, in the Discussion, we explicitly referenced robust functional studies, such as Choi, JH.et al[108], who demonstrated that COL1A-enriched ECM directly promotes CD44-dependent stemness and tumorigenicity in head and neck squamous cell carcinoma. Such evidence aligns closely with our spatial and transcriptomic findings.

UPDATE RESULTS: Next, we examined the ligand-receptor pairs (myCAFs and OSCC cells) in the spatial To validate these findings and provide further support for the biological plausibility of the myCAF-OSCC cell interactions and the proposed ECM-CD44 axis, we expanded our spatial analysis to include twelve additional publicly available OSCC spatial transcriptome samples (source: GSE208253) and conducted supportive analyses using publicly available in vitro co-culture data (source: GSE279481 and GSE178153/178154). In the expanded spatial cohort, we consistently observed spatially organized ECM-CD44 axis. In LNM-positive samples, myCAFs expressed higher levels of ECM-related genes, including the collagen gene family

(COL1A1-A2, COL6A1-A3), and the ITFs regions upregulated ECM-associated receptors, specifically SDCs, CD44, ITGAs, and ITGBs. Consistent with our observations, we validated enhanced spatial colocalization of myCAFs and OSCC cells at the ITFs in LNM-positive samples (Supporting Figure SXX). Consistent with this, spatial CD44 expression was significantly higher in LNM positive compared to LNM negative samples (P<0.001) (Letter Figure L6H). Analyses of publicly available in vitro co-culture datasets (GSE279481 and GSE178153/GSE178154) further supported the proposed interaction. While direct assessment of overall *CD44* upregulation upon CAF co-culture showed variability and did not consistently reach statistical significance across individual datasets, mechanistic modeling using this co-culture data provided stronger evidence, suggesting significant indirect effects of myCAF-related ECM on OSCC cell CD44 expression potentially mediated via integrin (β=0.29, 95% CI 0.17–0.40) and syndecan receptors (β=0.47, 95% CI 0.34–0.57) (Letter Figure L6A-6F, L10, L11). Furthermore, analysis of the independent microarray co-culture dataset (GSE178153/4) revealed that patient-derived HNSCC lines from LNM-positive tumors exhibited significantly higher CD44 expression compared to those from LNM-negative tumors (P=0.042), supporting the association between elevated *CD44* and LNM status in primary tumor cells (Letter Figure L6G). These findings confirm the intercellular interaction data from the scRNA-seq analysis and provide evidence that these interactions are driven by the spatial colocalization of myCAFs and OSCC cells within the ITFs.

Revised text: **Abstract**, **Result,** and **Supplementary Methods** section ("**Spatial validation cohort processing**", "**Tongue SCC co-culture RNAseq data processing**", "**Hierarchical Bayesian Modeling of ECM-CD44 axis**", "**Mediation analysis of ECM-CD44 axis via Integrin and Syndecan pathways**", and "**Microarray data processing**" subsection).
Revised Figures: **Figure1A, Figure 3A, Supporting Figure 4N-S**, and **Supporting Figure 5F-H**.
Revised Table: **Supporting Table S8**, **Supporting Table S9**, **Supporting Table S10**, **Supporting Table S11**.

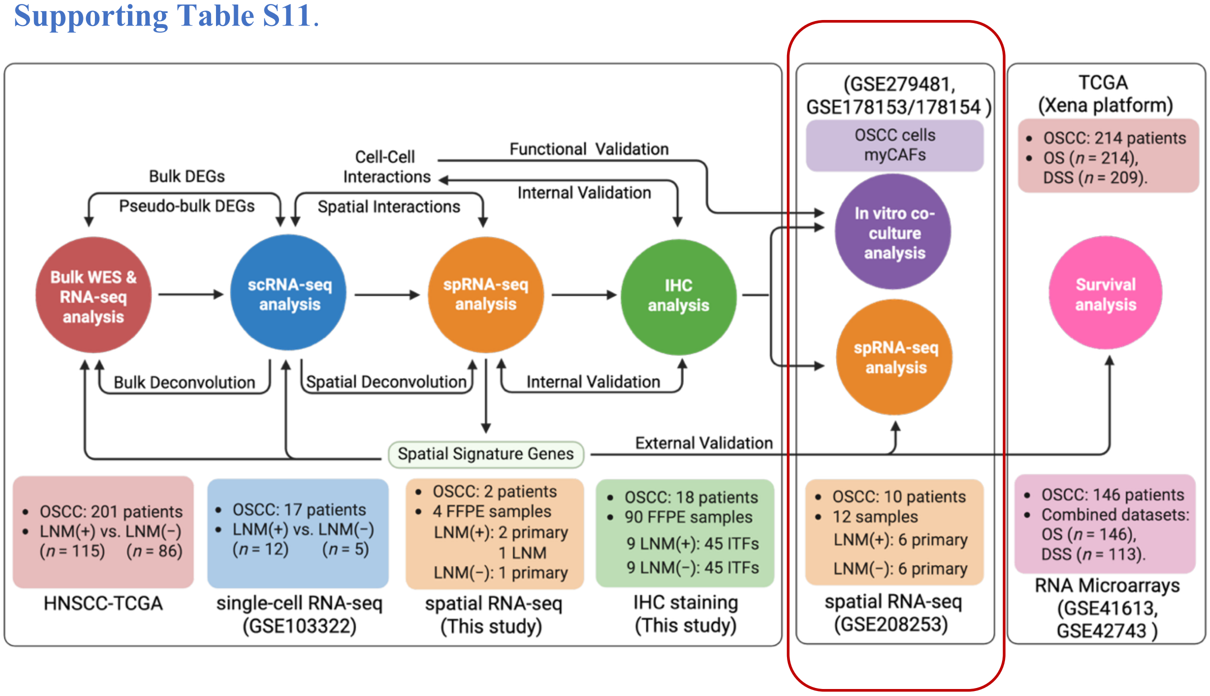

3. The discussion contrasts myCAF tumor-suppressive roles in pancreatic cancer with their pro-metastatic role here. A deeper exploration of tissue-specific CAF heterogeneity (e.g., comparing OSCC vs. PDAC stromal niches) would contextualize these findings.

RE: We thank the reviewer for the insightful suggestion regarding the need for deeper exploration of tissue-specific CAF heterogeneity to better contextualize our findings contrasting the pro-metastatic role of myCAFs in OSCC with their tumor-suppressive roles in PDAC. Indeed, myCAFs exhibit highly context-dependent functionality, influenced heavily by differences in the stromal niche. Adenocarcinoma (PDAC) is characterized by dense desmoplasia—a pronounced fibrotic stroma rich in αSMA-positive myCAFs that deposit abundant collagen type I, forming a physical barrier that restrains tumor expansion and limits invasive behavior. Landmark studies by Özdemir et al[121]. and Rhim et al[122]. clearly demonstrated that removal of these αSMA-rich myCAFs or reduction of fibrosis accelerated tumor progression, increased epithelial-mesenchymal transition (EMT), promoted immunosuppressive Treg infiltration, and led to poorer survival, underscoring a protective, tumor-restraining function of myCAFs in PDAC.

In stark contrast, OSCC typically presents with a less uniformly fibrotic, more permissive stromal niche. Here, myCAFs accumulate preferentially at invasive tumor fronts, facilitating cancer invasion and metastasis. They secrete factors such as TGF-β and matrix proteins (e.g., MFAP5), directly promoting EMT and cancer stemness. Studies by Vered et al[123]. and Ding et al[124]. provide clinical evidence that abundant αSMA-positive myCAFs correlate strongly with lymph node metastasis, advanced tumor stage, and poor prognosis in OSCC patients. These myCAFs are also actively involved in lymphangiogenesis and create an immune-suppressive microenvironment that facilitates tumor immune evasion, distinctively contrasting with PDAC myCAFs that maintain a more balanced immune milieu.

Recent single-cell and spatial transcriptomic analyses further refine these contrasts[125,126]. myCAFs in PDAC are spatially organized to maintain tumor containment, whereas myCAFs in OSCC facilitate tumor cell motility through a looser, patchy ECM structure. Additionally, PDAC stroma's mechanical stiffness, vasculature, and immune exclusion collectively contribute to tumor growth restriction[127], while the OSCC stroma provides pathways for cancer dissemination and nurtures immunosuppression[128].

Therefore, we agree with the reviewer that explicitly highlighting the unique stromal characteristics driving these functional divergences between PDAC and OSCC would significantly strengthen our manuscript. We have now revised the Discussion to succinctly emphasize these mechanistic contrasts, integrating recent insights into fibroblast heterogeneity and their stromal contexts. These clarifications provide a stronger biological rationale for our observed pro-metastatic role of myCAFs in OSCC and underscore the importance of precise stromal targeting in future therapeutic strategies.

UPDATE DISCUSSION: Although an increased presence of myCAFs in the TME has been associated with unfavorable outcomes in many solid cancers [12–14,33–35], the role of myCAFs varies depending on the type and its local environment (stromal niche). This difference in environment changes what myCAFs do. For instance, myCAFs can act as a tumor suppressor in

pancreatic ductal adenocarcinoma (PDAC) [36,37]. PDAC has a very dense stroma with myCAFs that deposit much collagen. This dense stroma forms a barrier that slows down tumor growth and invasion. Studies [38–41] show that removing these myCAFs or reducing this fibrosis makes PDAC grow faster and more aggressive, highlighting their protective role in this restrictive niche. In contrast, OSCC usually has a less dense, more open stroma. In this TME, myCAFs gather at the ITF and help OSCC cells invade and spread [12,14]. In our study, we found that increased numbers and functionality of myCAFs are associated with an LNM-positive microenvironment. myCAFs upregulated ECM-related genes, including those in the collagen gene family, in LNM-positive samples. Concurrently, OSCC cells upregulated ECM-associated receptors, specifically ITGA, ITGB, SDC, and CD44. This finding aligns with a previous report's finding that COL1A1 produced by fibroblasts interacts with CD44 in cancer cells, facilitating tumor progression in both HPV-positive and HPV-negative head and neck squamous cell carcinoma [43]. Additionally, through spatial transcriptomic and IHC analyses, we determined that the spatial interactions between OSCC cells and myCAFs occurred within the ITF. These findings show that myCAF function strongly depends on the specific stromal niche. While myCAFs can stop growth in dense PDAC tumors, oOur findings provide compelling evidence that myCAFs facilitate tumor progression in the OSCC microenvironment. Understanding these differences based on the TME is important for designing future treatments.

Revised text: **Discussion.**

4. The reliance on HPV-negative TCGA cases is justified but could be explicitly discussed in the context of HPV's known influence on HNSCC prognosis.

RE: The reviewer commented that a Human papillomavirus (HPV) influence on head and neck squamous cell carcinoma (HNSCC) prognosis is necessary and should be included in the Discussion. We have now added several lines and citing the five key papers below in the Discussion explaining why we used HPV-negative TCGA cases. HPV status is widely recognized as a critical independent prognostic factor in HNSCC, particularly within the oropharyngeal sub-site. Landmark clinical trials, such as the Radiation Therapy Oncology Group (RTOG; the RTOG 0129 study), have clearly demonstrated that HPV-positive HNSCC patients experience significantly improved overall survival compared to HPV-negative counterparts[129], reflecting distinct genomic and immune landscapes. HPV-positive tumors rarely exhibit disruptive mutations in TP53, likely due to viral E6-mediated p53 degradation, and frequently harbor activating PIK3CA mutations (observed in approximately 20–30% of cases compared to only ~5% in HPV-negative tumors)[130,131]. Additionally, HPV-positive tumors typically feature enhanced immune infiltration, particularly of CD8+ T-cells, correlating with superior disease-free survival and response to therapy[132].

However, it is critical to note that these prognostic implications predominantly apply to oropharyngeal carcinoma rather than oral cavity squamous cell carcinoma (OSCC). Recent comprehensive meta-analyses have demonstrated that HPV DNA is detected in approximately 6–10% of OSCC cases, significantly lower than the approximately 40–60% prevalence observed in oropharyngeal cancers[133]. Crucially, the few HPV-positive OSCC cases reported generally do not exhibit the pronounced survival benefit characteristic of HPV-positive oropharyngeal cancer.

Therefore, HPV's prognostic impact in OSCC is considered minimal, and routine clinical testing for p16/HPV status in OSCC is generally not advocated by clinical guidelines (e.g., National Comprehensive Cancer Network, NCCN guidelines), reflecting the virus's limited clinical relevance in this specific sub-site (oral cavity).

Given this clear epidemiological and clinical context, our study explicitly limited the TCGA analyses to HPV-negative OSCC cases to avoid confounding effects of HPV infection. Because tumor in anatomical boundary between the oral cavity and oropharynx overlapping regions such as the tongue base may share certain mutational and immune features. Therefore, by restricting our analysis to both oral cavity site annotations and HPV-negative status, we ensured the selection of a representative and biologically homogeneous OSCC cohort. This deliberate methodological decision allows us to isolate and robustly assess the prognostic value of the identified spatially-resolved 23-gene signature independent of viral influences. Such an approach not only ensures scientific rigor but also enhances clinical translatability, given the negligible role HPV plays in typical OSCC patient management and prognostication. We appreciate the reviewer for the comments, which have helped to improve the quality of the manuscript.

UPDATE DISCUSSION: Using publicly available multiomics data and original spatial transcriptomic data, we studied the role of myCAFs in promoting LNM in OSCC. For analyses using public HNSCC TCGA data, which includes various head and neck sub-sites, we applied strict inclusion criteria to define a homogeneous OSCC cohort. We focused on cases annotated as OSCC by site and confirmed HPV-negative status. While HPV is a critical prognostic factor in HNSCC, particularly in oropharyngeal tumors[129], its prevalence and prognostic impact are considerably lower (<10%) and limited in OSCC[133,134]. Accurately classifying cases and avoiding confounding is challenging in HNSCC cohorts like TCGA due to anatomical ambiguity (e.g., tongue base or tonsil region) and distinct HPV-positive tumor features[130–132]. Therefore, filtering by both specific oral cavity site annotation and HPV negativity was crucial. This created a homogeneous HPV-negative OSCC subtype, including cases from relevant boundary regions whose molecular profiles align with primary oral cavity tumors, thereby enhancing study validity. Analysis of this cohort revealed several initial insights. We found no significant association between key driver mutations and LNM status, suggesting that genetic events alone may not fully explain LNM potential. Instead, bulk RNA-seq analysis highlighted a muscle-contraction transcriptional program enriched in LNM-positive samples, indicating increased stromal activation. We also found a quantitative and functional activation of myCAFs in the LNM-positive microenvironment. In LNM-positive samples, myCAFs spatially colocalized with OSCC cells within the ITF, indicating their active role in promoting LNM. The myCAFs also sent ECM-related signals to OSCC cells, which activated key stemness-related genes in the OSCC cells through ECM-related receptors such as CD44. Furthermore, by analyzing the genes specifically expressed in the ITF, where myCAFs and OSCCs colocalize and where metastasis arises, we identified a 23-gene signature that was significantly associated with LNM status and poor OS in patients with OSCC.

Revised text: **Discussion.**

Minor comments:
1. In Figure 1C, ensure all gene names/abbreviations are defined (e.g., SYNE1, LRP1B).
RE: Thank you for pointing out this out. We defined all gene names/abbreviations in the Figure. 1C Legend and revised manuscript.

2. In Figure 4F, clarify how α-SMA positivity was quantified, which methodology used like percentage area or cell counts.

RE: Thank you very much for your comment. In Figure 4F, α-SMA positivity was initially quantified as the absolute myCAF density, defined as the α-SMA-positive area per square millimeter within each sample area, as measured using ImageJ. We did not use cell counts for this quantification.

To better characterize the composition of the OSCC microenvironment, especially within invasive tumor fronts (ITFs) that often display irregular architecture, we have now additionally included the relative proportion of α-SMA-positive area to the sum of α-SMA- and CK AE1/AE3-positive areas (myCAF proportion relative to tumor area, **Letter Figure L7A [new Figure 4F]**). This new metric reflects the proportion of myCAFs relative to tumor epithelial content, offering a complementary view of myCAF accumulation. The y-axis label and figure legend have been updated accordingly to clarify this distinction, and the Result section has been revised to define both measures.

Letter Figure L7A (new Figure 7B)

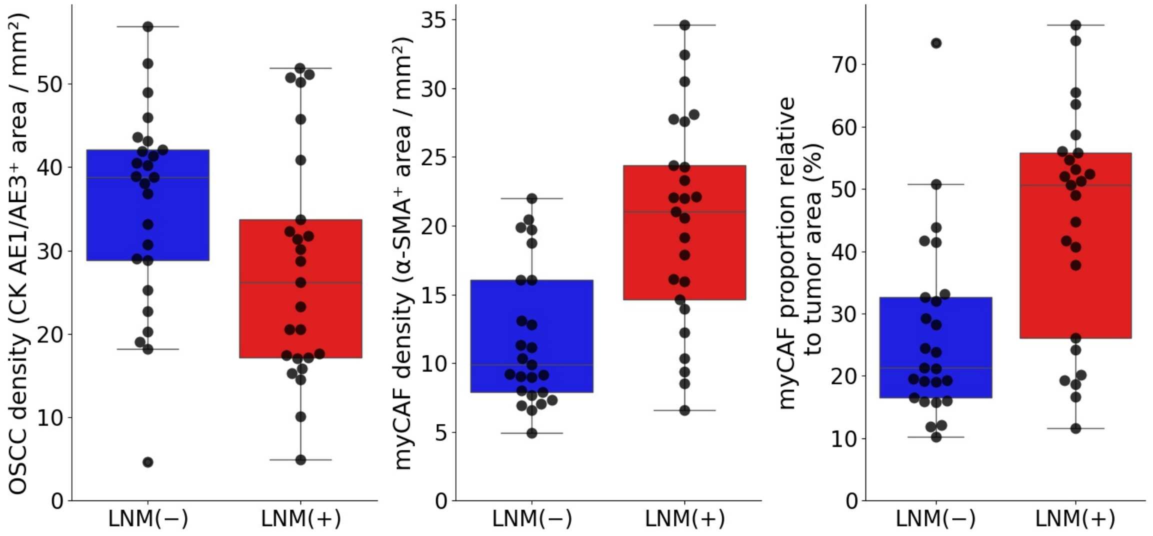

UPDATE RESULT: Consistent with the findings from the spatial transcriptomic analysis, significantly higher numbers of the absolute α-SMA-positive area per square millimeter (myCAF density) ~~myCAF markers (α smooth muscle actin [α-SMA]-~~positive markers) were detected in the ITFs of samples from patients with LNM compared with those of samples from patients without LNM (median : 19.14% [95% CI, 15.92%-21.99%] vs. 10.70% [95% CI, 9.06%-13.12%] , respectively; P <0.001; Figure 4F).

3. Address whether myCAF-OSCC interactions are exclusive to the ITF or also occur in central tumor regions.

RE: Thank you for your insightful question regarding the spatial distribution of myCAF-OSCC interactions. We appreciate the opportunity to clarify whether these interactions are confined to the invasive tumor front (ITF) or also occur in the central tumor areas.

Our investigations show that myCAF-OSCC interactions are not exclusive to the ITF, but they are significantly more prominent in this region (**Letter Figure L8A: Supporting Figure S4K** and **Letter Figure L8B; Supporting Figure S4N**). Spatial analysis revealed that while myCAFs are present in central tumor areas, their density is higher at the ITFs (**Letter Figure L8C: Figure 5G** and **Letter Figure L8D; Supporting Figure S4O**). Importantly, OSCC cells and myCAFs exhibit greater co-localization at the ITF, whereas in the tumor center, OSCC cells are more often found without nearby myCAF partners.

To further explore the importance of physical proximity suggested by these spatial observations and to directly address your query, we conducted supplementary in vitro co-culture experiments. It is important to note that these particular studies were designed to provide additional context in response to the review process. While the findings offer supporting insights, they are presented here with the acknowledgment of their preliminary nature due to experimental limitations, including a small sample size, which is why these specific data (**Letter Figure L8E-H**, **Letter Table L14, L15**) are detailed in this response letter rather than being incorporated into the main figures or results of the revised manuscript.

To model the contact-poor milieu of the central tumor regions, we cultured primary oral myofibroblasts in a trans-well system that prevents direct contact with OSCC cells (HSC-3, Ca9-22, **Letter Table L14** and **Letter Figure L8E**). Detailed explanations of the co-culture used in this study are described in previous papers [135,136]. Under these conditions, we observed only minimal changes in the expression of key ECM-related genes in myCAF-like cells, suggesting that close contact might be necessary for robust myCAF activation.

In support of this, spatial transcriptomic data from patient samples clearly showed a strong upregulation of the ECM signature specifically in myCAF-enriched regions at the invasive tumor front, where myCAFs and OSCC cells frequently interact directly. This finding contrasts with the less active state of myCAFs observed in the contact-limited in vitro model.

Taken together, these results indicate that while myCAFs exist throughout the tumor, the intense crosstalk between myCAFs and OSCC cells, particularly the pro-invasive ECM remodeling, is largely dependent on direct cell-cell contact. This contact dependency explains why these aggressive interactions are most characteristic of the invasive tumor front where tumor cells and myCAFs are in close proximity.

Letter Figure L8A (Supporting Figure S4J)

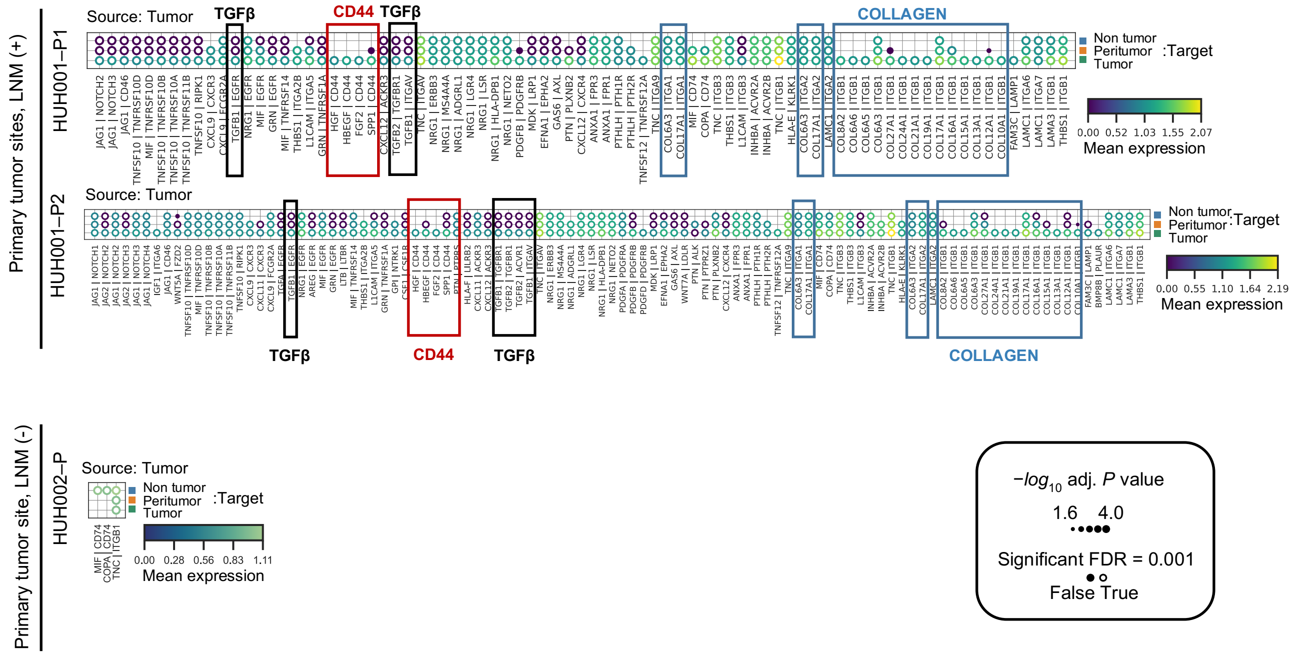

Letter Figure L8B (Supporting Figure S4N)

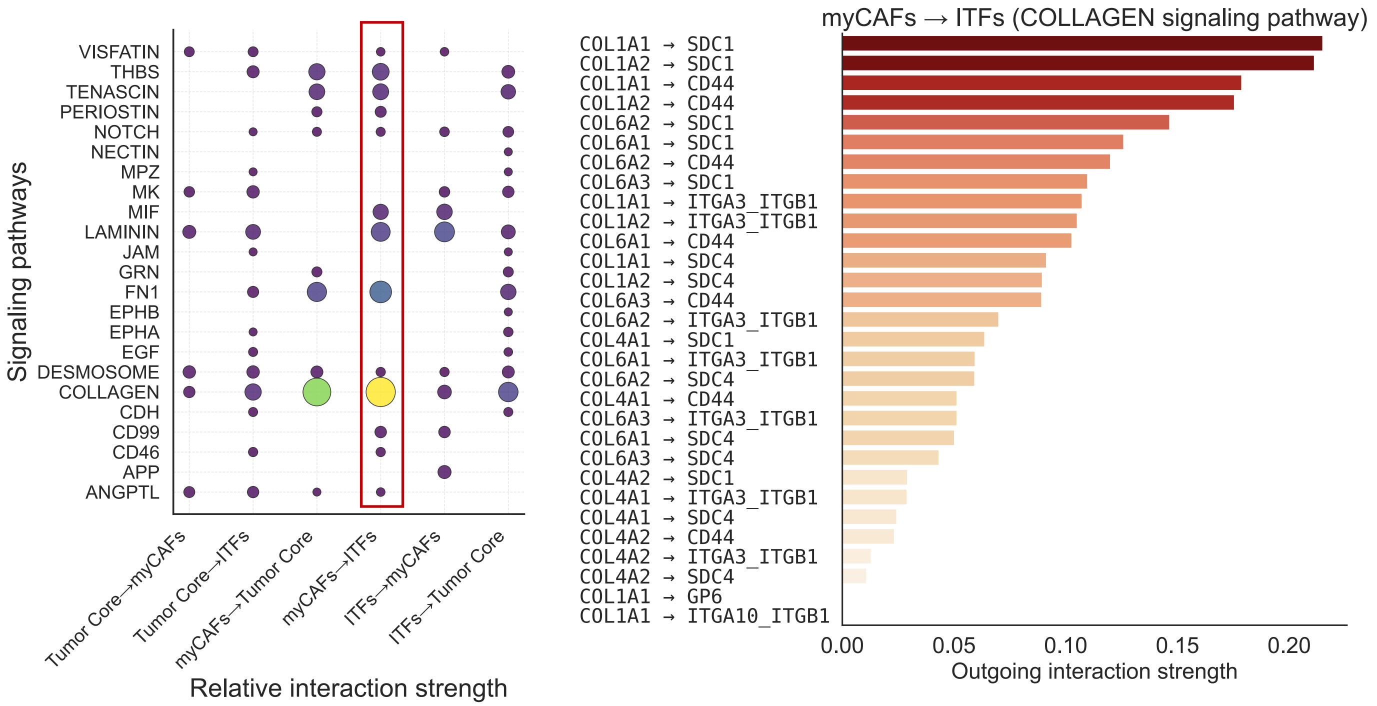

Letter Figure L8C (Figure 5G)

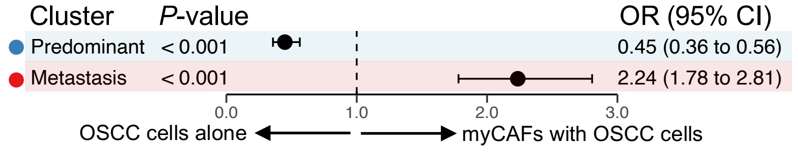

Letter Figure L8D (Supporting Figure S4O)

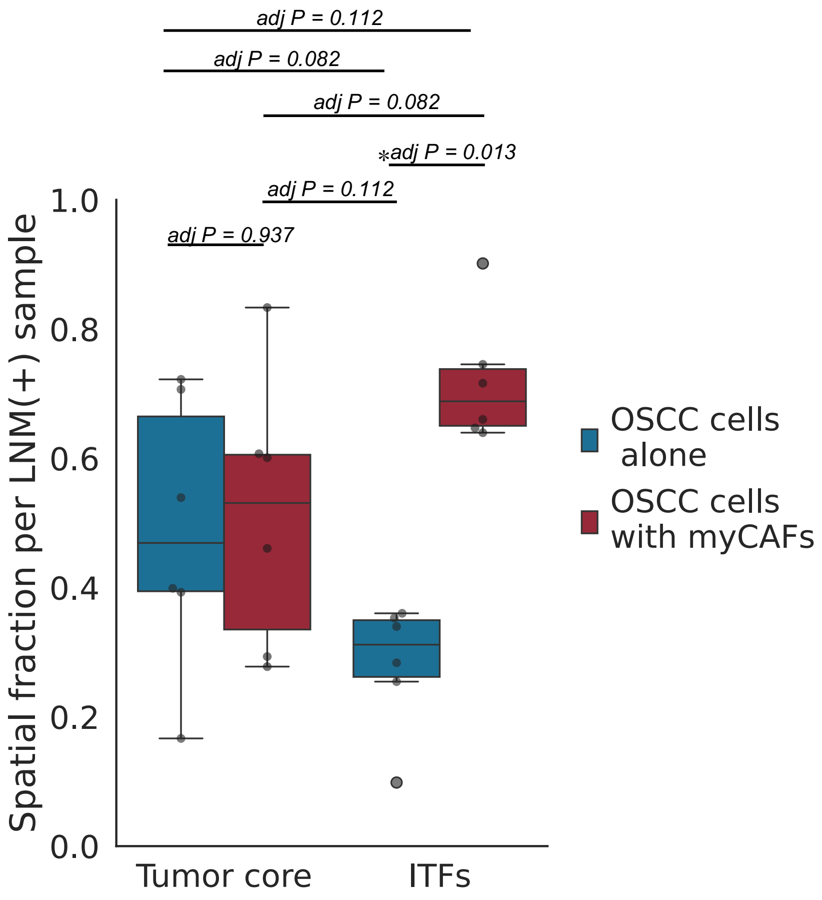

We processed microarray CEL files, performing background correction and normalization with the Robust Multi-Array Average (RMA) method, and annotated probes to gene symbols using the hugene20sttranscriptcluster.db database. We then filtered out low-variance genes (bottom 20%) and applied batch effect correction using the ComBat method across core myCAF markers and ECM genes. We first confirmed the expression level of the myCAFs marker (*ACTA2, TANGLN, MYL9, TPM2, MCAM*, **Letter Figure L8F**).

Letter Table L14

sample	cell type	treatment	co-culture
myFB1	myofiroblasts	Control	
myCAF-like1	myCAFs	Co-cultured	(myFB1_HSC3)
myCAF-like2	myCAFs	Co-cultured	(myFB1_Ca9-22)
myFB2	myofiroblasts	Control	
myCAF-like3	myCAFs	Co-cultured	(myFB2_Ca9-22)
myFB3	myofiroblasts	Co-cultured	(myFB2_myCAF3)

Letter Figure L8E

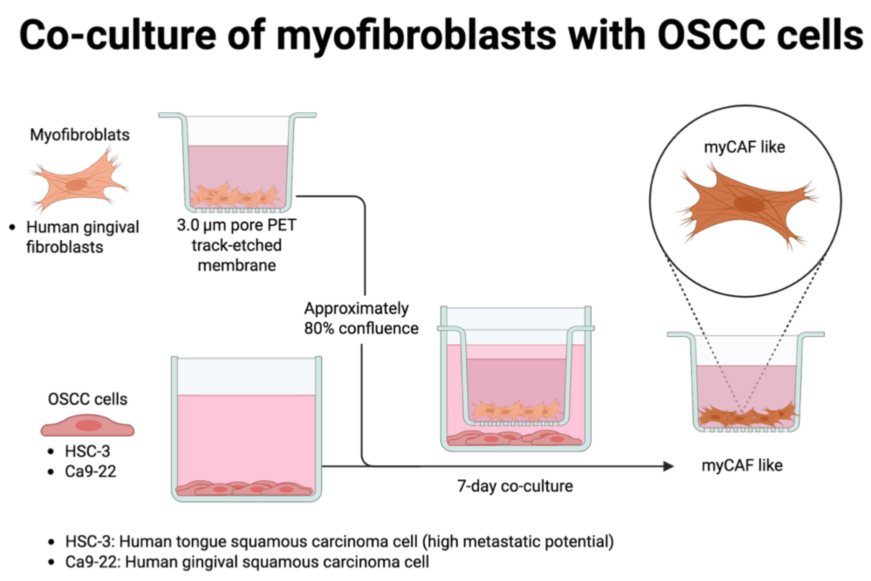

Letter Figure L8F

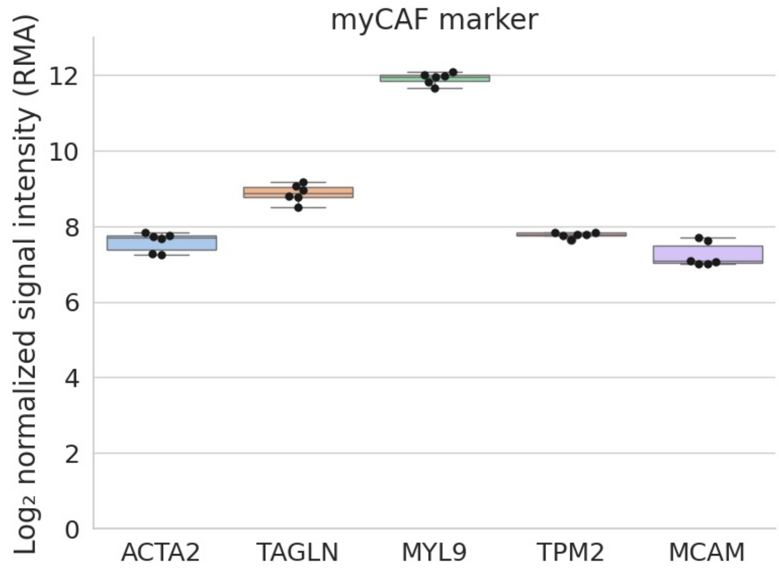

Next, we applied Z-score standardized for each gene, for comparison across samples. None of the fifteen core ECM genes showed significant induction in myCAF like (median $\log_2$FC = −0.18; all FDR [adjusted P] > 0.10, Letter Table L15; Letter Figure L8E). Although the small sample size limits statistical power, the uniformly small effect sizes argue against a biologically meaningful ECM up-regulation without cell-cell communication.

Letter Table L15

	log2FC	*P*	FDR
COL1A1	-0.65	0.11	0.23
COL1A2	-0.48	0.11	0.23
COL3A1	-0.71	0.25	0.31
COL4A1	0.09	0.82	0.88
COL4A2	0.10	0.15	0.29
COL5A1	-0.76	0.11	0.23
COL5A2	-0.69	0.11	0.23
COL5A3	-0.17	0.22	0.31
COL6A1	-0.12	0.25	0.31
COL6A2	-0.02	1.00	1.00
COL6A3	-0.43	0.25	0.31
COL7A1	0.04	0.11	0.23
COL13A1	0.01	0.62	0.71
LAMB1	-0.70	0.11	0.23
LAMB3	0.58	0.11	0.23

Letter Figure L8G

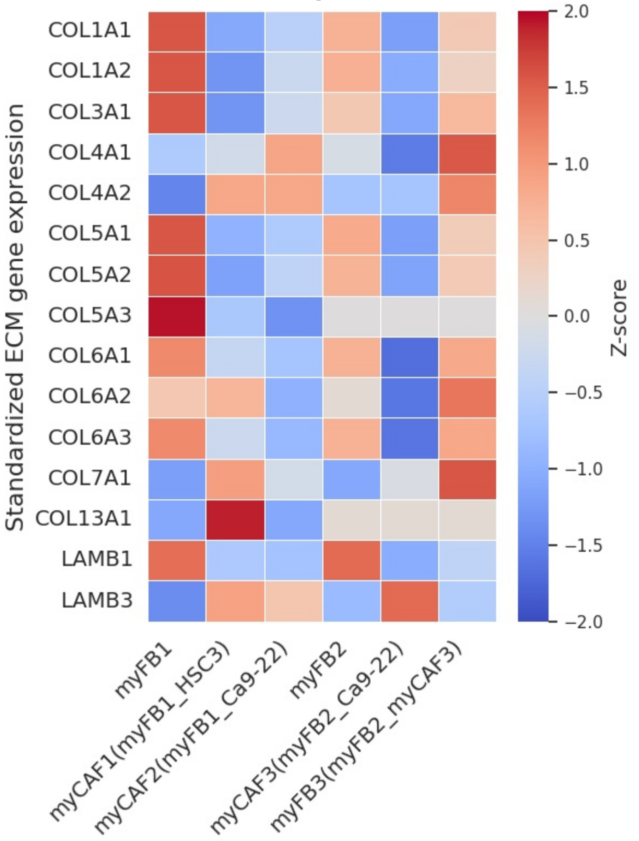

We compared the expression of ECM genes between myofibroblast (myFB) and myCAF groups using $\log_2$ fold change and Mann–Whitney U tests with FDR correction. While no genes reached statistical significance (all FDR > 0.23), we observed a trend toward decreased expression of collagens (e.g., COL1A1, COL3A1, COL5A1) and increased expression of LAMB3 in myCAFs compared to myFBs. These patterns may reflect the areas of OSCC cells alone in the OSCC microenvironment and suggest that potential co-localization and direct contact between tumor cells and myCAFs could activate myCAF–OSCC interaction. Nevertheless, further experimental validation is required.

Letter Figure L8H

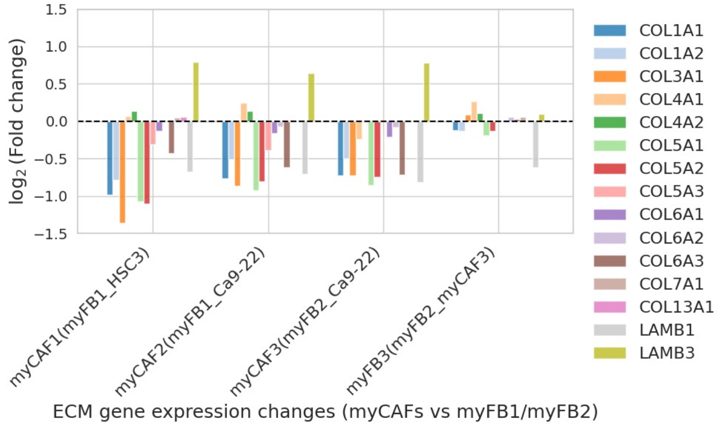

4. Please define abbreviations at first mention, such as TME in the abstract, scVI in Results, etc.

RE: Thank you. This was corrected. tumor microenvironment (TME) and single-cell Variational Inference (scVI) in revised manuscript.

Letter References

1. Biancalani T, Scalia G, Buffoni L, Avasthi R, Lu Z, Sanger A, et al. Deep learning and alignment of spatially resolved single-cell transcriptomes with Tangram. Nat Methods. 2021;18: 1352–1362.

2. Li H, Zhou J, Li Z, Chen S, Liao X, Zhang B, et al. A comprehensive benchmarking with practical guidelines for cellular deconvolution of spatial transcriptomics. Nat Commun. 2023;14: 1548.

3. Li B, Zhang W, Guo C, Xu H, Li L, Fang M, et al. Benchmarking spatial and single-cell transcriptomics integration methods for transcript distribution prediction and cell type deconvolution. Nat Methods. 2022;19: 662–670.

4. Brandwein-Gensler M, Teixeira MS, Lewis CM, Lee B, Rolnitzky L, Hille JJ, et al. Oral squamous cell carcinoma: histologic risk assessment, but not margin status, is strongly predictive of local disease-free and overall survival. Am J Surg Pathol. 2005;29: 167–178.

5. Xiao C, Wang L, Zhu L, Zhang C, Zhou J. Secreted frizzled-related protein 2 is epigenetically silenced and functions as a tumor suppressor in oral squamous cell carcinoma. Mol Med Rep. 2014;10: 2293–2298.

6. Yang Q, Huang T, Ye G, Wang B, Zhang X. Methylation of SFRP2 gene as a promising noninvasive biomarker using feces in colorectal cancer diagnosis: a systematic meta-analysis. Sci Rep. 2016;6: 33339.

7. Garcia D, Nasarre P, Bonilla IV, Hilliard E, Peterson YK, Spruill L, et al. Development of a novel humanized monoclonal antibody to secreted frizzled-related protein-2 that inhibits triple-negative breast cancer and angiosarcoma growth in vivo. Ann Surg Oncol. 2019;26: 4782–4790.

8. Fane ME, Ecker BL, Kaur A, Marino GE, Alicea GM, Douglass SM, et al. SFRP2 supersedes VEGF as an age-related driver of angiogenesis in melanoma, affecting response to anti-VEGF therapy in older patients. Clin Cancer Res. 2020;26: 5709–5719.

9. van Loon K, Huijbers EJM, Griffioen AW. Secreted frizzled-related protein 2: a key player in noncanonical Wnt signaling and tumor angiogenesis. Cancer Metastasis Rev. 2021;40: 191–203.

10. Matsuda Y, Hagio M, Ishiwata T. Nestin: a novel angiogenesis marker and possible target for tumor angiogenesis. World J Gastroenterol. 2013;19: 42–48.

11. Ravindran G, Devaraj H. Prognostic significance of neural stem cell markers, Nestin and Musashi-1, in oral squamous cell carcinoma: expression pattern of Nestin in the precancerous stages of oral squamous epithelium. Clin Oral Investig. 2015;19: 1251–1260.

12. Asleh K, Won JR, Gao D, Voduc KD, Nielsen TO. Nestin expression in breast cancer: association with prognosis and subtype on 3641 cases with long-term follow-up. Breast Cancer Res Treat. 2018;168: 107–115.

13. Krüger K, Wik E, Knutsvik G, Nalwoga H, Klingen TA, Arnes JB, et al. Expression of Nestin associates with BRCA1 mutations, a basal-like phenotype and aggressive breast cancer. Sci Rep. 2017;7. doi:10.1038/s41598-017-00862-w

14. Prosniak M, Kenyon LC, Hooper DC. Glioblastoma contains topologically distinct proliferative and metabolically defined subpopulations of Nestin- and Glut1-expressing cells. J Neuropathol Exp Neurol. 2021;80: 674–684.

15. Melander MC, Jürgensen HJ, Madsen DH, Engelholm LH, Behrendt N. The collagen receptor uPARAP/Endo180 in tissue degradation and cancer (Review). Int J Oncol. 2015;47: 1177–1188.

16. Chen J, Yang J, Li H, Yang Z, Zhang X, Li X, et al. Single-cell transcriptomics reveal the intratumoral landscape of infiltrated T-cell subpopulations in oral squamous cell carcinoma. Mol Oncol. 2021;15: 866–886.

17. Evans RJ, Perkins DW, Selfe J, Kelsey A, Birch GP, Shipley JM, et al. Endo180 (MRC2) antibody-drug conjugate for the treatment of sarcoma. Mol Cancer Ther. 2023;22: 240–253.

18. Gopalakrishnan KV, Kannan B, Pandi C, Jayaseelan VP, Arumugam P. Prognostic and clinicopathological significance of MRC2 expression in head and neck squamous cell carcinoma. J Stomatol Oral Maxillofac Surg. 2023;124: 101617.

19. Liu Y, Liang J, Zhang Y, Guo Q. Drug resistance and tumor immune microenvironment: An overview of current understandings (Review). Int J Oncol. 2024;65. doi:10.3892/ijo.2024.5684

20. Zhu L, Jiang Q, Meng J, Zhao H, Lin J. Pan-cancer analysis of COL15A1: an immunological and prognostic biomarker. Discov Oncol. 2024;15: 325.

21. Gao Y, Li J, Cheng W, Diao T, Liu H, Bo Y, et al. Cross-tissue human fibroblast atlas reveals myofibroblast subtypes with distinct roles in immune modulation. Cancer Cell. 2024;42: 1764-1783.e10.

22. Liu Y-T, Liu H-M, Ren J-G, Zhang W, Wang X-X, Yu Z-L, et al. Immune-featured stromal niches associate with response to neoadjuvant immunotherapy in oral squamous cell carcinoma. Cell Rep Med. 2025;6: 102024.

23. Jia Q, Xu B, Zhang Y, Ali A, Liao X. CCN family proteins in cancer: Insight into their structures and coordination role in tumor microenvironment. Front Genet. 2021;12: 649387.

24. Moritani NH, Kubota S, Nishida T, Kawaki H, Kondo S, Sugahara T, et al. Suppressive effect of overexpressed connective tissue growth factor on tumor cell growth in a human oral squamous cell carcinoma-derived cell line. Cancer Lett. 2003;192: 205–214.

25. Yang W-Y, Tang C-H, Chuang J-Y. Abstract 1392: CTGF inhibits cell motility in oral cancer cells through reducing COX-2 expression. Cancer Res. 2011;71: 1392–1392.

26. Wu Y-L, Li H-Y, Zhao X-P, Jiao J-Y, Tang D-X, Yan L-J, et al. Mesenchymal stem cell-derived CCN2 promotes the proliferation, migration and invasion of human tongue squamous cell carcinoma cells. Cancer Sci. 2017;108: 897–909.

27. Yamazaki M, Maruyama S, Abé T, Essa A, Babkair H, Cheng J, et al. MFG-E8 expression for progression of oral squamous cell carcinoma and for self-clearance of apoptotic cells. Lab Invest. 2014;94: 1260–1272.

28. Okamoto A, Sakakura K, Takahashi H, Motegi S-I, Kaira K, Yokobori-Kuwabara Y, et al. Immunological and clinicopathological significance of MFG-E8 expression in patients with oral squamous cell carcinoma. Pathol Oncol Res. 2020;26: 1263–1268.

29. Jung H, Kang J, Han K-M, Kim H. Prognostic value of Pentraxin3 protein expression in human malignancies: A systematic review and meta-analysis. Cancers (Basel). 2024;16. doi:10.3390/cancers16223754

30. Chang W-C, Wu S-L, Huang W-C, Hsu J-Y, Chan S-H, Wang J-M, et al. PTX3 gene activation in EGF-induced head and neck cancer cell metastasis. Oncotarget. 2015;6: 7741–7757.

31. Guo Y, Pan WK, Wang ZW, Su WH, Xu K, Jia H, et al. Identification of novel biomarkers for predicting prognosis and immunotherapy response in head and neck squamous cell carcinoma based on ceRNA network and immune infiltration analysis. Biomed Res Int. 2021;2021: 4532438.

32. Natarajan J, Hunter K, Mutalik VS, Radhakrishnan R. Overexpression of S100A4 as a biomarker of metastasis and recurrence in oral squamous cell carcinoma. J Appl Oral Sci. 2014;22: 426–433.

33. Moriyama-Kita M, Endo Y, Yonemura Y, Heizmann CW, Schäfer BW, Sasaki T, et al. Correlation of S100A4 expression with invasion and metastasis in oral squamous cell carcinoma. Oral Oncol. 2004;40: 496–500.

34. Zhang J, Nakamura T, Ito R, Ohbayashi C, Maeda S. The evaluation of both metastasis and prognosis of oral squamous cell carcinoma by S100A4 and E-cadherin immunostaining. Oral Med Pathol. 2004;9: 67–74.

35. Park W-Y, Gray JM, Holewinski RJ, Andresson T, So JY, Carmona-Rivera C, et al. Apoptosis-induced nuclear expulsion in tumor cells drives S100a4-mediated metastatic outgrowth through the RAGE pathway. Nat Cancer. 2023;4: 419–435.

36. Condamine T, Dominguez GA, Youn J-I, Kossenkov AV, Mony S, Alicea-Torres K, et al. Lectin-type oxidized LDL receptor-1 distinguishes population of human polymorphonuclear myeloid-derived suppressor cells in cancer patients. Sci Immunol. 2016;1. doi:10.1126/sciimmunol.aaf8943

37. Gallenkamp J, Spanier G, Wörle E, Englbrecht M, Kirschfink M, Greslechner R, et al. A novel multiplex detection array revealed systemic complement activation in oral squamous cell carcinoma. Oncotarget. 2018;9: 3001–3013.

38. Jing F, Mu J, Liu J, Hu C, Wu F, Gao Q. Senescent vascular endothelial cells promote oral squamous cell carcinoma progression through complement C3 activation. Arch Oral Biol. 2025;174: 106242.

39. Rezvani K. UBXD proteins: A family of proteins with diverse functions in cancer. Int J Mol Sci. 2016;17: 1724.

40. Ketkar H, Harrison AG, Graziano VR, Geng T, Yang L, Vella AT, et al. UBX domain protein 6 positively regulates JAK-STAT1/2 signaling. J Immunol. 2021;206: 2682–2691.

41. Broutian TR, Jiang B, Li J, Akagi K, Gui S, Zhou Z, et al. Human papillomavirus insertions identify the PIM family of serine/threonine kinases as targetable driver genes in head and neck squamous cell carcinoma. Cancer Lett. 2020;476: 23–33.

42. Etemad-Moghadam S, Khalili M, Tirgary F, Alaeddini M. Evaluation of myofibroblasts in oral epithelial dysplasia and squamous cell carcinoma. J Oral Pathol Med. 2009;38: 639–643.

43. Hnia K, Ramspacher C, Vermot J, Laporte J. Desmin in muscle and associated diseases: beyond the structural function. Cell Tissue Res. 2015;360: 591–608.

44. Kros JM, Zeneyedpour L, Pedrosa RMSM, Belcaid Z, Dik WA, Luider TM, et al. T cell induced expression of Coronin-1A facilitates blood-brain barrier transmigration of breast cancer cells. Sci Rep. 2024;14: 31516.

45. Oku T, Kaneko Y, Murofushi K, Seyama Y, Toyoshima S, Tsuji T. Phorbol ester-dependent phosphorylation regulates the association of p57/coronin-1 with the actin cytoskeleton. J Biol Chem. 2008;283: 28918–28925.

46. Rahmati Nezhad P, Riihilä P, Knuutila JS, Viiklepp K, Peltonen S, Kallajoki M, et al. Complement factor D is a novel biomarker and putative therapeutic target in cutaneous squamous cell carcinoma. Cancers (Basel). 2022;14: 305.

47. Ezure T, Sugahara M, Amano S. Senescent dermal fibroblasts negatively influence fibroblast extracellular matrix-related gene expression partly via secretion of complement factor D. Biofactors. 2019;45: 556–562.

48. You P, Wang D, Liu Z, Guan S, Xiao N, Chen H, et al. Knockdown of RFC4 inhibits cell proliferation of oral squamous cell carcinoma in vitro and in vivo. FEBS Open Bio. 2025;15: 346–358.

49. Hou Y, Wang S, Zhang Y, Huang X, Zhang X, He F, et al. Proteomics identifies LUC7L3 as a prognostic biomarker for hepatocellular carcinoma. Curr Issues Mol Biol. 2024;46: 4004–4020.

50. Solomon-Zemler R, Pozniak Y, Geiger T, Werner H. Identification of nucleolar protein NOM1 as a novel nuclear IGF1R-interacting protein. Mol Genet Metab. 2019;126: 259–265.

51. Simmons HM, Ruis BL, Kapoor M, Hudacek AW, Conklin KF. Identification of NOM1, a nucleolar, eIF4A binding protein encoded within the chromosome 7q36 breakpoint region targeted in cases of pediatric acute myeloid leukemia. Gene. 2005;347: 137–145.

52. Dong C, Dong G, Li L, Zhu L, Tempel W, Liu Y, et al. An asparagine/glycine switch governs product specificity of human N-terminal methyltransferase NTMT2. Commun Biol. 2018;1: 183.

53. Tan L, Li W, Su Q. The comprehensive analysis of the prognostic and functional role of N-terminal methyltransferases 1 in pan-cancer. PeerJ. 2023;11: e16263.

54. Chen P, Huang R, Hazbun TR. Unlocking the mysteries of alpha-N-terminal methylation and its diverse regulatory functions. J Biol Chem. 2023;299: 104843.

55. Zhao C, Yu M, Li Y. Pan-cancer analysis reveals the pro-oncogenic role of N6-methyladenosine (m6A)-regulated NTMT1 in head and neck squamous cell carcinoma. J Biochem Mol Toxicol. 2024;38: e23603.

56. Bonsignore LA, Butler JS, Klinge CM, Schaner Tooley CE. Loss of the N-terminal methyltransferase NRMT1 increases sensitivity to DNA damage and promotes mammary oncogenesis. Oncotarget. 2015;6: 12248–12263.

57. Shields KM, Tooley JG, Petkowski JJ, Wilkey DW, Garbett NC, Merchant ML, et al. Select human cancer mutants of NRMT1 alter its catalytic activity and decrease N-terminal trimethylation. Protein Sci. 2017;26: 1639–1652.

58. Faughn JD, Dean WL, Schaner Tooley CE. The N-terminal methyltransferase homologs NRMT1 and NRMT2 exhibit novel regulation of activity through heterotrimer formation. Protein Sci. 2018;27: 1585–1599.

59. Petkowski JJ, Schaner Tooley CE, Anderson LC, Shumilin IA, Balsbaugh JL, Shabanowitz J, et al. Substrate specificity of mammalian N-terminal α-amino methyltransferase NRMT. Biochemistry. 2012;51: 5942–5950.

60. Shen C, Han C, Li Z, Yan Y, Li C, Chen H, et al. Construction and validation of a prognostic model based on pyroptosis-related genes in bladder cancer. Comb Chem High Throughput Screen. 2024;27: 2335–2349.

61. Wang X-W, Madeddu L, Spirohn K, Martini L, Fazzone A, Becchetti L, et al. Assessment of community efforts to advance network-based prediction of protein-protein interactions. Nat Commun. 2023;14: 1582.

62. Huttlin EL, Bruckner RJ, Navarrete-Perea J, Cannon JR, Baltier K, Gebreab F, et al. Dual proteome-scale networks reveal cell-specific remodeling of the human interactome. Cell. 2021;184: 3022-3040.e28.

63. Kamat SS, Camara K, Parsons WH, Chen D-H, Dix MM, Bird TD, et al. Immunomodulatory lysophosphatidylserines are regulated by ABHD16A and ABHD12 interplay. Nat Chem Biol. 2015;11: 164–171.

64. Yan J, Zhang C, Xu Y, Huang Z, Ye Q, Qian X, et al. GPR34 is a metabolic immune checkpoint for ILC1-mediated antitumor immunity. Nat Immunol. 2024;25: 2057–2067.

65. Zhang X, Guo J, Shi X, Zhou X, Chen Q. LUC7L3 is a downstream factor of SRSF1 and prevents genomic instability. Cell Insight. 2024;3: 100170.

66. Li Y-Y, Mukaida N. Pathophysiological roles of Pim-3 kinase in pancreatic cancer development and progression. World J Gastroenterol. 2014;20: 9392–9404.

67. Atalay P, Ozpolat B. PIM3 kinase: A promising novel target in solid cancers. Cancers (Basel). 2024;16. doi:10.3390/cancers16030535

68. Choudhury R, Bahadi CK, Ray IP, Dash P, Pattanaik I, Mishra S, et al. PIM1 kinase and its diverse substrate in solid tumors. Cell Commun Signal. 2024;22: 529.

69. Cervantes-Gomez F, Stellrecht CM, Ayres ML, Keating MJ, Wierda WG, Gandhi V. PIM kinase inhibitor, AZD1208, inhibits protein translation and induces autophagy in primary chronic lymphocytic leukemia cells. Oncotarget. 2019;10: 2793–2809.

70. Bellon M, Nicot C. Targeting Pim kinases in hematological cancers: molecular and clinical review. Mol Cancer. 2023;22: 18.

71. Luszczak S, Kumar C, Sathyadevan VK, Simpson BS, Gately KA, Whitaker HC, et al. PIM kinase inhibition: co-targeted therapeutic approaches in prostate cancer. Signal Transduct Target Ther. 2020;5: 7.

72. Cortes J, Tamura K, DeAngelo DJ, de Bono J, Lorente D, Minden M, et al. Phase I studies of AZD1208, a proviral integration Moloney virus kinase inhibitor in solid and haematological cancers. Br J Cancer. 2018;118: 1425–1433.

73. Murdocca M, De Masi C, Pucci S, Mango R, Novelli G, Di Natale C, et al. LOX-1 and cancer: an indissoluble liaison. Cancer Gene Ther. 2021;28: 1088–1098.

74. Wu L, Liu Y, Deng W, Wu T, Bu L, Chen L. OLR1 is a pan-cancer prognostic and immunotherapeutic predictor associated with EMT and cuproptosis in HNSCC. Int J Mol Sci. 2023;24. doi:10.3390/ijms241612904

75. Zhang P, Zhao Y, Xia X, Mei S, Huang Y, Zhu Y, et al. Expression of OLR1 gene on tumor-associated macrophages of head and neck squamous cell carcinoma, and its correlation with clinical outcome. Oncoimmunology. 2023;12: 2203073.

76. Castro F, Cardoso AP, Gonçalves RM, Serre K, Oliveira MJ. Interferon-gamma at the Crossroads of tumor immune surveillance or evasion. Front Immunol. 2018;9: 847.

77. Ding H, Wang G, Yu Z, Sun H, Wang L. Role of interferon-gamma (IFN-γ) and IFN-γ receptor 1/2 (IFNγR1/2) in regulation of immunity, infection, and cancer development: IFN-γ-dependent or independent pathway. Biomed Pharmacother. 2022;155: 113683.

78. Rosenzweig SD, Schwartz OM, Brown MR, Leto TL, Holland SM. Characterization of a dipeptide motif regulating IFN-gamma receptor 2 plasma membrane accumulation and IFN-gamma responsiveness. J Immunol. 2004;173: 3991–3999.

79. Soh J, Donnelly RJ, Kotenko S, Mariano TM, Cook JR, Wang N, et al. Identification and sequence of an accessory factor required for activation of the human interferon gamma receptor. Cell. 1994;76: 793–802.

80. Zhang S-F, Wang X-Y, Fu Z-Q, Peng Q-H, Zhang J-Y, Ye F, et al. TXNDC17 promotes paclitaxel resistance via inducing autophagy in ovarian cancer. Autophagy. 2015;11: 225–238.

81. Zhang Z, Wang A, Li H, Zhi H, Lu F. RETRACTED: STAT3-dependent TXNDC17 expression mediates Taxol resistance through inducing autophagy in human colorectal cancer cells. Gene. 2016;584: 75–82.

82. Kocatürk B. Identification of thioredoxin domain containing family members' expression pattern and prognostic value in diffuse gliomas via in silico analysis. Cancer Med. 2023;12: 3830–3844.

83. Pham D, Tan X, Balderson B, Xu J, Grice LF, Yoon S, et al. Robust mapping of spatiotemporal trajectories and cell-cell interactions in healthy and diseased tissues. Nat Commun. 2023;14: 7739.

84. Feng Q-S, Shan X-F, Yau V, Cai Z-G, Xie S. Facilitation of tumor stroma-targeted therapy: Model difficulty and co-culture organoid method. Pharmaceuticals (Basel). 2025;18. doi:10.3390/ph18010062

85. Arebro J, Lee C-M, Bennewith KL, Garnis C. Cancer-associated fibroblast heterogeneity in malignancy with focus on oral squamous cell carcinoma. Int J Mol Sci. 2024;25: 1300.

86. Su B, Sun R, Zhang L, Zhang J, Feng X, Cao Z, et al. Single-cell RNA sequencing-based immunological feature analysis of a COVID-19 patient with recurrent positive SARS-CoV-2 RNA. Chin Med J (Engl). 2022;135: 1000–1002.

87. Verstegen NJM, Pollastro S, Unger P-PA, Marsman C, Elias G, Jorritsma T, et al. Single-cell analysis reveals dynamics of human B cell differentiation and identifies novel B and antibody-secreting cell intermediates. Elife. 2023;12. doi:10.7554/eLife.83578

88. Takahashi K, Podyma-Inoue KA, Saito M, Sakakitani S, Sugauchi A, Iida K, et al. TGF-β generates a population of cancer cells residing in G1 phase with high motility and metastatic potential via KRTAP2-3. Cell Rep. 2022;40: 111411.

89. Qin Y, He LD, Sheng ZJ, Yong MM, Sheng YS, Wei Dong X, et al. Increased CCL19 and CCL21 levels promote fibroblast ossification in ankylosing spondylitis hip ligament tissue. BMC Musculoskelet Disord. 2014;15: 316.

90. Chen Z, Zhou L, Liu L, Hou Y, Xiong M, Yang Y, et al. Single-cell RNA sequencing highlights the role of inflammatory cancer-associated fibroblasts in bladder urothelial carcinoma. Nat Commun. 2020;11: 5077.

91. Kieffer Y, Hocine HR, Gentric G, Pelon F, Bernard C, Bourachot B, et al. Single-cell analysis reveals fibroblast clusters linked to immunotherapy resistance in cancer. Cancer Discov. 2020;10: 1330–1351.

92. Cords L, Tietscher S, Anzeneder T, Langwieder C, Rees M, de Souza N, et al. Cancer-associated fibroblast classification in single-cell and spatial proteomics data. Nat Commun. 2023;14: 4294.

93. Chen L, Shi V, Wang S, Sun L, Freeman R, Yang J, et al. SCCA1/SERPINB3 suppresses antitumor immunity and blunts therapy-induced T cell responses via STAT-dependent chemokine production. J Clin Invest. 2023;133. doi:10.1172/JCI163841

94. Hu Y, Han Y, He M, Zhang Y, Zou X. S100 proteins in head and neck squamous cell carcinoma (Review). Oncol Lett. 2023;26: 362.

95. Raffat MA, Hadi NI, Hosein M, Mirza S, Ikram S, Akram Z. S100 proteins in oral squamous cell carcinoma. Clin Chim Acta. 2018;480: 143–149.

96. Qu ZF, Ma H, Duan XF, Wu R, Zou Y. The expression and significance of S100A9 in oral squamous cell carcinoma. Lin Chuang Er Bi Yan Hou Tou Jing Wai Ke Za Zhi. 2017;31: 219–222.

97. Rodig SJ, Kutok JL, Paterson JC, Nitta H, Zhang W, Chapuy B, et al. The pre-B-cell receptor associated protein VpreB3 is a useful diagnostic marker for identifying c-MYC translocated lymphomas. Haematologica. 2010;95: 2056–2062.

98. Kurkalang S, Roy S, Acharya A, Mazumder P, Mazumder S, Patra S, et al. Single-cell transcriptomic analysis of gingivo-buccal oral cancer reveals two dominant cellular programs. Cancer Sci. 2023;114: 4732–4746.

99. Meyer-Hoffert U, Wu Z, Kantyka T, Fischer J, Latendorf T, Hansmann B, et al. Isolation of SPINK6 in human skin. J Biol Chem. 2010;285: 32174–32181.

100. Nakayama R, Arikawa K, Bhawal UK. The epigenetic regulation of CXCL14 plays a role in the pathobiology of oral cancers. J Cancer. 2017;8: 3014–3027.

101. Krishnamurty AT, Shyer JA, Thai M, Gandham V, Buechler MB, Yang YA, et al. LRRC15+ myofibroblasts dictate the stromal setpoint to suppress tumour immunity. Nature. 2022;611: 148–154.

102. Dominguez CX, Müller S, Keerthivasan S, Koeppen H, Hung J, Gierke S, et al. Single-cell RNA sequencing reveals stromal evolution into LRRC15+ myofibroblasts as a determinant of patient response to cancer immunotherapy. Cancer Discov. 2020;10: 232–253.

103. Domingueti C-B, Janini J-B-M, Paranaíba L-M-R, Lozano-Burgos C, Olivero P, González-Arriagada W-A. Prognostic value of immunoexpression of CCR4, CCR5, CCR7 and CXCR4 in squamous cell carcinoma of tongue and floor of the mouth. Med Oral Patol Oral Cir Bucal. 2019;24: e354–e363.

104. Kim SC, Kellett T, Wang S, Nishi M, Nagre N, Zhou B, et al. TRIM72 is required for effective repair of alveolar epithelial cell wounding. Am J Physiol Lung Cell Mol Physiol. 2014;307: L449-59.

105. Arora R, Cao C, Kumar M, Sinha S, Chanda A, McNeil R, et al. Spatial transcriptomics reveals distinct and conserved tumor core and edge architectures that predict survival and targeted therapy response. Nat Commun. 2023;14: 5029.

106. Waas M, Karamboulas C, Wu BZ, Khan S, Poon S, Meens J, et al. Molecular correlates for HPV-negative head and neck cancer engraftment prognosticate patient outcomes. Nat Commun. 2024;15: 10869.

107. Wiechec E, Magan M, Matic N, Ansell-Schultz A, Kankainen M, Monni O, et al. Cancer-associated fibroblasts modulate transcriptional signatures involved in proliferation, differentiation and metastasis in head and neck squamous cell carcinoma. Cancers (Basel). 2021;13: 3361.

108. Choi J-H, Lee B-S, Jang JY, Lee YS, Kim HJ, Roh J, et al. Single-cell transcriptome profiling of the stepwise progression of head and neck cancer. Nat Commun. 2023;14: 1055.

109. Sun Y, Chen Y, Zhao H, Wang J, Liu Y, Bai J, et al. Lactate-driven type I collagen deposition facilitates cancer stem cell-like phenotype of head and neck squamous cell carcinoma. iScience. 2024;27: 109340.

110. Chen C, Zhao S, Karnad A, Freeman JW. The biology and role of CD44 in cancer progression: therapeutic implications. J Hematol Oncol. 2018;11. doi:10.1186/s13045-018-0605-5

111. Knutson JR, Iida J, Fields GB, McCarthy JB. CD44/chondroitin sulfate proteoglycan and alpha 2 beta 1 integrin mediate human melanoma cell migration on type IV collagen and invasion of basement membranes. Mol Biol Cell. 1996;7: 383–396.

112. Hagedorn H, Schreiner M, Wiest I, Tubel J, Schleicher ED, Nerlich AG. Defective basement membrane in laryngeal carcinomas with heterogeneous loss of distinct components. Hum Pathol. 1998;29: 447–454.

113. Lim YC, Oh S-Y, Kim H. Cellular characteristics of head and neck cancer stem cells in type IV collagen-coated adherent cultures. Exp Cell Res. 2012;318: 1104–1111.

114. Liang X, Osman TA-H, Sapkota D, Neppelberg E, Lybak S, Liavaag PG, et al. Rapid adherence to collagen IV enriches for tumour initiating cells in oral cancer. Eur J Cancer. 2014;50: 3262–3270.

115. Fejza A, Camicia L, Poletto E, Carobolante G, Mongiat M, Andreuzzi E. ECM remodeling in squamous cell carcinoma of the aerodigestive tract: Pathways for cancer dissemination and emerging biomarkers. Cancers (Basel). 2021;13: 2759.

116. Wang H, Zhou H, Ni H, Shen X. COL11A1-driven epithelial-mesenchymal transition and stemness of pancreatic cancer cells induce cell migration and invasion by modulating the AKT/GSK-3β/Snail pathway. Biomolecules. 2022;12: 391.

117. Kumar KV, Hema KN. Extracellular matrix in invasion and metastasis of oral squamous cell carcinoma. J Oral Maxillofac Pathol. 2019;23: 10–16.

118. Patankar SR, Wankhedkar DP, Tripathi NS, Bhatia SN, Sridharan G. Extracellular matrix in oral squamous cell carcinoma: Friend or foe? Indian J Dent Res. 2016;27: 184–189.

119. Yang Y, Sun H, Yu H, Wang L, Gao C, Mei H, et al. Tumor-associated-fibrosis and active collagen-CD44 axis characterize a poor-prognosis subtype of gastric cancer and contribute to tumor immunosuppression. J Transl Med. 2025;23: 123.

120. Dong C, Zhao Y, Yang S, Jiao X. LINC00173 blocks GATA6-mediated transcription of COL5A1 to affect malignant development of oral squamous cell carcinoma. J Oral Pathol Med. 2023;52: 493–503.

121. Özdemir BC, Pentcheva-Hoang T, Carstens JL, Zheng X, Wu C-C, Simpson TR, et al. Depletion of carcinoma-associated fibroblasts and fibrosis induces immunosuppression and accelerates pancreas cancer with reduced survival. Cancer Cell. 2014;25: 719–734.

122. Rhim AD, Oberstein PE, Thomas DH, Mirek ET, Palermo CF, Sastra SA, et al. Stromal elements act to restrain, rather than support, pancreatic ductal adenocarcinoma. Cancer Cell. 2014;25: 735–747.

123. Vered M, Dayan D, Yahalom R, Dobriyan A, Barshack I, Bello IO, et al. Cancer-associated fibroblasts and epithelial-mesenchymal transition in metastatic oral tongue squamous cell carcinoma. Int J Cancer. 2010;127: 1356–1362.

124. Ding L, Zhang Z, Shang D, Cheng J, Yuan H, Wu Y, et al. α-Smooth muscle actin-positive myofibroblasts, in association with epithelial-mesenchymal transition and lymphogenesis, is a critical prognostic parameter in patients with oral tongue squamous cell carcinoma. J Oral Pathol Med. 2014;43: 335–343.

125. McAndrews KM, Chen Y, Darpolor JK, Zheng X, Yang S, Carstens JL, et al. Identification of functional heterogeneity of carcinoma-associated fibroblasts with distinct IL6-mediated therapy resistance in pancreatic cancer. Cancer Discov. 2022;12: 1580–1597.

126. Chen Y, Kim J, Yang S, Wang H, Wu C-J, Sugimoto H, et al. Type I collagen deletion in αSMA+ myofibroblasts augments immune suppression and accelerates progression of pancreatic cancer. Cancer Cell. 2021;39: 548-565.e6.

127. Wan Y, Hu Q, Sun K, Shi J, Liu L, Zhang X, et al. Heterogenous cancer-associated fibroblasts related tumor microenvironment marked by CD10/KLF4/TIAM1 were identified in pancreatic adenocarcinoma by integrated transcriptomics. Front Immunol. 2025;16: 1557698.

128. Hu C, Zhang Y, Wu C, Huang Q. Heterogeneity of cancer-associated fibroblasts in head and neck squamous cell carcinoma: opportunities and challenges. Cell Death Discov. 2023;9: 124.

129. Ang KK, Harris J, Wheeler R, Weber R, Rosenthal DI, Nguyen-Tân PF, et al. Human papillomavirus and survival of patients with oropharyngeal cancer. N Engl J Med. 2010;363: 24–35.

130. Nichols AC, Palma DA, Chow W, Tan S, Rajakumar C, Rizzo G, et al. High frequency of activating PIK3CA mutations in human papillomavirus-positive oropharyngeal cancer. JAMA Otolaryngol Head Neck Surg. 2013;139: 617–622.

131. Huang Q, Li F, Ji M, Lin L, Hu C. Evaluating the prognostic significance of p53 and TP53 mutations in HPV-negative hypopharyngeal carcinoma patients: a 5-year follow-up retrospective study. BMC Cancer. 2023;23: 324.

132. Almangush A, Jouhi L, Atula T, Haglund C, Mäkitie AA, Hagström J, et al. Tumour-infiltrating lymphocytes in oropharyngeal cancer: a validation study according to the criteria of the International Immuno-Oncology Biomarker Working Group. Br J Cancer. 2022;126: 1589–1594.

133. Katirachi SK, Grønlund MP, Jakobsen KK, Grønhøj C, von Buchwald C. The prevalence of HPV in oral cavity squamous cell carcinoma. Viruses. 2023;15. doi:10.3390/v15020451

134. Fonsêca TC, Jural LA, Marañón-Vásquez GA, Magno MB, Roza ALOC, Ferreira DMTP, et al. Global prevalence of human papillomavirus-related oral and oropharyngeal squamous cell carcinomas: a systematic review and meta-analysis. Clin Oral Investig. 2023;28: 62.

135. Furudate K, Matsumiya T, Hayakari R, Xing F, Kubota K, Sakaki H, et al. Clock gene expressions are altered by co-culture of gingival fibroblasts and oral cancer cells. Hirosaki Med J. 2016;66: 135–142.

136. Dudás J, Bitsche M, Schartinger V, Falkeis C, Sprinzl GM, Riechelmann H. Fibroblasts produce brain-derived neurotrophic factor and induce mesenchymal transition of oral tumor cells. Oral Oncol. 2011;47: 98–103.
